# Mechanism of lignin inhibition of enzymatic biomass deconstruction

**DOI:** 10.1186/s13068-015-0379-8

**Published:** 2015-12-21

**Authors:** Josh V. Vermaas, Loukas Petridis, Xianghong Qi, Roland Schulz, Benjamin Lindner, Jeremy. C. Smith

**Affiliations:** UT/ORNL Center for Molecular Biophysics, Oak Ridge National Laboratory, 37831 Oak Ridge, TN USA; Center for Biophysics and Quantitative Biology, University of Illinois at Urbana-Champaign, 61801 Urbana, IL USA; Department of Biochemistry and Cellular and Molecular Biology, University of Tennessee, 37996 Knoxville, TN USA; University of Tennessee/Oak Ridge National Laboratory Center for Molecular Biophysics, P.O.Box 2008, Oak Ridge, TN 37831-6309 USA

**Keywords:** Biofuel, Lignin, Cel7A, Cellulose crystallinity

## Abstract

**Background:**

The conversion of plant biomass to ethanol via enzymatic cellulose hydrolysis offers a potentially sustainable route to biofuel production. However, the inhibition of enzymatic activity in pretreated biomass by lignin severely limits the efficiency of this process.

**Results:**

By performing atomic-detail molecular dynamics simulation of a biomass model containing cellulose, lignin, and cellulases (*Tr*Cel7A), we elucidate detailed lignin inhibition mechanisms. We find that lignin binds preferentially both to the elements of cellulose to which the cellulases also preferentially bind (the hydrophobic faces) and also to the specific residues on the cellulose-binding module of the cellulase that are critical for cellulose binding of *Tr*Cel7A (Y466, Y492, and Y493).

**Conclusions:**

Lignin thus binds exactly where for industrial purposes it is least desired, providing a simple explanation of why hydrolysis yields increase with lignin removal.

**Electronic supplementary material:**

The online version of this article (doi:10.1186/s13068-015-0379-8) contains supplementary material, which is available to authorized users.

## Background

Sustainable global economic growth requires the development of technologies that will reduce the environmental footprint of energy consumption, including the adoption of renewable, energy-dense transportation fuels [1]. The production of biofuels from abundant lignocellulosic biomass is a potential alternative to fossil fuels. However, a significant barrier to cost-effective cellulosic biofuel production is the current inefficient hydrolysis of cellulose glycosidic bonds to fermentable sugars by cellulase enzymes [2–4].

Cellulose hydrolysis by cellulases is typically preceded by thermochemical pretreatment of biomass to increase the accessibility of the cellulose substrate to the enzyme. Dilute acid pretreatment removes almost all biomass components apart from the cellulose itself and lignin [5–7], a poly-aromatic amorphous and hydrophobic plant polymer [8]. However, even after pretreatment, enzymatic cellulose hydrolysis remains incomplete [9]. Overcoming this inefficiency presents one of the most important challenges in biotechnology [2–4, 10–13].

There is considerable evidence implicating lignin as a major culprit in reducing cellulase efficiency in pretreated biomass [3, 14–23], though its mechanism of action has not been definitively elucidated. Various lignin-related inhibitory processes have been proposed, including cellulose association with lignin, blocking enzymatic access to cellulose [15–18], and the unproductive binding of the enzymes to lignin [19–23]. Unproductive binding has been proposed to be non-specific and to occur via hydrophobic  [19, 22, 23] or electrostatic interactions [24–26], although no direct evidence has been observed for either hypothesis. It is also suspected that the cellulose-binding module (CBM) of cellulases participates in lignin binding, as enzymes containing a CBM have a higher affinity for lignin than those without one [20, 22]. However, an atomic-detailed characterization of how cellulases become inhibited by lignin is currently lacking.

In order to rationally design improved pretreatment processes which minimize the lignin’s adverse effect in biofuel production and guide current developments in lignin bioengineering, it is important to understand mechanistically how lignin interferes with cellulose degradation [27–29]. Here, we report molecular dynamics (MD) simulations of a model of a pretreated multi-component biomass system, containing lignin, cellulose fibers of different degrees of crystallinity, and the industrially important [30–32] *Trichoderma reesei* fungal cellulase (*Tr*Cel7A) enzyme. The simulation system models the crowded lignocellulosic environment in which *Tr*Cel7A operates during industrial biomass hydrolysis. The results indicate that lignin associates preferentially with the hydrophobic surface of cellulose, which is also the preferred substrate of *Tr*Cel7A. Lignin is also found to bind preferentially to the CBM tyrosine residues 466, 492, and 493, which have been identified as being critical to cellulose binding [33–38]. Thus, lignin directly and competitively inhibits the recognition mechanism of the cellulase consistent with a competitive inhibition mechanism previously postulated by mutagenesis work and biochemical assays [9, 25, 39]. These atomistic details of the interaction of a cellulase within a crowded biomass environment, including both substrate interactions and lignin inhibition, explain why lignin is such an effective barrier to efficient enzymatic hydrolysis of post-pretreated biomass.

## Results and discussion

**Fig. 1:**
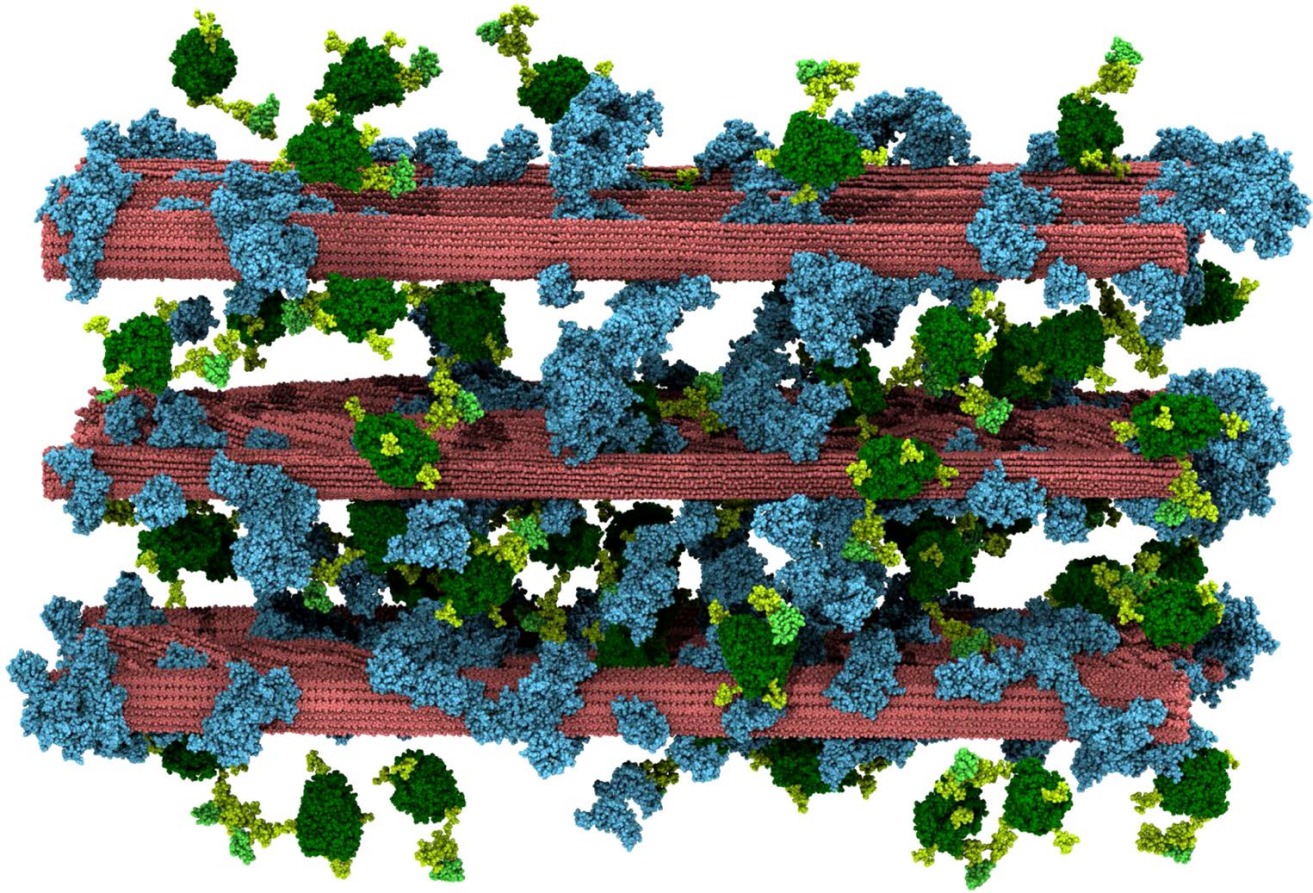
Side view of the initial state of the lignocellulosic biomass system. Cellulose fibrils are *red*, lignin molecules *blue*, and *Tr*Cel7A enzymes *green*; the CBMs have a lighter color than the CDs, while glycosylations and linker regions are in *pastel green*. An animation from this starting structure is given as an Additional file 1

The simulation specifically investigates the binding of Cel7A to cellulose prior to the enzyme hydrolyzing a glucan chain, and how this binding is affected by the presence of lignin. The simulation model was devised to represent a pretreated biomass system of cellulose and lignin at room temperature upon the addition of cellulolytic enzyme. Other components of biomass, such as pectins and hemicellulose, are assumed to have been removed [5]. As detailed in Sect. “Methods,” a large variety of experimental data was used to construct a realistic model. The simulation system consisted of nine cellulose fibers, of which six were crystalline and the other three non-crystalline [40], 54 glycosylated *Tr*Cel7A enzymes, and 468 lignin molecules in explicit solvent. In the starting structure of the system, i.e., prior to the simulation (Fig. 1), no enzymes are bound to the biomass, but there is extensive cellulose–lignin association derived from previous simulations of pretreated biomass [40] which remained virtually unchanged after the addition of the enzymes in the current study. Three different cellulose fiber–lignin distribution combinations were present in the simulation: CH (crystalline cellulose, high lignin coverage), CL (crystalline cellulose, low lignin coverage), and NonC (non-Crystalline cellulose, low lignin coverage). These combinations are analyzed independently throughout the text when clear differences were found in the properties observables studied.

### Network formation

**Fig. 2:**
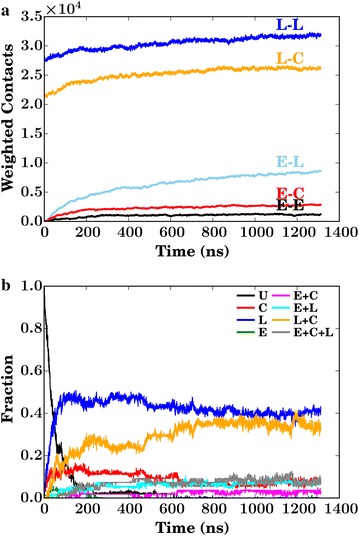
**a** Contact counts as a function of time between enzyme E, lignin L, and cellulose C molecules. **b** Time traces of the fraction of the 54 enzymes that are unbound, *U*; bound only to cellulose, C; bound only to lignin, L; bound only to other enzymes, E; bound to enzymes and cellulose, *E+C*; bound to enzymes and lignin, *E+L*; bound to lignin and cellulose, *L+C*; bound to other enzymes, lignin and cellulose, *E+C+L*. In this analysis, an enzyme is said to be bound if any of its heavy atoms are within 3.2 Å of a heavy atom in another molecule

The intermolecular contacts, a measure of binding thermodynamics and defined in Eq. 1, indicate that during the simulation the degrees of lignin–lignin and lignin–cellulose association do not vary significantly (Fig. 2a), as would be expected for the pre-equilibrated lignocellulose fibrils used here. As the simulation progresses a gradual increase in the number of enzymatic contacts is observed as the enzymes diffuse to the lignocellulose. However, all enzymes are bound to another interaction partner within 600 ns (Fig. 2b), so the growth in the number of enzyme–lignin contacts seen over the second half of the simulation in Fig. 2a arises from enzymes that are already bound optimizing their interfacial area with the lignin.

The cellulases overwhelmingly interact with either only lignin or both lignin and cellulose. Together, these equally large populations account for approximately 80 % of all enzymes (Fig. 2b). This corresponds to 160 mg of protein bound to 1 g of biomass “solids” (cellulose and lignin), in broad agreement with the experimentally determined cellulase binding capacity of thermochemically pretreated biomass systems, which is 160 mg/g for Douglas-fir softwood [41], 170 mg/g for poplar [42], and 140–150 mg/g for corn stover [7, 43].

**Fig. 3:**
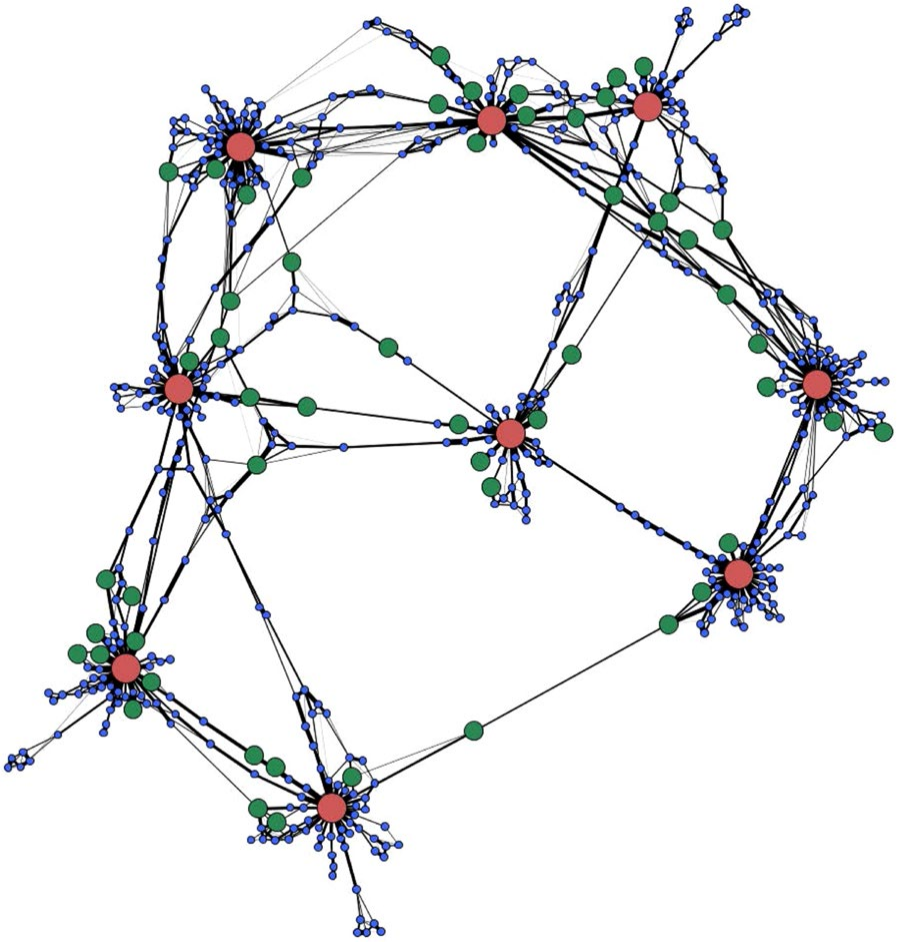
Schematic representation of the network formed by the individual biomass components at the end of the simulation. Each *circle* represents one element of the system: the large *red circles* are for cellulose fibrils, the *small blue circles* are for lignin molecules, and the intermediate *green circles* are for *Tr*Cel7A enzymes. The *black lines* connecting the components indicate a contact between two components, and the thickness represents the degree of contact (the contact number). The position of the individual particles is arbitrary, with the position determined using the ForceAtlas algorithm of Gephi [45], which treats the connection as springs connecting the elements. An animation of the time-evolution of this representation is given as an Additional file 3

The cellulase interactions do not take place in isolation, but rather are part of a crowded mesh formed by the superstructure formed by the biomass constituents (Fig. 3). This shows that lignin mediates the formation of a fully interconnected network of cellulose, lignin, and *Tr*Cel7A, with each molecule linked to all others directly or indirectly. These networks arise spontaneously in the simulations, and are only possible due to the simulation incorporating multiple cellulose fibrils. Within the network, cellulose fibrils act as hubs, i.e., have numerous connections to other molecules. *Tr*Cel7A and lignin acts as a “glue” connecting these hubs. Within the network, lignin’s role depends on its morphology. We identify three types of lignin aggregates (Additional file 2: Figure S1): “sheets,” in which lignin monolayers bind to a single cellulose fiber; “piles,” in which the lignin aggregates onto a single cellulose fibril but not as a monolayer; and “linkages,” in which the lignin aggregates connect cellulose fibrils. If lignin adopts an extended morphology (a sheet or linkage), more surface is exposed, and lignin’s propensity to bind to enzymes is increased (Table S1). Therefore, piles are the least effective at trapping enzymes and hence the least inhibitory to cellulase action. It has been shown that increasing the hydrophobicity of lignin reduces its radius of gyration thereby making it more compact [44], which may favor pile formation over other lignin morphologies.

An implication of the existence of lignin-mediated networks is the retardation of enzyme diffusion due to confinement. Indeed, binding to cellulose or lignin leads to a three orders of magnitude slowdown in enzyme translational diffusion, decreasing from an initial ${\sim }10^{-6}$ cm$^2$/s to a final ${\sim }10^{-9}$ cm$^2$/s, and one order in rotational diffusion, from ${\sim }10^6$ to ${\sim }10^5$ rad$^2$/s (Additional file 2: Figure S2). In comparison, the translational diffusion coefficient of proteins in living cells is ${\sim }10^{-7}$ cm$^2$/s [46] and that of bound cellulases processing on a cellulose surface is ${\sim }10^{-10}-10^{-11}$ cm$^2$/s [47].

### Cellulase binding to cellulose in the presence of lignin

**Fig. 4:**
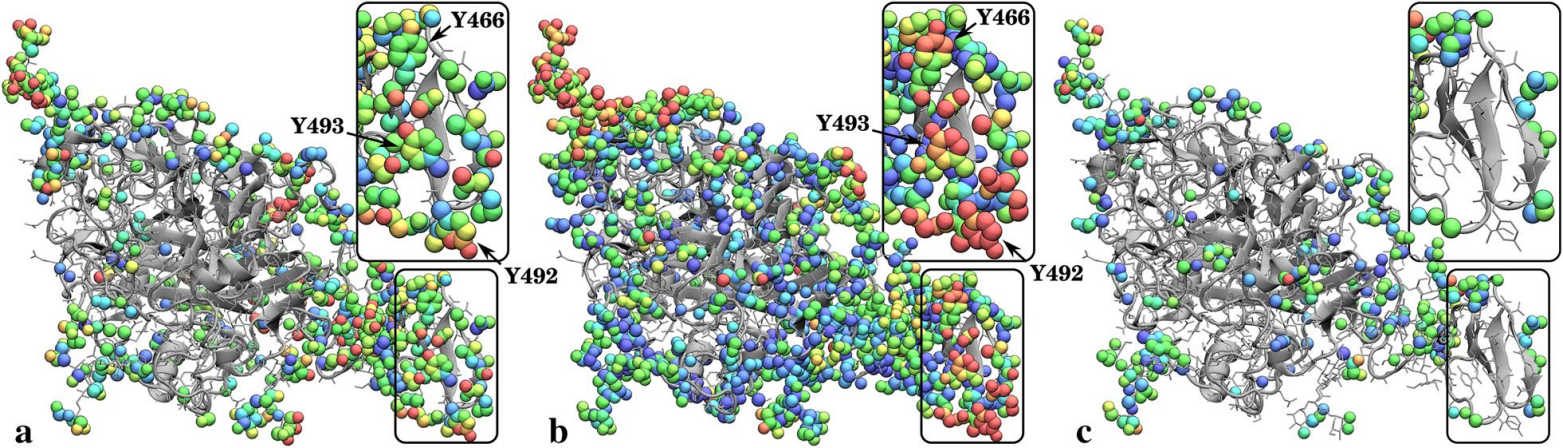
Number of contacts, averaged over all enzymes and over the last 300 ns of simulation, at the end of the simulation of *Tr*Cel7A with cellulose (**a**), lignin (**b**), and other enzymes (**c**) mapped onto a model of *Tr*Cel7A. Cooler (*blue*) colors indicate fewer contacts, while warmer (*red*) indicate more. These figures are also available as Additional file 4: Video S1; Additional file 5: Video S2; Additional file 6: Video S3 as well as downloadable pdb files where the contact number is in the beta column (Additional files 7, 8 and 9)

Cellulase binding to cellulose is the first step of the mechanism of enzymatic deconstruction. *Tr*Cel7A possesses a typical cellulase multidomain organization, with a large catalytic domain (CD) connected to a CBM via a flexible linker. The enzyme possesses posttranslational modifications, in which the linker is highly *O*-glycosylated and the CD *N*-glycosylated [32, 48]. To obtain a molecular-level description of this binding in the presence of lignin we determined the propensity of the individual enzyme residues to participate in cellulose-*Tr*Cel7A binding and mapped them onto the *Tr*Cel7A structure (Fig. 4a; Additional file 4: Video S1; Additional file 5: Video S2; Additional file 6: Video S3; Table 1). From Fig. 4a, two regions stand out as forming the most contacts to cellulose: three Tyr CBM residues and the linker glycosylation sugars. The linker glycosylations have been previously demonstrated to interact with cellulose [32], although their physiological role has not been fully elucidated. The linker has been suggested to convey resistance to proteolysis [49], increase protein solubility [50], minimize contact between the CD and the CBM [51], and promote binding to cellulose [32]. Here, the glycosylations are found to participate significantly in *Tr*Cel7A binding not only to cellulose, but also to lignin and other *Tr*Cel7A molecules (Table 1).

**Table 1:**
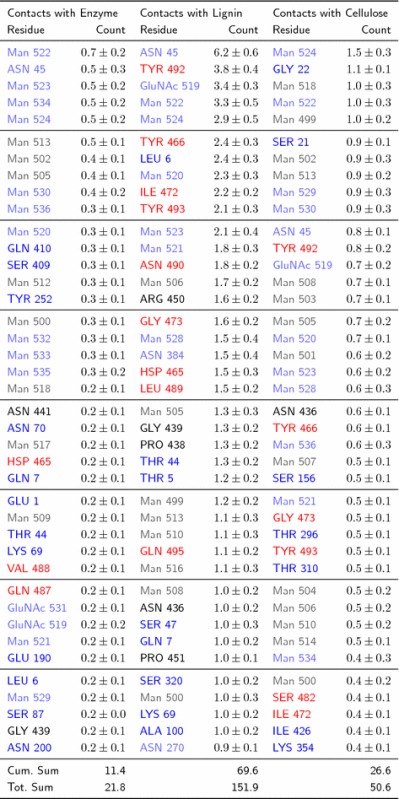
40 residues of Cel7A interacting most frequently with other enzymes, lignin and cellulose

The values are the average number of contacts a cellulase residue makes with other cellulases (enzyme), lignin and cellulose for simulation times $t> 1000\ \mathrm{ns}$

The residues are color-coded based on their location within Cel7A

Blue text indicates a residue that is part of the CD, red of the CBM and black of the linker

Lighter text in lower case indicates a glycosylation (sugar) monomer, while bold upper case is an amino acid residue

The cumulative sum of the top 40 contacts and the total sum of all contacts are reported in the final two rows

The flat hydrophobic surface on the CBM formed by three tyrosine residues (Y466, Y492 and Y493) promotes binding to the hydrophobic surfaces of cellulose fibers [33–38]. In the present simulations, the tyrosine residues form extensive contacts with the lignin. Indeed lignin outcompetes cellulose in terms of interacting with these residues (Table 1; Additional file 10).

However, in over half of the trajectories individual enzymes form interactions with the cellulose substrate. Among the 30 enzymes that bind to cellulose within our simulation, there are many that have their substrate tunnel aligned perpendicular to the fibril axis, some of which are only loosely connected via glycosylations to the fibril. A full gallery of all of these interactions is available as an Additional file 11. From our sampling, there are more cases where the substrate tunnel is aligned parallel to the cellulose fibril than where it is anti-parallel (Additional file 2: Figure S4). The observed preference toward a parallel orientation would facilitate processive binding, although we can identify no clear mechanism as to the origins of the preferential parallel orientation. It is possible that this orientation is enforced by the directionality of the CBM, as has been previously postulated [38, 52]. However, given how few CBMs are actually bound to cellulose (see the gallery available online provided as Additional file 11), this cannot be determined based on our simulations.

### Cellulose association with lignin

**Fig. 5:**
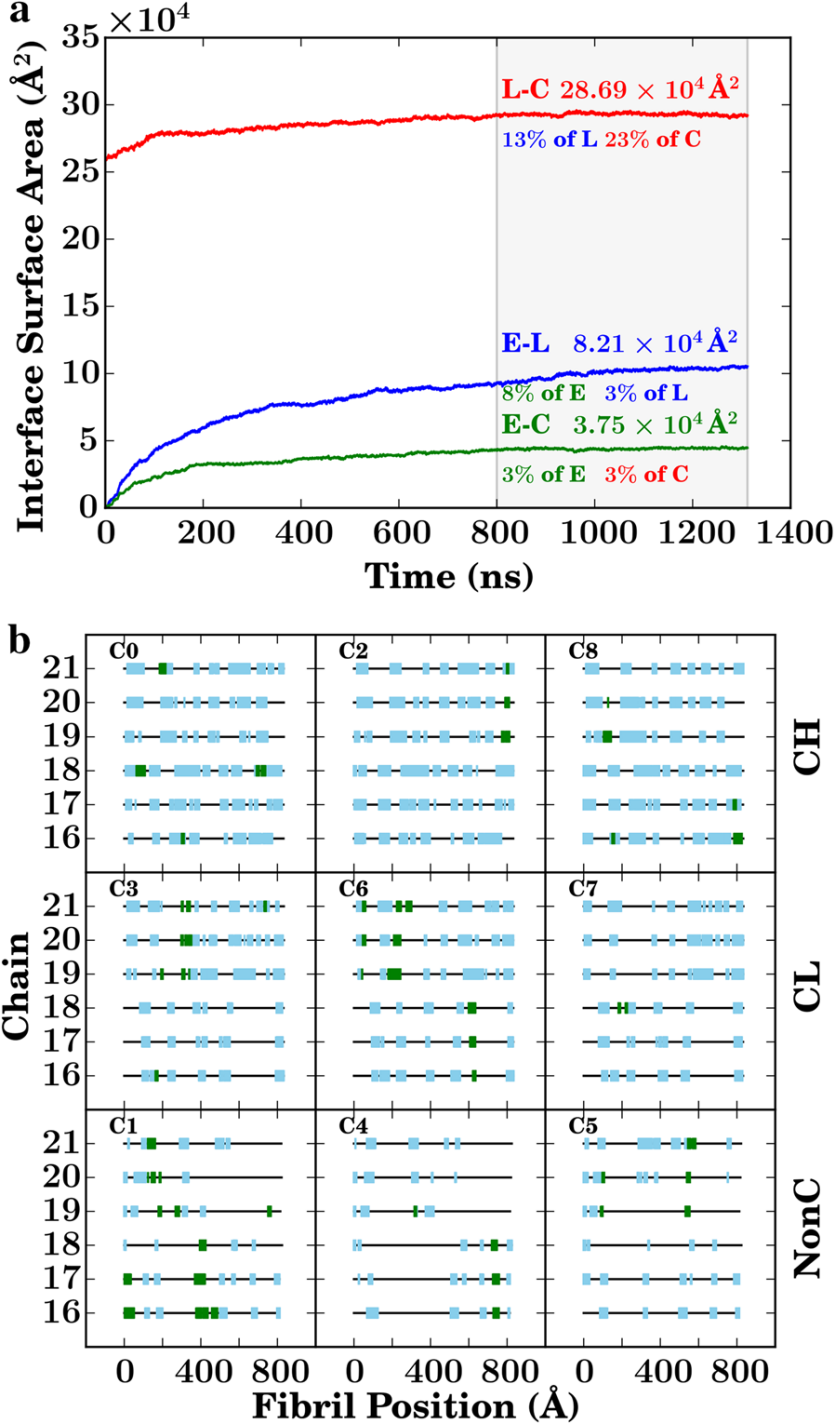
**a** Interface surface area for cellulose (*C*), lignin (*L*), and enzymes (*E*), their means values (for $t>800$ ns) labeled above the *curves*. The % fraction of interface area over the total surface area of a species is also labeled below the *curves*. **b** Pictorial representation of the final configuration of the simulation, showing the positions of lignins (*blue*) and enzymes (*green*) on the hydrophobic surface of the nine cellulose fibrils (*black line*). The average “procession length” (distance along the fibril between two lignin clusters) depends on the type of fibril. CH fibrils have the shortest procession lengths (3.5 nm), CL fibrils intermediate (5.5 nm), and NonC the longest (9.2 nm)

The cellulose surface is crowded. Nearly a quarter of the total cellulose surface area is consistently covered by lignin, significantly reducing the area accessible to the enzymes (Fig. 5a). In addition, the presence of lignin molecules on the cellulose surface is likely to interfere with the processive mechanism of cellulose hydrolysis [31], reducing the distance an enzyme bound to cellulose can travel before its path is blocked by a lignin molecule (Fig. 5b).

**Fig. 6:**
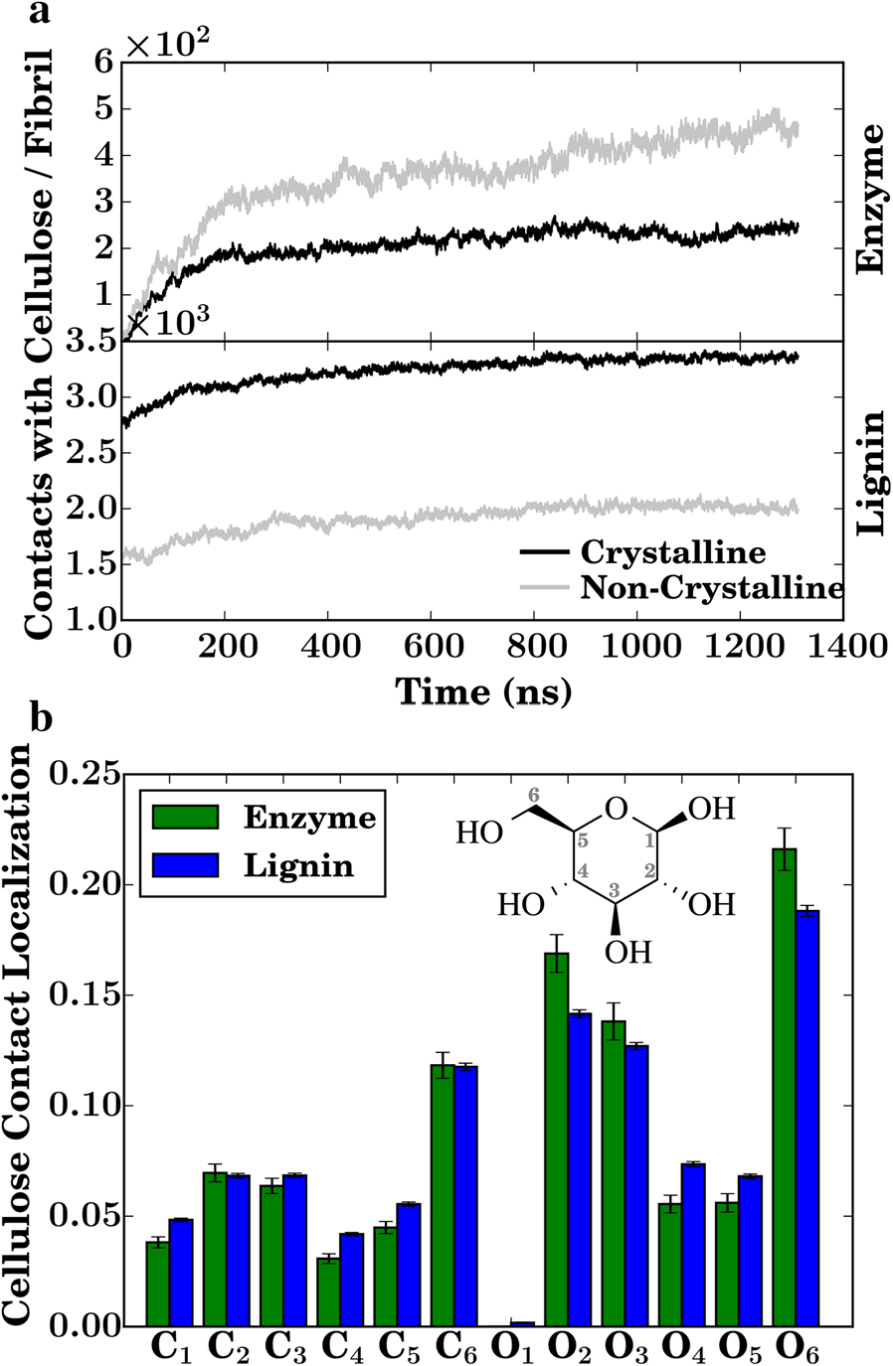
**a** Contacts per fibril of crystalline and non-crystalline cellulose with the enzyme and with lignin. **b** Normalized number of contacts between any specific cellulose heavy atom and lignin and enzymes

Non-crystalline cellulose was engaged in twice as many contacts with the enzyme per fibril than does the crystalline polymer (Fig. 6a), which may be due in part to a reduced affinity of non-crystalline cellulose for lignin [40]. The reduced affinity in turn increases the surface area available for enzymatic binding, and in fact the non-crystalline cellulose surface has comparatively little lignin coverage (Table 2). A second factor favoring enzymatic binding to non-crystalline cellulose is the accessibility of surface cellulose hydroxyl groups, which account for more than half of the cellulose–enzyme contacts (Fig. 6b); a larger fraction of these is buried in crystalline cellulose than in the non-crystalline form. Due to the lower lignin coverage of non-crystalline cellulose, enzymes can, in principle, process this form for a larger distance before being blocked by lignin (Fig. 5b).

**Table 2:** Total fibril cellulose surface area ($$A_T$$), cellulose–enzyme contact area ($$A_E$$), cellulose–lignin contact area ($$A_L$$), and their corresponding ratios ($$A_E/A_T)$$ and ($$A_L/A_T$$) for the three initial cellulose–lignin fibril combinations: CH (crystalline cellulose, high lignin coverage), CL (crystalline cellulose, low lignin coverage), and NonC (non-crystalline cellulose, high lignin coverage)

	$A_T$	$A_E$	$A_L$	${A_E}/{A_T}$	${A_L}/{A_T}$
	$$(10^4$$ Å$$^2)$$	$$(10^4$$ Å$$^2)$$	$$(10^4$$ Å$$^2)$$		
CH	***6.51***	***0.18***	***3.02***	***0.03***	***0.46***
	*6.74*	*0.15*	*1.33*	*0.02*	*0.20*
CL	***6.51***	***0.23***	***2.11***	***0.04***	***0.32***
	*6.74*	*0.24*	*0.97*	*0.04*	*0.14*
NonC	***6.57***	***0.30***	***1.16***	***0.05***	***0.18***
	*7.76*	*0.36*	*1.17*	*0.05*	*0.15*

Rows with "bold italic" background correspond to hydrophobic surfaces, while those with "italic" background correspond to the hydrophilic cellulose surfaces

The quantities reported here are the averages over the last 500 ns

**Fig. 7:**
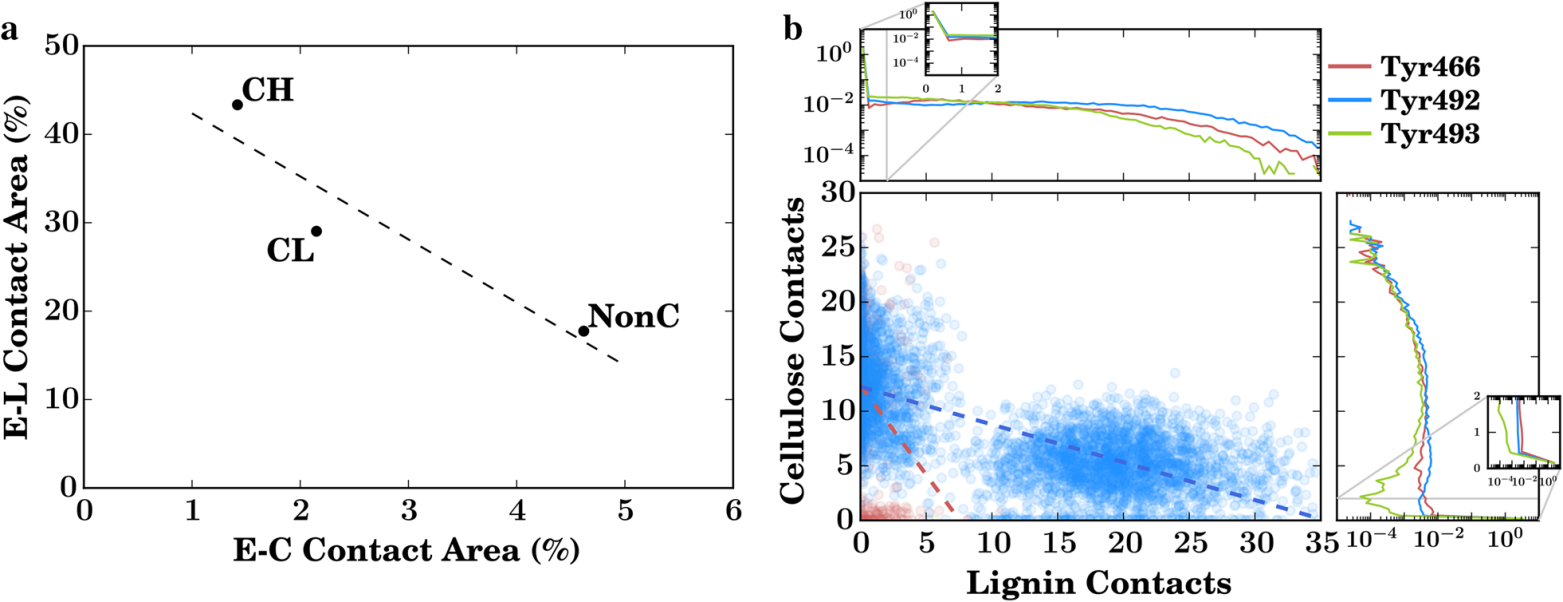
**a** Fraction of hydrophobic cellulose covered by lignin and enzymes per cellulose fibril type. Individual fibril types are labeled. The* dotted line* is a linear regression to the data. This contains the same information as Table 2. **b** Comparison of the number of simultaneous contacts between the specific CBM tyrosine residues, with a scatterplot in the main panel, and log-probability distributions for direct comparisons along each axis

Chains of crystalline cellulose on hydrophobic surfaces can be more readily decrystallized than those on hydrophilic surfaces [53]. The present simulations reveal a preferential association of both lignin and the enzymes with the hydrophobic face of the cellulose fibers (for a chain-by-chain analysis see Additional file 2: Figure S5). Lignin contacts lead to the hydrophobic chains of crystalline cellulose being only poorly accessible, with 30–40 % of their total surface area covered by lignin and only ${\sim }3\,\%$ covered by enzymes (Table 2). In contrast, in the non-crystalline fibers, the lignin contact area with the “hydrophobic” face is reduced by about half to ${\sim }18\,\%$, while the proportion in contact with cellulases nearly doubles (Table 2). Moreover, the trend line between lignin and enzyme coverage of cellulose for the hydrophobic faces (Fig. 7a) has a negative slope, confirming competitive binding.

**Fig. 8:**
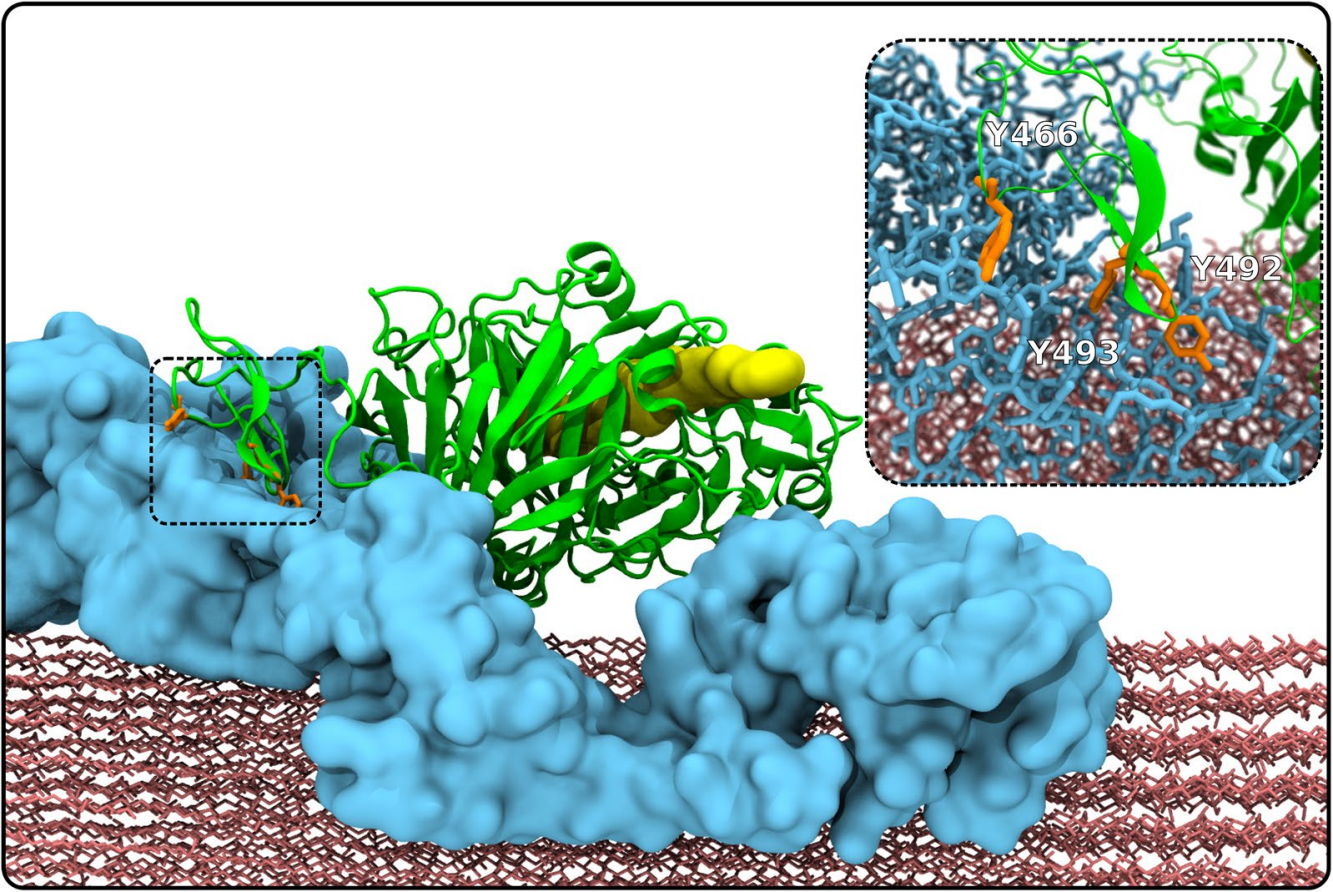
Snapshot of the simulation in which *Tr*Cel7A (*green cartoon*) is bound unproductively to a lignin cluster (*blue surface*) on a cellulose fiber (*red*). The CBM residues Y466, Y492, and Y493 are *orange*. The location CD catalytic tunnel is shown by a *yellow* *spacefilling* representation, and is provided for reference. No cellulose was within the tunnel at any point during the simulation, as the complete fibrils did not decrystallize. The *inset* is an enlarged image delineated by the *dotted rectangle*, which highlights the Tyr (*orange*)–lignin (*blue*) interactions. A gallery of images showing the cases where *Tr*Cel7A enzymes interact with cellulose are provided in the supplementary information†

### Unproductive binding of enzyme to lignin

**Fig. 9:**
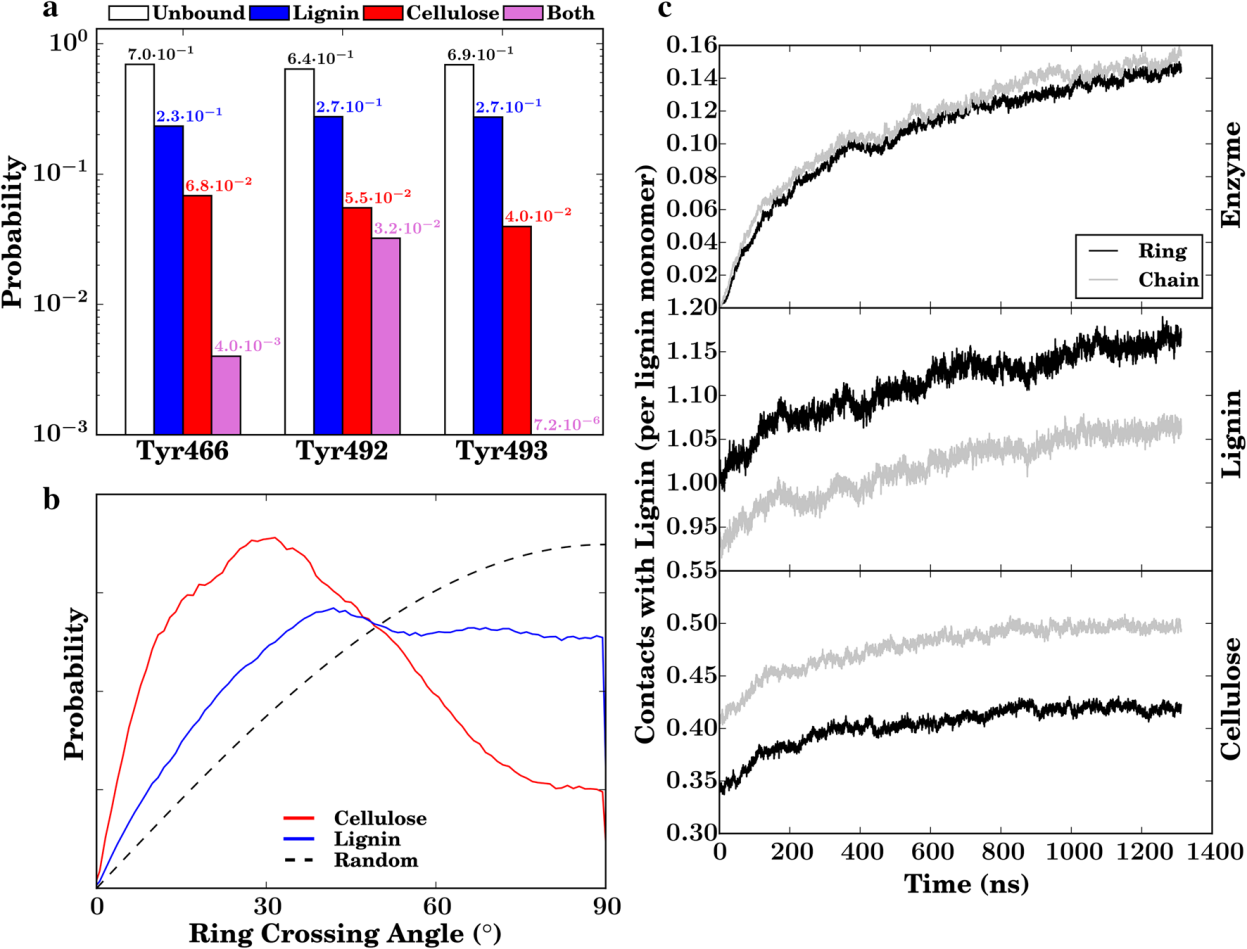
**a** Probabilities of the three CBM Tyr residues (466, 492, and 493) being contact in contact to only lignin, only cellulose, both lignin and not bound to either (unbound). **b** The crossing angle between the ring normals of the three CBM Tyr residues (466, 492, and 493) and the closest (within 5 Å) biomass ring (the glucose ring of cellulose or the phenolic ring of lignin). The *dotted lines* are distributions that would be obtained without an angular energetic preference from a random distribution. **c** Number of contacts per lignin residue with the enzyme (*top*), other lignins (*middle*), or cellulose (*bottom*). Contacts are labeled as “ring” when involving the lignin atoms C$_1$–C$_6$, O$_3$, O$_4$, and C$_{10}$, while “chain” involves atoms C$_7$–C$_9$, O$_7$–O$_9$

Enzymes that bind irreversibly to lignin are prevented from binding to their cellulose substrate, such as the example configuration shown in (Fig. 8). The most probable lignin–enzyme contacts involve either CBM residues or glycosylation sugars on the CD (Fig. 4b and Table 1). Three CBM tyrosine residues (Y466, Y492, Y493) that are known to recognize and bind to cellulose [33–38] play an outsized role in the lignin–enzyme association process. In the simulations, the probability of these residues binding to lignin is approximately five times higher than their binding to cellulose (Fig. 9). Figure 7b also indicates that, for the most part, the CBM Y466 and Y493 residues interact exclusively with either lignin or cellulose due to geometrical constraints, further suggesting that binding to lignin indeed impedes binding to cellulose. This is shown in another way in Fig. 7, which demonstrates that an individual residue is only rarely in contact with both lignin and cellulose. Taken together, these findings imply a competitive inhibition mechanism of *Tr*Cel7A, in which the binding of lignin to the CBM Tyr residues prevents cellulose recognition.

To obtain further information on the Tyr-lignin binding we examined the stacking interactions of the aromatic side chains of the Tyr residues as determined by the angle $\gamma$ between the planes of the tyrosine and the lignin/cellulose rings [54]. For the Tyr-cellulose stacking, the two rings are almost parallel, with a relatively narrow distribution peaked at $\gamma \, {\simeq }\, 30^{\circ }$ that deviates from that that would be obtained in the absence of an angular energetic preference (Fig. 9b) [54]. However, for the interaction of the Tyr residues with the phenolic rings of lignin $\gamma$ has a broader distribution, which is more similar to what would be expected if there were no intrinsic angular energetic preference. This suggests enthalpy plays a more significant role in determining the orientation preferences of Tyr–cellulose than Tyr–lignin interactions.

It has been suggested that enzymes may become denatured on the lignin surface [9]. However, in the ${\sim }$µs timescales examined here, no clear trend was observed between the average residue root mean square fluctuation, an approximate measure of the propensity to denature, and the number of residue-lignin contacts (Additional file 2: Figure S6). Rather than denaturing, the enzymes compact to a mean radius of gyration of $24.8\pm 1.0$ Å (Additional file 2: Figure S7) over the course of the simulation, in line with experimentally determined radius of gyration for Cel7A in solution of $26.1\pm 2.1$Å [55].

We find that the interactions lignin makes with other lignin molecules, cellulose, and cellulases are qualitatively different. Although lignin is hydrophobic overall due to its phenolic rings, monolignols also contain a flexible three-carbon (C$_7$–C$_9$) chain with hydroxyl groups (Additional file 2: Figure S8). Inter-lignin association is dominated by interactions between the rings, defined here as involving atoms C$_1$–C$_6$, O$_3$, O$_4$ , and C$_{10}$ (Fig. 9c; Additional file 2: Figure S8). In contrast, enzyme association with the lignin flexible chains (C$_7$–C$_9$ and O$_7$–O$_9$) is as frequent as with the lignin rings. Finally, when associating with cellulose lignin interacts mostly via its flexible chain atoms. Thus it is not simply a matter that either ring-mediated hydrophobic [19, 22, 23] or hydroxyl-mediated electrostatic interactions[24–26] that drive unproductive binding to lignin, but rather both elements contribute to the overall binding.

## Conclusions

Atomistic MD simulations of a multi-component system of cellulose, lignin, and an industrially important cellulase, *Tr*Cel7A, described here have led to a mechanistic understanding of how lignin in biomass systems impedes binding of cellulase enzymes to cellulose, thus hindering hydrolysis. Lignin is known to directly associate with cellulose and restrict its hydrolysis by cellulases [15, 16, 18]. The present simulations confirm the binding of lignin to cellulose, which decreases both the surface area available for enzymatic binding (Figs. 5a, 6a) and the length of the cellulose chain that can be processed before a lignin blocks its path (Fig. 5b) [18, 31]. Furthermore, lignin is found to bind preferentially to the hydrophobic faces of cellulose (Table 2), as does *Tr*Cel7A  [36, 56], amplifying the inhibitory effect. Importantly, the relationship between lignin and enzymatic binding (Fig. 7a) indicates a competitive binding mechanism, in which both enzyme and inhibitor (lignin) bind favorably to the substrate (cellulose). The simulations thus establish a link between cellulose accessibility to cellulases, a key physical property influencing pretreated biomass hydrolysis [57], and cellulose–lignin association.

Secondly, *Tr*Cel7A is also known to bind unproductively to lignin, further limiting its ability to hydrolyze cellulose [19–23]. The present simulations confirm this and provide atomic details of the interactions. Lignin forms specific interactions with those Tyr residues (Y466, Y492 and Y493) on the CBM that have been shown to anchor the enzyme to its cellulosic substrate (Fig. 8; Table 1). The relationship between Tyr binding to lignin and cellulose (Fig. 7b) indicates a second mechanism for competitive inhibition, in which specific binding of the inhibitor (lignin) to the recognition site on the enzyme (CBM) blocks the enzyme substrate binding. The Tyr–lignin interactions may be particularly difficult to engineer away in the enzyme, as mutations to the CBM that might disrupt the interaction with lignin will likely also reduce the affinity of the CBM for cellulose. Engineering the lignin within biomass may be a better approach, possibly by making it more hydrophobic such that it compacts [44] and presents a smaller interaction surface area.

In conclusion, the present study furnishes a detailed description of interactions of a cellulase in a model crowded, pretreated, lignocellulosic environment. Lignin impedes enzymatic action by two competitive binding processes, the molecular bases of which are described here: binding to the hydrophobic face of cellulose, the preferred substrate of *Tr*Cel7A; and specific binding to the tyrosine residues of the CBM that recognize and bind cellulose. Lignin thus binds exactly where for industrial purposes it is least desired, providing a simple explanation why hydrolysis yields increase with lignin removal. These findings explain why lignin is so effective at blocking cellulose hydrolysis by *Tr*Cel7A. This molecular-level description may be used to rationally optimize biofuel production processes which minimize lignin interference. This could, for example, be achieved by pretreatments that lead to non-crystalline cellulose, which associates less with lignin than the crystalline form.

## Methods

### Model

A 23.7-million atom, multi-component simulation model was build to represent a pretreated biomass system of cellulose and lignin at room temperature upon the addition of cellulolytic enzyme. The model consists of cellulose fibers, lignin molecules, and Cel7A cellulases. Other components of biomass, such as pectins and hemicellulose, are assumed to have been removed by dilute acid pretreatment [5].

Hexagonal cellulose fibers were constructed, each containing 36 glucose chains [58] of degree of polymerization (d.p.) 160. Pretreated cellulose has a d.p. ${\gtrsim }140$ [59]. Since cellulose in pretreated biomass exists in both highly crystalline and more amorphous forms, both types of fibers were modeled: six crystalline fibers, obtained from the crystal structure of cellulose $I{\beta }$ [60]; and three non-crystalline, obtained by simulating crystalline cellulose at 650 K for 1 ns [40].

468 lignin molecules (52 per cellulose fibril) were included, comprising 18 copies each of 26 distinct lignin molecules obtained from previous studies [61, 62]. All lignin molecules consisted of 61 monolignol monomers, and the lignin molecular weight, degree of branching, monomer, and linkage composition are consistent with those of softwood lignin [61]. Briefly, structural models of the individual lignin molecules were generated by first deriving the bonding topologies of the molecules and subsequently generating the 3D coordinates. To generate the topologies, a variety of experimental data on the bulk chemical composition of softwood lignins was used. Softwood lignins are composed mainly of G units [63–65] and therefore only G units were used here. The average linkage composition used is typical of softwoods [65, 66]: $\beta$-*O*-4${^{\prime }}$ 50 %, 5-5${^{\prime }}$ 30 %, $\alpha$-*O*-4${^{\prime }}$ 10 %, and $\beta$-5${^{\prime }}$ 10 %. The models also contain equal amounts of left- and right-handed $\beta$-*O*-4${^{\prime }}$, $\alpha$-*O*-4${^{\prime }}$ and $\beta$-5${^{\prime }}$ linkages, so as to make the molecules optically inactive, in accord with experiment [67]. Each molecule comprised 61 G units leading to a molecular weight of 13 kDa, within the experimentally determined range [68]. Finally, an average crosslink density of 0.052, or 3.2 branch points per 61 monomers, was used, again as has been derived experimentally, for spruce wood [69]. The number of branch points per molecule and their location along the chain were assigned randomly using a computer algorithm: the resulting 26 distinct lignin topologies have varying degrees of branching: one molecule has zero branch points, three have one, four have two, six have three, seven have four, three have five, and one molecule has six.

Subject to the constraints imposed by the above experimental data, random primary structures of lignins were generated, producing 25 molecules that are different from each other but consistent with the average chemical properties of softwood lignin. For example, although for all 26 molecules $50\,\%$ of linkages are of the $\beta$-*O*-4${^{\prime }}$ kind, the positions of these linkages varies between molecules, as does the position of the branch points, and the lengths of the branches are different. Relaxed 3D structures for the lignin molecules were obtained from previous simulations [40].

The starting lignin and cellulose coordinates were obtained from the final state of previous MD simulations of pretreated lignocellulose, in which 52 lignin molecules aggregated on the surface of individual cellulose fibers [40]. Three states were used here, obtained from the end states of three prior simulations [40]: crystalline cellulose with high lignin coverage (CH), crystalline cellulose with low lignin coverage (CL), and non-crystalline cellulose with low lignin coverage (NonC). (In our previous work, CH, CL, and NonC were denoted NC, FC, and FN, respectively [40]). Nine cellulose fibers (and the lignin molecules associated with them) were placed parallel to each other, such that all cellulose fibers (three NC, three FC, and three NonC) have the same neighbors when periodic boundary conditions are applied.

54 identical *tr*Cel7A enzymes were constructed using the crystal structure of the catalytic domain [70] and the NMR structure of the CBM [33]. The linker sequence was built as a linear segment connecting the two domains. *N*-glycans were attached to residues 45, 270, and 384 of the catalytic domain, and *O*-glycans were attached to the linker, in a manner consistent with experimental data [48, 71]. This glycosylation pattern is that suggested by mass spectrometric methods [48, 71]. The 54 enzymes were placed in the unoccupied space of the simulation box using a local algorithm that randomly varied their positions and orientations until placements were achieved without steric clashes with other macromolecules already in the system. The system was solvated by 7.1 M water molecules and was subsequently neutralized using Na ions.

The relative mass ratio $R_{c:l}$ of cellulose to lignin is 1.5 g cellulose per g of lignin, which is typical of thermochemically pretreated biomass: $R_{c\,l}\approx 1.8-1.9$ for pretreated corn stover [7, 72, 73], $R_{c\,l}\approx 1.7$ for pretreated switchgrass [74], $R_{c\,l}\approx 1.2$ for pretreated poplar [75], and $R_{c\,l}\approx 0.9-1.2$ for pretreated pine [76, 77]. Overall, the absolute concentration of the solutes was higher than in typical enzyme binding experiments. For example, the cellulose concentration was 60 g/L (6 % w/v), while that commonly employed in enzyme binding is typically ${\sim }$10 g/L (1 % w/v) [7, 42, 77]. The enzyme loading corresponds to 230 mg protein/g of biomass solids (cellulose and lignin), which is within the range typically used in enzyme binding experiments (0–2000 mg/g) [7, 42, 77].

The dimensions of the simulation box are 95 nm $\times$ 62.5 nm $\times$ 62.5 nm. The overall size of the system is determined by several requirements. The first is to match physical characteristics of the system, i.e., that pretreated cellulose fibers have lengths ${\gtrsim }100$ nm [59], the lignin-to-cellulose ratio and the typical enzyme loading. The second is to obtain statistically meaningful enzyme binding propensities, which require ${\sim }50$*tr*Cel7A molecules to be simulated. Finally, the system consists of highly heterogeneous mesoscale interactions determined by the variety of lignin polymers and association modes.

### Molecular dynamics simulations

The simulations were performed with GROMACS 4.6 [78] using the TIP3P water model [79] and the CHARMM36 carbohydrate [80–82], protein [83, 84, 85], and lignin [86] force fields. Fast hydrogen angle vibrations and rotations were removed employing the virtual sites method [87], thus allowing a 4 fs integration time step. The non-bonded electrostatic interactions were calculated using the reaction field zero (RFZ) method [88] with a 12 Å force and 15.68 Å neighbor-list cutoff. It has been shown that RFZ is of accuracy similar to the commonly used Particle Mesh Ewald method for biomass systems while allowing significantly better parallel computational efficiency above 10,000 cores [89]. A shifting function was applied to the entire Van der Waals potential so that the interaction is zero at the cutoff distance of 12 Å. Neighbor searching was performed every 16 time steps. Bonds were constrained using the LINCS algorithm [90] and the water internal dynamics was constrained using the SETTLE routine [91].The system was simulated in the NPT ensemble.

The equilibration was performed in three steps, during which the temperature was controlled with the Nose–Hoover [92] algorithm (time constant $\tau =$ 1 ps) and, apart from the second step, pressure was controlled with the Berendsen algorithm [93] ($\tau =$ 1 ps). First, 3000 steps were performed, with pressure coupling, employing an integration time step of 1 fs, no virtual sites and constraining only bonds containing hydrogen atoms. Subsequently, 50,000 steps without pressure coupling were performed, with a time step of 2 fs, no virtual sites and position restraints applied on all solute atoms. Finally, 25,000 steps with pressure coupling were performed, with a 4 fs time step, virtual sites on and bonds containing all atoms constrained.

For production, the temperature and pressure were controlled using the velocity rescale thermostat [94] ($\tau =$1 ps) and the Parrinello–Rahman barostat [95] ($\tau =$ 4 ps). Virtual sites and a 4 fs time step were used and all bond lengths were constrained. The total simulation time was 1312 ns. The simulations were carried out on the TITAN XC6 Supercomputer at Oak Ridge National Laboratory, using 60,000 cores at a peak performance of 45 ns/day.

### Analysis methodology

The analysis of multi-million atom, µs-long MD simulations introduces unique challenges, chief among them being the computational time required to obtain quantities of interest over the entire trajectory using serial approaches. To address this in part, our analysis was was carried out with purpose-build python-based VMD scripts [96] on only the heavy atoms of the solutes (cellulose, lignin, and enzyme), thus reducing the number of atoms to be analyzed by a factor of 20. This reduces the memory requirement of the analysis scripts as well as the time to solution, as the time to execute many basic operations (such as selecting subsets of atoms or loading trajectory files) scales linearly with the number of atoms.

The critical concept underlying most of the analysis is that of contact. Traditionally, a “contact” would use a fixed cutoff distance, and if two atoms were within this cutoff, they would be considered in contact. However, the choice of the cutoff value will impact tremendously the number of contacts found. Short cutoffs favor strong interactions such as hydrogen bonds, while longer cutoffs will begin to capture non-specific hydrophobic interactions. We strike a balance between these two extremes by adopting a weighted contact definition similar to the native contact definition introduced by Sheinerman and Brooks [97]. Specifically, the number of contacts between heavy atom *i* in interaction group A and all the heavy atoms in interaction group B is defined as

1 $$\begin{aligned} C_{i}={\displaystyle \sum _{j\in B} {\frac{1}{1+\exp \left( 5\,{\AA }^{-1}\left( d_{ij}-4\,{\AA }\right) \right) } }} \end{aligned}.$$

Here, groups A and B are subsets of the system (cellulose, lignin, or enzyme), and $d_{ij}$ is the distance between atoms *i* and *j*. If groups A and group B are identical (for instance, in the calculation of lignin–lignin contacts), we only count the contacts between unique molecules, neglecting internal molecular contacts. This approach will count both weaker hydrophobic and stronger electrostatic interactions, and will give more weight to the stronger short-range interactions.

Contacts are made and broken repeatedly over the course of the simulation. Indeed, 83–93 % of interactions formed break within 100 ns in our analysis. However, due to some particularly long-lived interactions, on the $\upmu$s timescale, the mean duration of binding events to cellulases is on the order of tens of nanoseconds (Additional file 2: Figure S3). This may not be representative of the overall binding time in vivo due to limitations in timescale for typical MD simulations. While classical MD now routinely brings to life multi-million atom structures [98], atomistic MD of large complexes remains limited to ns-$\upmu$s timescales due to the fs-scale timesteps required for accurate integration in time. Therefore, slow (relaxation time > $\upmu$s) enzyme-biomass dissociation processes and similarly long binding events are not captured here. Explicit rare-event methods or biased sampling may be useful for characterizing such kinetics.

Further analysis was performed to determine the orientation of the bound Cel7A relative to the long axis of the cellulose and the rotational and translational diffusion constants. These analyses were implemented as python-based VMD [96] scripts, stored using numpy [99], and plotted using matplotlib [100]. In addition, the formation and time evolution of the interaction networks present in the simulation were carried out using the NetworkX library [101] and the Gephi program [45].

#### Surface area computation

**Fig. 10:**
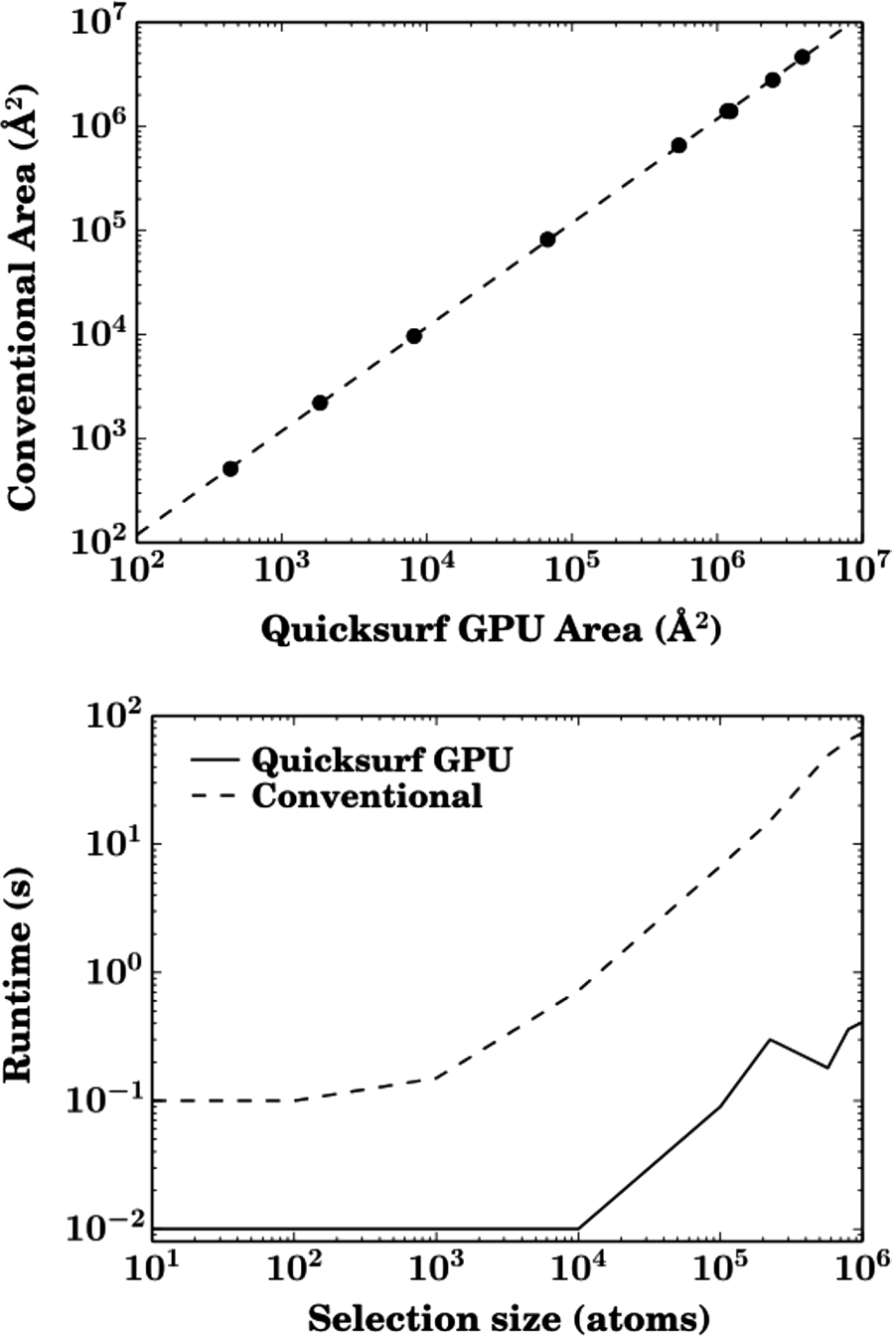
Accuracy (*top*) and runtime (*bottom*) of a conventional approach vs. our GPU-accelerated surface area calculation for test atom selections of a given size. The r-value for the linear fit between the conventional surface area and the GPU-calculated surface area is 0.99997 with a slope of 0.9997; however the intercept in the plot is not zero, indicating a consistent percentage offset of ~20 %. The runtimes represent the time required to calculate the surface area of a single atom selection once

Computing surface area for large systems using conventional algorithms, where many random points on a sphere around every atom in the selection are checked for proximity to nearby atoms, was determined to be too inefficient for our purposes, as a single calculation on the complete trajectory was estimated to take a month in a serial process. Instead, we developed a new tool to efficiently calculate interfacial surface area by utilizing methodologies from the computer graphics literature which had already been incorporated into VMD [96]. In brief, we calculate the surface area using the grid-based QuickSurf [102] representation, and combined the surfaces from different groups of atoms to obtain the interfacial surface area between two groups. This approach is ${\sim }100$ times faster than the conventional solvent accessible surface area (SASA) calculation implemented in VMD. A conventional SASA calculation on 100,000 atoms evaluates 500 points per atom and determines if they are within a cutoff distance (3–5 Å) of other nearby atoms (20–30 atoms) in that selection, which overall requires over 1 billion distance comparisons. In contrast, the QuickSurf surface calculation performed on the same 100,000 atoms evaluates the value of a Gaussian on a grid with a resolution on the order of 1 Å. The Gaussian function is assumed to be negligible 5–7 Å away from its center (depending on the resolution requested), and therefore in total we only evaluate the Gaussian ${\sim }100$ million times for each atom selection for which the area is computed. Additional computation is required to generate a surface using the marching cubes algorithm [103] and to calculate the surface area from the resulting triangles. All of the aforementioned steps were carried out on a GPU and the net result is a calculation that is 100–300 fold faster (Fig. 10), depending on the size of the selection, compared to a conventional SASA calculation performed on one CPU.

To compute the surface, we added 3 Å to the radius of every heavy atom, so as to represent the radii of both the heavy atom and the missing hydrogens, then scaled them by 0.47 when calculating the Gaussian, and use 0.4 as the Gaussian density threshold for computing the surface. These parameters were determined by converting the optimal parameters found by Grant and Pickup [104], with a 1.5 Å grid spacing found through experimentation. Example surfaces and how they compare are shown in Additional file 2: Figure S9.

One particular caution to using the above approach is that the surfaces tend to be 10–20 % smaller than those computed by SASA, due to the smoother Gaussian surfaces that paper over the nooks and crannies between atoms (Additional file 2: Figure S10). However, while the absolute values may be different, the trends and the relative surface areas are consistent between the two methods. In our particular application, where we are interested in the interface area relative to the total surface area, the difference between this method and conventional SASA is expected to be minimal.

## Additional files

10.1186/s13068-015-0379-8 This is an animation of the full trajectory, using the same representation as in Fig. 1.
10.1186/s13068-015-0379-8 Supplementary document providing supplementary figures and tables.
10.1186/s13068-015-0379-8 This is an animation of how the contacts change with time, using the same representation as in Fig. 3.
10.1186/s13068-015-0379-8 3-D representation of the contacts points between two TrCel7A enzymes. Heavy atoms that are found to makecontacts are colored based on the contact number, with warmer colors having more contacts relative to coolercolors. For context, the remaining protein structure is also shown, along with the heavy atoms for the remainingresidues. This animation is related to Fig. 4c.
10.1186/s13068-015-0379-8 3-D representation of contacts points between TrCel7A and lignin. Heavy atoms that are found to make contactsare colored based on the contact number, with warmer colors having more contacts relative to cooler colors. Forcontext, the remaining protein structure is also shown, along with the heavy atoms for the remaining residues. Thisanimation is related to Fig. 4b.
10.1186/s13068-015-0379-8 3-D representation of contacts points between TrCel7A copies and cellulose. Heavy atoms that are found to makecontacts are colored based on the contact number, with warmer colors having more contacts relative to coolercolors. For context, the remaining protein structure is also shown, along with the heavy atoms for the remainingresidues. This animation is related to Fig. 4a.
10.1186/s13068-015-0379-8 PDB file of a single copy of the enzyme taken from the trajectory where the average enzyme-enzyme contactnumber is provided in the beta column.
10.1186/s13068-015-0379-8 PDB file of a single copy of the enzyme taken from the trajectory where the average lignin-enzyme contact numberis provided in the beta column.
10.1186/s13068-015-0379-8 PDB file of a single copy of the enzyme taken from the trajectory where the average cellulose-enzyme contactnumber is provided in the beta column.
10.1186/s13068-015-0379-8 A file containing the initial coordinates for the simulation system for independent analysis.
10.1186/s13068-015-0379-8 A zip archive containing a gallery of each of the cellulases that bound to cellulose in the context of their environment. Each image within the gallery is one snapshot taken from the end of the trajectory showing the relative position of each enzyme (green) that makes contact with the cellulose (red). Nearby lignins are shown in blue, and the substrate tunnel is a yellow surface to orient the viewer. The three tyrosine residues are shown in orange. Note that for each protein, there are 4 images, taken from different relative orientations to the cellulose fibril (0, 90, 180, and 270), and are labeled accordingly in their filenames.

## Abbreviations

CBM: Cellulose-binding module
TrCel7A: *Trichoderma reesei* fungal cellulase Cel7A
MD: Molecular dynamics
CH: Crystalline cellulose, high lignin coverage
CL: Crystalline cellulose, low lignin coverage
NonC: Non-crystalline cellulose, low lignin coverage
CD: Catalytic domain
