# Supplementary material for: Mechanism of lignin inhibition of enzymatic biomass deconstruction
Source: Biotechnol Biofuels. 2015 Dec 21;8:217. doi: 10.1186/s13068-015-0379-8 (PMC4687093; doi:10.1186/s13068-015-0379-8)
Supplement: Supplementary file 2 — 10.1186/s13068-015-0379-8 Supplementary document providing supplementary figures and tables. [file 13068_2015_379_MOESM2_ESM.pdf]

Supporting information for: Interference Trinity: Lignins Role in Biomass Recalcitrance

Josh V. Vermaas,[†,‡] Xianghong Qi,[¶,‡] Roland Schulz,[¶,‡] Loukas Petridis,[‡] and Jeremy C. Smith[*,¶,‡]

†*Center for Biophysics and Computational Biology, University of Illinois at Urbana-Champaign, Urbana, Illinois 61801*

‡*UT/ORNL Center for Molecular Biophysics, Oak Ridge National Laboratory, Oak Ridge, Tennessee 37830*

¶*Department of Biochemistry and Cellular and Molecular Biology, University of Tennessee, Knoxville, TN 37996*

E-mail: smithjc@ornl.gov

Supporting Tables and Figures

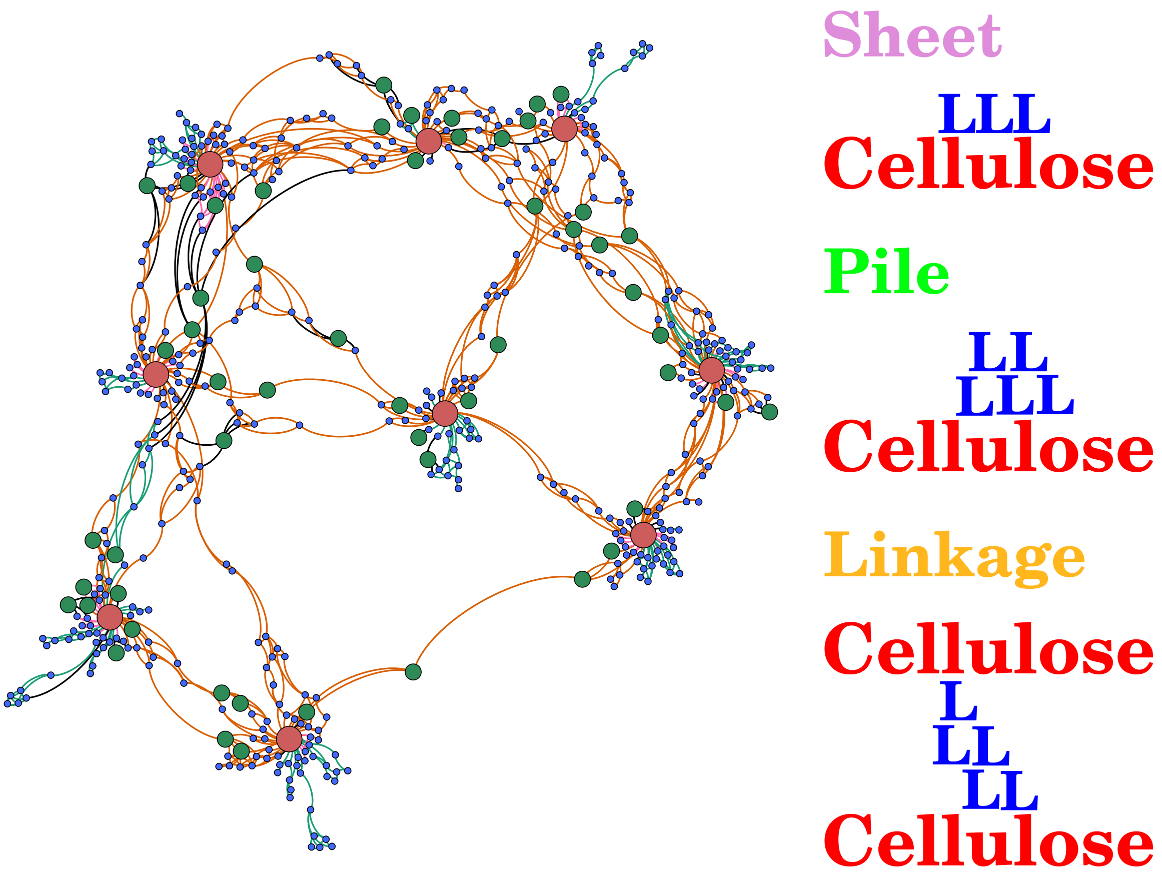

Figure S1: Alternative representation of Fig. 3, where individual links between elements are colored according to the type of lignin cluster they belong to. On the right hand side is a schematic showing the general topology of each type of lignin cluster, where "L" stands in for a lignin molecule. "Sheets" are monolayers of lignin on a single cellulose fibril, and are colored in pink. "Piles" are lignin aggregates onto a single cellulose fibril, and are colored in green. "Linkages" are lignin and protein aggregates that link together two cellulose fibrils. We have colored these linkages orange.

Table S1: Statistics of each lignin cluster type, summarizing the number of clusters, number of lignins, number of enzymes, average cluster size, and the ratio of lignins to enzymes in each cluster type. The clusters are visualized in Fig. S1.

Cluster mode	Pile	Sheet	Linkage
Number of clusters	22	27	17
Total number of lignin molecules	136	63	266
Total number of bound enzymes	6	9	44
Average size	6.45	2.67	18.24
Lignin / Enzyme Ratio	22.7	7.0	6.0

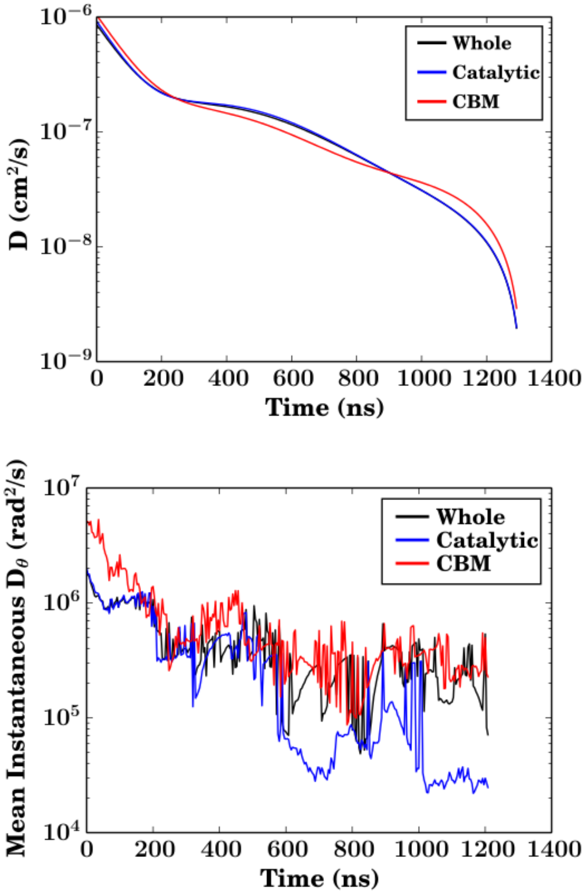

Figure S2: Time-dependent, instantaneous translational (D, top) and rotational (D_θ, bottom) diffusion constant of *tr*Cel7A. Angular diffusion constant (D_θ) is calculated for the whole protein (black), catalytic domain (blue), and CBM (red). We find the CBM, possibly due to its smaller size, to reorient more rapidly than the CD. D is obtained by approximating the MSD by the differentiable function: $6Dt = MSD(t) \approx A\exp\left(k\left(t-1312\right)\right) + \sum_{i=1}^{5} b_i \left(t-1312\right)^{2i} + c$ (dashed lines on the left). The angular diffusion constant (D_θ) over time for the whole protein (black), catalytic domain (blue), and CBM (red), was estimated from the angular autocorrelation function, the standard method for determining angular diffusion from MD trajectories.[S1] D and D_θ are therefore calculated using two different approaches.

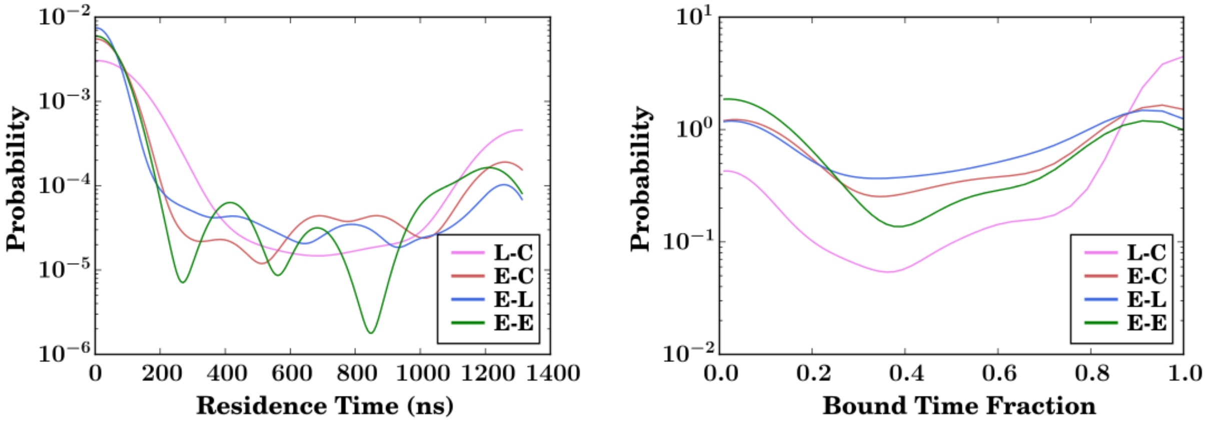

Figure S3: Residence time (left) and bound time fraction (right) distributions for four different interaction types: lignin-cellulose (L-C, pink), enzyme-cellulose (E-C, red), enzyme-lignin (E-L, blue) and enzyme-enzyme (E-E, green).The residence times, a measure of binding kinetics, are calculated by declaring a contact formed when the closest contact distance between molecules is less than 3 Å, and broken when the distance exceeds 5 Å for longer than 1 ns. The mean residence times are: 186 ns for L-C contacts, 72 ns for E-C contacts, 46 ns for E-L contacts, and 64 ns for E-E contacts. The bound time fraction is the ratio of the total time a specific contact is formed (allowing contacts to form and break) divided by the total simulation time. All plotted distributions have been smoothed by a Gaussian kernel density estimator.

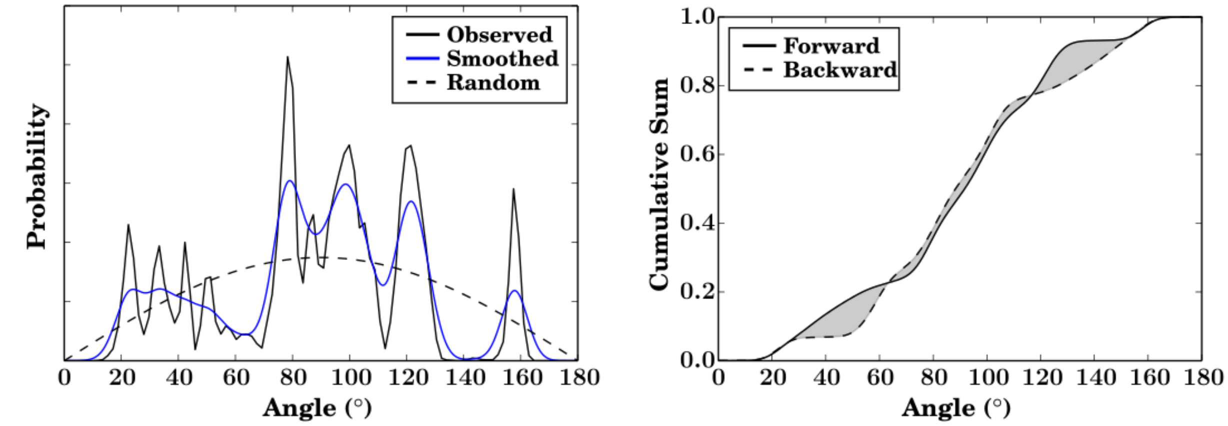

Figure S4: Probability distribution and its cumulative sum of the angle θ between the substrate tunnel of a CD with respect to the long axis of the cellulose to which it is bound (averaged over the 30 bound enzymes and over the last 300 ns). *L*eft: The solid black line is the exact calculated probability distribution, the blue line is a Gaussian-smoothed version, and the dotted black line is the distribution expected if an orientation were completely random. *R*ight: The cumulative sum of the distribution measured going either forwards, from 0° to 180° (solid line) or backwards, from 180° to 0° (dotted line). The difference between the two lines (gray area), highlights the preferential orientation of the CD such that the substrate tunnel is parallel rather than anti-parallel to the cellulose fibril beneath it.

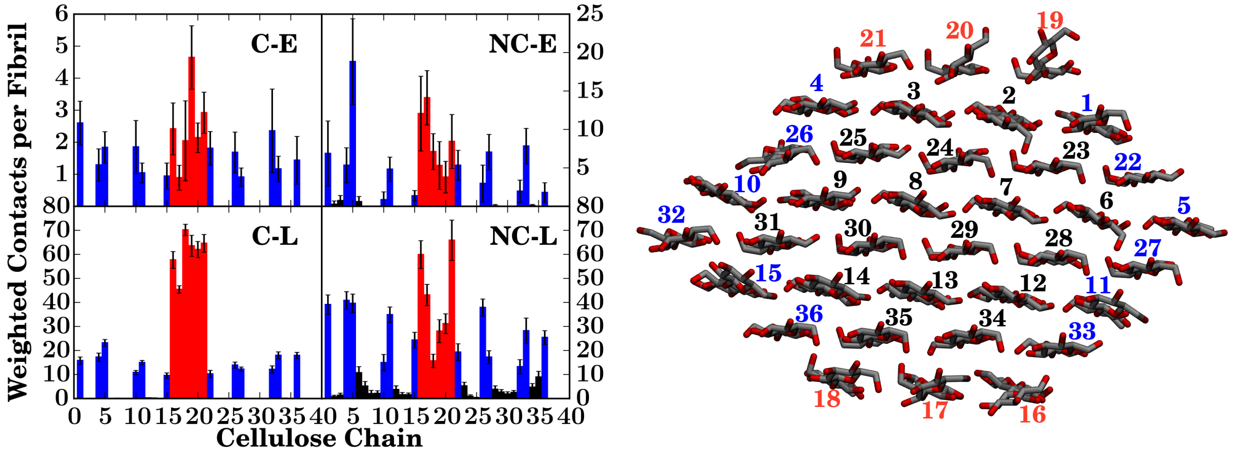

Figure S5: Left panel: chain by chain breakdown of the contacts per fibril, involving crystalline cellulose (C), non-crystalline cellulose (NC), lignin (L) and enzymes (E). Hydrophobic cellulose chains are labeled in red, hydrophilic chains in blue, and internal chains in black. Right panel: a legend of the labeling scheme of each chain. Hydrophobic chains are labeled in red, hydrophilic chains in blue, and internal chains in black.

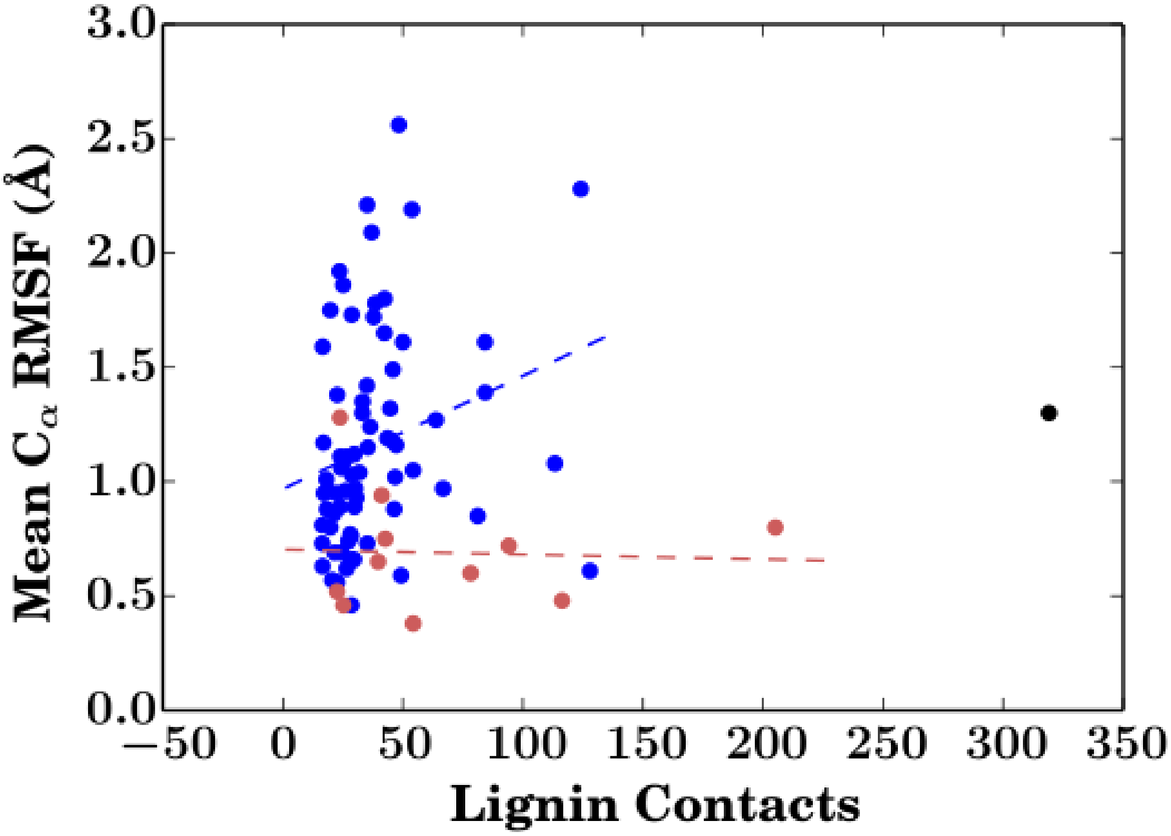

Figure S6: Average C_α room mean square fluctuation (RMSF) of each enzyme residue as a function of the average number of residue-lignin contacts. Blue points representing residues of the CD, and red points of the CBM. The dotted lines represent the best linear fit to the scattered points, showing a lack of clear correlation between contacts and high RMSF, the latter being an indication of denaturation.

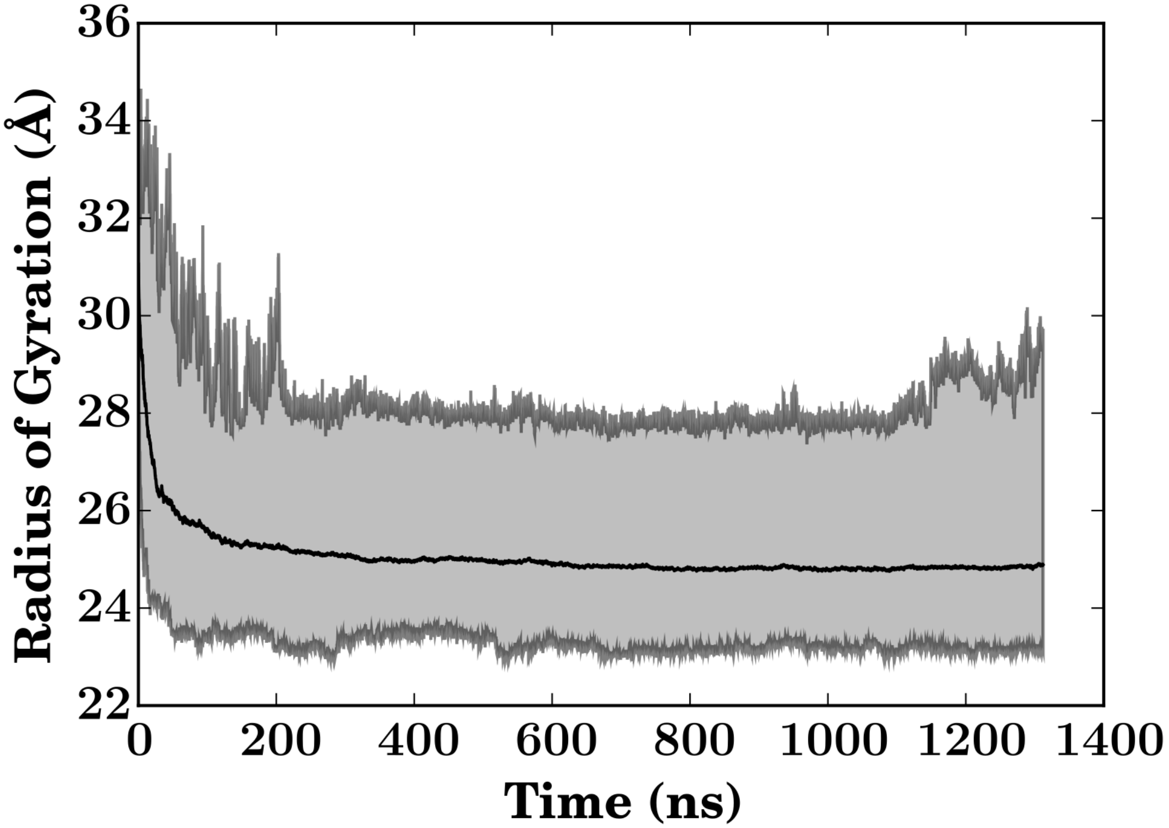

Figure S7: Mean enzyme radius of gyration over time. The mean value is drawn as a black line, and the full range of the radius of gyration is shaded.

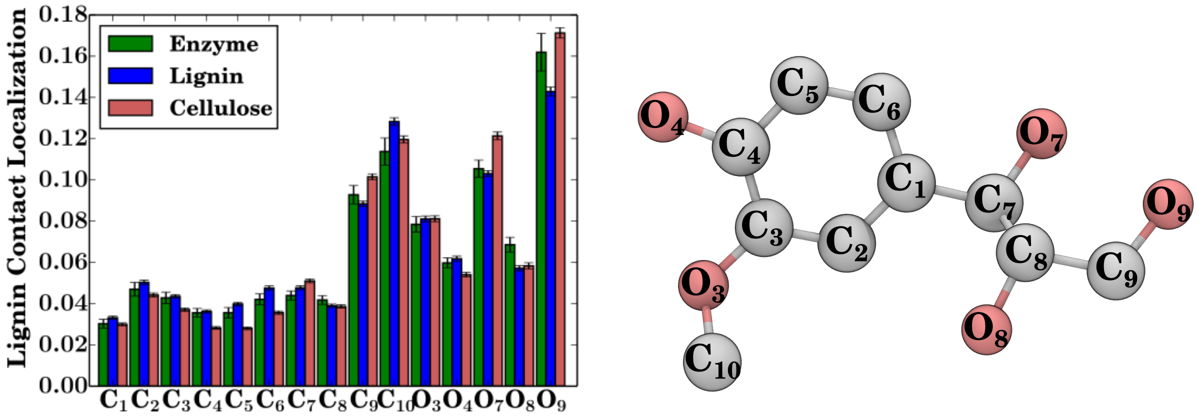

Figure S8: (Left) Average normalized number of contacts between lignin heavy atoms and the three interaction partners: enzyme, other lignins and cellulose. (Right) Simple representation of the lignin monomer used in lignin construction, with each of the heavy atoms labeled by name.

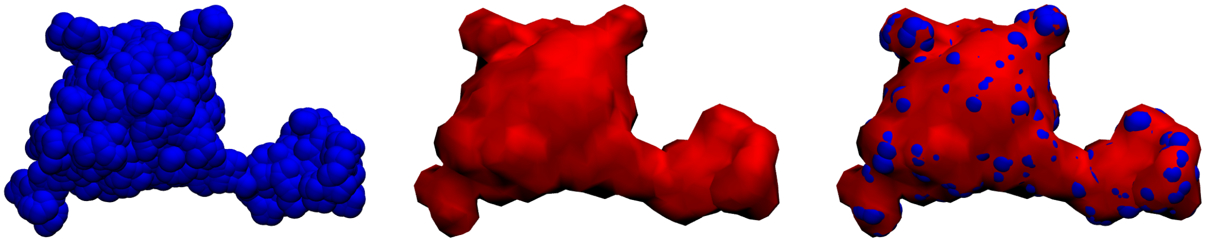

Figure S9: Conventional SASA (blue, left) and QuickSurf (red, center) surfaces around a single copy of Cel7A, in addition to their overlay (right). The surfaces used are 3 Å surface beyond the heavy atom radii. The QuickSurf resolution shown is 1.5 Å, as was used in the surface area calculation. Finer resolutions yield more aesthetically pleasing figures, but also increase runtime.

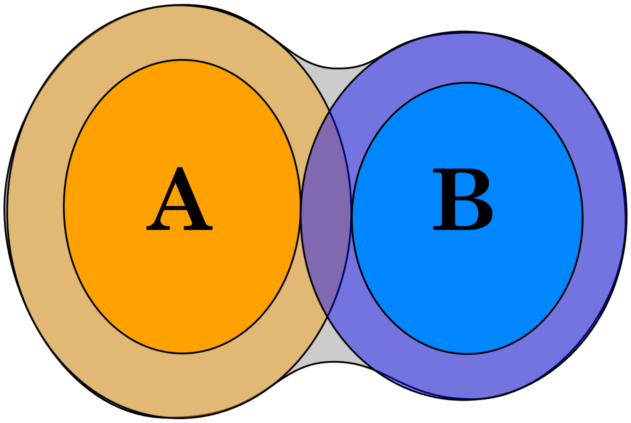

Figure S10: Schematic demonstrating the interface calculation from surface area alone. Given S_A, the surface area of group A (orange surface), S_B, the surface area of group B (blue surface), and S_{AB} the gaussian surface area of the combined selection (gray surface), we approximate the interface area (IA) between A and B as $IA = \frac{1}{2}(S_A + S_B - S_{AB})$. Note that this approximation slightly underestimates the contact surface due to the smooth gaussian surface between the two groups. However since this approximation is used consistently, the trends remain unchanged.

References

(S1) Smith, P. E.; van Gunsteren, W. F. *Journal of Molecular Biology* **1994**, *236*, 629–36.
