# Supplementary figures and images for: Mechanism of lignin inhibition of enzymatic biomass deconstruction (part 1 of 2)
Source: Biotechnol Biofuels. 2015 Dec 21;8:217. doi: 10.1186/s13068-015-0379-8 (PMC4687093; doi:10.1186/s13068-015-0379-8)

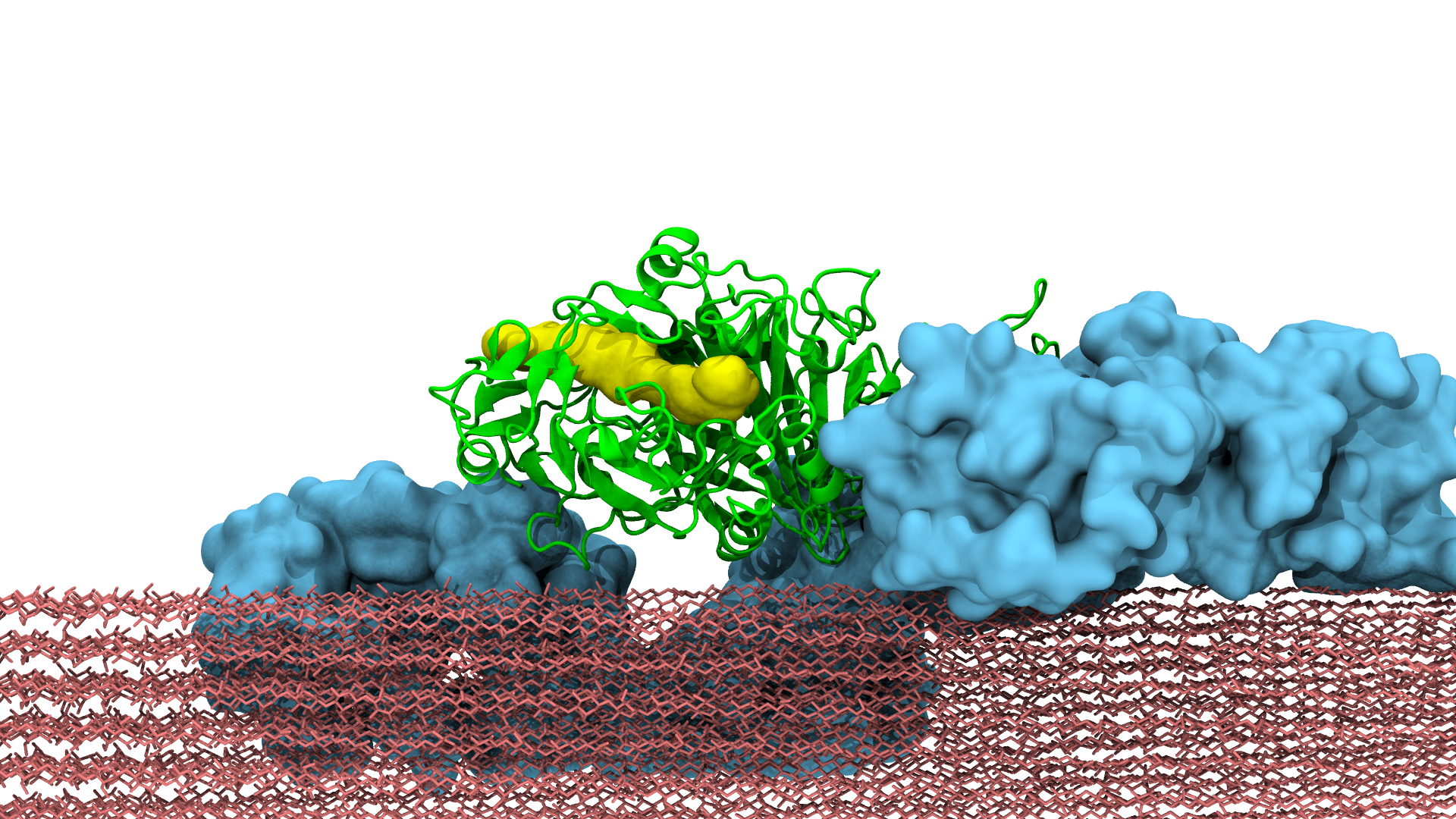

Supplement: Supplementary file 11 — 10.1186/s13068-015-0379-8 A zip archive containing a gallery of each of the cellulases that bound to cellulose in the context of their environment. Each image within the gallery is one snapshot taken from the end of the trajectory showing the relative position of each enzyme (green) that makes contact with the cellulose (red). Nearby lignins are shown in blue, and the substrate tunnel is a yellow surface to orient the viewer. The three tyrosine residues are shown in orange. Note that for each protein, there are 4 images, taken from different relative orientations to the cellulose fibril (0, 90, 180, and 270), and are labeled accordingly in their filenames. [file 13068_2015_379_MOESM11_ESM.zip › gallery/C-0_P-06_0.png]

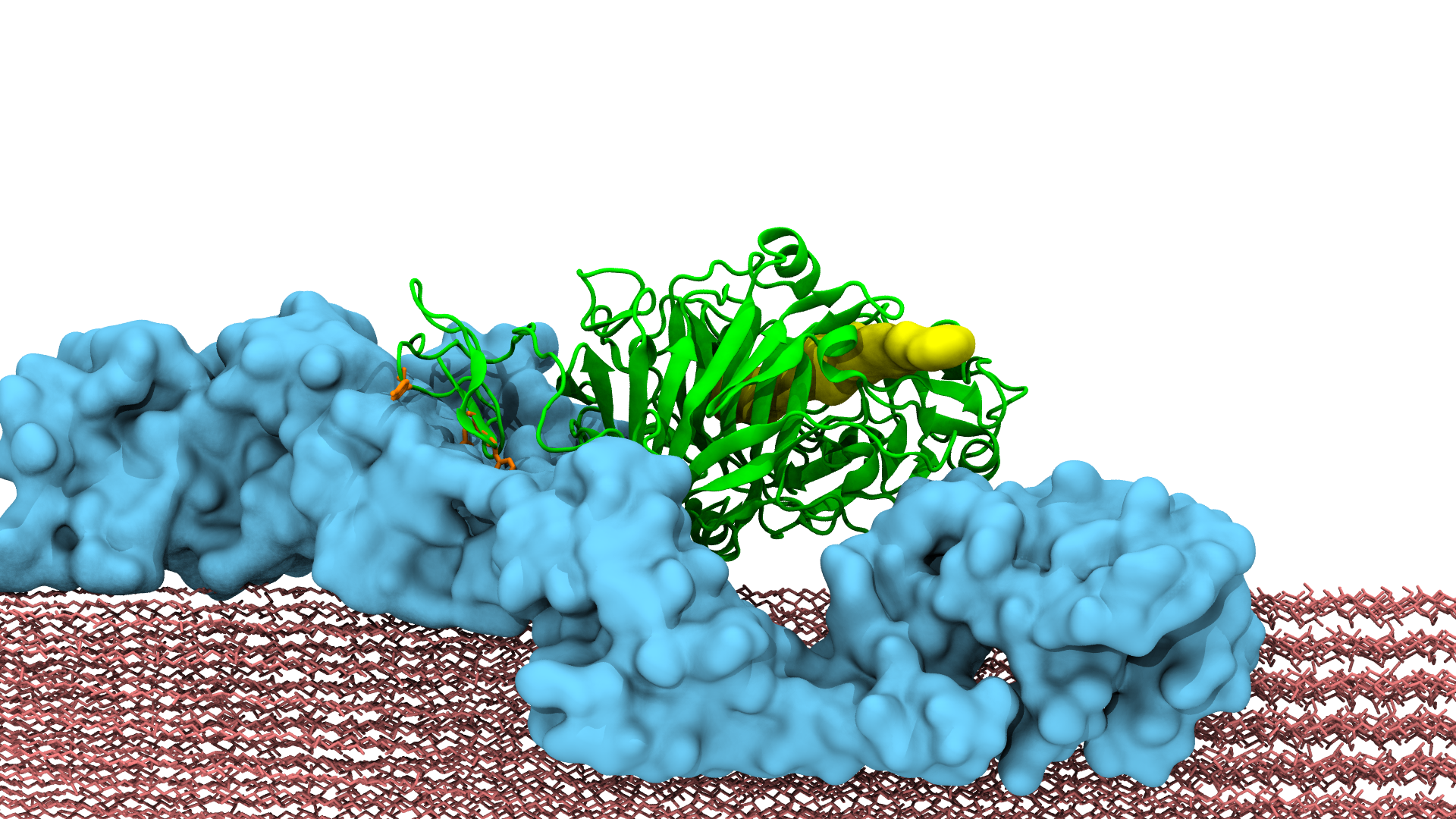

Supplement: Supplementary file 11 — 10.1186/s13068-015-0379-8 A zip archive containing a gallery of each of the cellulases that bound to cellulose in the context of their environment. Each image within the gallery is one snapshot taken from the end of the trajectory showing the relative position of each enzyme (green) that makes contact with the cellulose (red). Nearby lignins are shown in blue, and the substrate tunnel is a yellow surface to orient the viewer. The three tyrosine residues are shown in orange. Note that for each protein, there are 4 images, taken from different relative orientations to the cellulose fibril (0, 90, 180, and 270), and are labeled accordingly in their filenames. [file 13068_2015_379_MOESM11_ESM.zip › gallery/C-0_P-06_180.png]

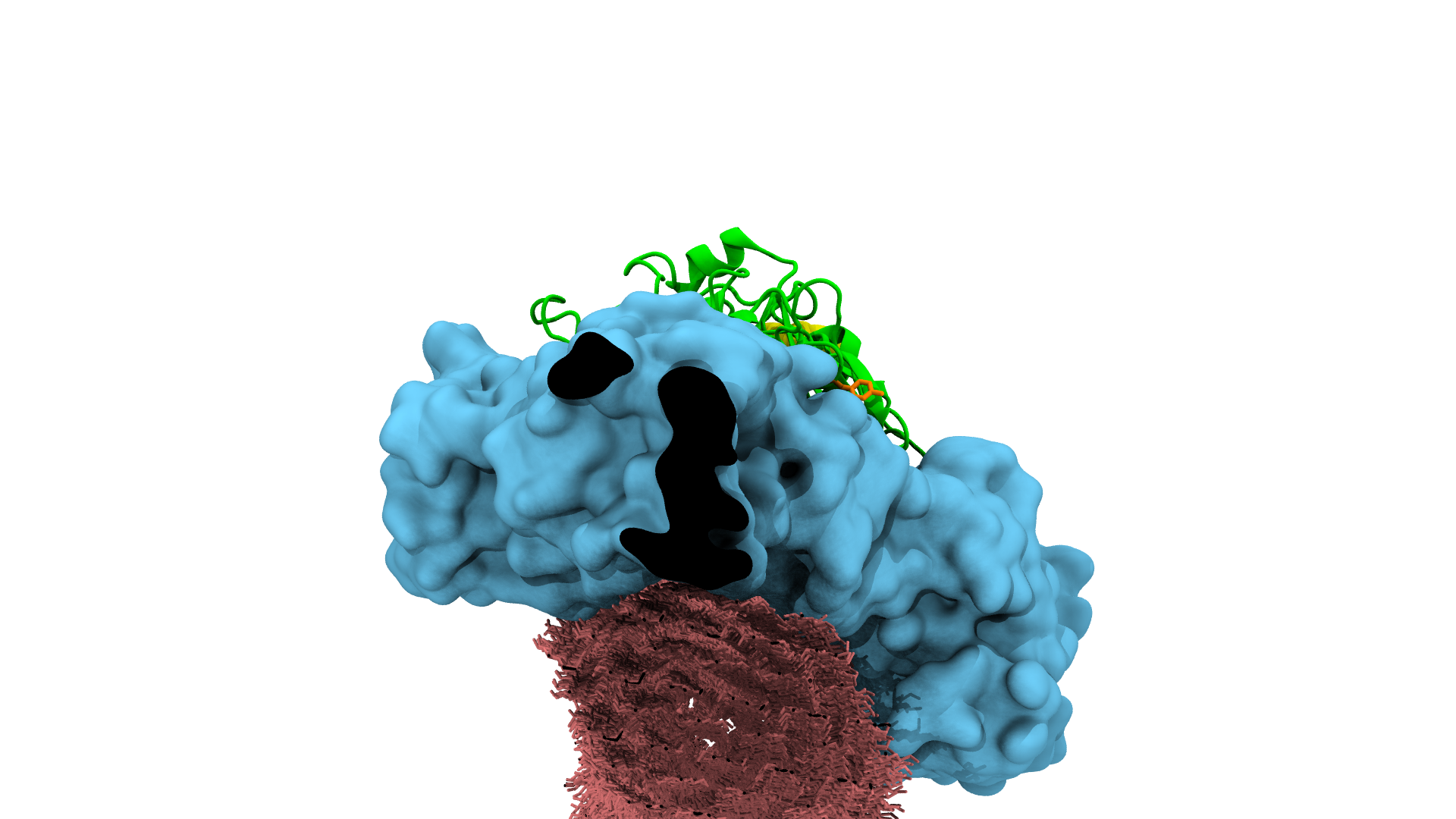

Supplement: Supplementary file 11 — 10.1186/s13068-015-0379-8 A zip archive containing a gallery of each of the cellulases that bound to cellulose in the context of their environment. Each image within the gallery is one snapshot taken from the end of the trajectory showing the relative position of each enzyme (green) that makes contact with the cellulose (red). Nearby lignins are shown in blue, and the substrate tunnel is a yellow surface to orient the viewer. The three tyrosine residues are shown in orange. Note that for each protein, there are 4 images, taken from different relative orientations to the cellulose fibril (0, 90, 180, and 270), and are labeled accordingly in their filenames. [file 13068_2015_379_MOESM11_ESM.zip › gallery/C-0_P-06_270.png]

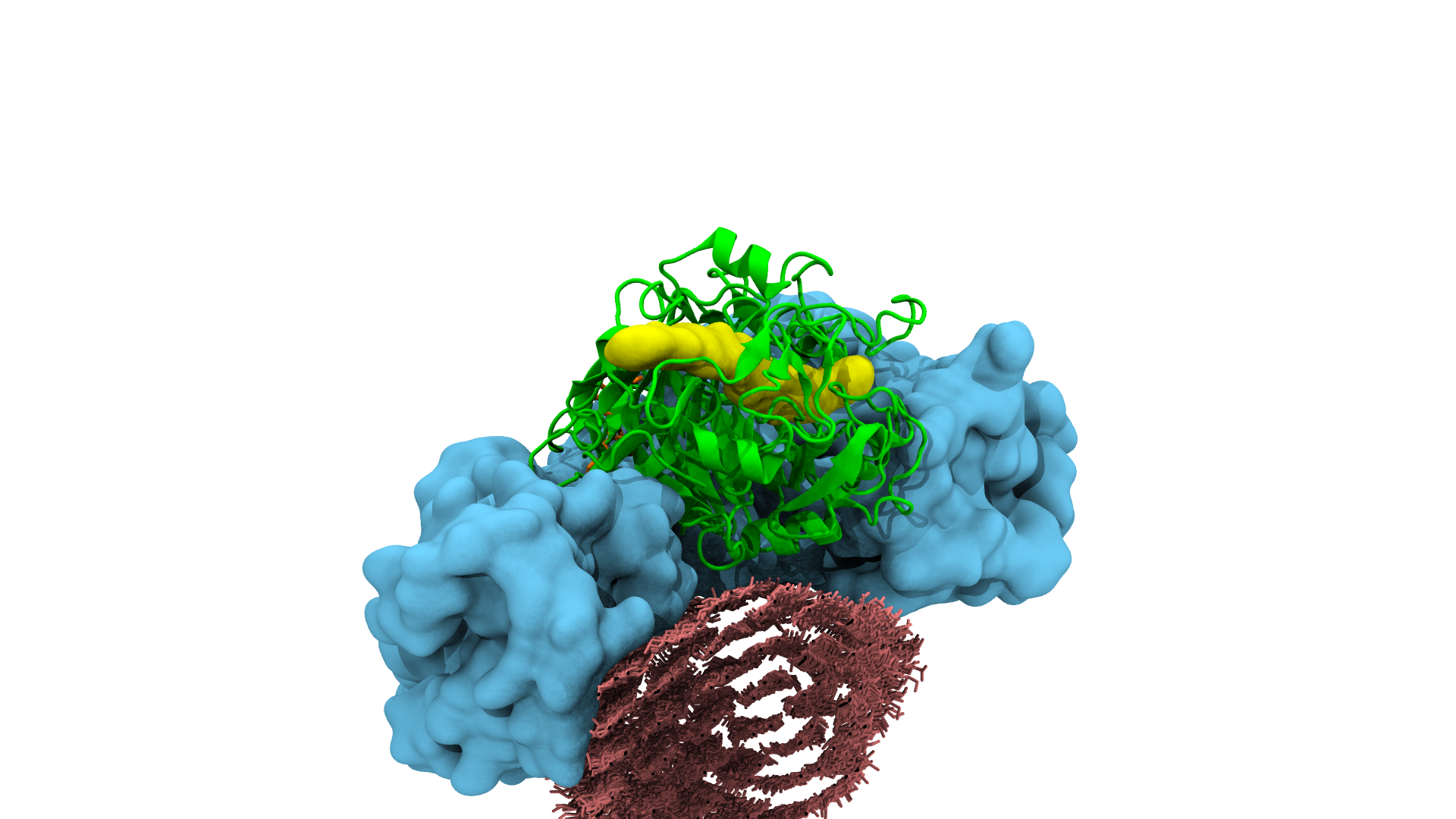

Supplement: Supplementary file 11 — 10.1186/s13068-015-0379-8 A zip archive containing a gallery of each of the cellulases that bound to cellulose in the context of their environment. Each image within the gallery is one snapshot taken from the end of the trajectory showing the relative position of each enzyme (green) that makes contact with the cellulose (red). Nearby lignins are shown in blue, and the substrate tunnel is a yellow surface to orient the viewer. The three tyrosine residues are shown in orange. Note that for each protein, there are 4 images, taken from different relative orientations to the cellulose fibril (0, 90, 180, and 270), and are labeled accordingly in their filenames. [file 13068_2015_379_MOESM11_ESM.zip › gallery/C-0_P-06_90.png]

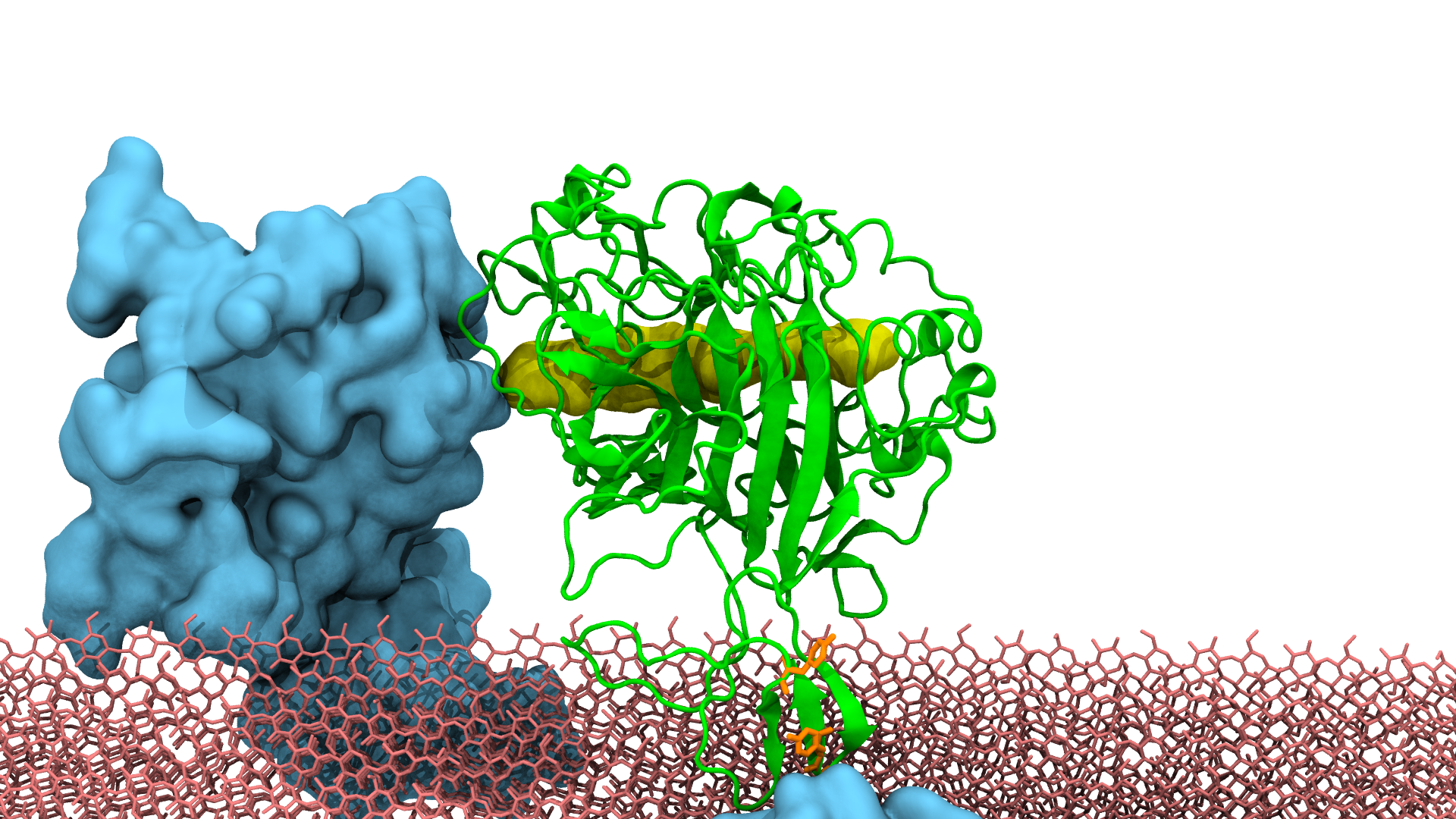

Supplement: Supplementary file 11 — 10.1186/s13068-015-0379-8 A zip archive containing a gallery of each of the cellulases that bound to cellulose in the context of their environment. Each image within the gallery is one snapshot taken from the end of the trajectory showing the relative position of each enzyme (green) that makes contact with the cellulose (red). Nearby lignins are shown in blue, and the substrate tunnel is a yellow surface to orient the viewer. The three tyrosine residues are shown in orange. Note that for each protein, there are 4 images, taken from different relative orientations to the cellulose fibril (0, 90, 180, and 270), and are labeled accordingly in their filenames. [file 13068_2015_379_MOESM11_ESM.zip › gallery/C-0_P-08_0.png]

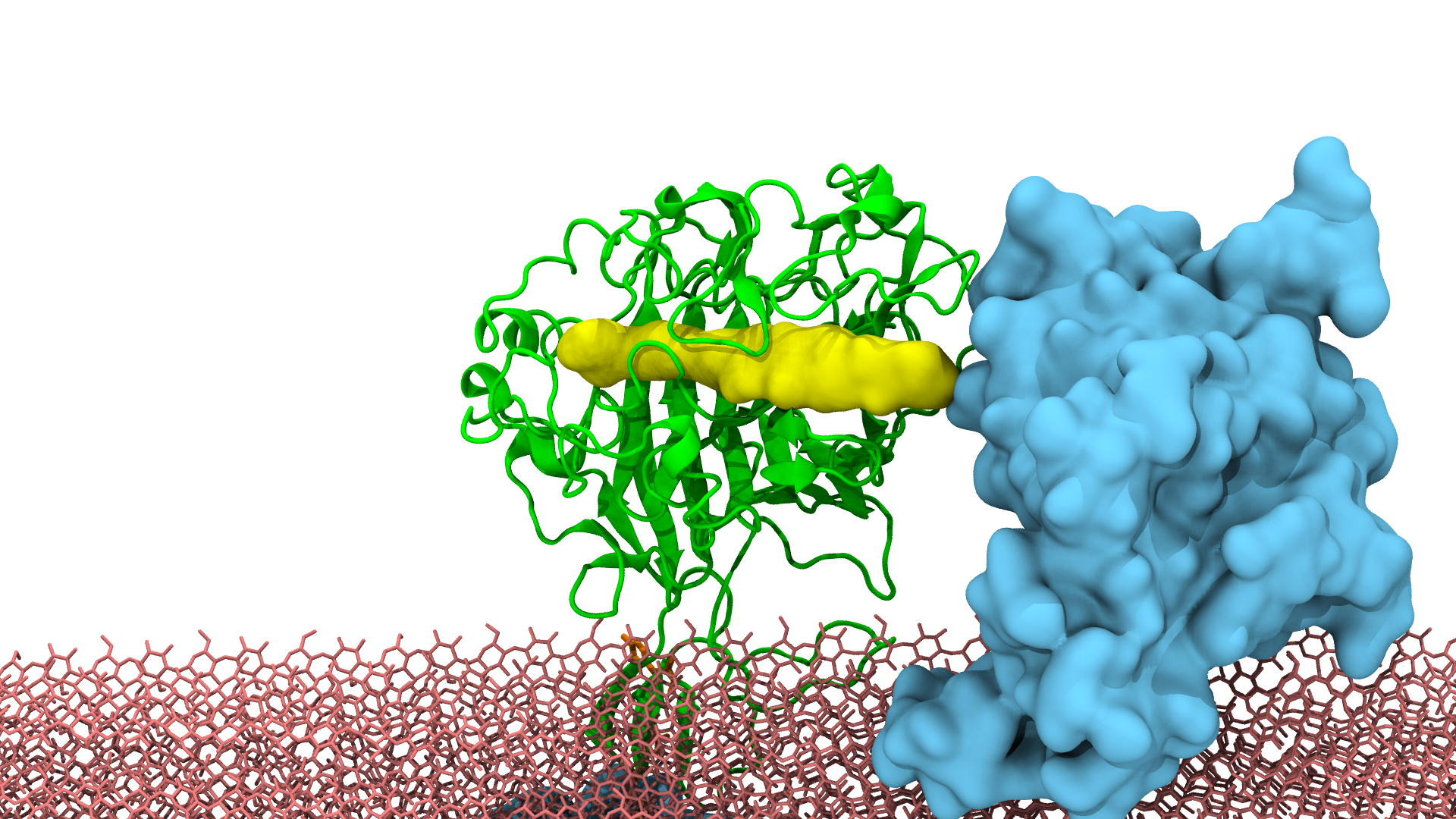

Supplement: Supplementary file 11 — 10.1186/s13068-015-0379-8 A zip archive containing a gallery of each of the cellulases that bound to cellulose in the context of their environment. Each image within the gallery is one snapshot taken from the end of the trajectory showing the relative position of each enzyme (green) that makes contact with the cellulose (red). Nearby lignins are shown in blue, and the substrate tunnel is a yellow surface to orient the viewer. The three tyrosine residues are shown in orange. Note that for each protein, there are 4 images, taken from different relative orientations to the cellulose fibril (0, 90, 180, and 270), and are labeled accordingly in their filenames. [file 13068_2015_379_MOESM11_ESM.zip › gallery/C-0_P-08_180.png]

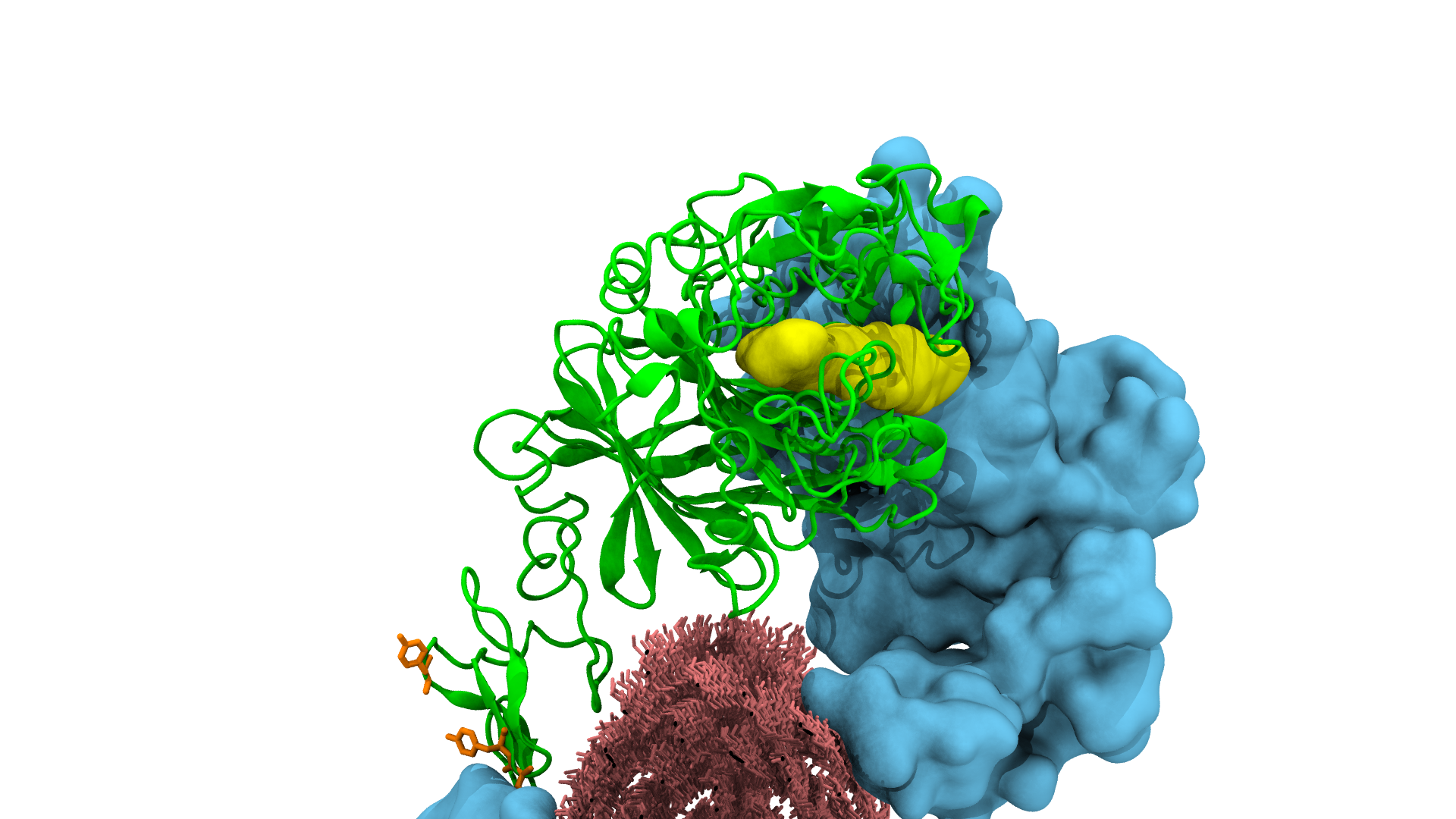

Supplement: Supplementary file 11 — 10.1186/s13068-015-0379-8 A zip archive containing a gallery of each of the cellulases that bound to cellulose in the context of their environment. Each image within the gallery is one snapshot taken from the end of the trajectory showing the relative position of each enzyme (green) that makes contact with the cellulose (red). Nearby lignins are shown in blue, and the substrate tunnel is a yellow surface to orient the viewer. The three tyrosine residues are shown in orange. Note that for each protein, there are 4 images, taken from different relative orientations to the cellulose fibril (0, 90, 180, and 270), and are labeled accordingly in their filenames. [file 13068_2015_379_MOESM11_ESM.zip › gallery/C-0_P-08_270.png]

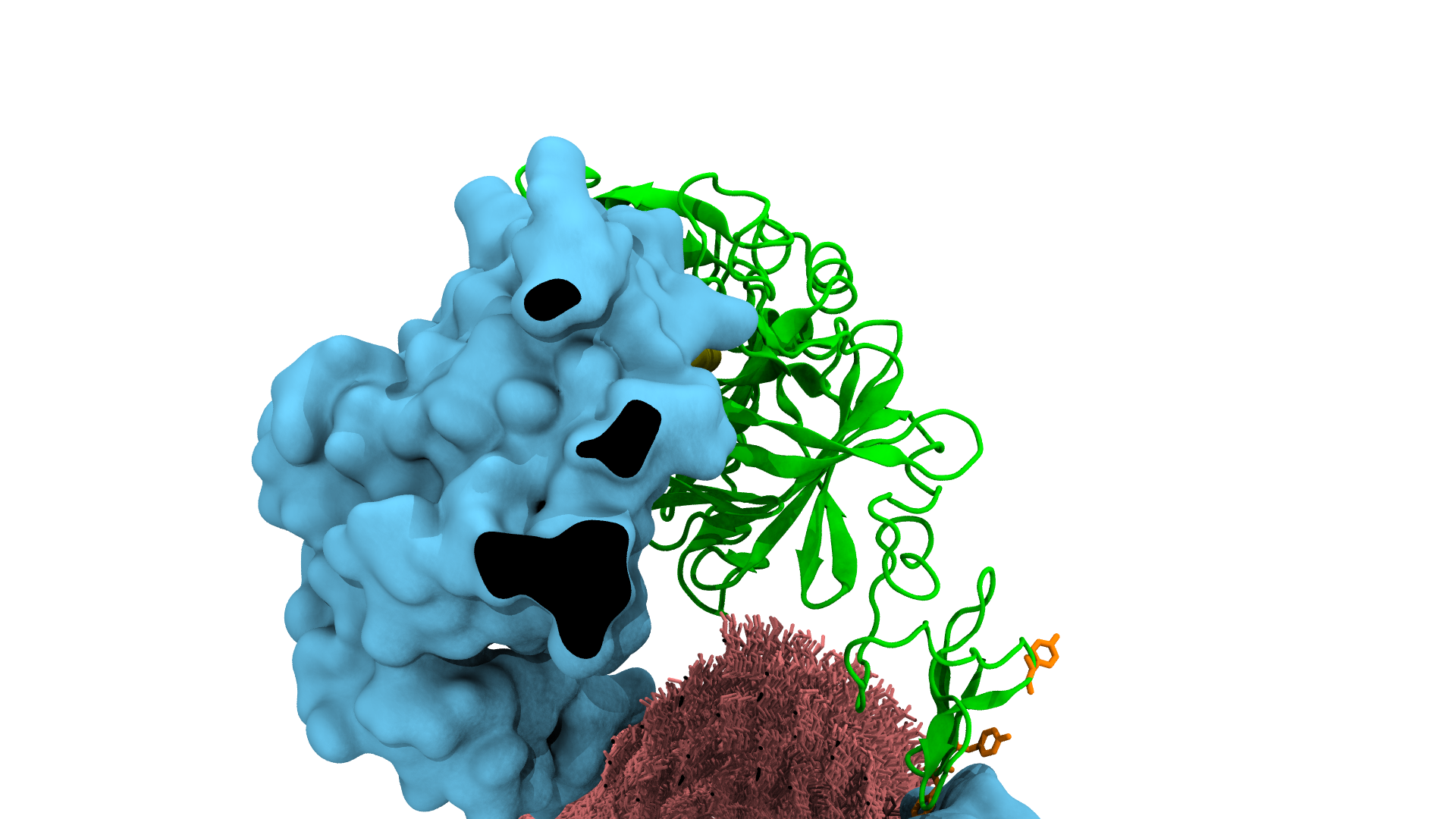

Supplement: Supplementary file 11 — 10.1186/s13068-015-0379-8 A zip archive containing a gallery of each of the cellulases that bound to cellulose in the context of their environment. Each image within the gallery is one snapshot taken from the end of the trajectory showing the relative position of each enzyme (green) that makes contact with the cellulose (red). Nearby lignins are shown in blue, and the substrate tunnel is a yellow surface to orient the viewer. The three tyrosine residues are shown in orange. Note that for each protein, there are 4 images, taken from different relative orientations to the cellulose fibril (0, 90, 180, and 270), and are labeled accordingly in their filenames. [file 13068_2015_379_MOESM11_ESM.zip › gallery/C-0_P-08_90.png]

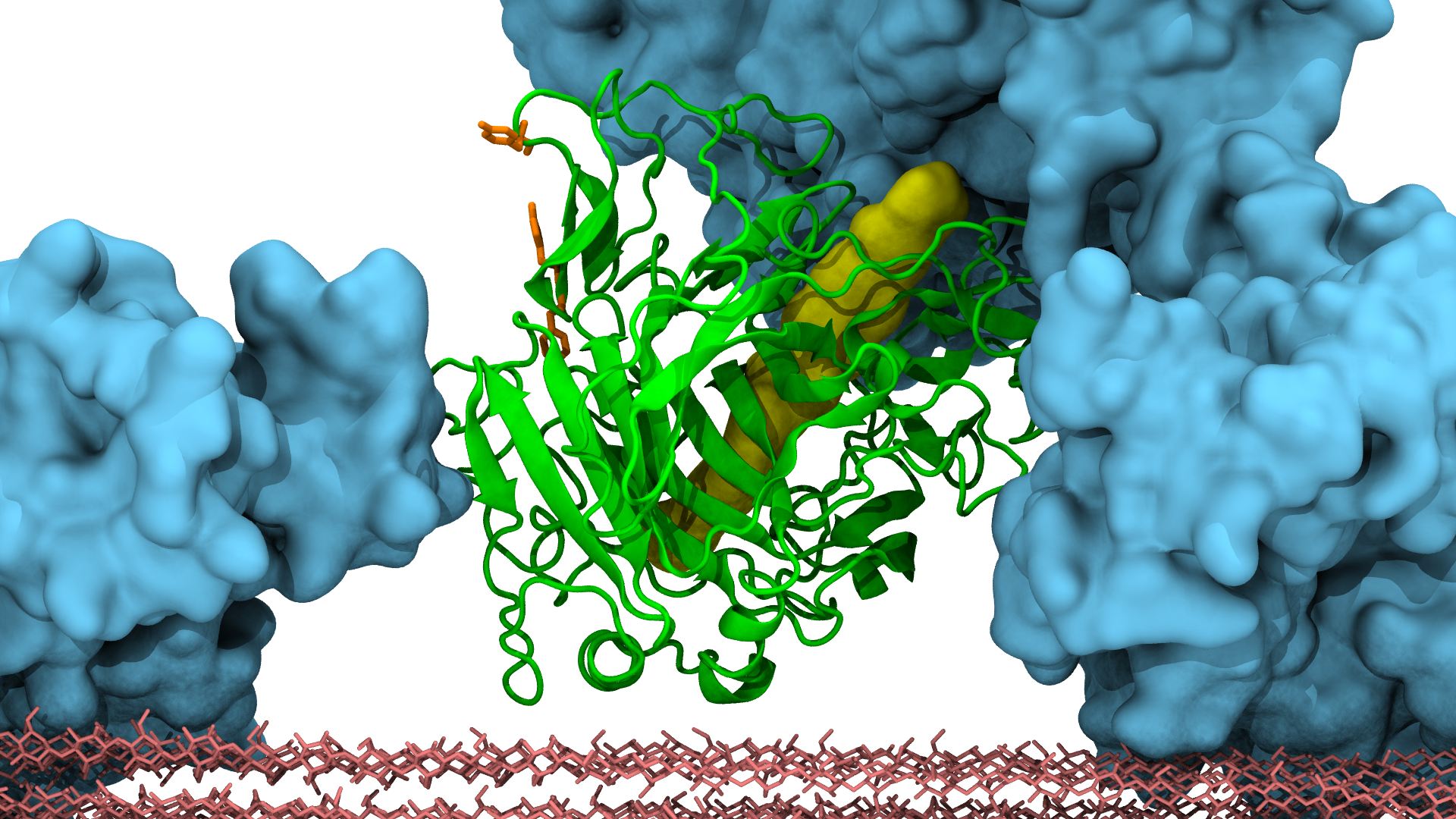

Supplement: Supplementary file 11 — 10.1186/s13068-015-0379-8 A zip archive containing a gallery of each of the cellulases that bound to cellulose in the context of their environment. Each image within the gallery is one snapshot taken from the end of the trajectory showing the relative position of each enzyme (green) that makes contact with the cellulose (red). Nearby lignins are shown in blue, and the substrate tunnel is a yellow surface to orient the viewer. The three tyrosine residues are shown in orange. Note that for each protein, there are 4 images, taken from different relative orientations to the cellulose fibril (0, 90, 180, and 270), and are labeled accordingly in their filenames. [file 13068_2015_379_MOESM11_ESM.zip › gallery/C-0_P-33_0.png]

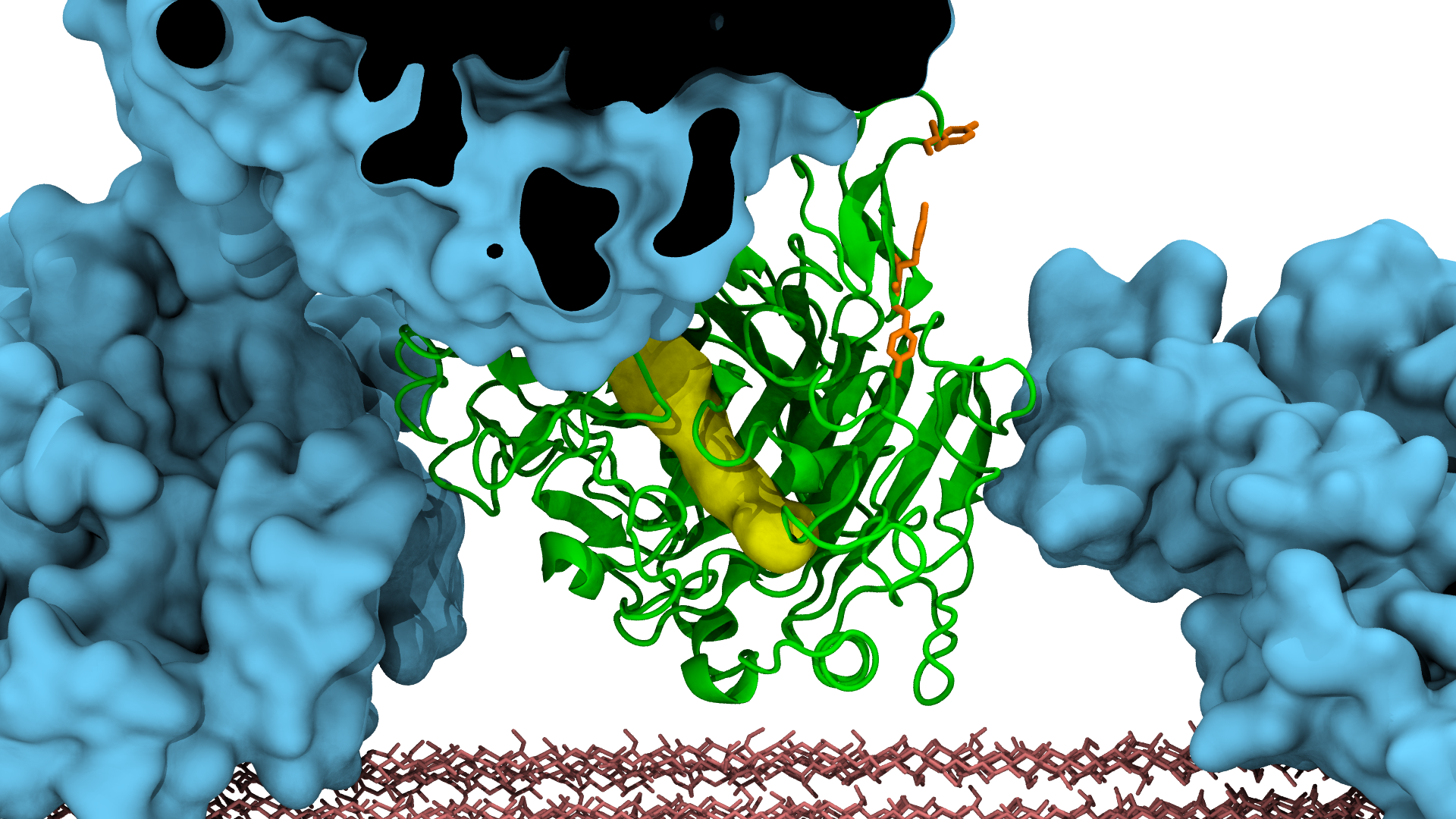

Supplement: Supplementary file 11 — 10.1186/s13068-015-0379-8 A zip archive containing a gallery of each of the cellulases that bound to cellulose in the context of their environment. Each image within the gallery is one snapshot taken from the end of the trajectory showing the relative position of each enzyme (green) that makes contact with the cellulose (red). Nearby lignins are shown in blue, and the substrate tunnel is a yellow surface to orient the viewer. The three tyrosine residues are shown in orange. Note that for each protein, there are 4 images, taken from different relative orientations to the cellulose fibril (0, 90, 180, and 270), and are labeled accordingly in their filenames. [file 13068_2015_379_MOESM11_ESM.zip › gallery/C-0_P-33_180.png]

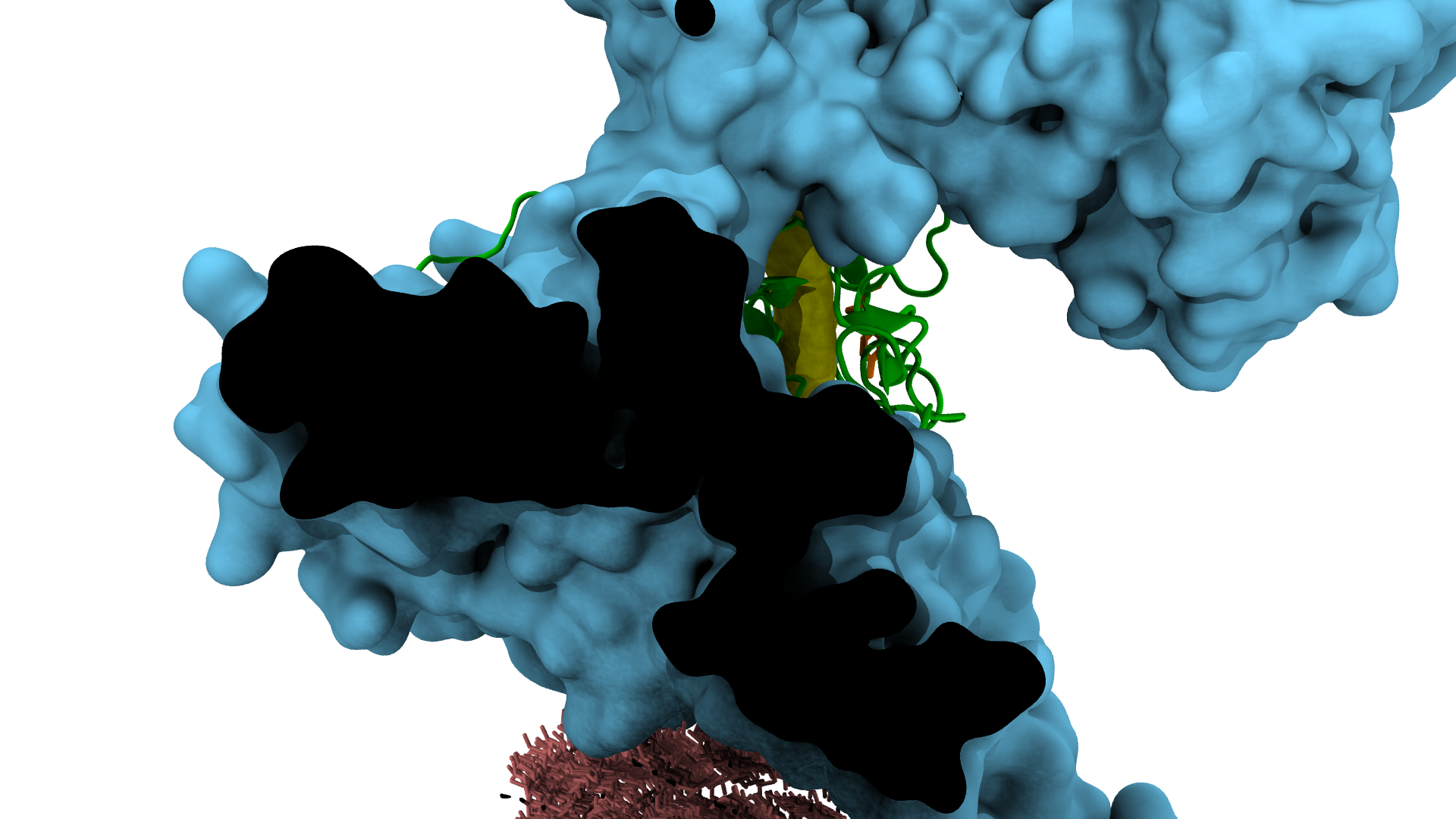

Supplement: Supplementary file 11 — 10.1186/s13068-015-0379-8 A zip archive containing a gallery of each of the cellulases that bound to cellulose in the context of their environment. Each image within the gallery is one snapshot taken from the end of the trajectory showing the relative position of each enzyme (green) that makes contact with the cellulose (red). Nearby lignins are shown in blue, and the substrate tunnel is a yellow surface to orient the viewer. The three tyrosine residues are shown in orange. Note that for each protein, there are 4 images, taken from different relative orientations to the cellulose fibril (0, 90, 180, and 270), and are labeled accordingly in their filenames. [file 13068_2015_379_MOESM11_ESM.zip › gallery/C-0_P-33_270.png]

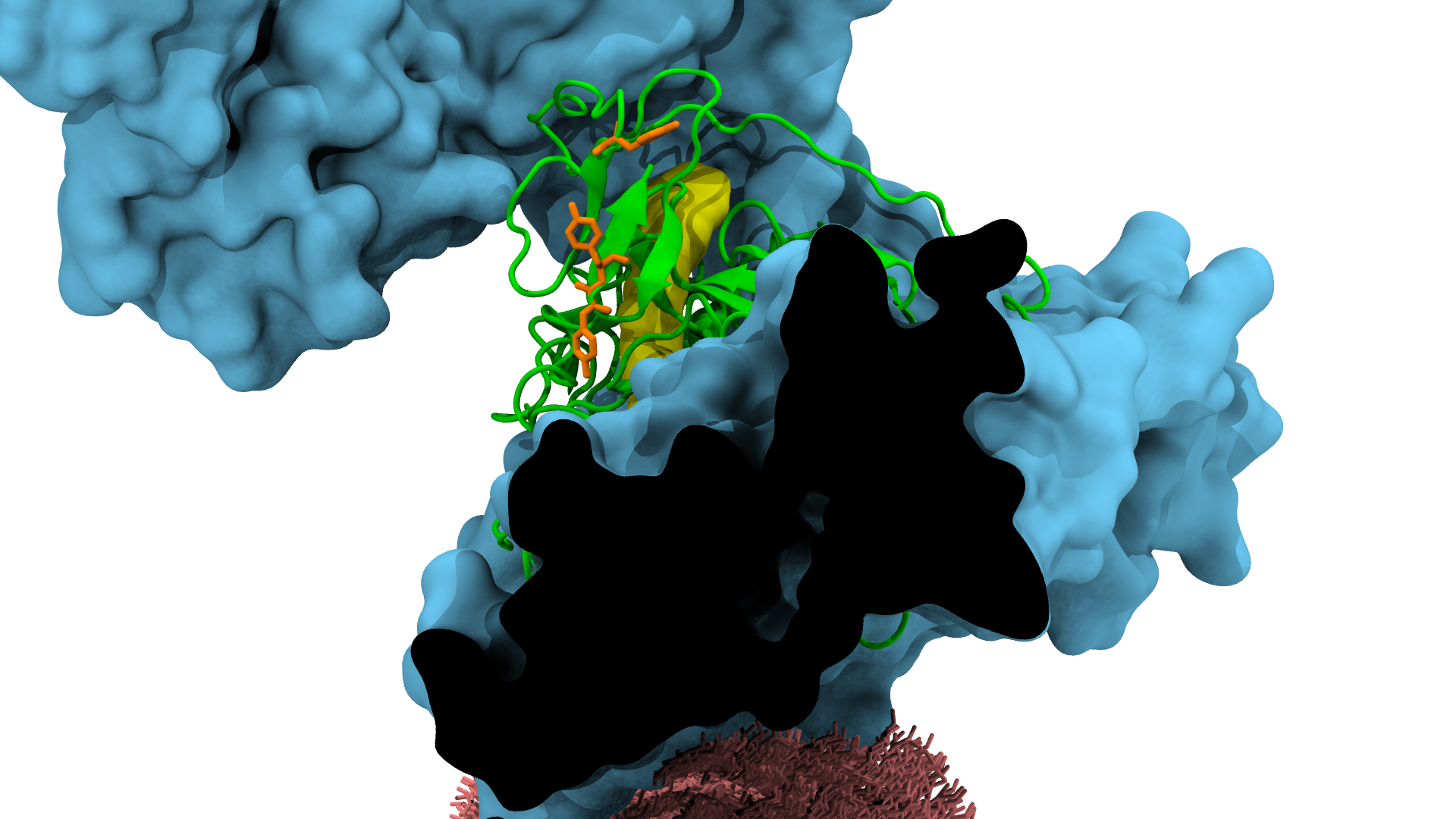

Supplement: Supplementary file 11 — 10.1186/s13068-015-0379-8 A zip archive containing a gallery of each of the cellulases that bound to cellulose in the context of their environment. Each image within the gallery is one snapshot taken from the end of the trajectory showing the relative position of each enzyme (green) that makes contact with the cellulose (red). Nearby lignins are shown in blue, and the substrate tunnel is a yellow surface to orient the viewer. The three tyrosine residues are shown in orange. Note that for each protein, there are 4 images, taken from different relative orientations to the cellulose fibril (0, 90, 180, and 270), and are labeled accordingly in their filenames. [file 13068_2015_379_MOESM11_ESM.zip › gallery/C-0_P-33_90.png]

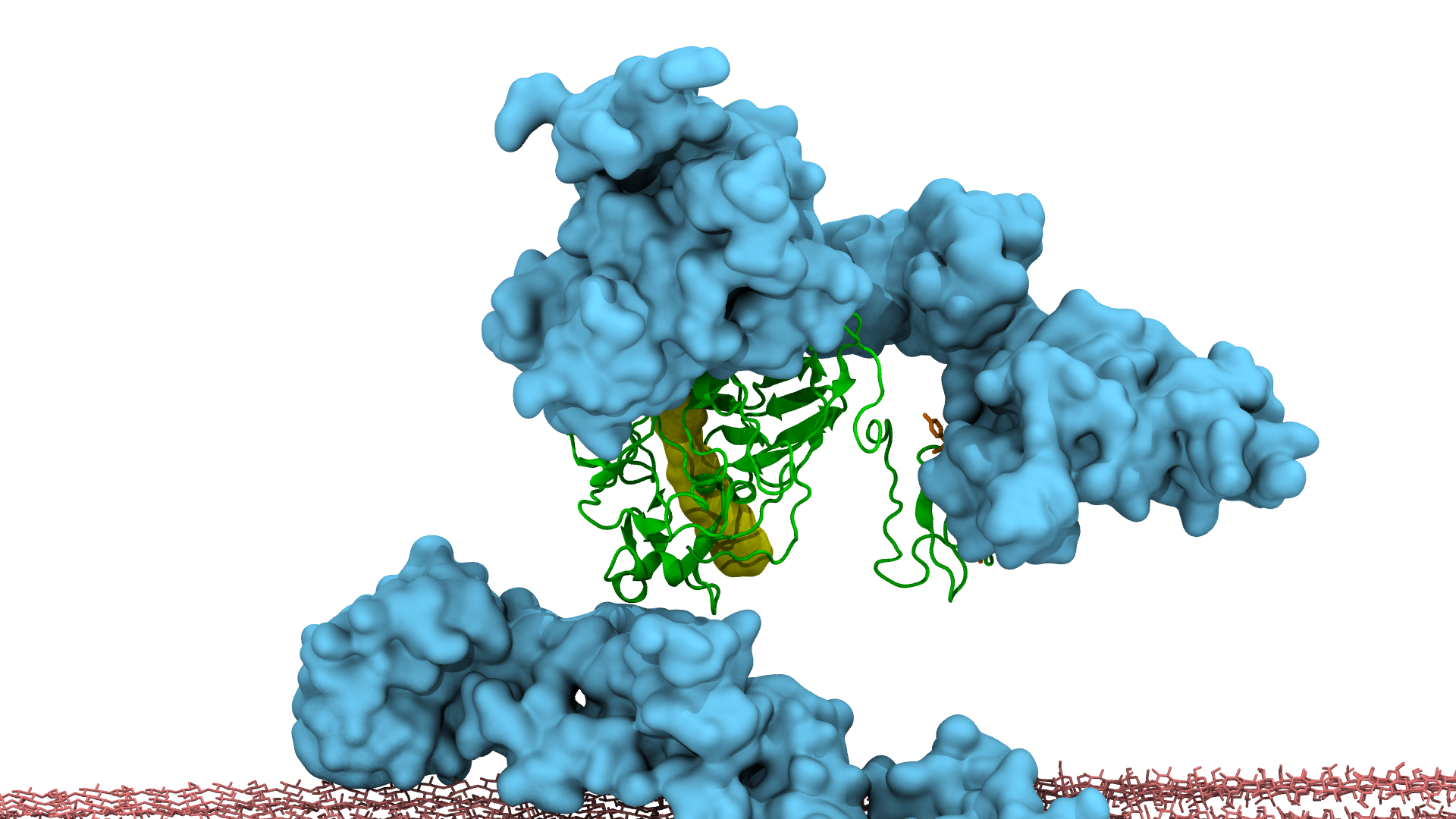

Supplement: Supplementary file 11 — 10.1186/s13068-015-0379-8 A zip archive containing a gallery of each of the cellulases that bound to cellulose in the context of their environment. Each image within the gallery is one snapshot taken from the end of the trajectory showing the relative position of each enzyme (green) that makes contact with the cellulose (red). Nearby lignins are shown in blue, and the substrate tunnel is a yellow surface to orient the viewer. The three tyrosine residues are shown in orange. Note that for each protein, there are 4 images, taken from different relative orientations to the cellulose fibril (0, 90, 180, and 270), and are labeled accordingly in their filenames. [file 13068_2015_379_MOESM11_ESM.zip › gallery/C-0_P-43_0.png]

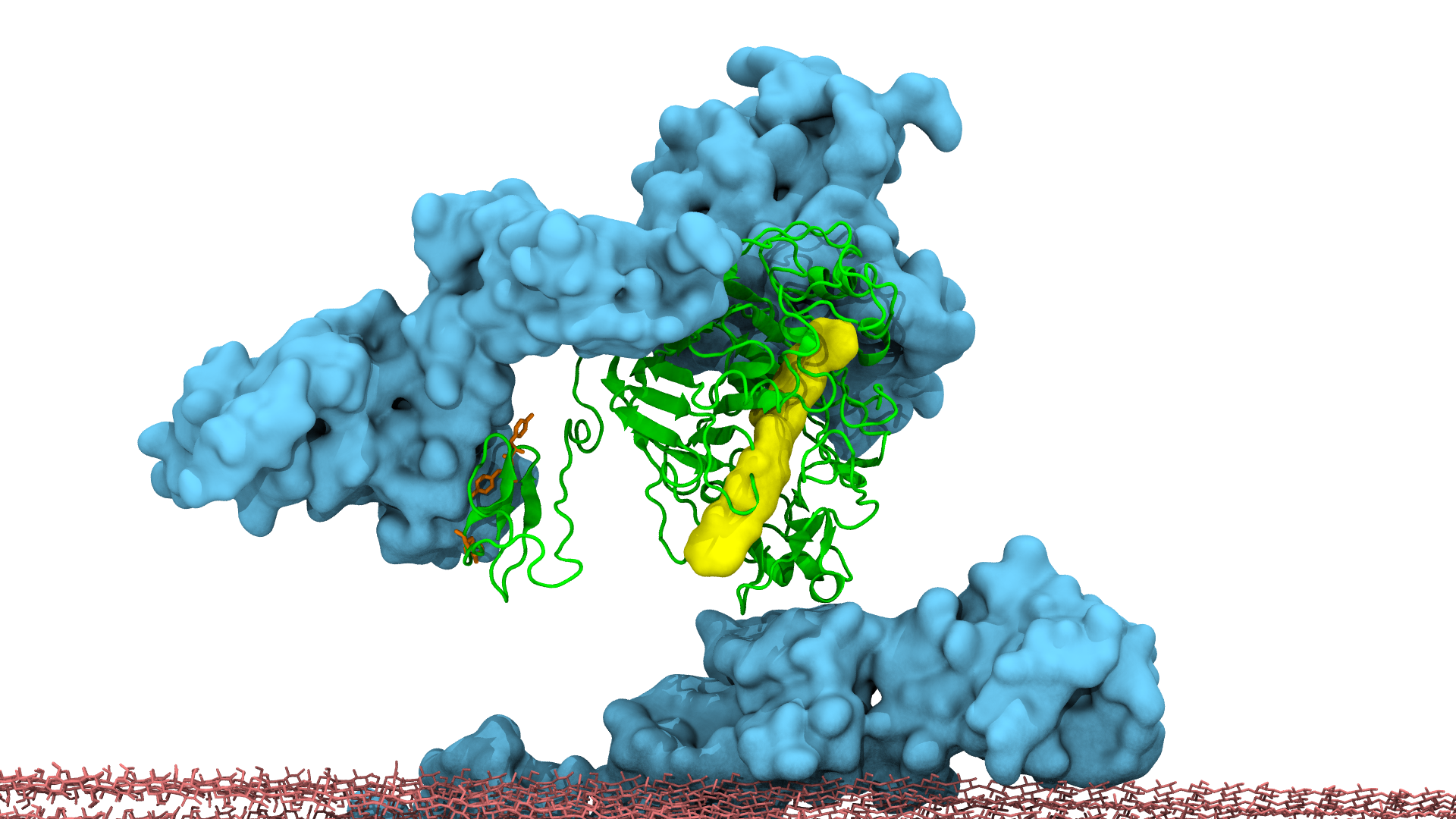

Supplement: Supplementary file 11 — 10.1186/s13068-015-0379-8 A zip archive containing a gallery of each of the cellulases that bound to cellulose in the context of their environment. Each image within the gallery is one snapshot taken from the end of the trajectory showing the relative position of each enzyme (green) that makes contact with the cellulose (red). Nearby lignins are shown in blue, and the substrate tunnel is a yellow surface to orient the viewer. The three tyrosine residues are shown in orange. Note that for each protein, there are 4 images, taken from different relative orientations to the cellulose fibril (0, 90, 180, and 270), and are labeled accordingly in their filenames. [file 13068_2015_379_MOESM11_ESM.zip › gallery/C-0_P-43_180.png]

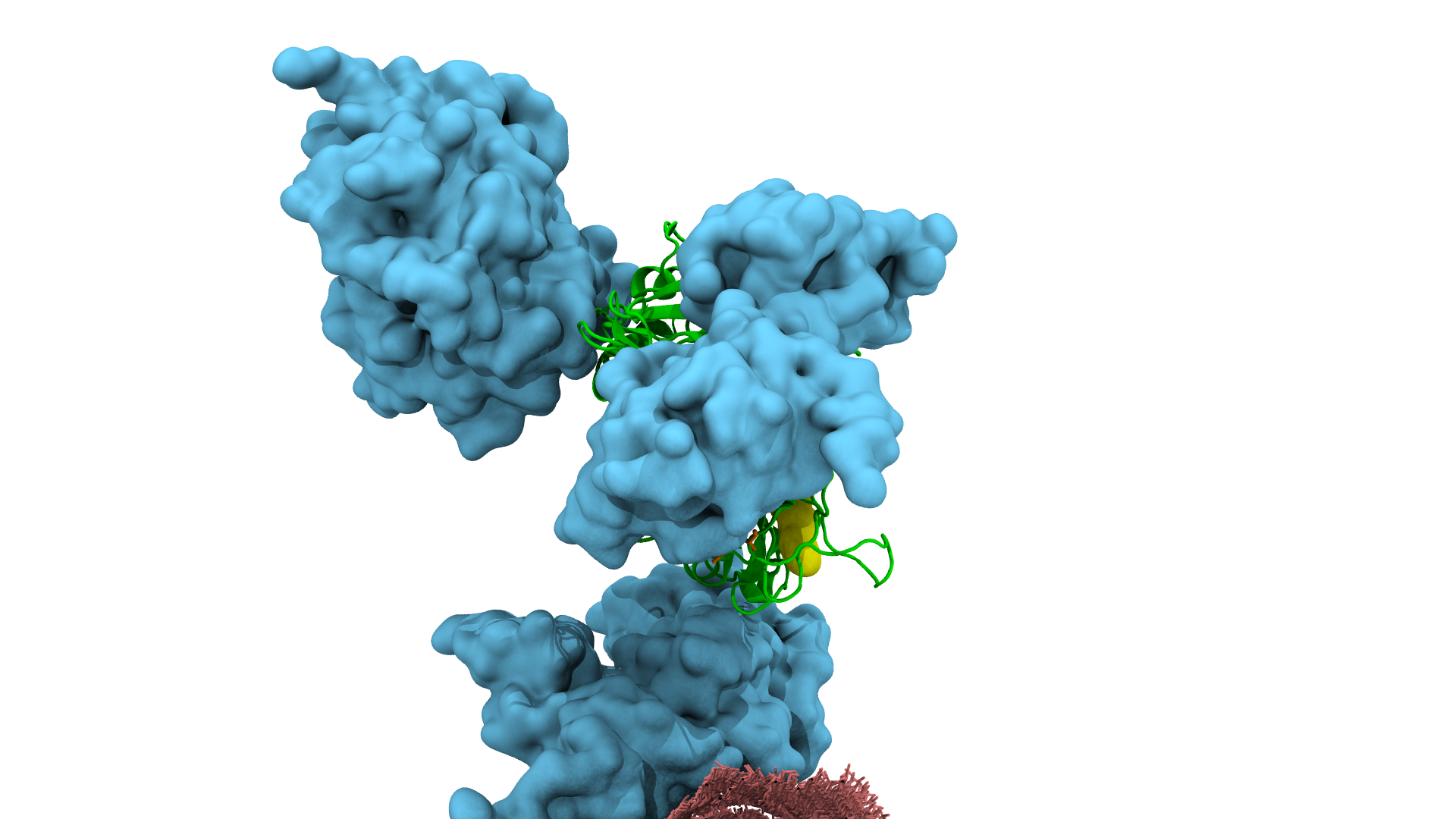

Supplement: Supplementary file 11 — 10.1186/s13068-015-0379-8 A zip archive containing a gallery of each of the cellulases that bound to cellulose in the context of their environment. Each image within the gallery is one snapshot taken from the end of the trajectory showing the relative position of each enzyme (green) that makes contact with the cellulose (red). Nearby lignins are shown in blue, and the substrate tunnel is a yellow surface to orient the viewer. The three tyrosine residues are shown in orange. Note that for each protein, there are 4 images, taken from different relative orientations to the cellulose fibril (0, 90, 180, and 270), and are labeled accordingly in their filenames. [file 13068_2015_379_MOESM11_ESM.zip › gallery/C-0_P-43_270.png]

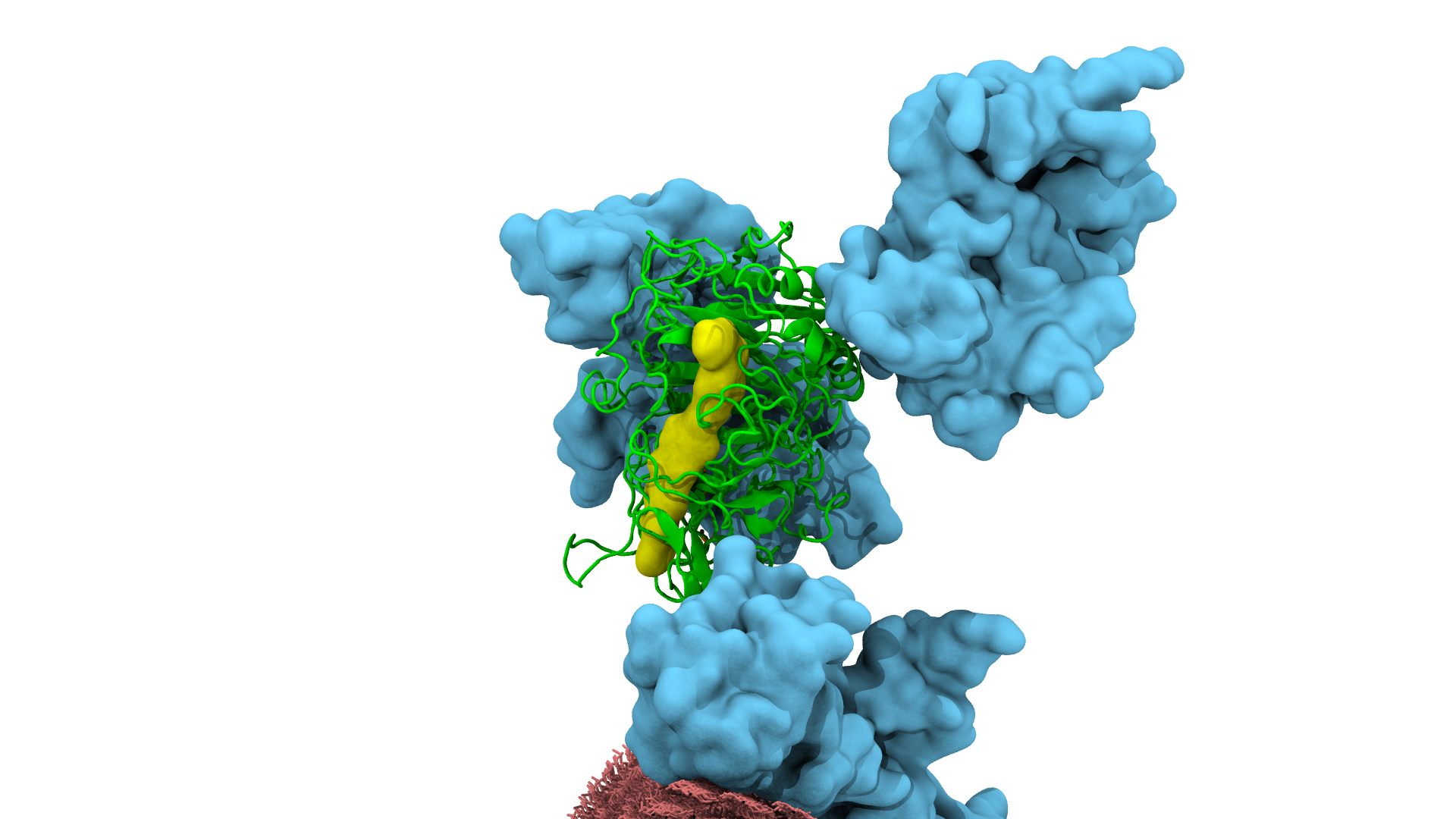

Supplement: Supplementary file 11 — 10.1186/s13068-015-0379-8 A zip archive containing a gallery of each of the cellulases that bound to cellulose in the context of their environment. Each image within the gallery is one snapshot taken from the end of the trajectory showing the relative position of each enzyme (green) that makes contact with the cellulose (red). Nearby lignins are shown in blue, and the substrate tunnel is a yellow surface to orient the viewer. The three tyrosine residues are shown in orange. Note that for each protein, there are 4 images, taken from different relative orientations to the cellulose fibril (0, 90, 180, and 270), and are labeled accordingly in their filenames. [file 13068_2015_379_MOESM11_ESM.zip › gallery/C-0_P-43_90.png]

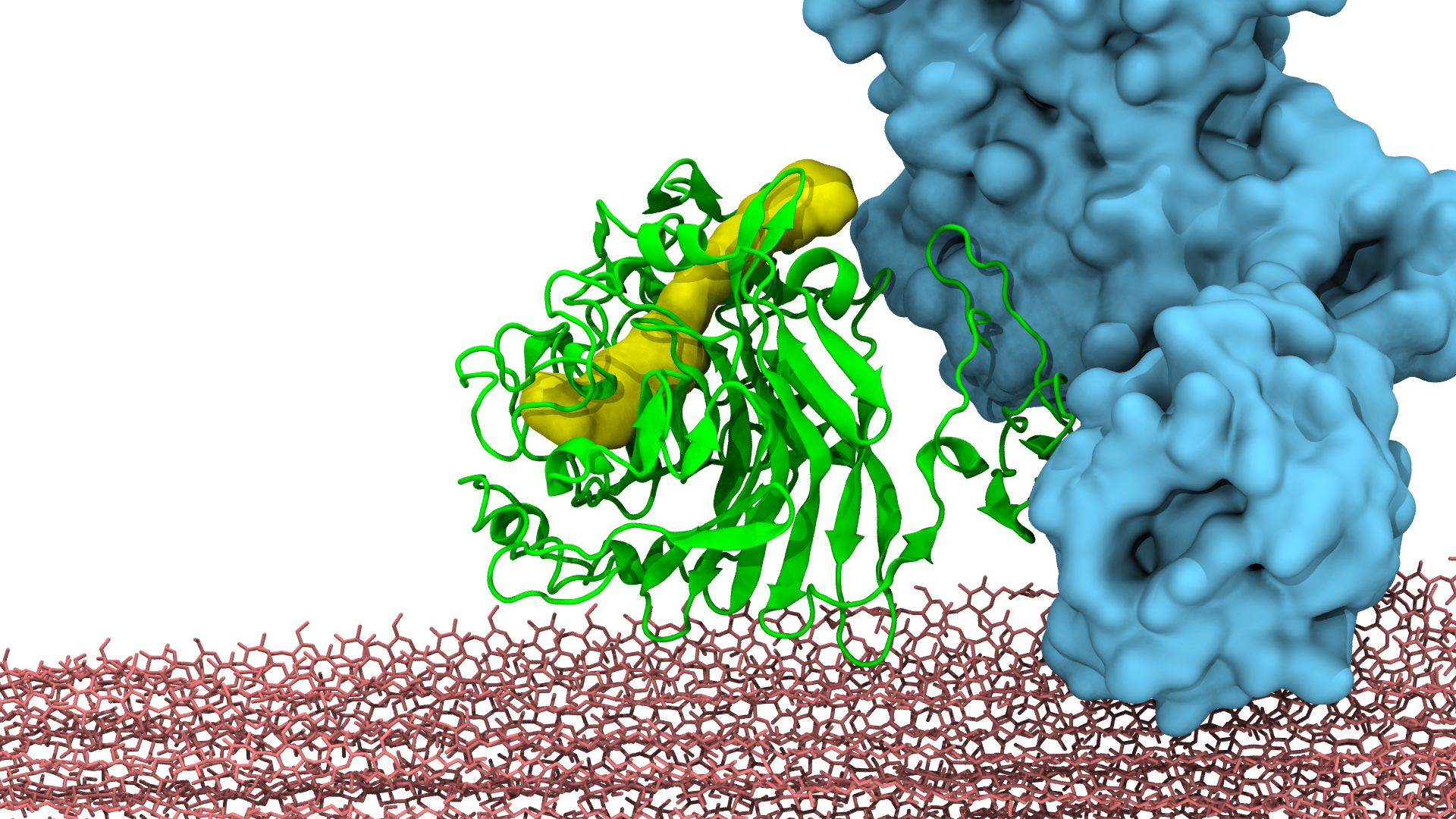

Supplement: Supplementary file 11 — 10.1186/s13068-015-0379-8 A zip archive containing a gallery of each of the cellulases that bound to cellulose in the context of their environment. Each image within the gallery is one snapshot taken from the end of the trajectory showing the relative position of each enzyme (green) that makes contact with the cellulose (red). Nearby lignins are shown in blue, and the substrate tunnel is a yellow surface to orient the viewer. The three tyrosine residues are shown in orange. Note that for each protein, there are 4 images, taken from different relative orientations to the cellulose fibril (0, 90, 180, and 270), and are labeled accordingly in their filenames. [file 13068_2015_379_MOESM11_ESM.zip › gallery/C-1_P-00_0.png]

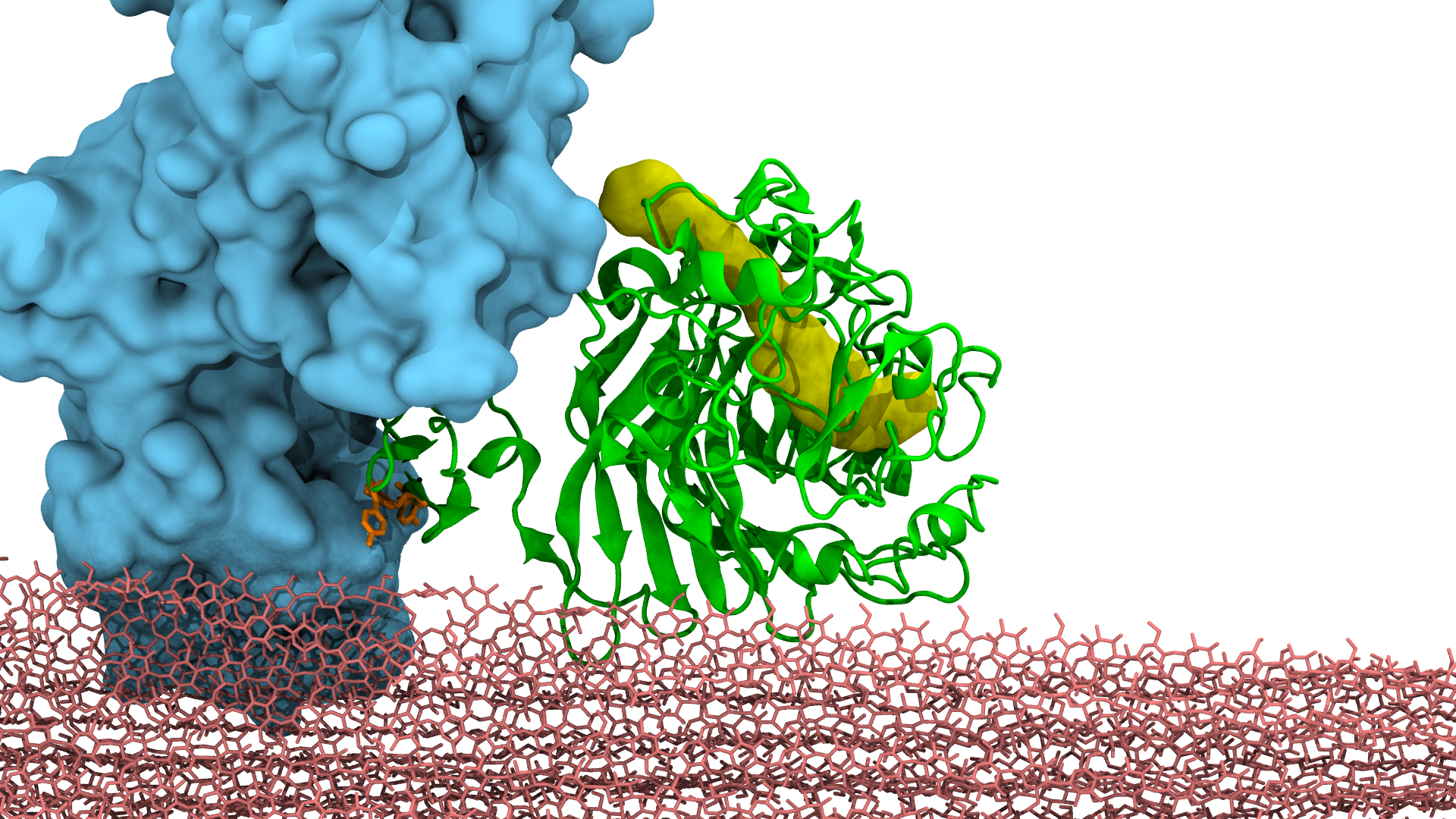

Supplement: Supplementary file 11 — 10.1186/s13068-015-0379-8 A zip archive containing a gallery of each of the cellulases that bound to cellulose in the context of their environment. Each image within the gallery is one snapshot taken from the end of the trajectory showing the relative position of each enzyme (green) that makes contact with the cellulose (red). Nearby lignins are shown in blue, and the substrate tunnel is a yellow surface to orient the viewer. The three tyrosine residues are shown in orange. Note that for each protein, there are 4 images, taken from different relative orientations to the cellulose fibril (0, 90, 180, and 270), and are labeled accordingly in their filenames. [file 13068_2015_379_MOESM11_ESM.zip › gallery/C-1_P-00_180.png]

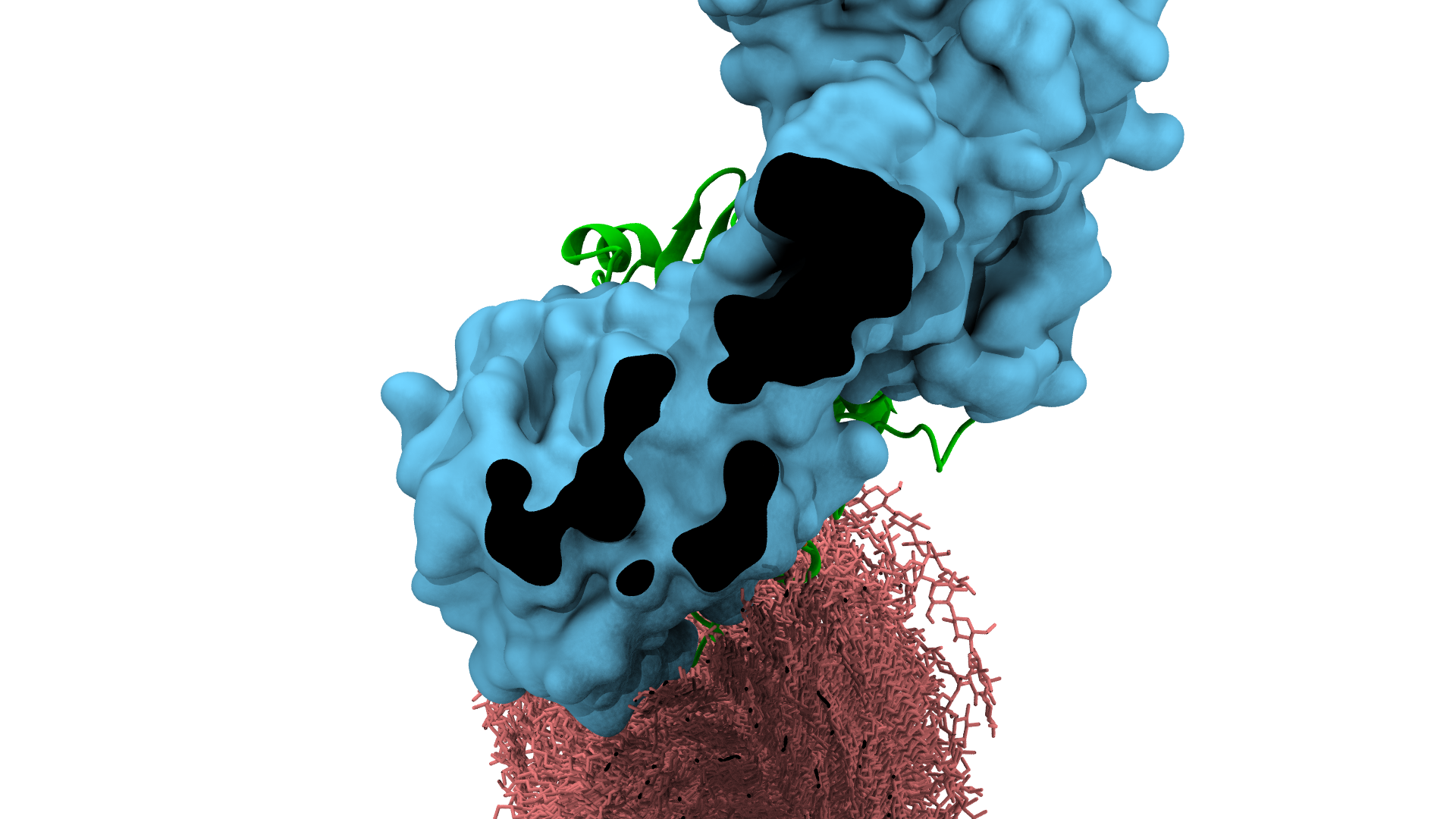

Supplement: Supplementary file 11 — 10.1186/s13068-015-0379-8 A zip archive containing a gallery of each of the cellulases that bound to cellulose in the context of their environment. Each image within the gallery is one snapshot taken from the end of the trajectory showing the relative position of each enzyme (green) that makes contact with the cellulose (red). Nearby lignins are shown in blue, and the substrate tunnel is a yellow surface to orient the viewer. The three tyrosine residues are shown in orange. Note that for each protein, there are 4 images, taken from different relative orientations to the cellulose fibril (0, 90, 180, and 270), and are labeled accordingly in their filenames. [file 13068_2015_379_MOESM11_ESM.zip › gallery/C-1_P-00_270.png]

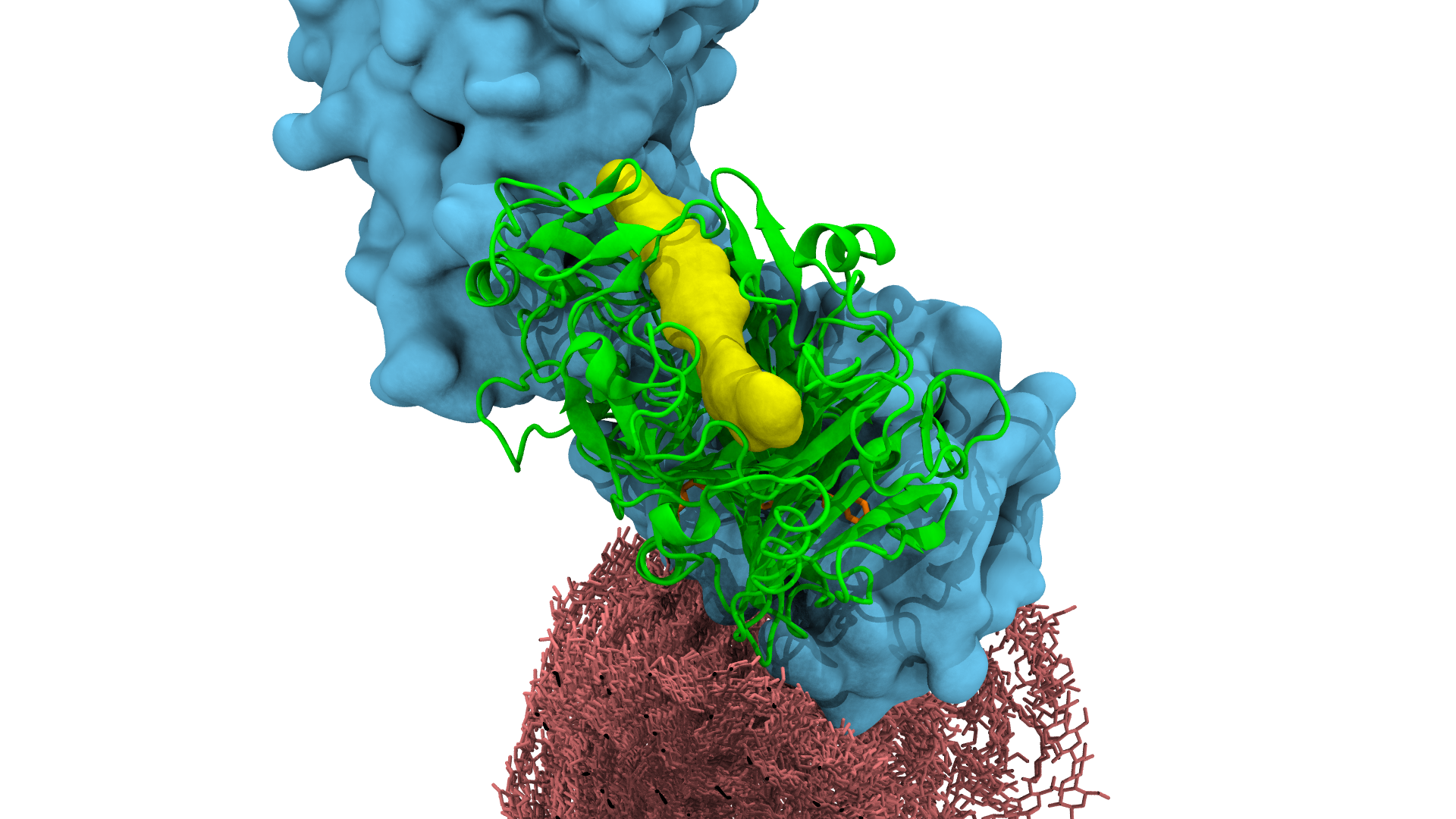

Supplement: Supplementary file 11 — 10.1186/s13068-015-0379-8 A zip archive containing a gallery of each of the cellulases that bound to cellulose in the context of their environment. Each image within the gallery is one snapshot taken from the end of the trajectory showing the relative position of each enzyme (green) that makes contact with the cellulose (red). Nearby lignins are shown in blue, and the substrate tunnel is a yellow surface to orient the viewer. The three tyrosine residues are shown in orange. Note that for each protein, there are 4 images, taken from different relative orientations to the cellulose fibril (0, 90, 180, and 270), and are labeled accordingly in their filenames. [file 13068_2015_379_MOESM11_ESM.zip › gallery/C-1_P-00_90.png]

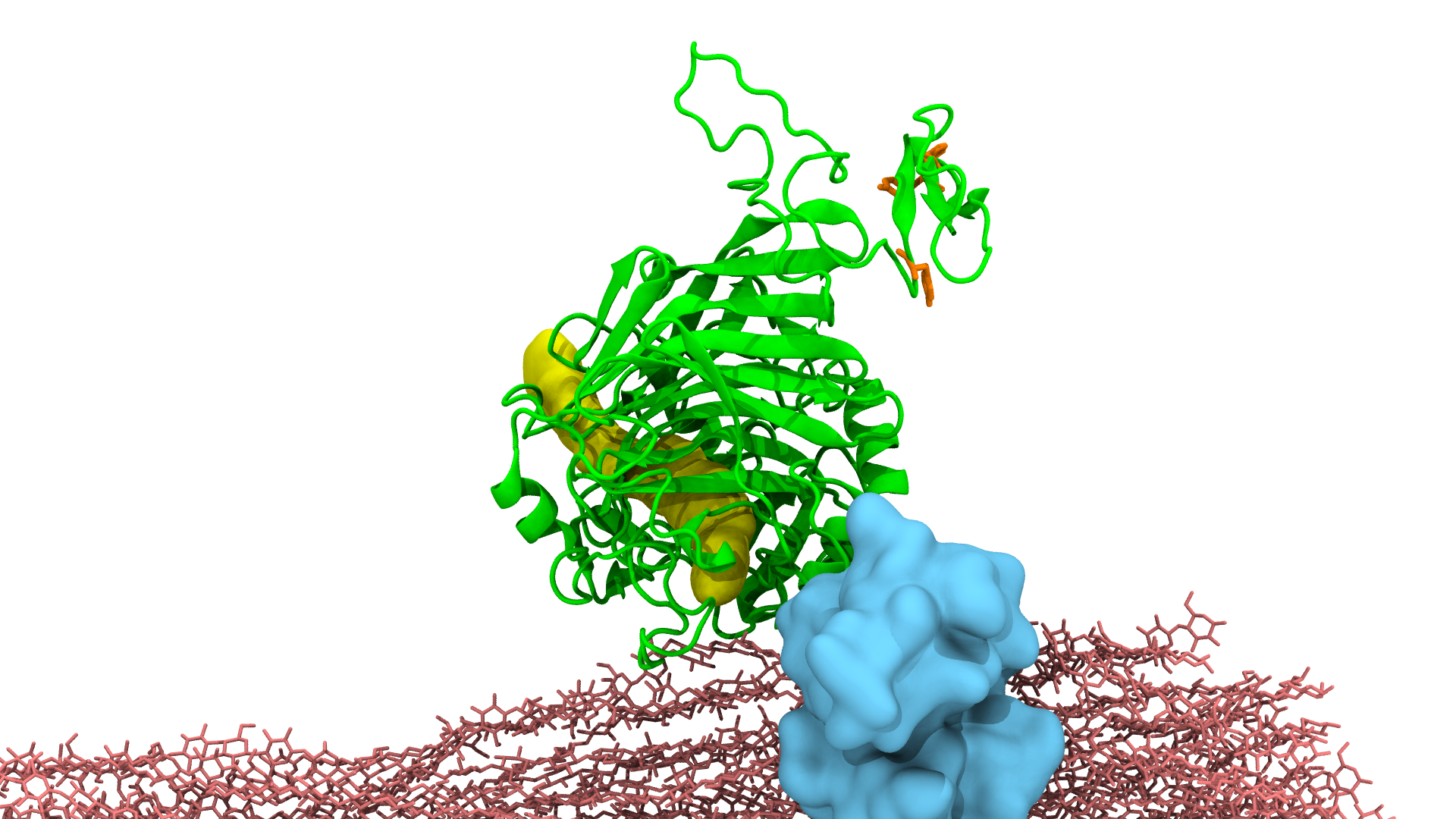

Supplement: Supplementary file 11 — 10.1186/s13068-015-0379-8 A zip archive containing a gallery of each of the cellulases that bound to cellulose in the context of their environment. Each image within the gallery is one snapshot taken from the end of the trajectory showing the relative position of each enzyme (green) that makes contact with the cellulose (red). Nearby lignins are shown in blue, and the substrate tunnel is a yellow surface to orient the viewer. The three tyrosine residues are shown in orange. Note that for each protein, there are 4 images, taken from different relative orientations to the cellulose fibril (0, 90, 180, and 270), and are labeled accordingly in their filenames. [file 13068_2015_379_MOESM11_ESM.zip › gallery/C-1_P-22_0.png]

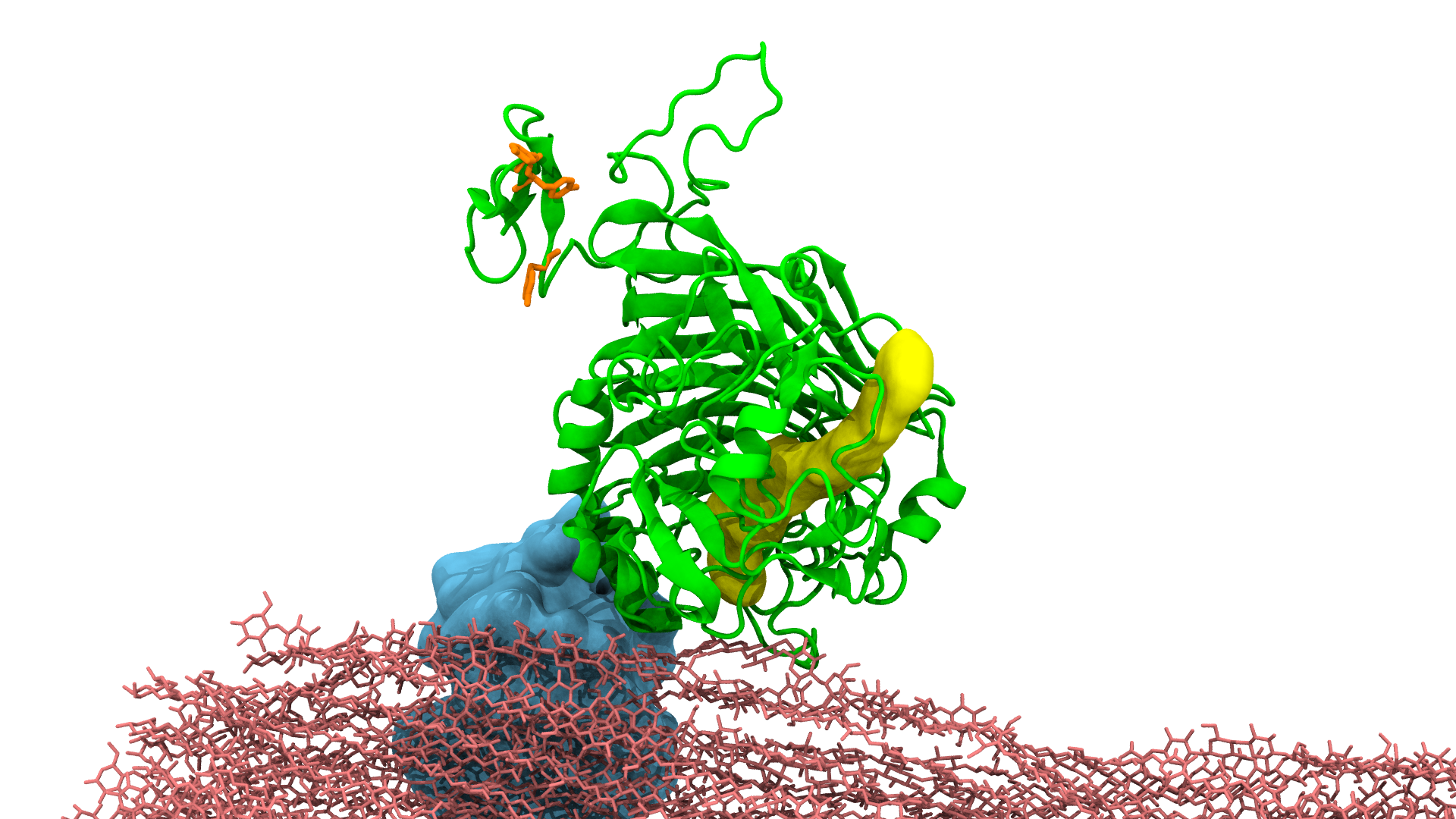

Supplement: Supplementary file 11 — 10.1186/s13068-015-0379-8 A zip archive containing a gallery of each of the cellulases that bound to cellulose in the context of their environment. Each image within the gallery is one snapshot taken from the end of the trajectory showing the relative position of each enzyme (green) that makes contact with the cellulose (red). Nearby lignins are shown in blue, and the substrate tunnel is a yellow surface to orient the viewer. The three tyrosine residues are shown in orange. Note that for each protein, there are 4 images, taken from different relative orientations to the cellulose fibril (0, 90, 180, and 270), and are labeled accordingly in their filenames. [file 13068_2015_379_MOESM11_ESM.zip › gallery/C-1_P-22_180.png]

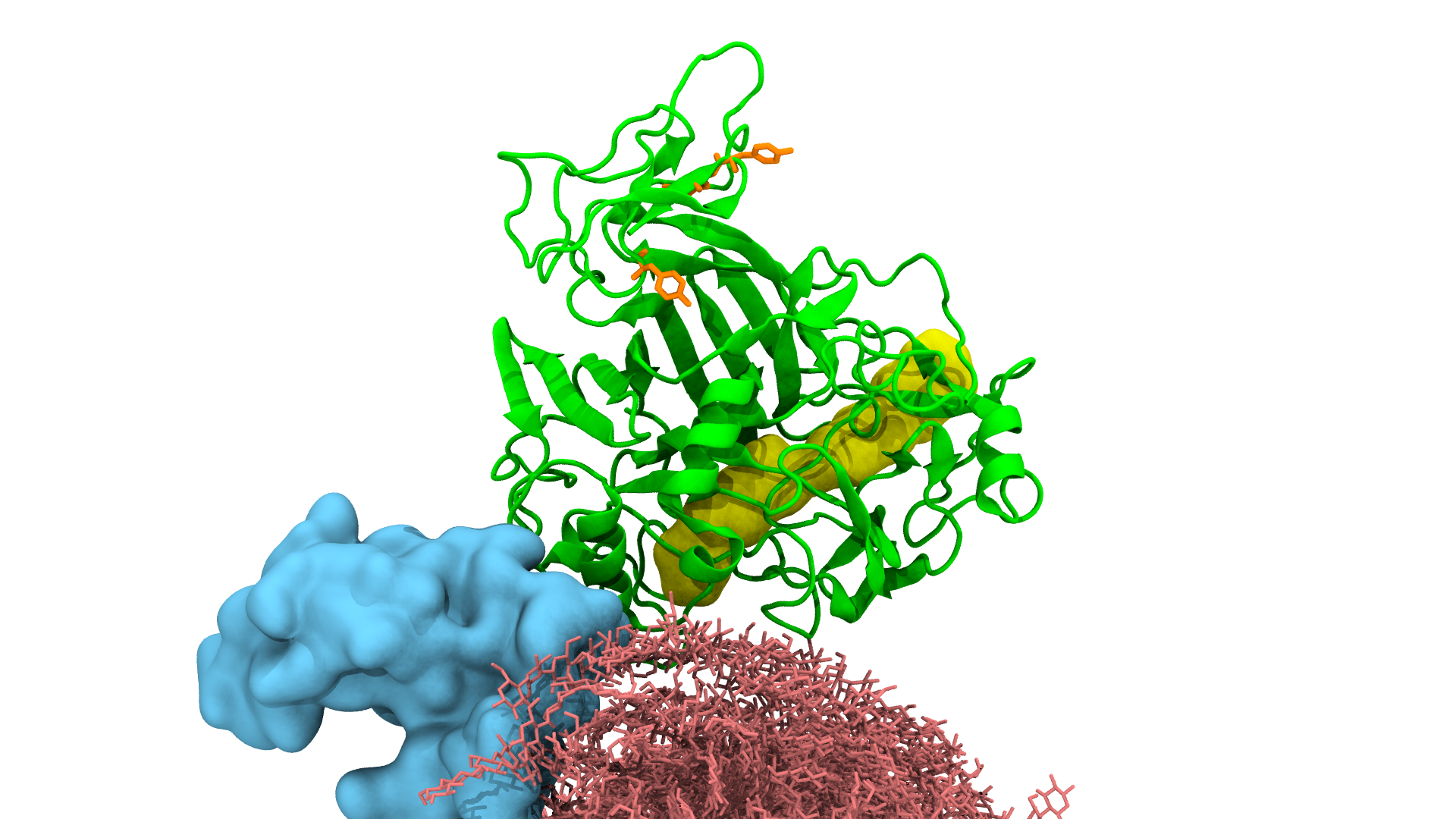

Supplement: Supplementary file 11 — 10.1186/s13068-015-0379-8 A zip archive containing a gallery of each of the cellulases that bound to cellulose in the context of their environment. Each image within the gallery is one snapshot taken from the end of the trajectory showing the relative position of each enzyme (green) that makes contact with the cellulose (red). Nearby lignins are shown in blue, and the substrate tunnel is a yellow surface to orient the viewer. The three tyrosine residues are shown in orange. Note that for each protein, there are 4 images, taken from different relative orientations to the cellulose fibril (0, 90, 180, and 270), and are labeled accordingly in their filenames. [file 13068_2015_379_MOESM11_ESM.zip › gallery/C-1_P-22_270.png]

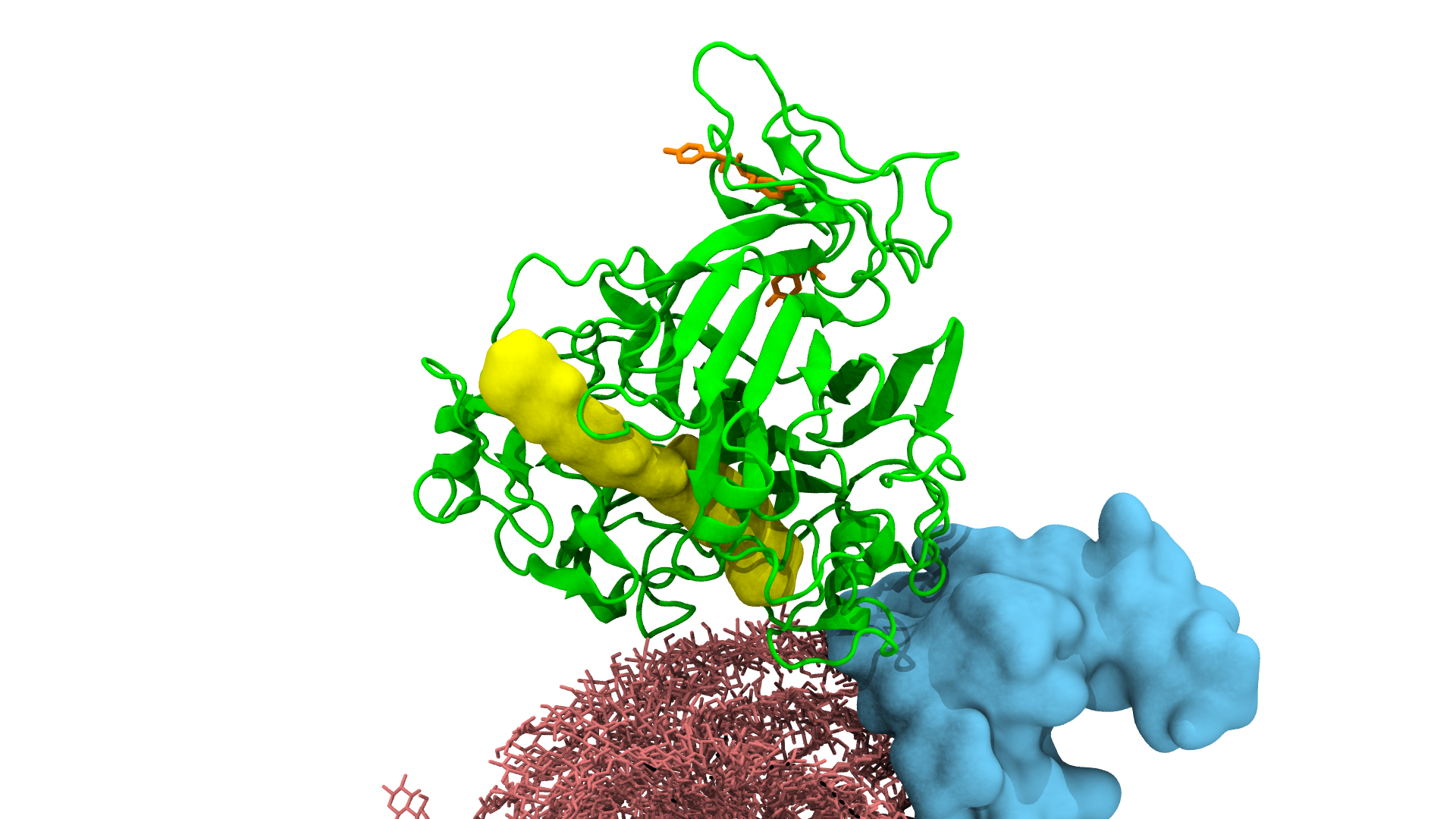

Supplement: Supplementary file 11 — 10.1186/s13068-015-0379-8 A zip archive containing a gallery of each of the cellulases that bound to cellulose in the context of their environment. Each image within the gallery is one snapshot taken from the end of the trajectory showing the relative position of each enzyme (green) that makes contact with the cellulose (red). Nearby lignins are shown in blue, and the substrate tunnel is a yellow surface to orient the viewer. The three tyrosine residues are shown in orange. Note that for each protein, there are 4 images, taken from different relative orientations to the cellulose fibril (0, 90, 180, and 270), and are labeled accordingly in their filenames. [file 13068_2015_379_MOESM11_ESM.zip › gallery/C-1_P-22_90.png]

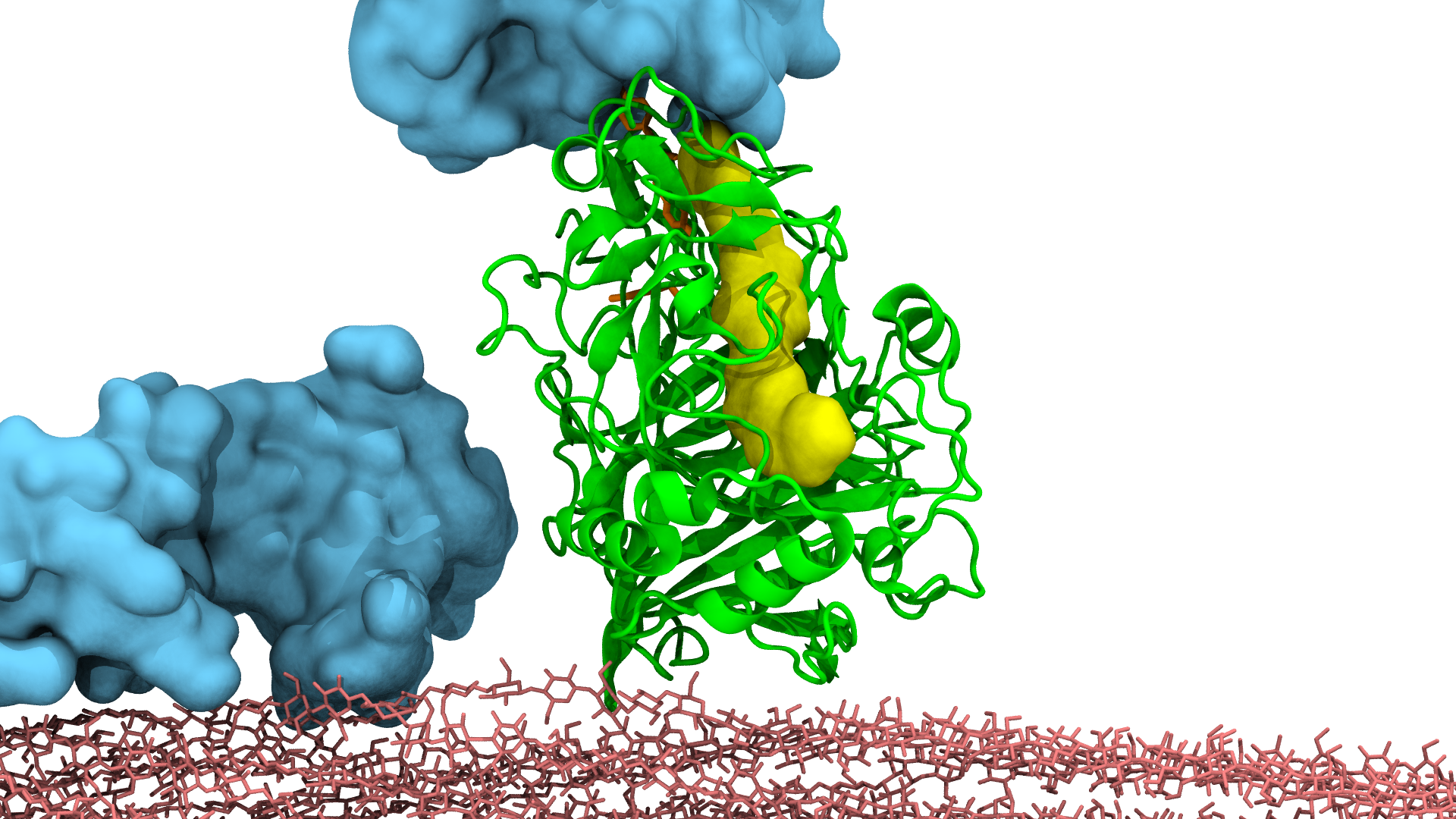

Supplement: Supplementary file 11 — 10.1186/s13068-015-0379-8 A zip archive containing a gallery of each of the cellulases that bound to cellulose in the context of their environment. Each image within the gallery is one snapshot taken from the end of the trajectory showing the relative position of each enzyme (green) that makes contact with the cellulose (red). Nearby lignins are shown in blue, and the substrate tunnel is a yellow surface to orient the viewer. The three tyrosine residues are shown in orange. Note that for each protein, there are 4 images, taken from different relative orientations to the cellulose fibril (0, 90, 180, and 270), and are labeled accordingly in their filenames. [file 13068_2015_379_MOESM11_ESM.zip › gallery/C-1_P-34_0.png]

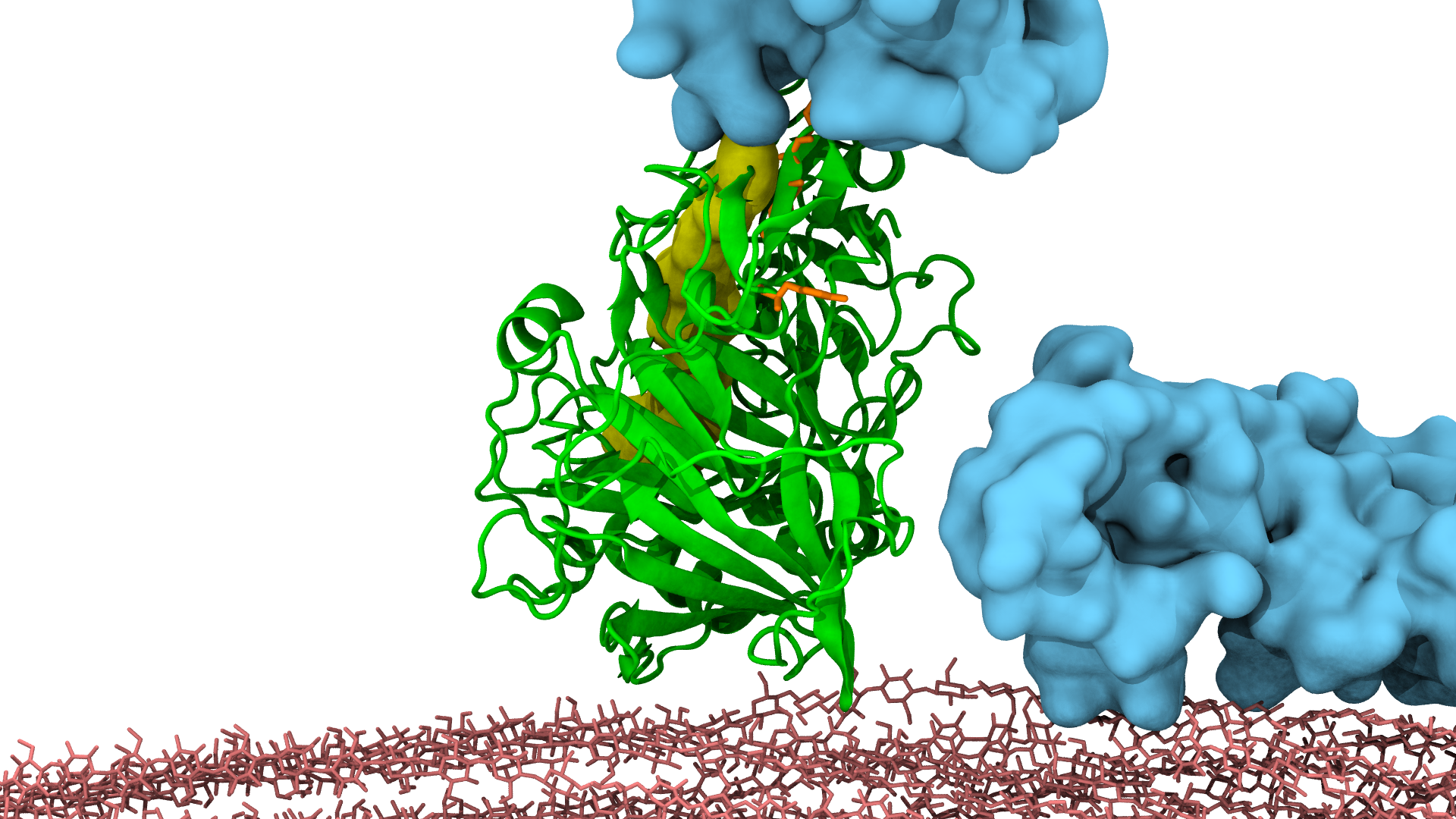

Supplement: Supplementary file 11 — 10.1186/s13068-015-0379-8 A zip archive containing a gallery of each of the cellulases that bound to cellulose in the context of their environment. Each image within the gallery is one snapshot taken from the end of the trajectory showing the relative position of each enzyme (green) that makes contact with the cellulose (red). Nearby lignins are shown in blue, and the substrate tunnel is a yellow surface to orient the viewer. The three tyrosine residues are shown in orange. Note that for each protein, there are 4 images, taken from different relative orientations to the cellulose fibril (0, 90, 180, and 270), and are labeled accordingly in their filenames. [file 13068_2015_379_MOESM11_ESM.zip › gallery/C-1_P-34_180.png]

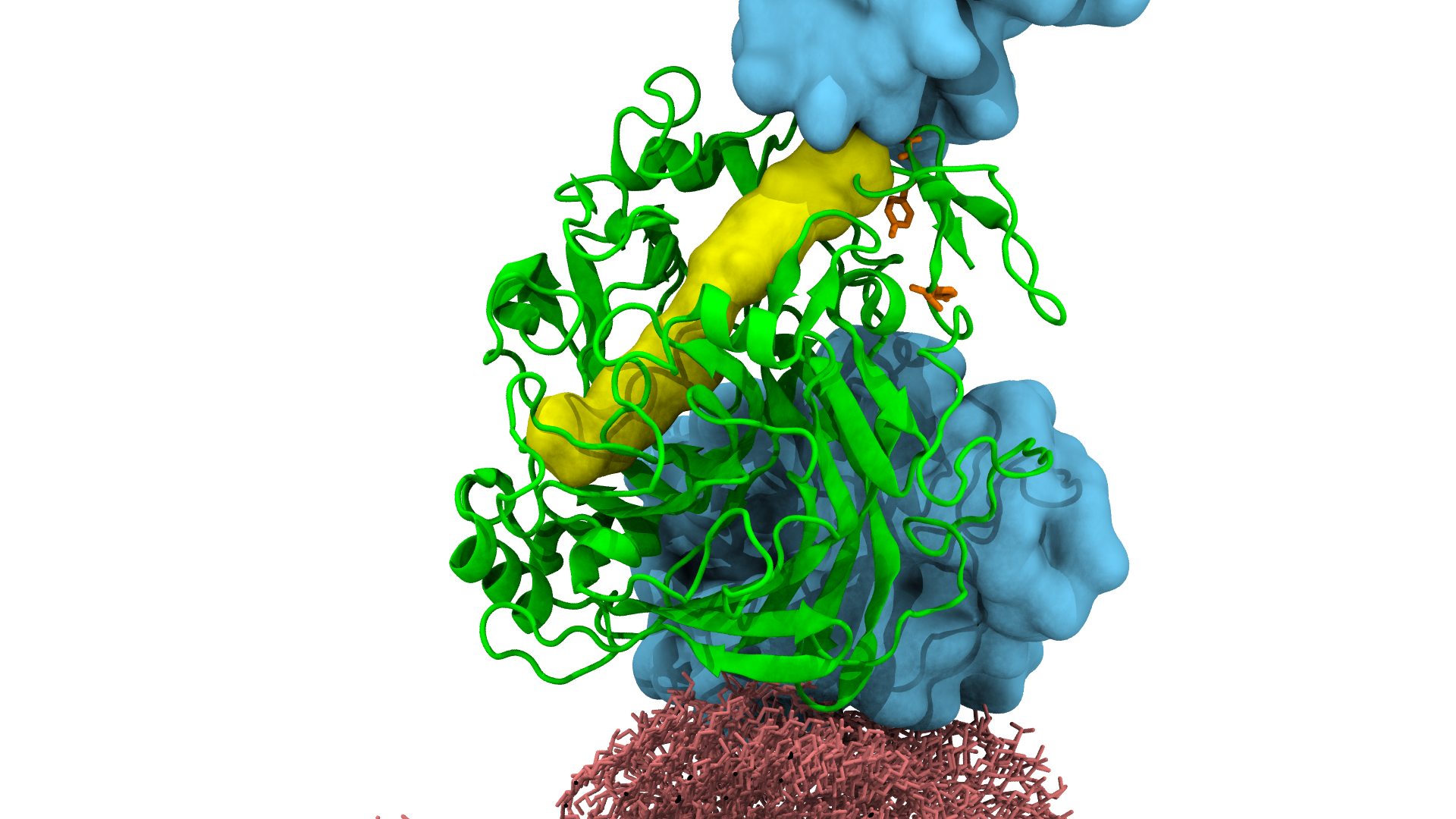

Supplement: Supplementary file 11 — 10.1186/s13068-015-0379-8 A zip archive containing a gallery of each of the cellulases that bound to cellulose in the context of their environment. Each image within the gallery is one snapshot taken from the end of the trajectory showing the relative position of each enzyme (green) that makes contact with the cellulose (red). Nearby lignins are shown in blue, and the substrate tunnel is a yellow surface to orient the viewer. The three tyrosine residues are shown in orange. Note that for each protein, there are 4 images, taken from different relative orientations to the cellulose fibril (0, 90, 180, and 270), and are labeled accordingly in their filenames. [file 13068_2015_379_MOESM11_ESM.zip › gallery/C-1_P-34_270.png]

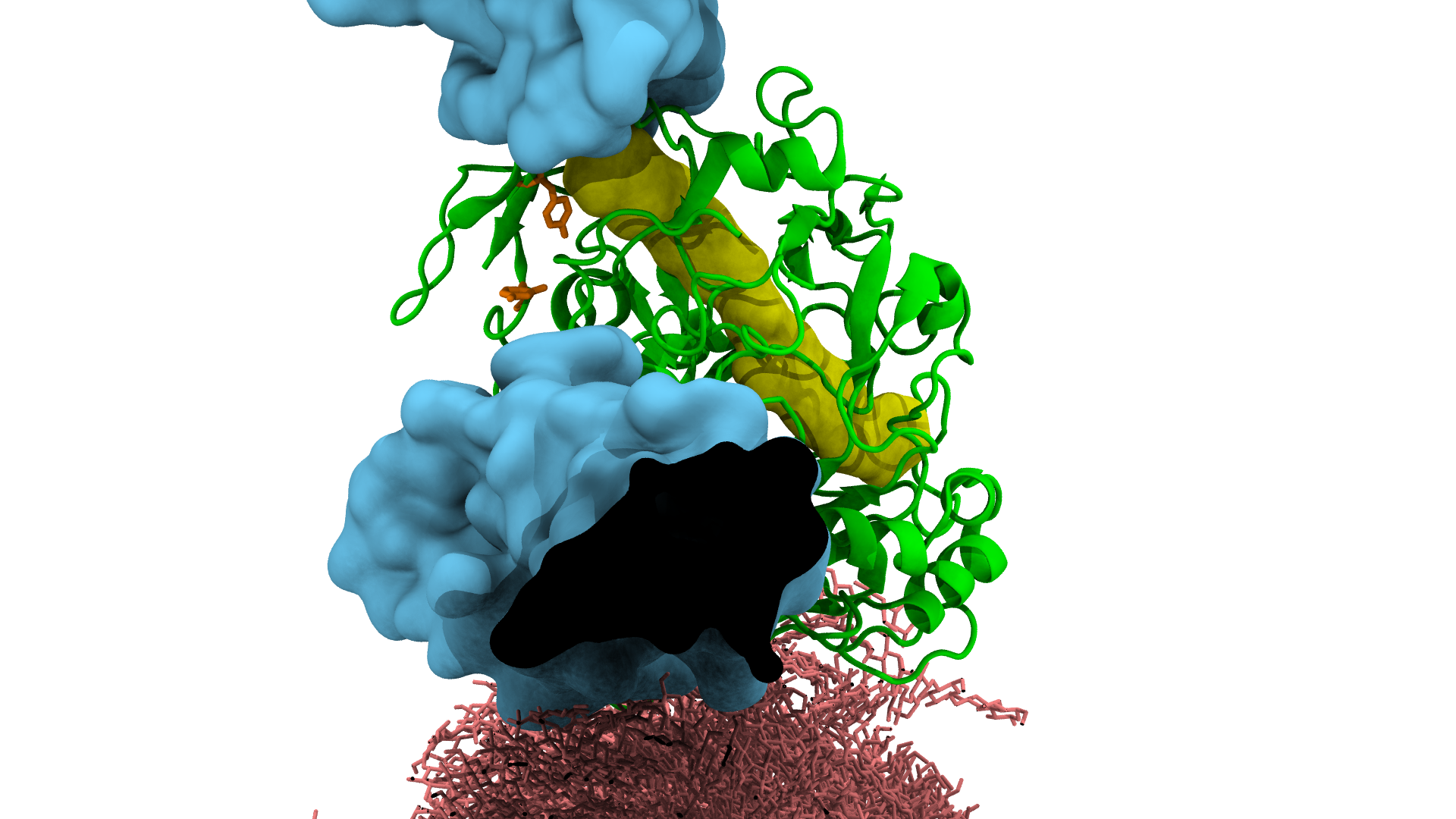

Supplement: Supplementary file 11 — 10.1186/s13068-015-0379-8 A zip archive containing a gallery of each of the cellulases that bound to cellulose in the context of their environment. Each image within the gallery is one snapshot taken from the end of the trajectory showing the relative position of each enzyme (green) that makes contact with the cellulose (red). Nearby lignins are shown in blue, and the substrate tunnel is a yellow surface to orient the viewer. The three tyrosine residues are shown in orange. Note that for each protein, there are 4 images, taken from different relative orientations to the cellulose fibril (0, 90, 180, and 270), and are labeled accordingly in their filenames. [file 13068_2015_379_MOESM11_ESM.zip › gallery/C-1_P-34_90.png]

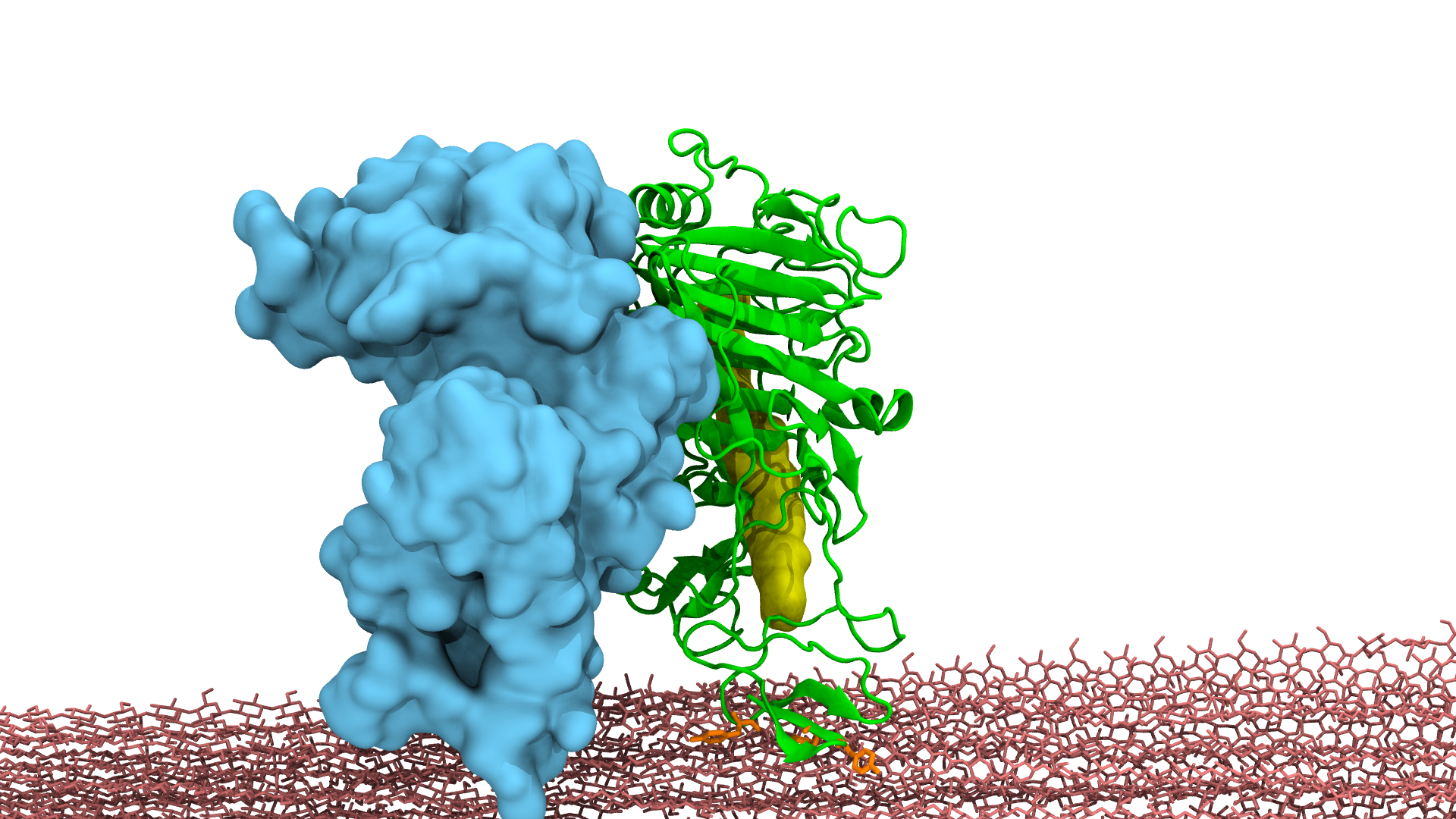

Supplement: Supplementary file 11 — 10.1186/s13068-015-0379-8 A zip archive containing a gallery of each of the cellulases that bound to cellulose in the context of their environment. Each image within the gallery is one snapshot taken from the end of the trajectory showing the relative position of each enzyme (green) that makes contact with the cellulose (red). Nearby lignins are shown in blue, and the substrate tunnel is a yellow surface to orient the viewer. The three tyrosine residues are shown in orange. Note that for each protein, there are 4 images, taken from different relative orientations to the cellulose fibril (0, 90, 180, and 270), and are labeled accordingly in their filenames. [file 13068_2015_379_MOESM11_ESM.zip › gallery/C-1_P-36_0.png]

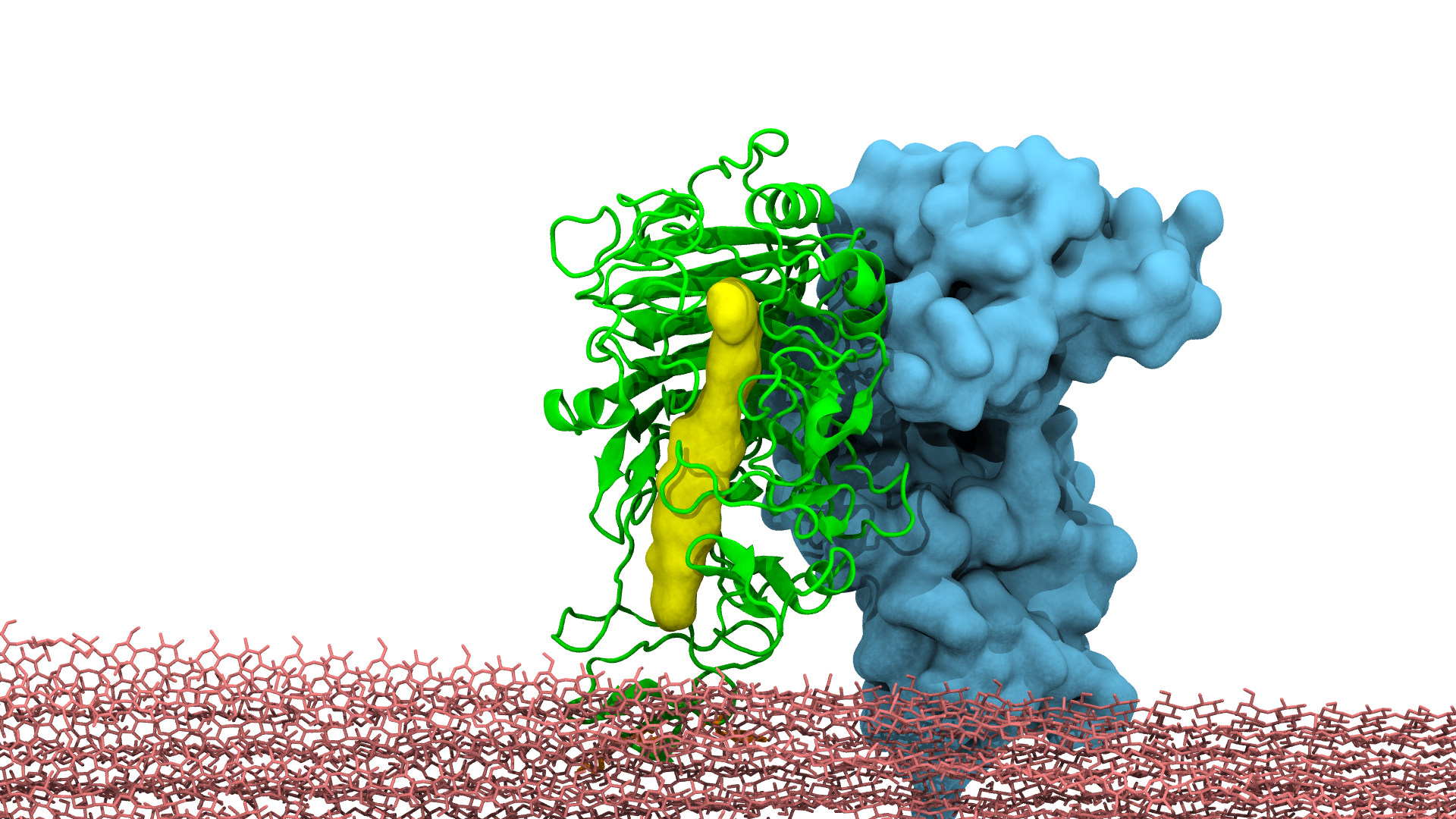

Supplement: Supplementary file 11 — 10.1186/s13068-015-0379-8 A zip archive containing a gallery of each of the cellulases that bound to cellulose in the context of their environment. Each image within the gallery is one snapshot taken from the end of the trajectory showing the relative position of each enzyme (green) that makes contact with the cellulose (red). Nearby lignins are shown in blue, and the substrate tunnel is a yellow surface to orient the viewer. The three tyrosine residues are shown in orange. Note that for each protein, there are 4 images, taken from different relative orientations to the cellulose fibril (0, 90, 180, and 270), and are labeled accordingly in their filenames. [file 13068_2015_379_MOESM11_ESM.zip › gallery/C-1_P-36_180.png]

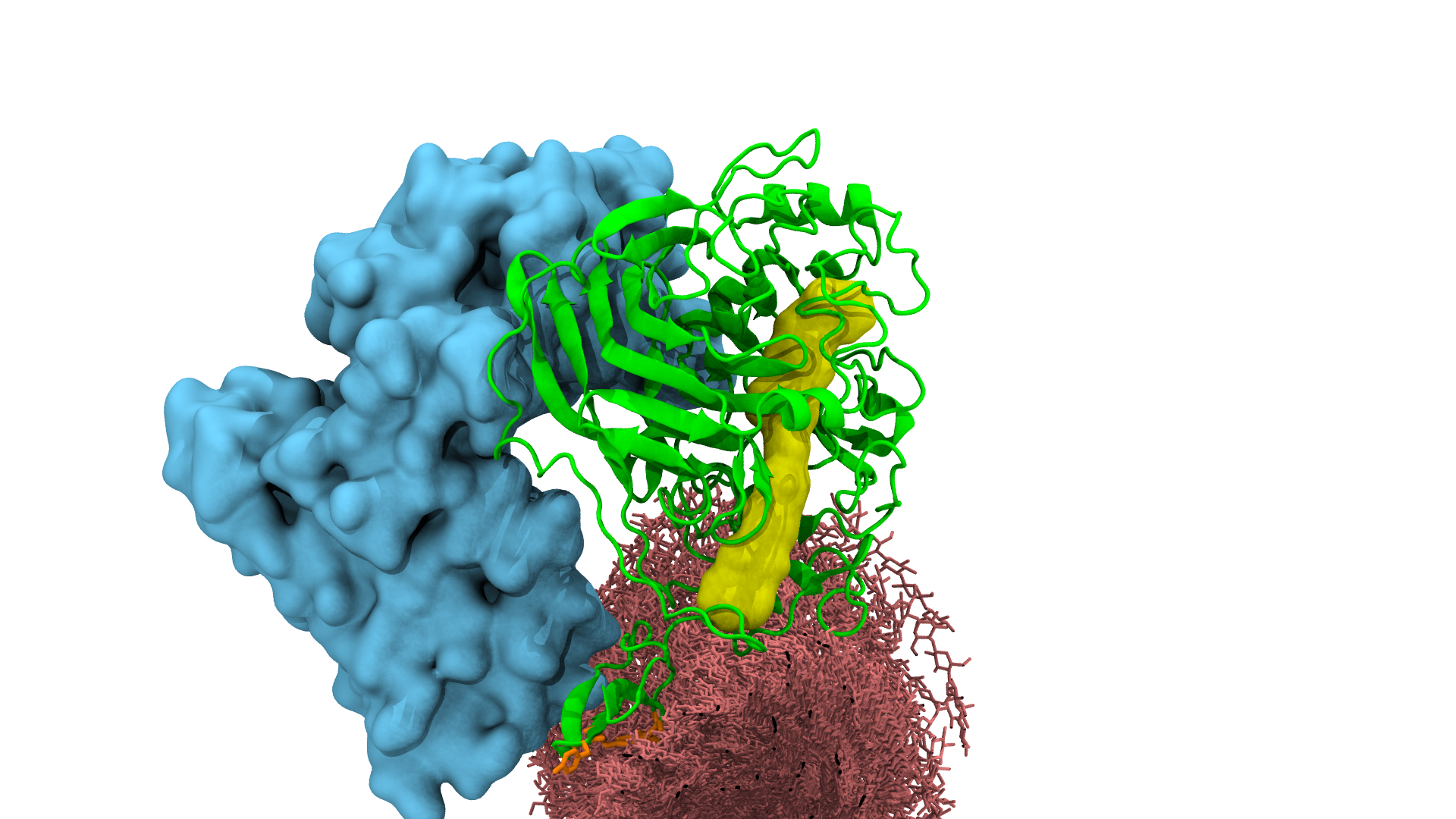

Supplement: Supplementary file 11 — 10.1186/s13068-015-0379-8 A zip archive containing a gallery of each of the cellulases that bound to cellulose in the context of their environment. Each image within the gallery is one snapshot taken from the end of the trajectory showing the relative position of each enzyme (green) that makes contact with the cellulose (red). Nearby lignins are shown in blue, and the substrate tunnel is a yellow surface to orient the viewer. The three tyrosine residues are shown in orange. Note that for each protein, there are 4 images, taken from different relative orientations to the cellulose fibril (0, 90, 180, and 270), and are labeled accordingly in their filenames. [file 13068_2015_379_MOESM11_ESM.zip › gallery/C-1_P-36_270.png]

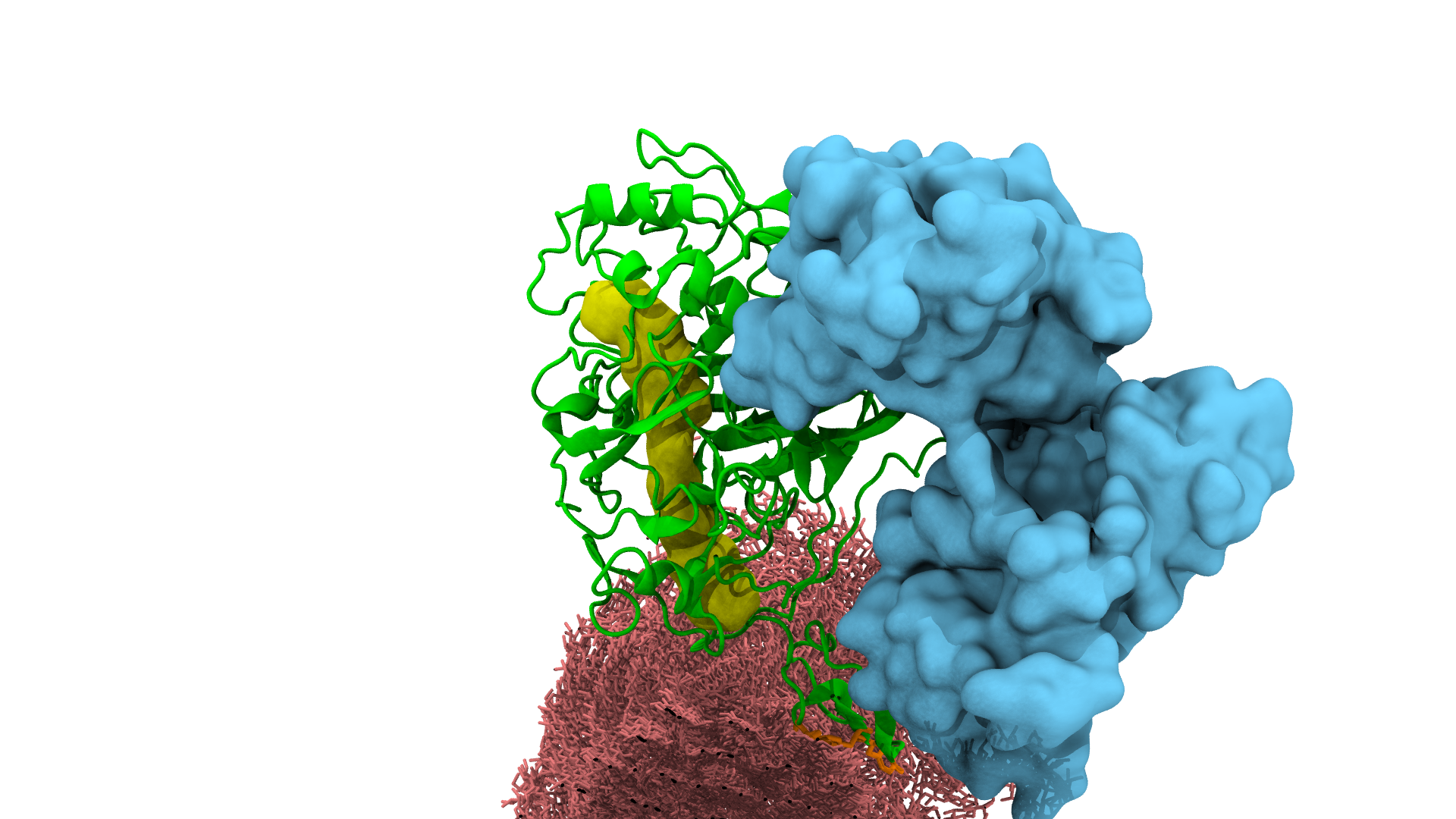

Supplement: Supplementary file 11 — 10.1186/s13068-015-0379-8 A zip archive containing a gallery of each of the cellulases that bound to cellulose in the context of their environment. Each image within the gallery is one snapshot taken from the end of the trajectory showing the relative position of each enzyme (green) that makes contact with the cellulose (red). Nearby lignins are shown in blue, and the substrate tunnel is a yellow surface to orient the viewer. The three tyrosine residues are shown in orange. Note that for each protein, there are 4 images, taken from different relative orientations to the cellulose fibril (0, 90, 180, and 270), and are labeled accordingly in their filenames. [file 13068_2015_379_MOESM11_ESM.zip › gallery/C-1_P-36_90.png]

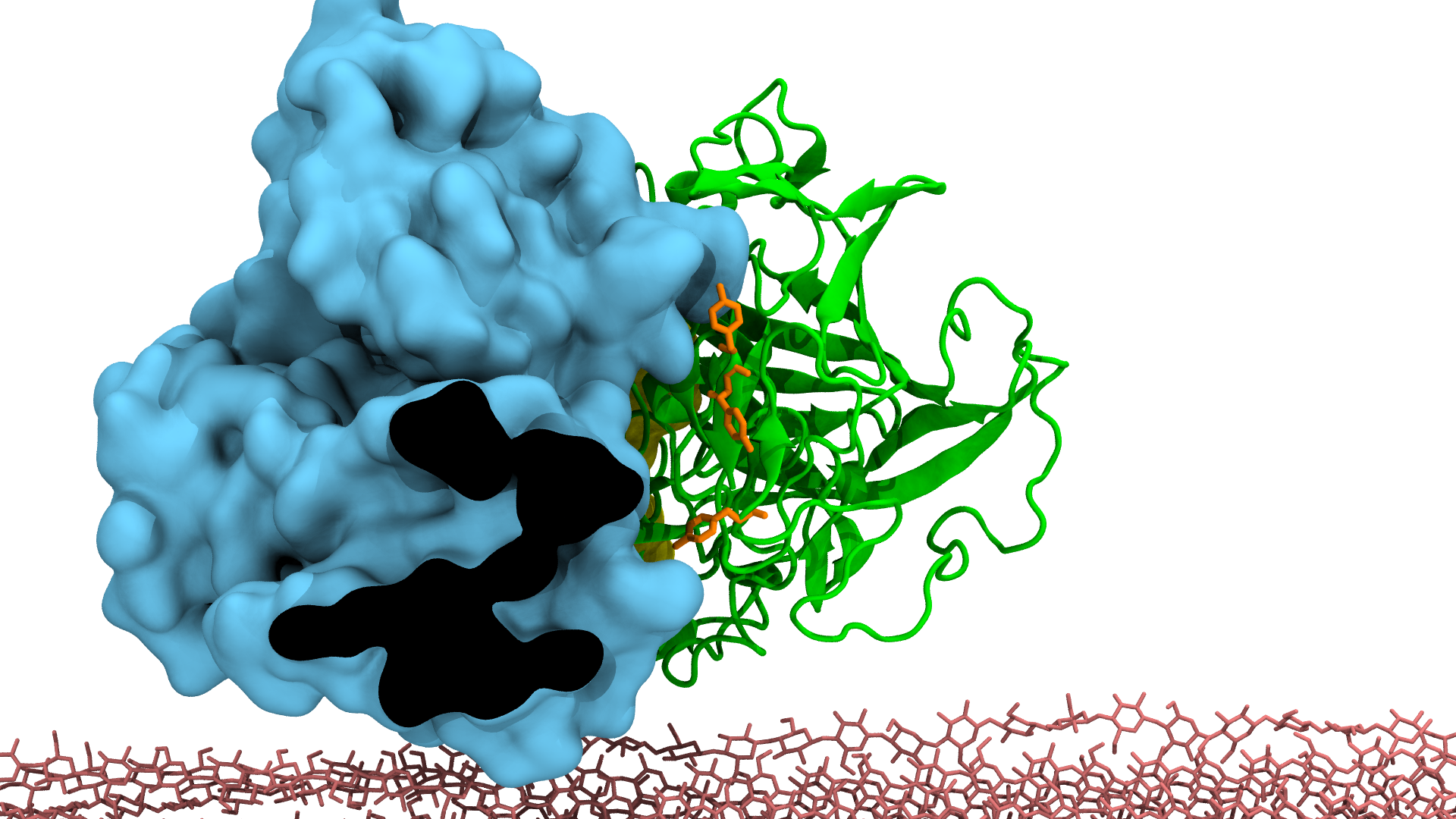

Supplement: Supplementary file 11 — 10.1186/s13068-015-0379-8 A zip archive containing a gallery of each of the cellulases that bound to cellulose in the context of their environment. Each image within the gallery is one snapshot taken from the end of the trajectory showing the relative position of each enzyme (green) that makes contact with the cellulose (red). Nearby lignins are shown in blue, and the substrate tunnel is a yellow surface to orient the viewer. The three tyrosine residues are shown in orange. Note that for each protein, there are 4 images, taken from different relative orientations to the cellulose fibril (0, 90, 180, and 270), and are labeled accordingly in their filenames. [file 13068_2015_379_MOESM11_ESM.zip › gallery/C-1_P-39_0.png]

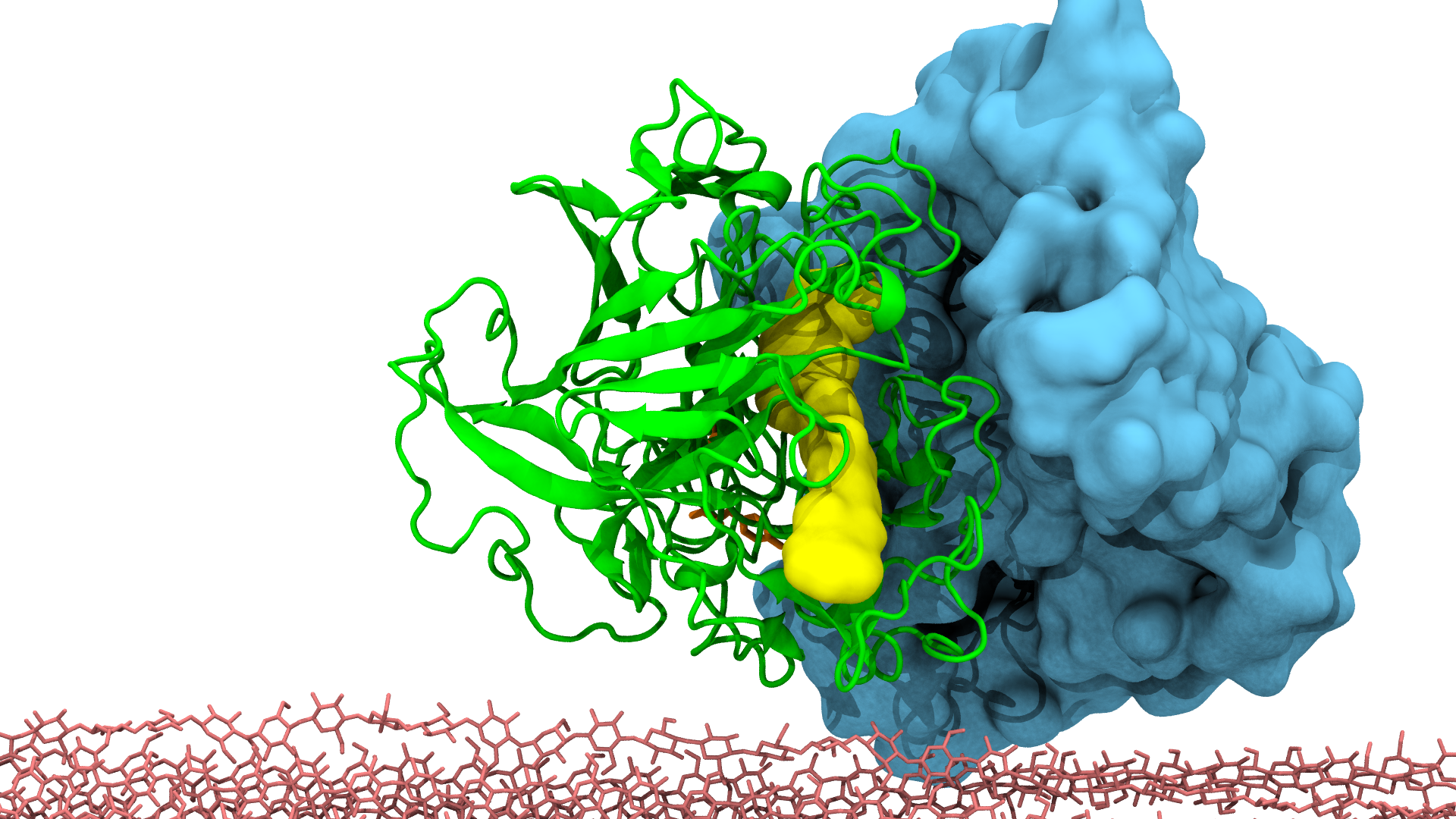

Supplement: Supplementary file 11 — 10.1186/s13068-015-0379-8 A zip archive containing a gallery of each of the cellulases that bound to cellulose in the context of their environment. Each image within the gallery is one snapshot taken from the end of the trajectory showing the relative position of each enzyme (green) that makes contact with the cellulose (red). Nearby lignins are shown in blue, and the substrate tunnel is a yellow surface to orient the viewer. The three tyrosine residues are shown in orange. Note that for each protein, there are 4 images, taken from different relative orientations to the cellulose fibril (0, 90, 180, and 270), and are labeled accordingly in their filenames. [file 13068_2015_379_MOESM11_ESM.zip › gallery/C-1_P-39_180.png]

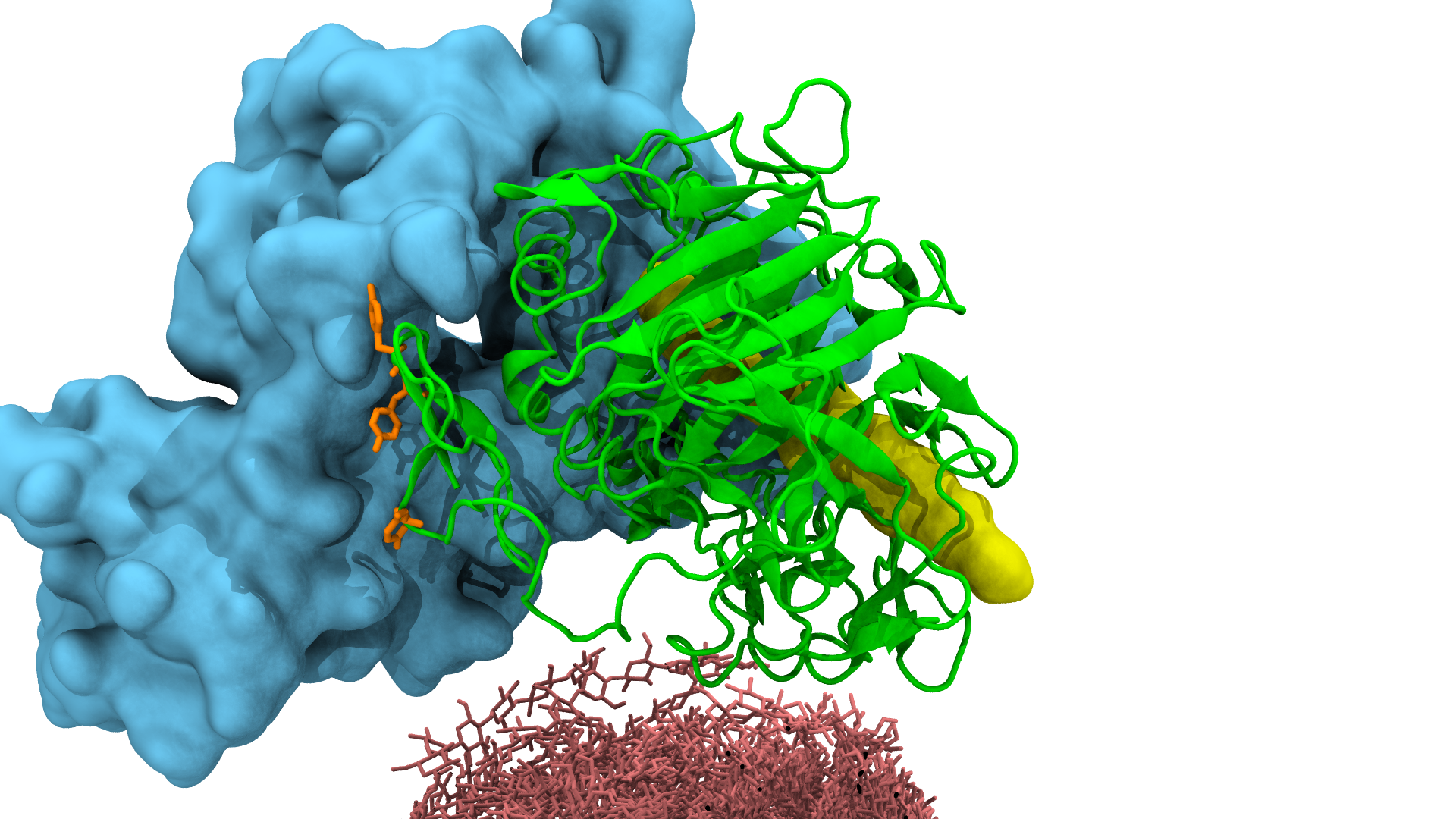

Supplement: Supplementary file 11 — 10.1186/s13068-015-0379-8 A zip archive containing a gallery of each of the cellulases that bound to cellulose in the context of their environment. Each image within the gallery is one snapshot taken from the end of the trajectory showing the relative position of each enzyme (green) that makes contact with the cellulose (red). Nearby lignins are shown in blue, and the substrate tunnel is a yellow surface to orient the viewer. The three tyrosine residues are shown in orange. Note that for each protein, there are 4 images, taken from different relative orientations to the cellulose fibril (0, 90, 180, and 270), and are labeled accordingly in their filenames. [file 13068_2015_379_MOESM11_ESM.zip › gallery/C-1_P-39_270.png]

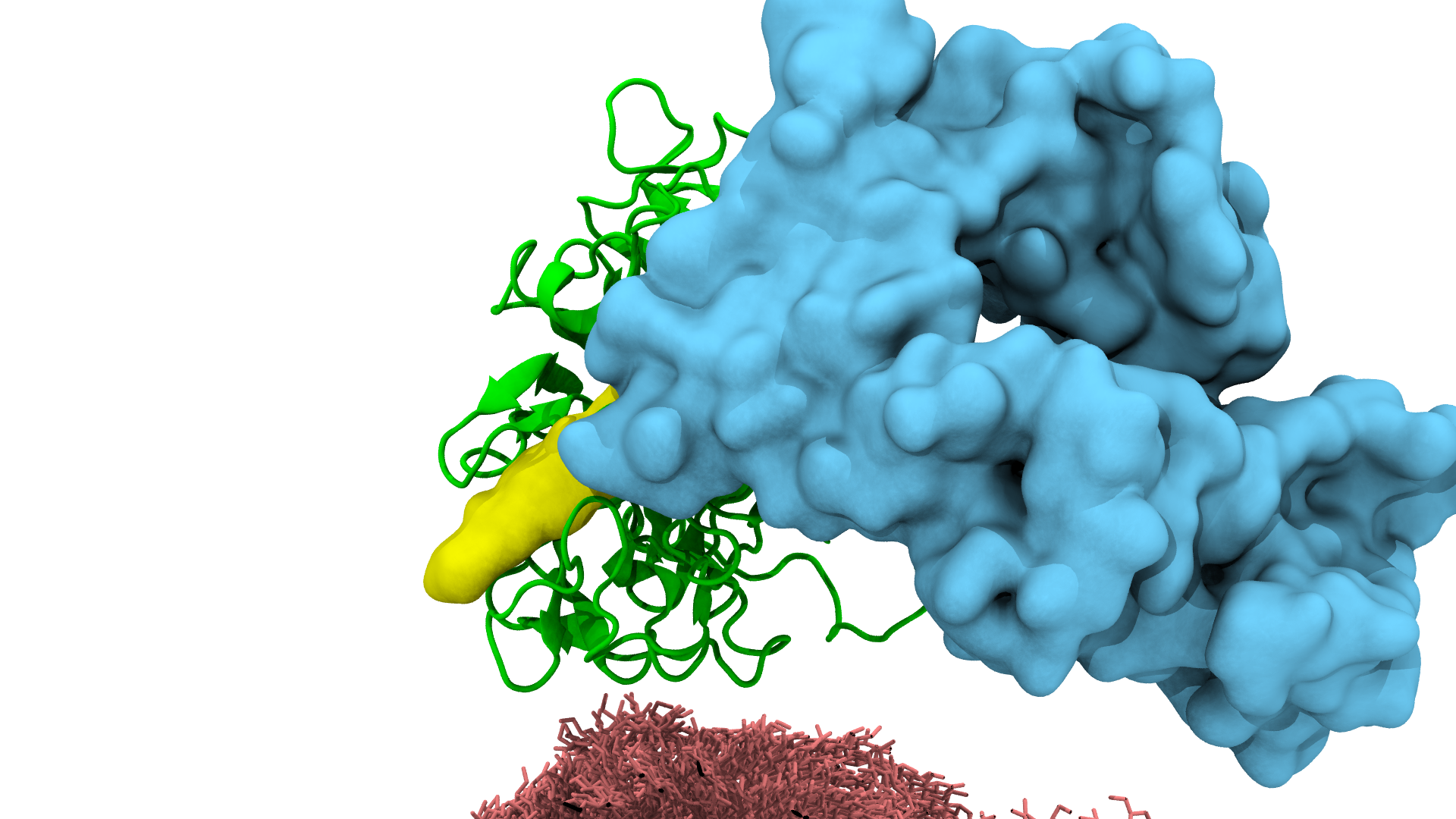

Supplement: Supplementary file 11 — 10.1186/s13068-015-0379-8 A zip archive containing a gallery of each of the cellulases that bound to cellulose in the context of their environment. Each image within the gallery is one snapshot taken from the end of the trajectory showing the relative position of each enzyme (green) that makes contact with the cellulose (red). Nearby lignins are shown in blue, and the substrate tunnel is a yellow surface to orient the viewer. The three tyrosine residues are shown in orange. Note that for each protein, there are 4 images, taken from different relative orientations to the cellulose fibril (0, 90, 180, and 270), and are labeled accordingly in their filenames. [file 13068_2015_379_MOESM11_ESM.zip › gallery/C-1_P-39_90.png]

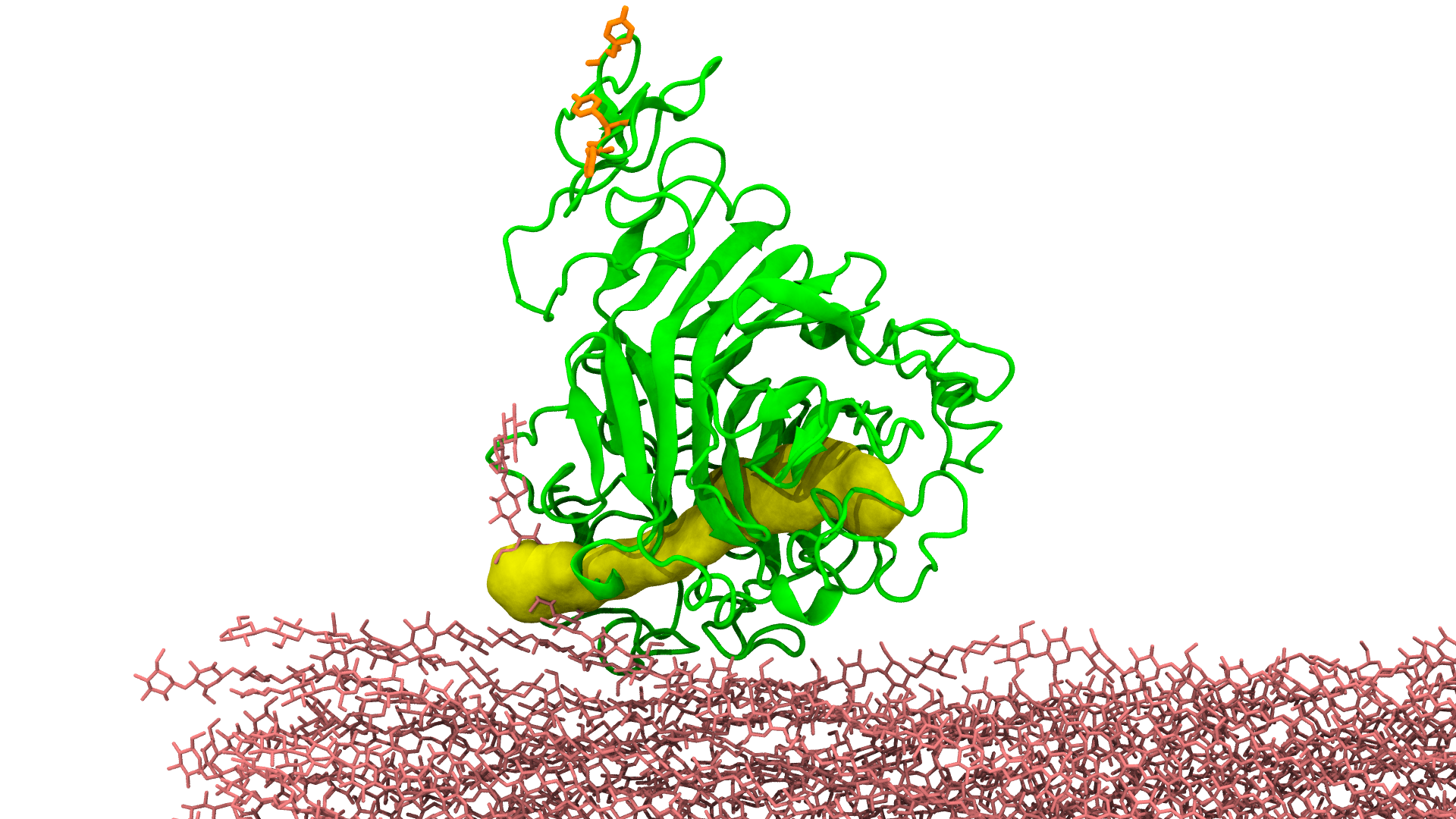

Supplement: Supplementary file 11 — 10.1186/s13068-015-0379-8 A zip archive containing a gallery of each of the cellulases that bound to cellulose in the context of their environment. Each image within the gallery is one snapshot taken from the end of the trajectory showing the relative position of each enzyme (green) that makes contact with the cellulose (red). Nearby lignins are shown in blue, and the substrate tunnel is a yellow surface to orient the viewer. The three tyrosine residues are shown in orange. Note that for each protein, there are 4 images, taken from different relative orientations to the cellulose fibril (0, 90, 180, and 270), and are labeled accordingly in their filenames. [file 13068_2015_379_MOESM11_ESM.zip › gallery/C-1_P-49_0.png]

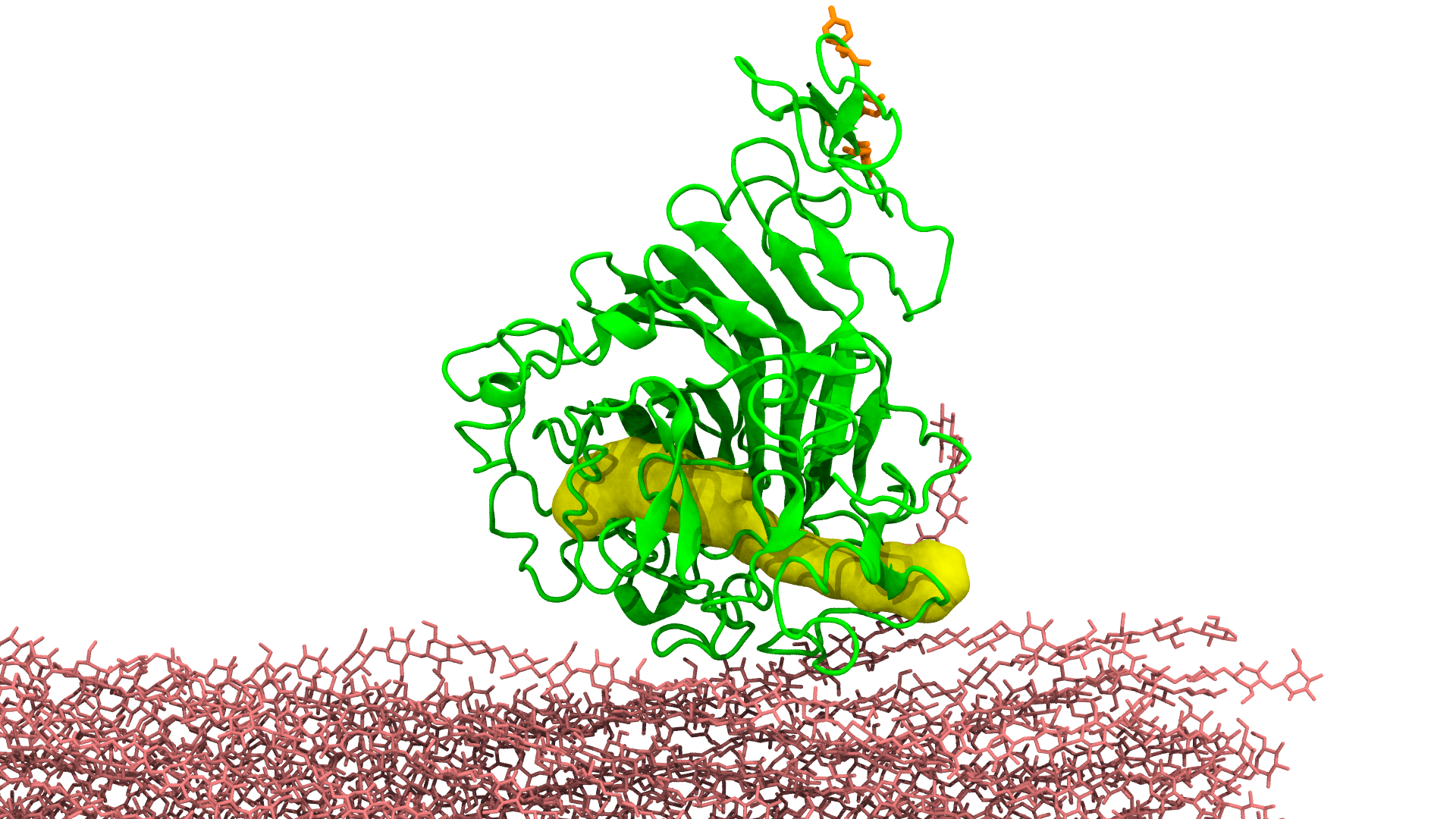

Supplement: Supplementary file 11 — 10.1186/s13068-015-0379-8 A zip archive containing a gallery of each of the cellulases that bound to cellulose in the context of their environment. Each image within the gallery is one snapshot taken from the end of the trajectory showing the relative position of each enzyme (green) that makes contact with the cellulose (red). Nearby lignins are shown in blue, and the substrate tunnel is a yellow surface to orient the viewer. The three tyrosine residues are shown in orange. Note that for each protein, there are 4 images, taken from different relative orientations to the cellulose fibril (0, 90, 180, and 270), and are labeled accordingly in their filenames. [file 13068_2015_379_MOESM11_ESM.zip › gallery/C-1_P-49_180.png]

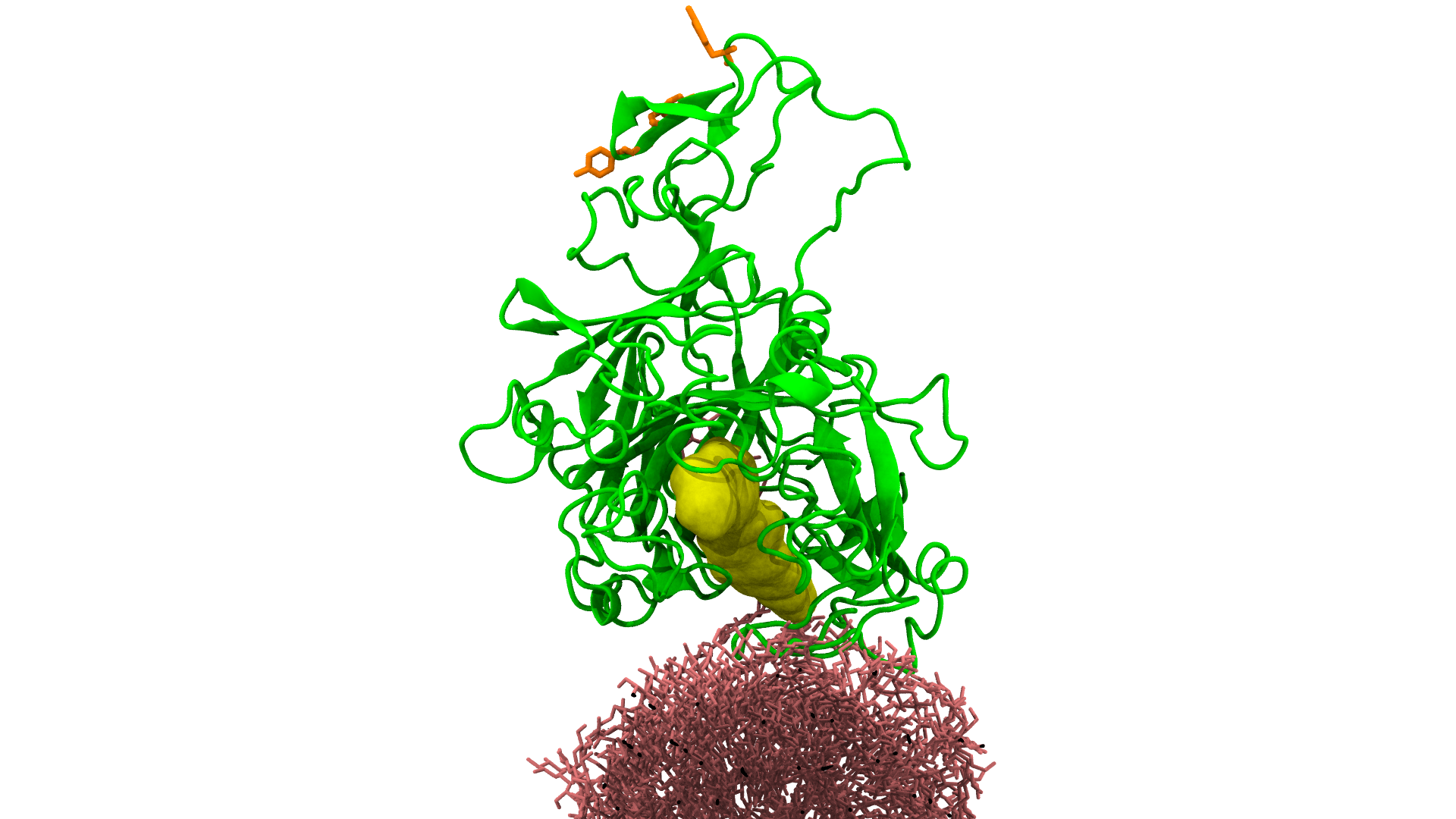

Supplement: Supplementary file 11 — 10.1186/s13068-015-0379-8 A zip archive containing a gallery of each of the cellulases that bound to cellulose in the context of their environment. Each image within the gallery is one snapshot taken from the end of the trajectory showing the relative position of each enzyme (green) that makes contact with the cellulose (red). Nearby lignins are shown in blue, and the substrate tunnel is a yellow surface to orient the viewer. The three tyrosine residues are shown in orange. Note that for each protein, there are 4 images, taken from different relative orientations to the cellulose fibril (0, 90, 180, and 270), and are labeled accordingly in their filenames. [file 13068_2015_379_MOESM11_ESM.zip › gallery/C-1_P-49_270.png]

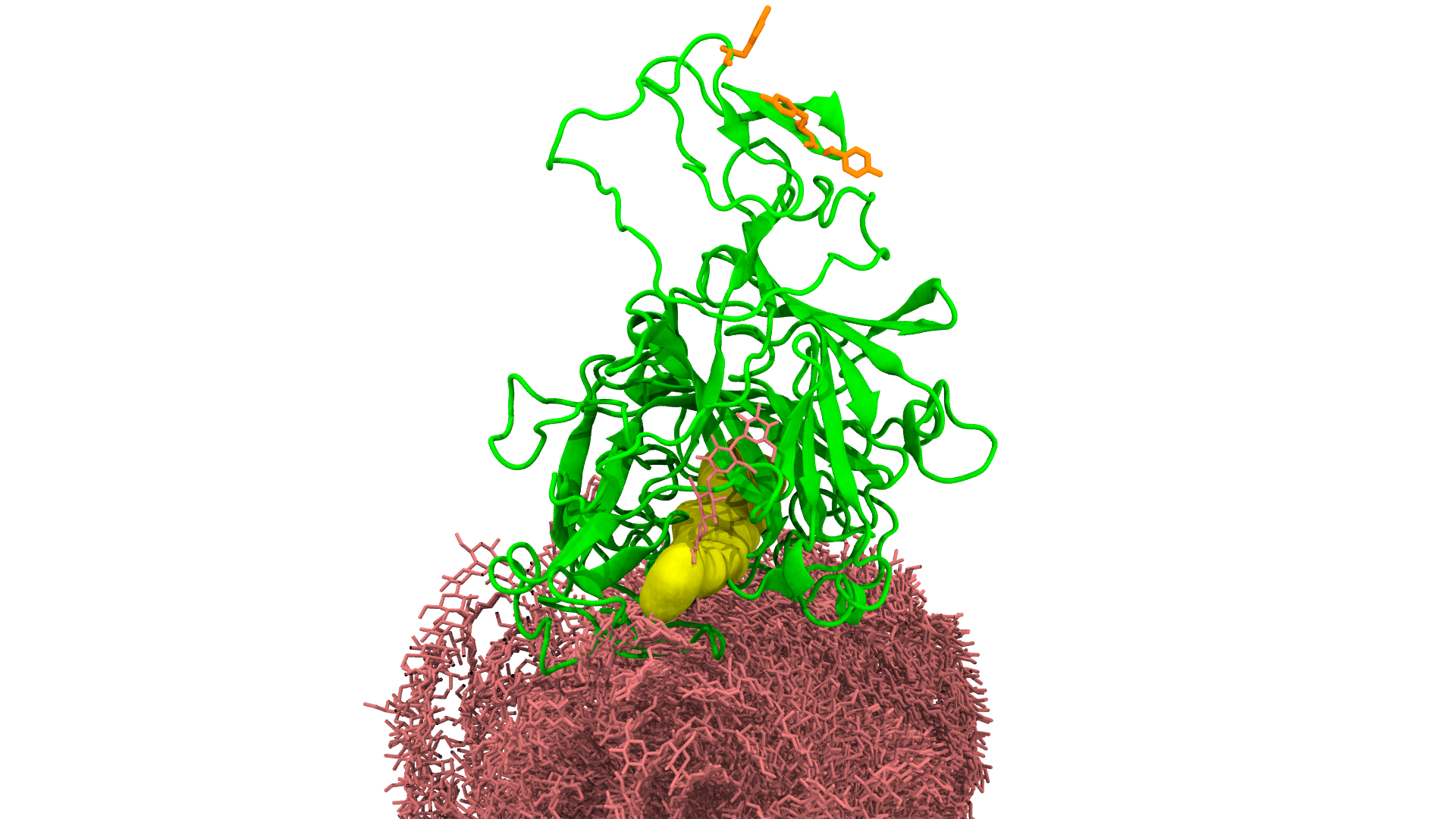

Supplement: Supplementary file 11 — 10.1186/s13068-015-0379-8 A zip archive containing a gallery of each of the cellulases that bound to cellulose in the context of their environment. Each image within the gallery is one snapshot taken from the end of the trajectory showing the relative position of each enzyme (green) that makes contact with the cellulose (red). Nearby lignins are shown in blue, and the substrate tunnel is a yellow surface to orient the viewer. The three tyrosine residues are shown in orange. Note that for each protein, there are 4 images, taken from different relative orientations to the cellulose fibril (0, 90, 180, and 270), and are labeled accordingly in their filenames. [file 13068_2015_379_MOESM11_ESM.zip › gallery/C-1_P-49_90.png]

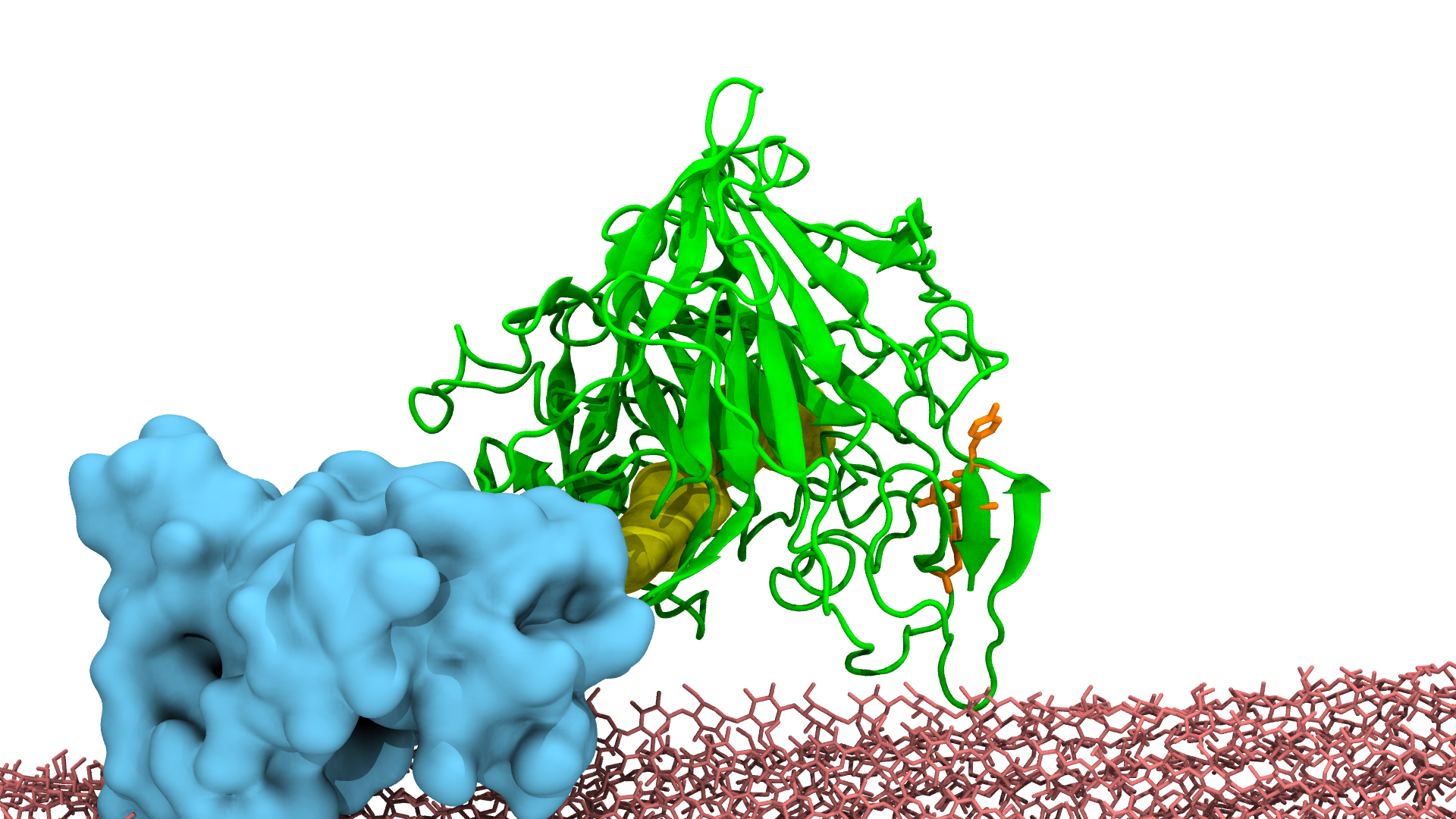

Supplement: Supplementary file 11 — 10.1186/s13068-015-0379-8 A zip archive containing a gallery of each of the cellulases that bound to cellulose in the context of their environment. Each image within the gallery is one snapshot taken from the end of the trajectory showing the relative position of each enzyme (green) that makes contact with the cellulose (red). Nearby lignins are shown in blue, and the substrate tunnel is a yellow surface to orient the viewer. The three tyrosine residues are shown in orange. Note that for each protein, there are 4 images, taken from different relative orientations to the cellulose fibril (0, 90, 180, and 270), and are labeled accordingly in their filenames. [file 13068_2015_379_MOESM11_ESM.zip › gallery/C-1_P-53_0.png]

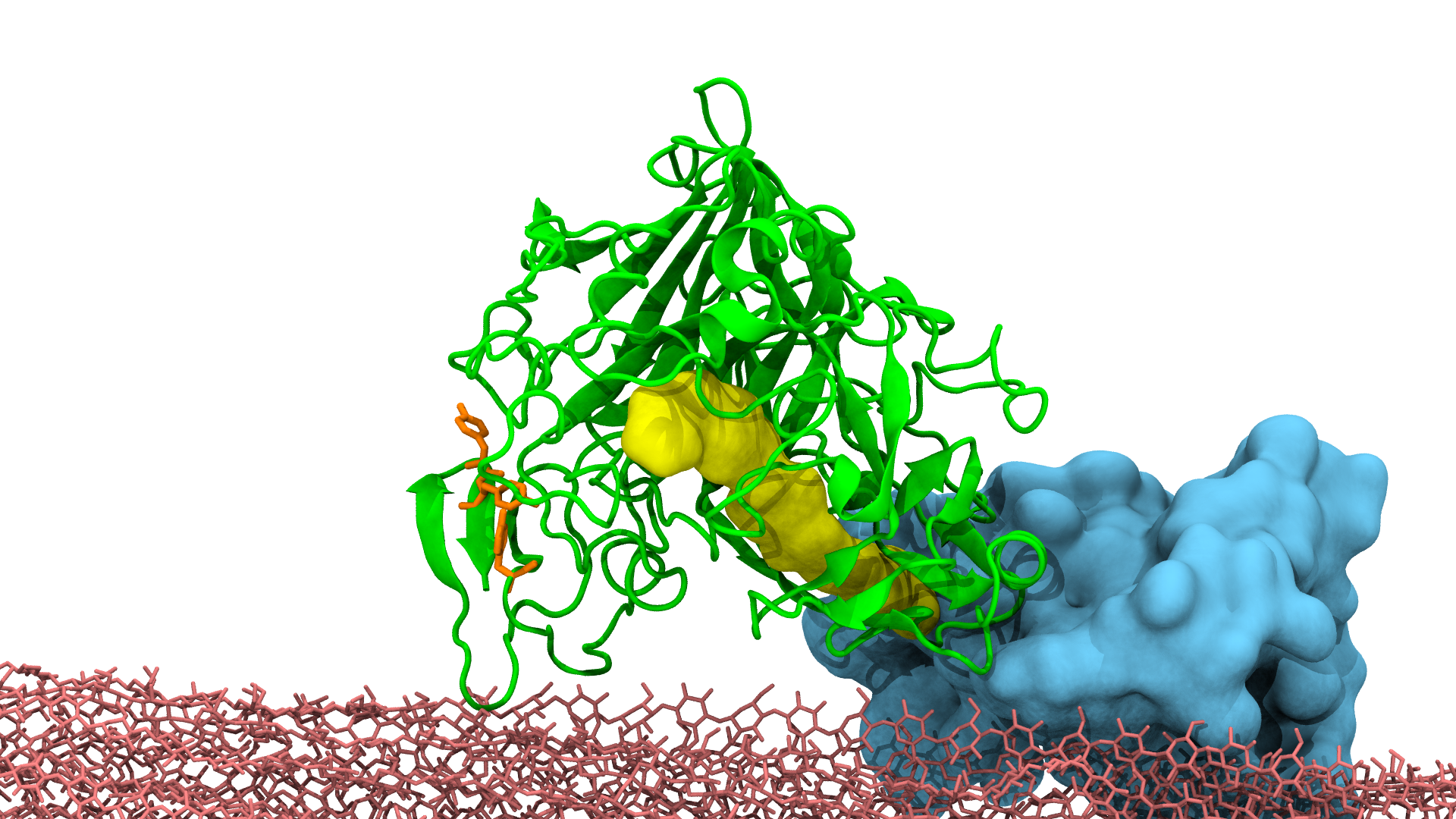

Supplement: Supplementary file 11 — 10.1186/s13068-015-0379-8 A zip archive containing a gallery of each of the cellulases that bound to cellulose in the context of their environment. Each image within the gallery is one snapshot taken from the end of the trajectory showing the relative position of each enzyme (green) that makes contact with the cellulose (red). Nearby lignins are shown in blue, and the substrate tunnel is a yellow surface to orient the viewer. The three tyrosine residues are shown in orange. Note that for each protein, there are 4 images, taken from different relative orientations to the cellulose fibril (0, 90, 180, and 270), and are labeled accordingly in their filenames. [file 13068_2015_379_MOESM11_ESM.zip › gallery/C-1_P-53_180.png]

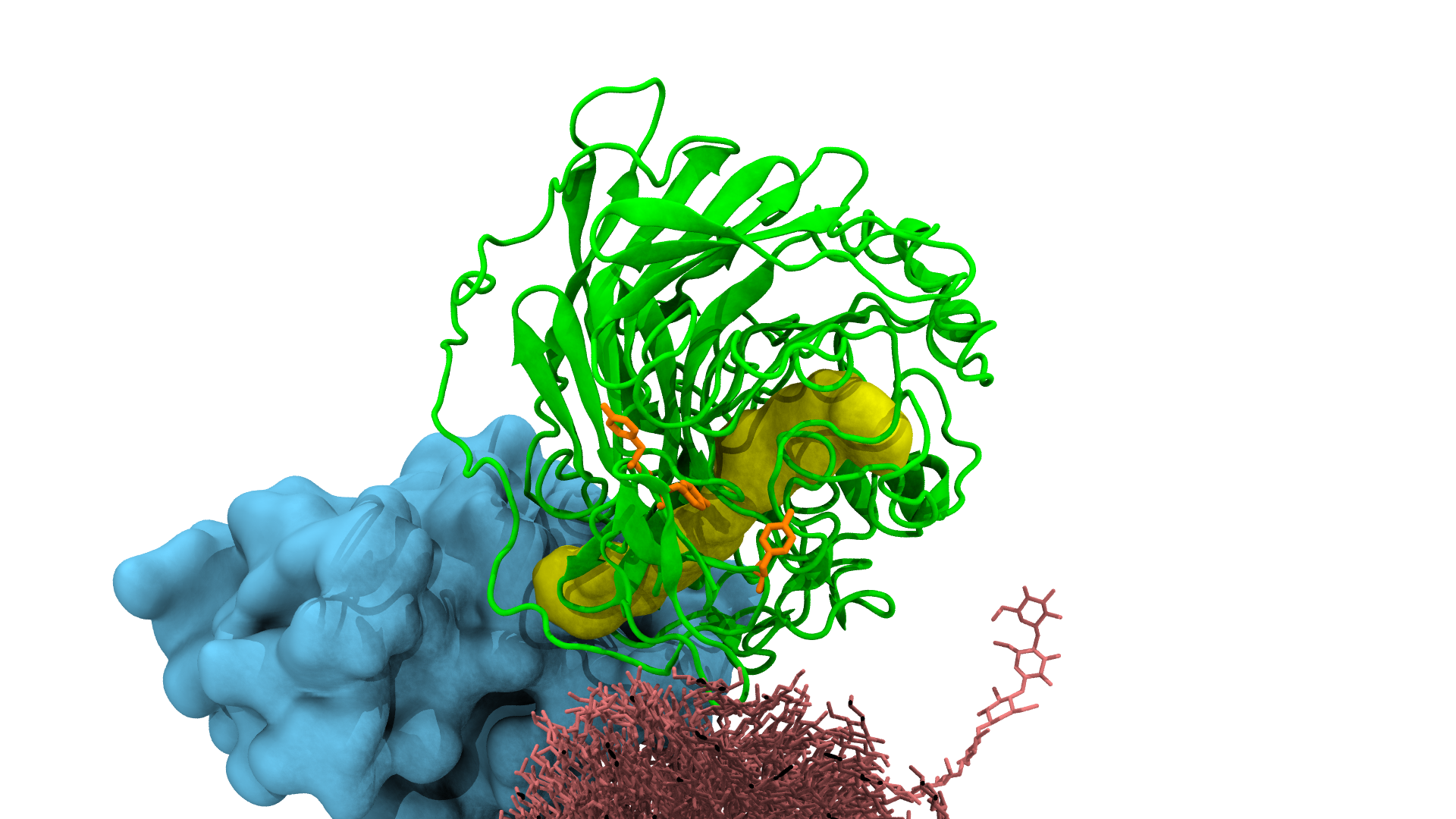

Supplement: Supplementary file 11 — 10.1186/s13068-015-0379-8 A zip archive containing a gallery of each of the cellulases that bound to cellulose in the context of their environment. Each image within the gallery is one snapshot taken from the end of the trajectory showing the relative position of each enzyme (green) that makes contact with the cellulose (red). Nearby lignins are shown in blue, and the substrate tunnel is a yellow surface to orient the viewer. The three tyrosine residues are shown in orange. Note that for each protein, there are 4 images, taken from different relative orientations to the cellulose fibril (0, 90, 180, and 270), and are labeled accordingly in their filenames. [file 13068_2015_379_MOESM11_ESM.zip › gallery/C-1_P-53_270.png]

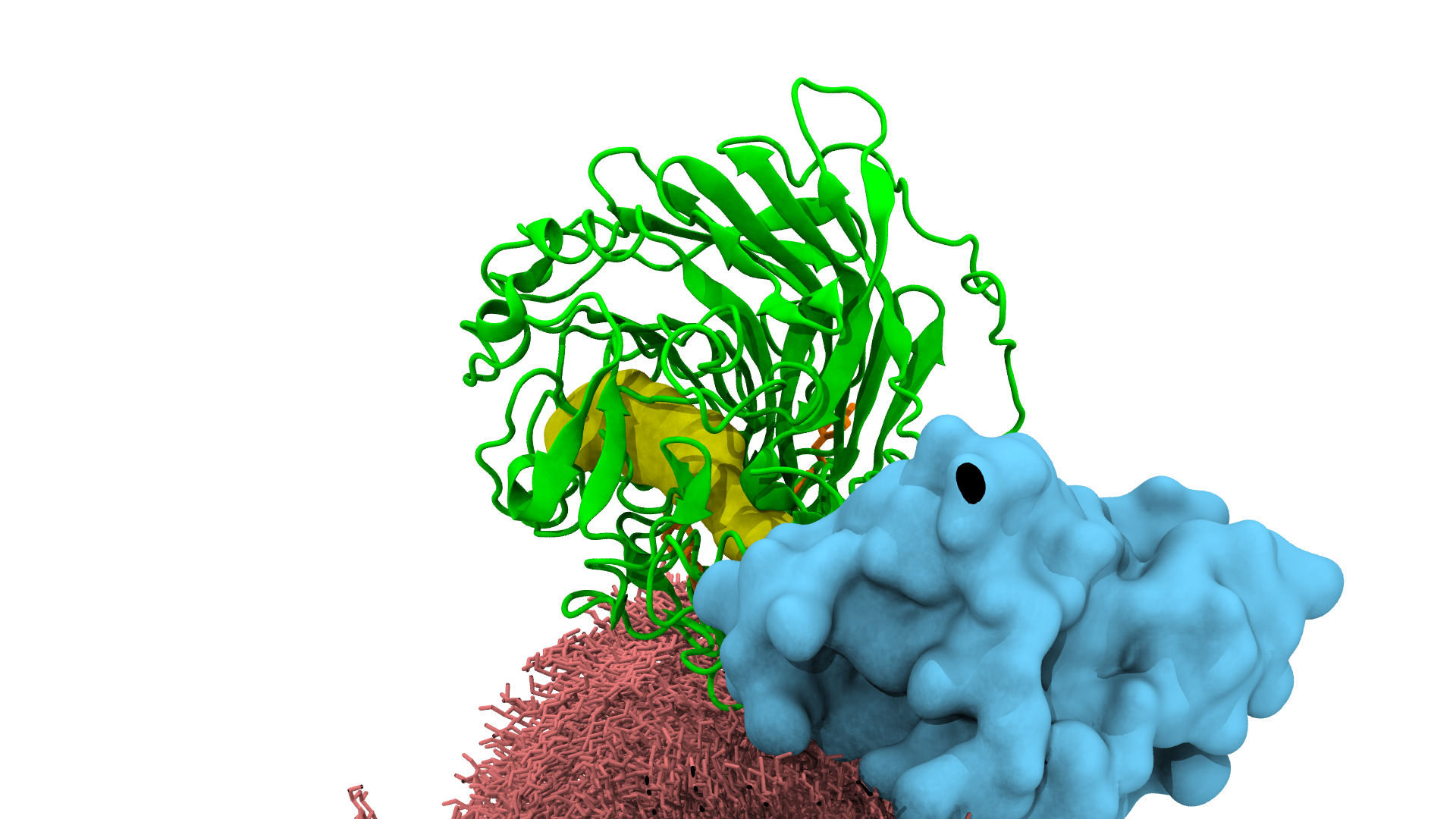

Supplement: Supplementary file 11 — 10.1186/s13068-015-0379-8 A zip archive containing a gallery of each of the cellulases that bound to cellulose in the context of their environment. Each image within the gallery is one snapshot taken from the end of the trajectory showing the relative position of each enzyme (green) that makes contact with the cellulose (red). Nearby lignins are shown in blue, and the substrate tunnel is a yellow surface to orient the viewer. The three tyrosine residues are shown in orange. Note that for each protein, there are 4 images, taken from different relative orientations to the cellulose fibril (0, 90, 180, and 270), and are labeled accordingly in their filenames. [file 13068_2015_379_MOESM11_ESM.zip › gallery/C-1_P-53_90.png]

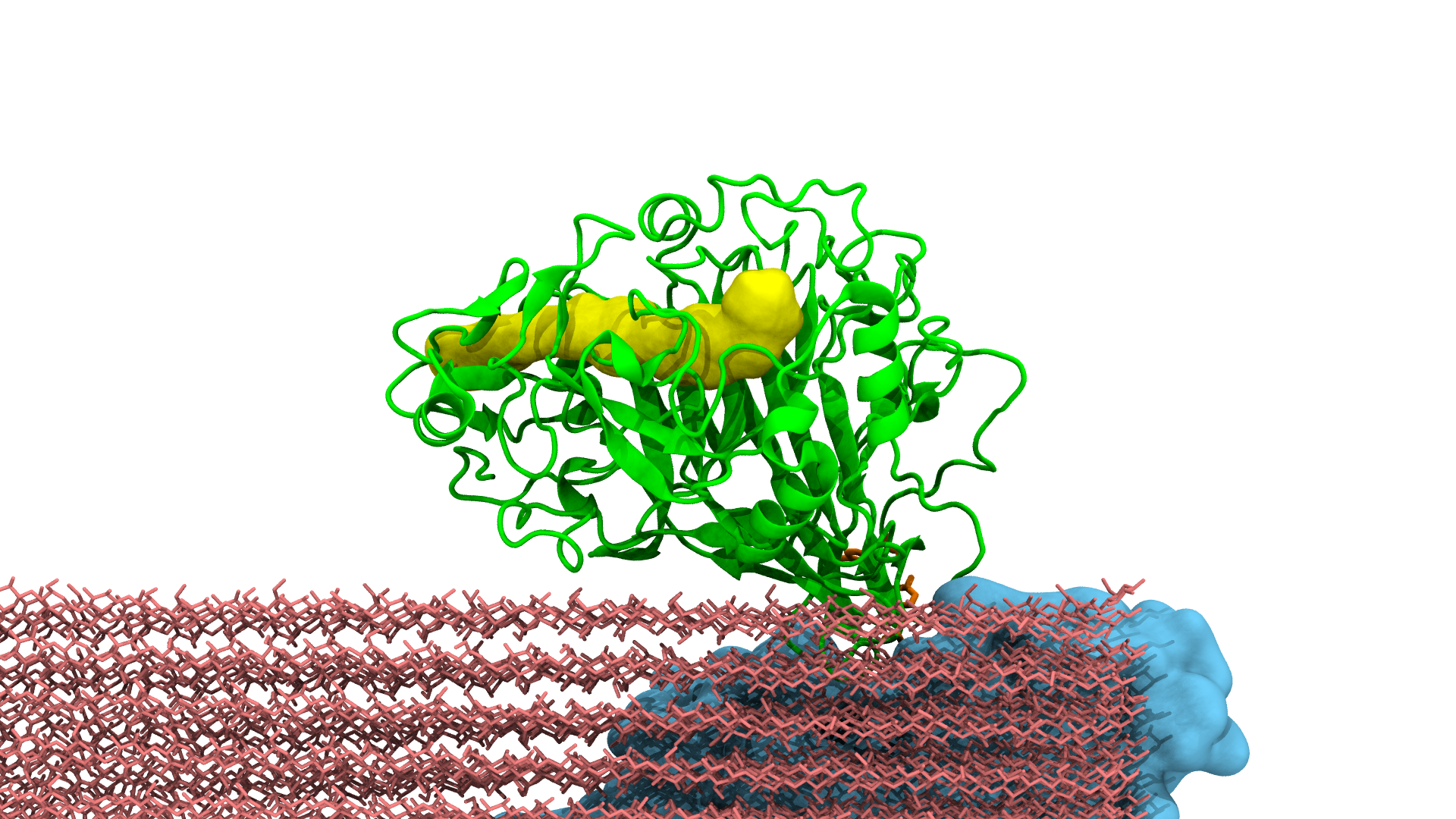

Supplement: Supplementary file 11 — 10.1186/s13068-015-0379-8 A zip archive containing a gallery of each of the cellulases that bound to cellulose in the context of their environment. Each image within the gallery is one snapshot taken from the end of the trajectory showing the relative position of each enzyme (green) that makes contact with the cellulose (red). Nearby lignins are shown in blue, and the substrate tunnel is a yellow surface to orient the viewer. The three tyrosine residues are shown in orange. Note that for each protein, there are 4 images, taken from different relative orientations to the cellulose fibril (0, 90, 180, and 270), and are labeled accordingly in their filenames. [file 13068_2015_379_MOESM11_ESM.zip › gallery/C-2_P-48_0.png]

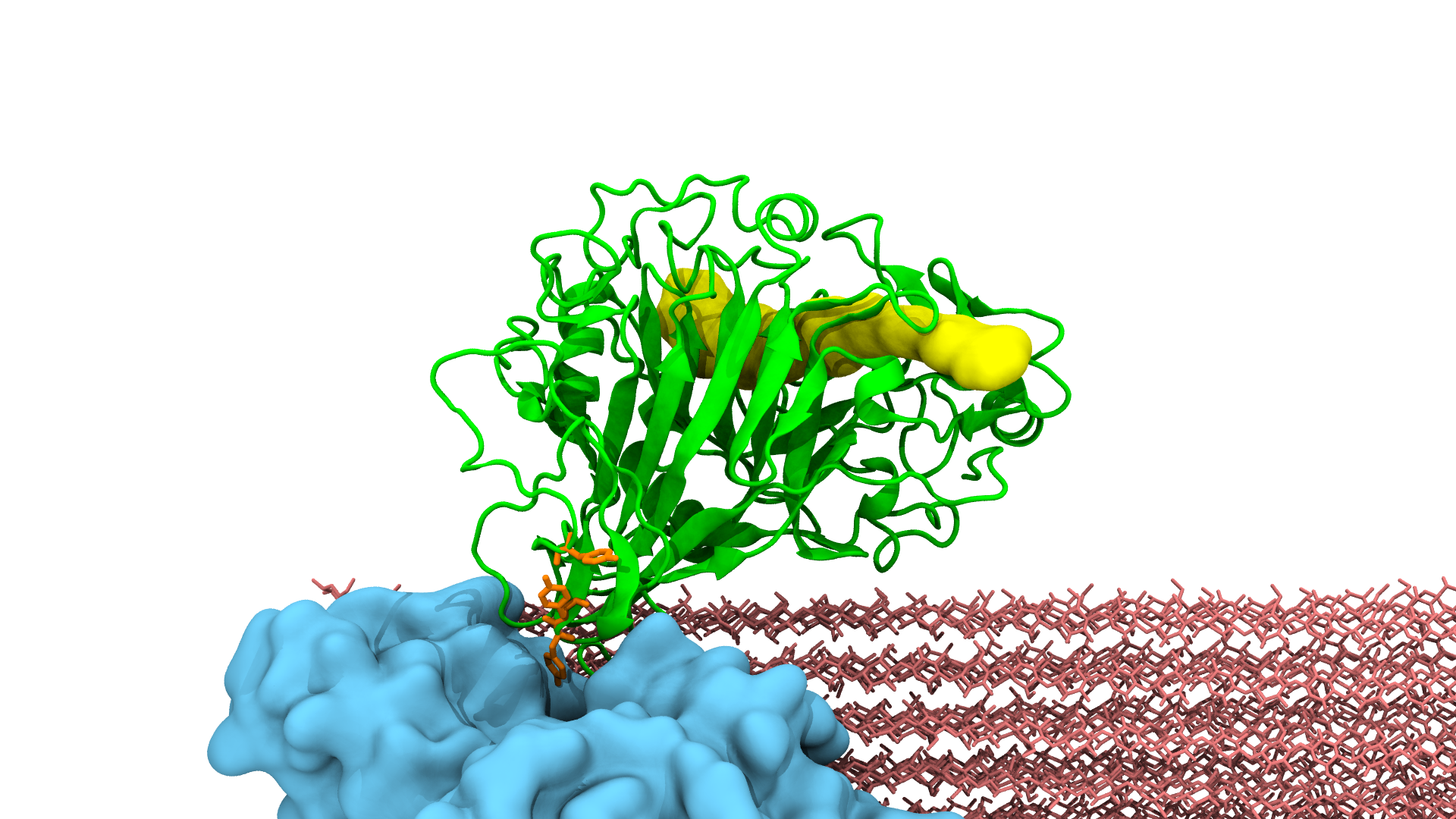

Supplement: Supplementary file 11 — 10.1186/s13068-015-0379-8 A zip archive containing a gallery of each of the cellulases that bound to cellulose in the context of their environment. Each image within the gallery is one snapshot taken from the end of the trajectory showing the relative position of each enzyme (green) that makes contact with the cellulose (red). Nearby lignins are shown in blue, and the substrate tunnel is a yellow surface to orient the viewer. The three tyrosine residues are shown in orange. Note that for each protein, there are 4 images, taken from different relative orientations to the cellulose fibril (0, 90, 180, and 270), and are labeled accordingly in their filenames. [file 13068_2015_379_MOESM11_ESM.zip › gallery/C-2_P-48_180.png]

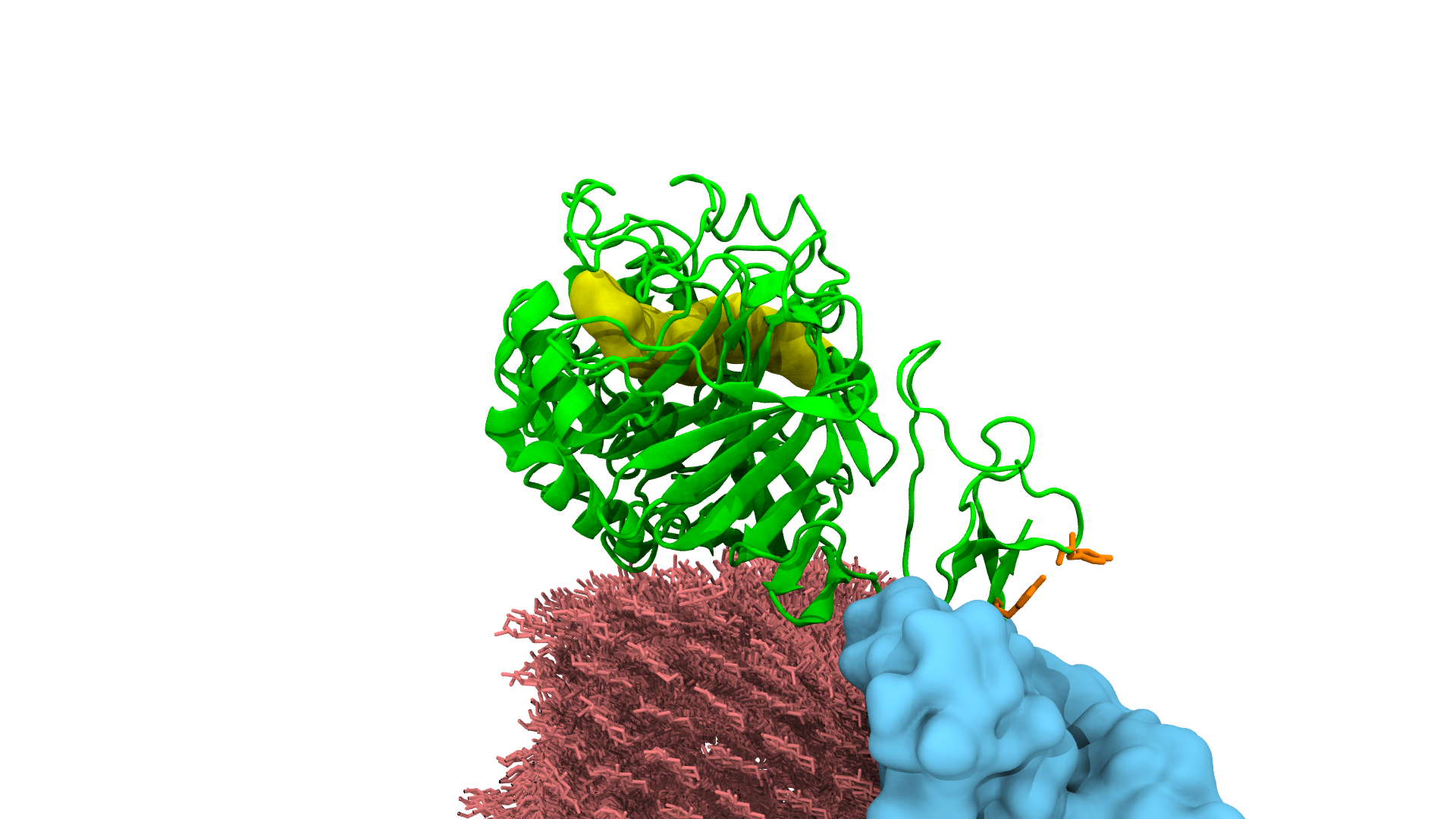

Supplement: Supplementary file 11 — 10.1186/s13068-015-0379-8 A zip archive containing a gallery of each of the cellulases that bound to cellulose in the context of their environment. Each image within the gallery is one snapshot taken from the end of the trajectory showing the relative position of each enzyme (green) that makes contact with the cellulose (red). Nearby lignins are shown in blue, and the substrate tunnel is a yellow surface to orient the viewer. The three tyrosine residues are shown in orange. Note that for each protein, there are 4 images, taken from different relative orientations to the cellulose fibril (0, 90, 180, and 270), and are labeled accordingly in their filenames. [file 13068_2015_379_MOESM11_ESM.zip › gallery/C-2_P-48_270.png]

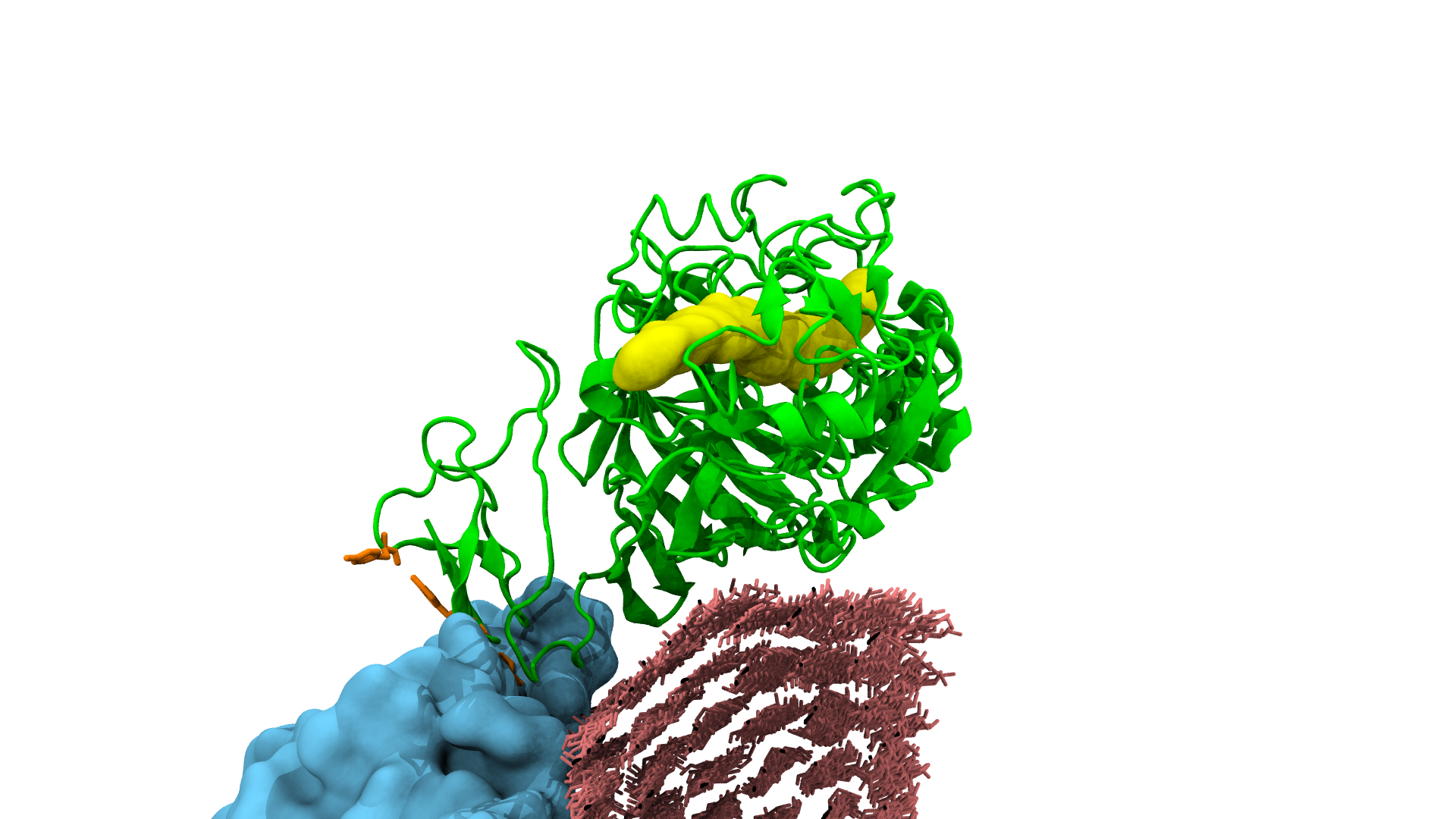

Supplement: Supplementary file 11 — 10.1186/s13068-015-0379-8 A zip archive containing a gallery of each of the cellulases that bound to cellulose in the context of their environment. Each image within the gallery is one snapshot taken from the end of the trajectory showing the relative position of each enzyme (green) that makes contact with the cellulose (red). Nearby lignins are shown in blue, and the substrate tunnel is a yellow surface to orient the viewer. The three tyrosine residues are shown in orange. Note that for each protein, there are 4 images, taken from different relative orientations to the cellulose fibril (0, 90, 180, and 270), and are labeled accordingly in their filenames. [file 13068_2015_379_MOESM11_ESM.zip › gallery/C-2_P-48_90.png]

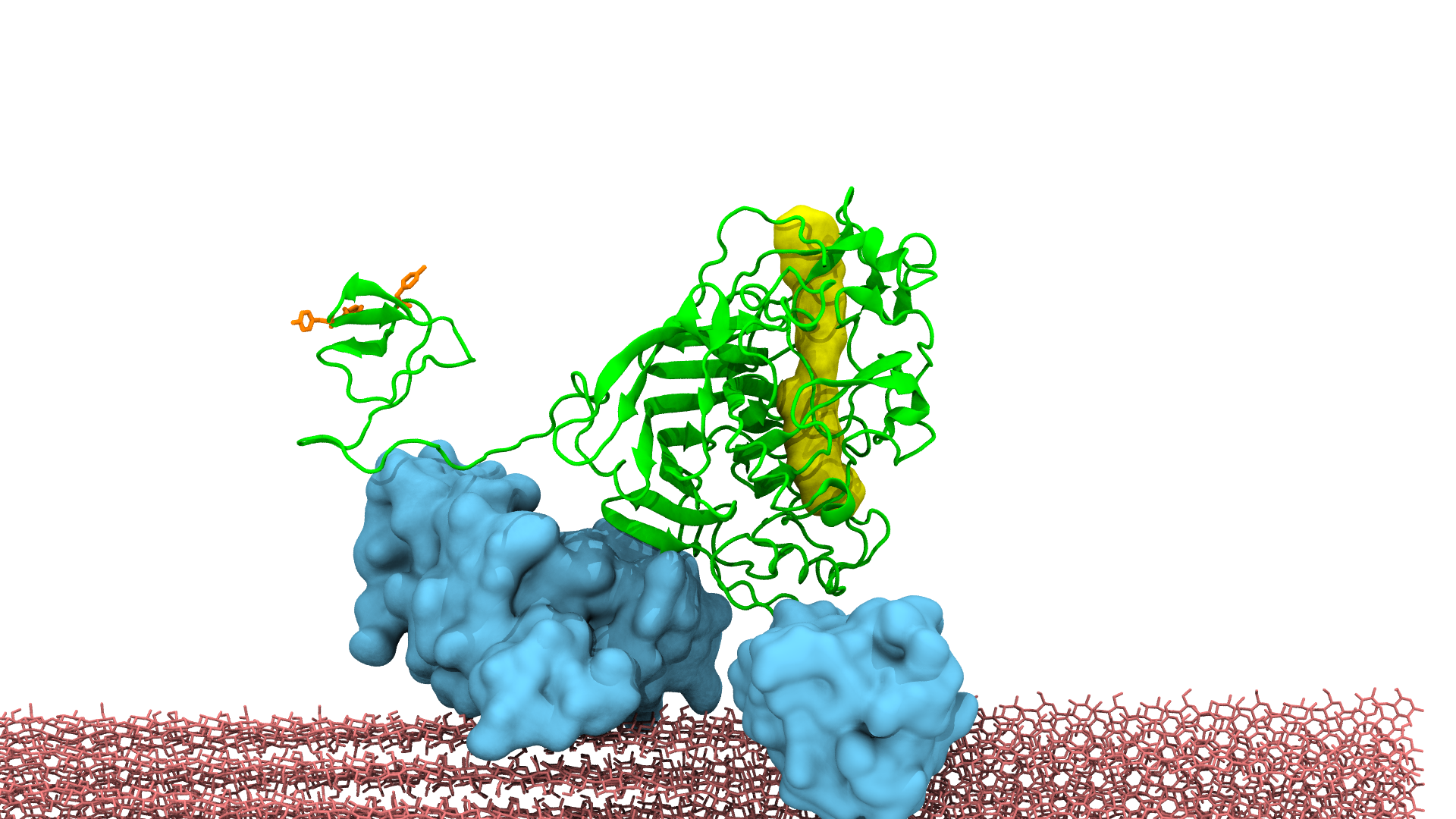

Supplement: Supplementary file 11 — 10.1186/s13068-015-0379-8 A zip archive containing a gallery of each of the cellulases that bound to cellulose in the context of their environment. Each image within the gallery is one snapshot taken from the end of the trajectory showing the relative position of each enzyme (green) that makes contact with the cellulose (red). Nearby lignins are shown in blue, and the substrate tunnel is a yellow surface to orient the viewer. The three tyrosine residues are shown in orange. Note that for each protein, there are 4 images, taken from different relative orientations to the cellulose fibril (0, 90, 180, and 270), and are labeled accordingly in their filenames. [file 13068_2015_379_MOESM11_ESM.zip › gallery/C-3_P-16_0.png]

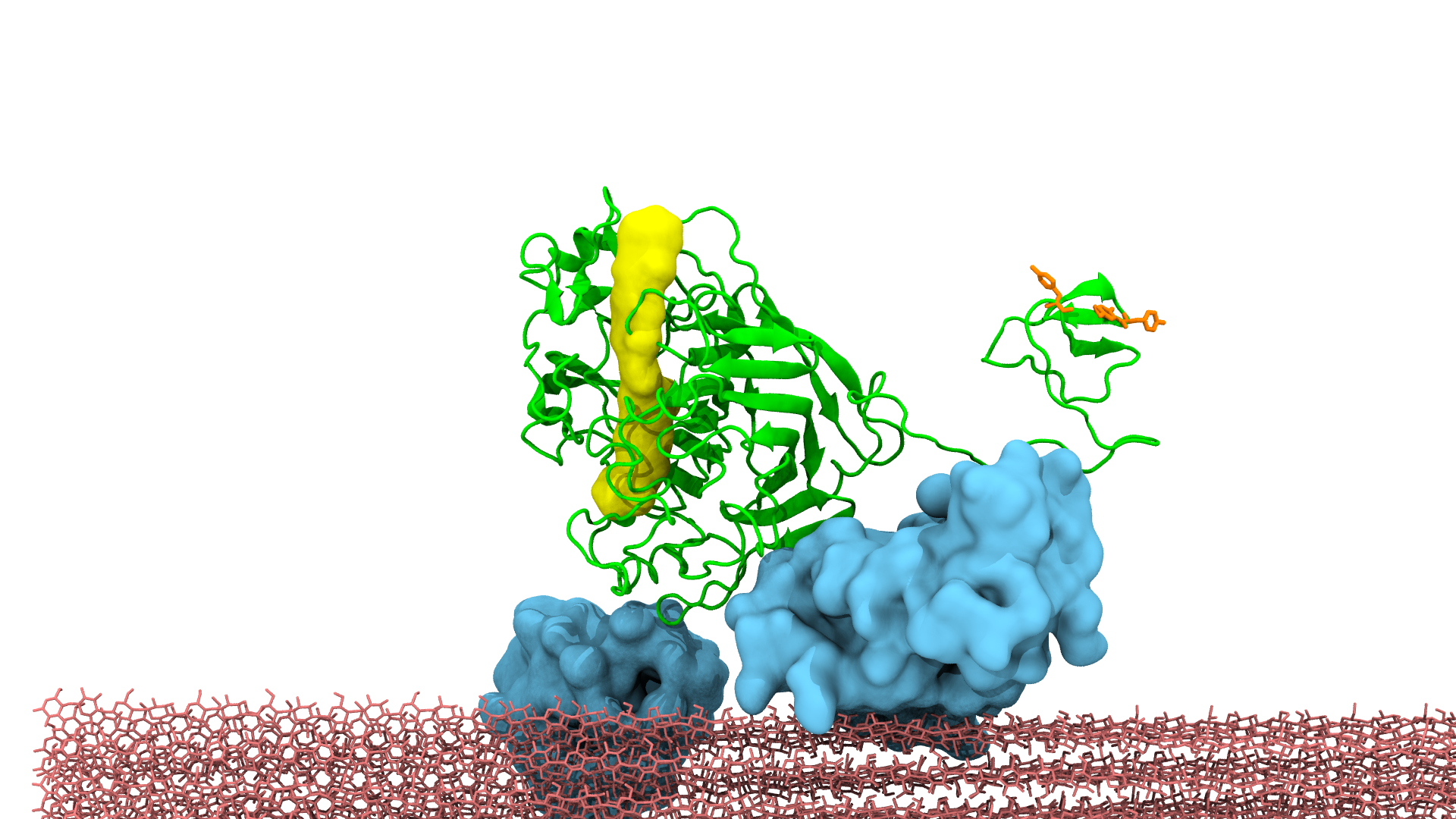

Supplement: Supplementary file 11 — 10.1186/s13068-015-0379-8 A zip archive containing a gallery of each of the cellulases that bound to cellulose in the context of their environment. Each image within the gallery is one snapshot taken from the end of the trajectory showing the relative position of each enzyme (green) that makes contact with the cellulose (red). Nearby lignins are shown in blue, and the substrate tunnel is a yellow surface to orient the viewer. The three tyrosine residues are shown in orange. Note that for each protein, there are 4 images, taken from different relative orientations to the cellulose fibril (0, 90, 180, and 270), and are labeled accordingly in their filenames. [file 13068_2015_379_MOESM11_ESM.zip › gallery/C-3_P-16_180.png]

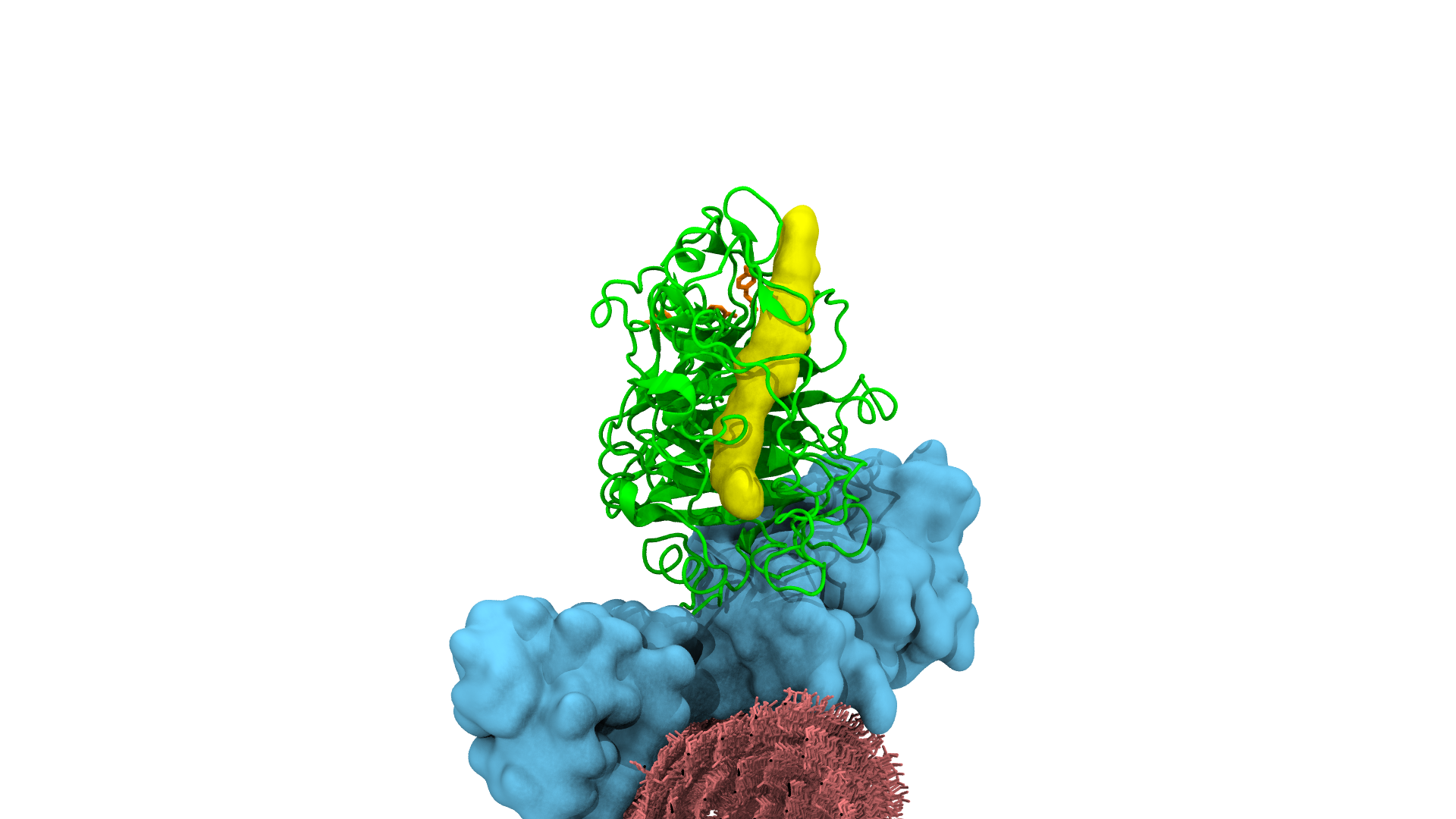

Supplement: Supplementary file 11 — 10.1186/s13068-015-0379-8 A zip archive containing a gallery of each of the cellulases that bound to cellulose in the context of their environment. Each image within the gallery is one snapshot taken from the end of the trajectory showing the relative position of each enzyme (green) that makes contact with the cellulose (red). Nearby lignins are shown in blue, and the substrate tunnel is a yellow surface to orient the viewer. The three tyrosine residues are shown in orange. Note that for each protein, there are 4 images, taken from different relative orientations to the cellulose fibril (0, 90, 180, and 270), and are labeled accordingly in their filenames. [file 13068_2015_379_MOESM11_ESM.zip › gallery/C-3_P-16_270.png]

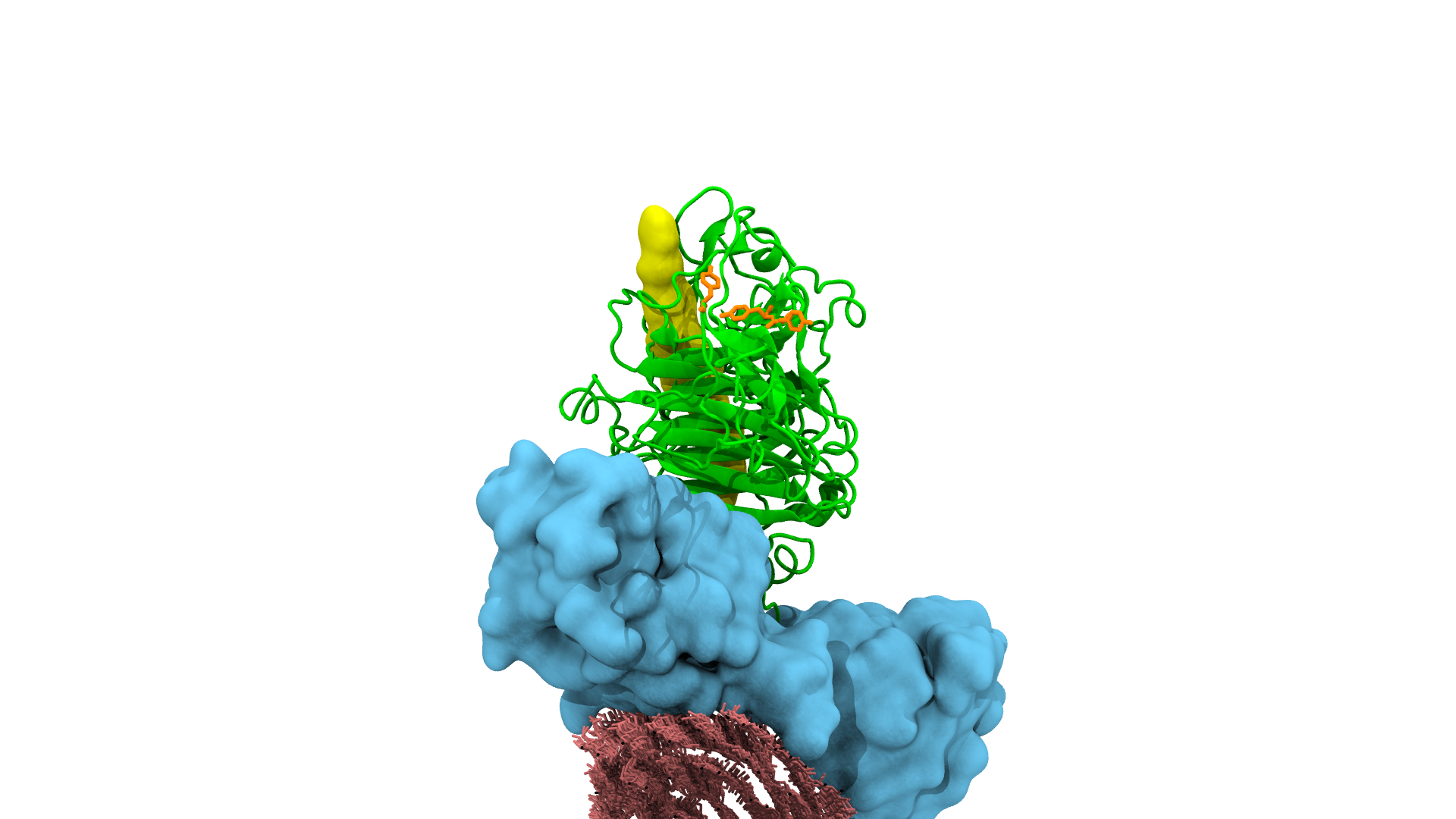

Supplement: Supplementary file 11 — 10.1186/s13068-015-0379-8 A zip archive containing a gallery of each of the cellulases that bound to cellulose in the context of their environment. Each image within the gallery is one snapshot taken from the end of the trajectory showing the relative position of each enzyme (green) that makes contact with the cellulose (red). Nearby lignins are shown in blue, and the substrate tunnel is a yellow surface to orient the viewer. The three tyrosine residues are shown in orange. Note that for each protein, there are 4 images, taken from different relative orientations to the cellulose fibril (0, 90, 180, and 270), and are labeled accordingly in their filenames. [file 13068_2015_379_MOESM11_ESM.zip › gallery/C-3_P-16_90.png]

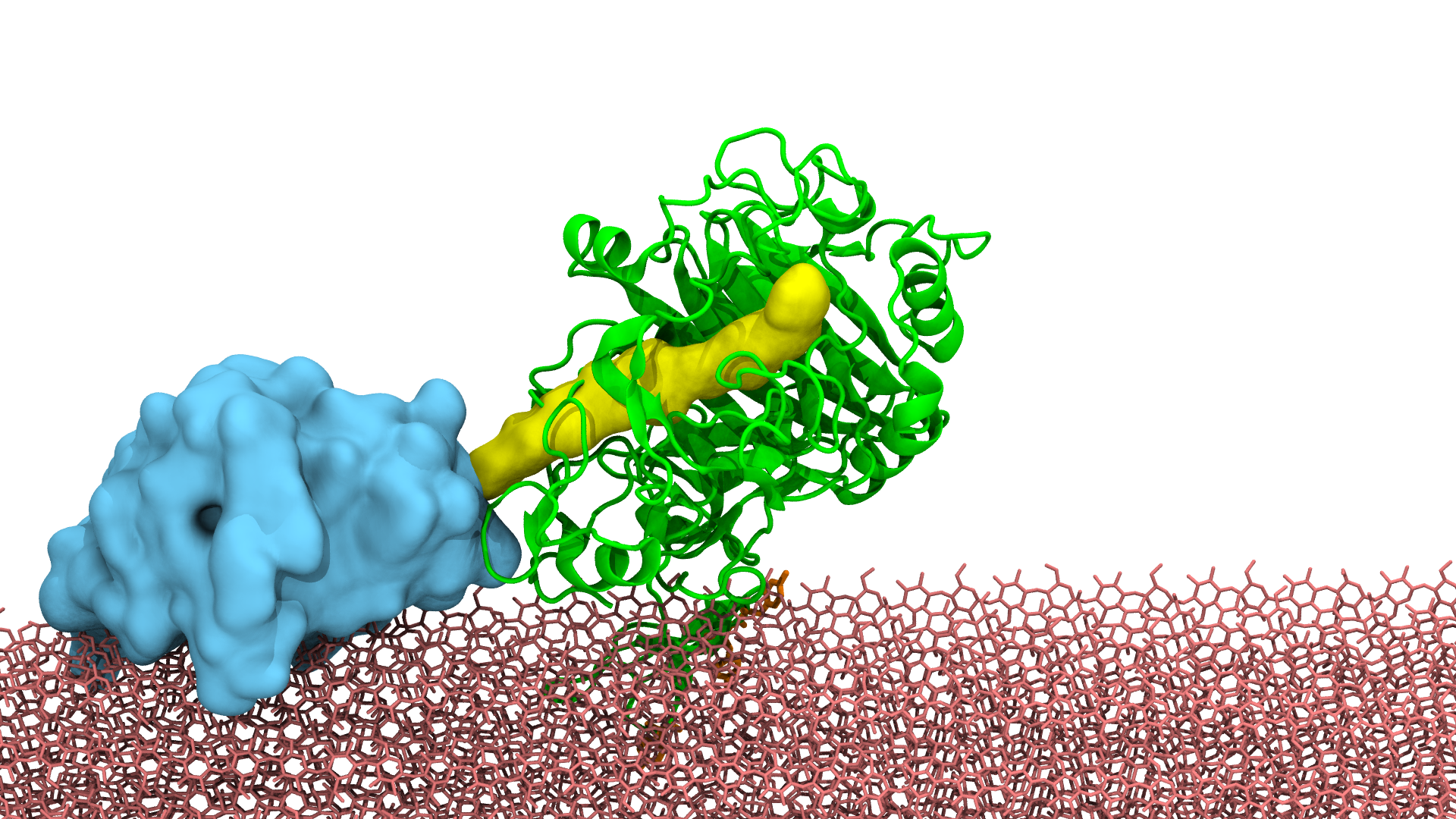

Supplement: Supplementary file 11 — 10.1186/s13068-015-0379-8 A zip archive containing a gallery of each of the cellulases that bound to cellulose in the context of their environment. Each image within the gallery is one snapshot taken from the end of the trajectory showing the relative position of each enzyme (green) that makes contact with the cellulose (red). Nearby lignins are shown in blue, and the substrate tunnel is a yellow surface to orient the viewer. The three tyrosine residues are shown in orange. Note that for each protein, there are 4 images, taken from different relative orientations to the cellulose fibril (0, 90, 180, and 270), and are labeled accordingly in their filenames. [file 13068_2015_379_MOESM11_ESM.zip › gallery/C-3_P-19_0.png]

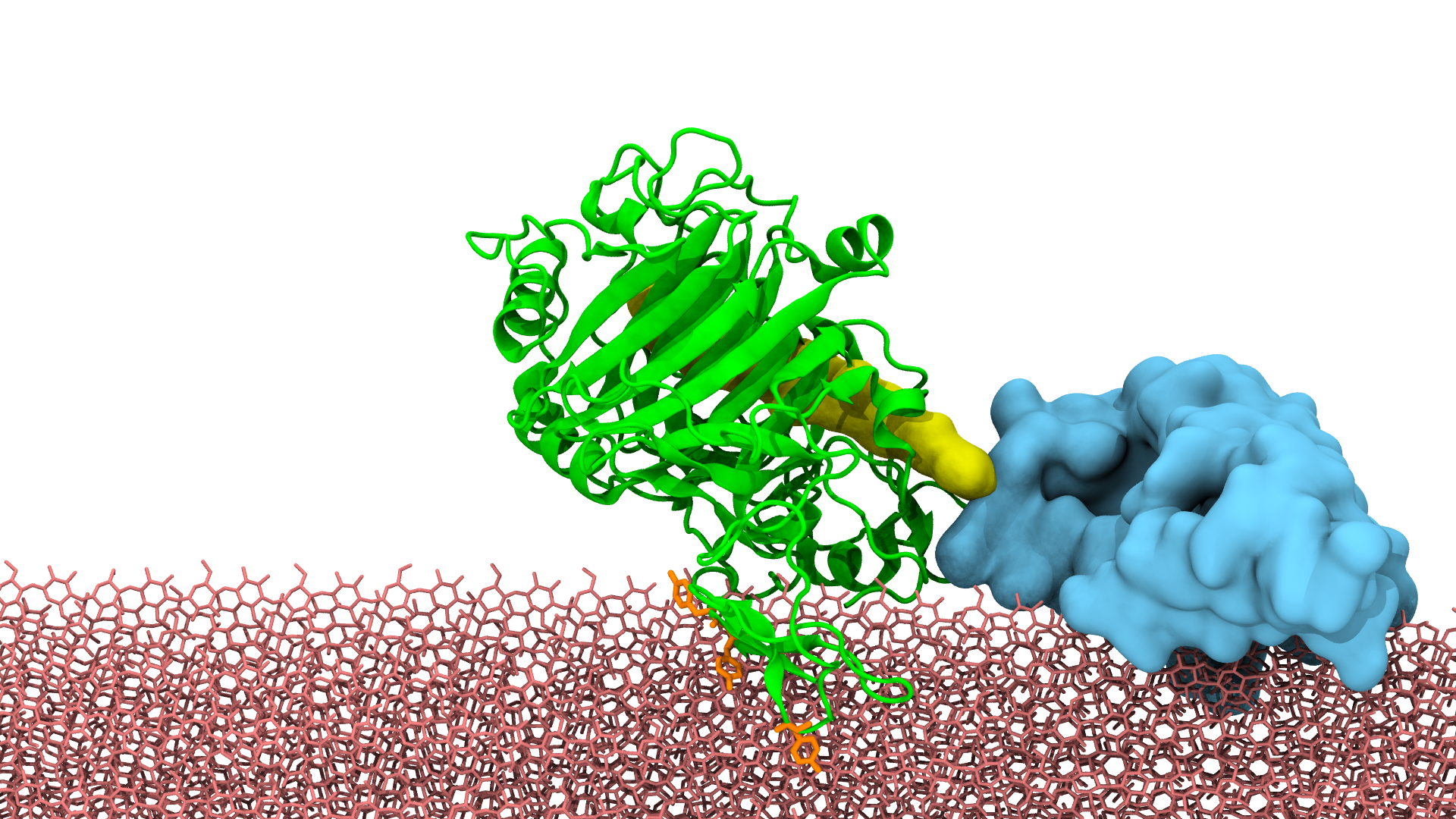

Supplement: Supplementary file 11 — 10.1186/s13068-015-0379-8 A zip archive containing a gallery of each of the cellulases that bound to cellulose in the context of their environment. Each image within the gallery is one snapshot taken from the end of the trajectory showing the relative position of each enzyme (green) that makes contact with the cellulose (red). Nearby lignins are shown in blue, and the substrate tunnel is a yellow surface to orient the viewer. The three tyrosine residues are shown in orange. Note that for each protein, there are 4 images, taken from different relative orientations to the cellulose fibril (0, 90, 180, and 270), and are labeled accordingly in their filenames. [file 13068_2015_379_MOESM11_ESM.zip › gallery/C-3_P-19_180.png]

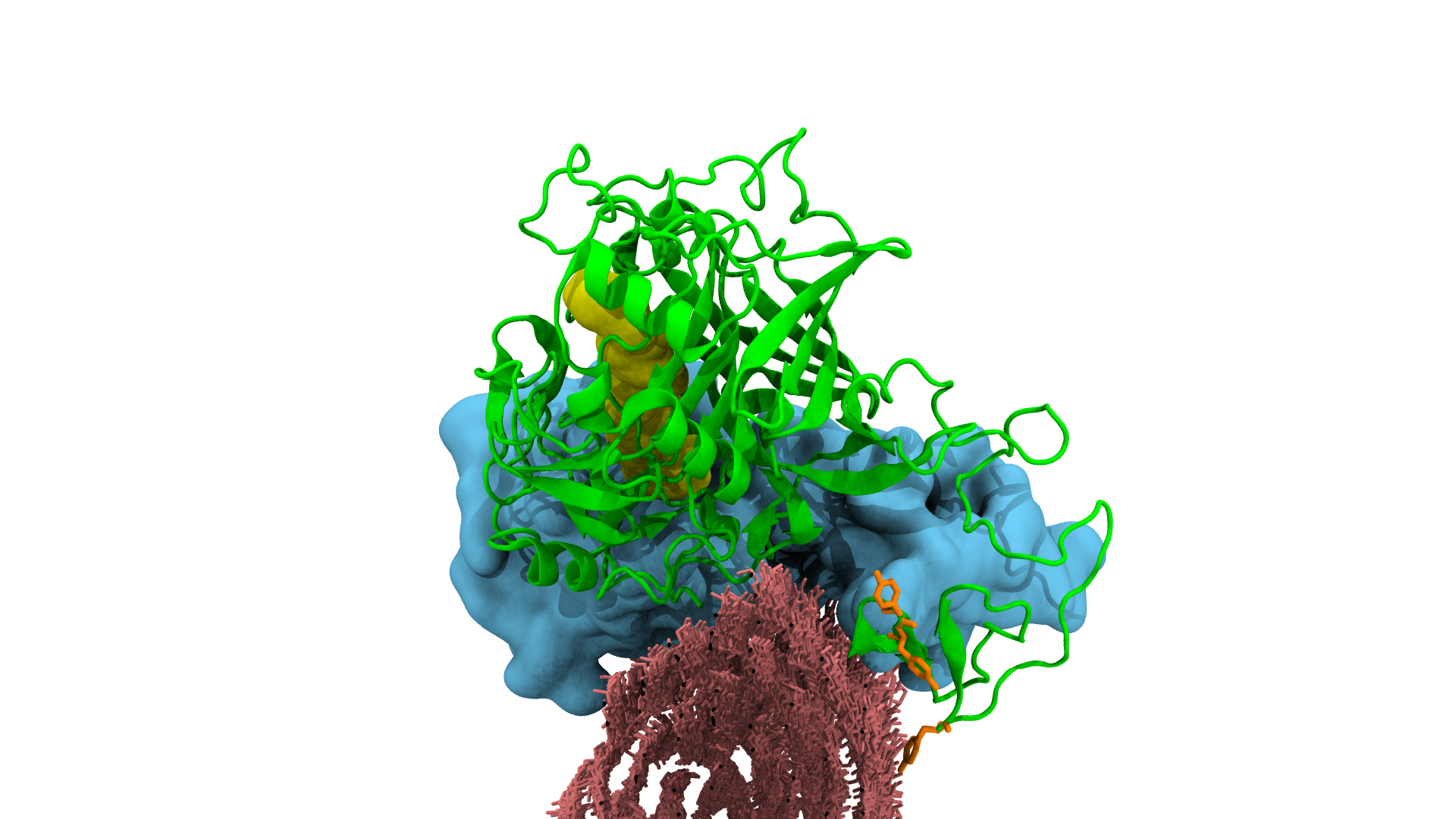

Supplement: Supplementary file 11 — 10.1186/s13068-015-0379-8 A zip archive containing a gallery of each of the cellulases that bound to cellulose in the context of their environment. Each image within the gallery is one snapshot taken from the end of the trajectory showing the relative position of each enzyme (green) that makes contact with the cellulose (red). Nearby lignins are shown in blue, and the substrate tunnel is a yellow surface to orient the viewer. The three tyrosine residues are shown in orange. Note that for each protein, there are 4 images, taken from different relative orientations to the cellulose fibril (0, 90, 180, and 270), and are labeled accordingly in their filenames. [file 13068_2015_379_MOESM11_ESM.zip › gallery/C-3_P-19_270.png]

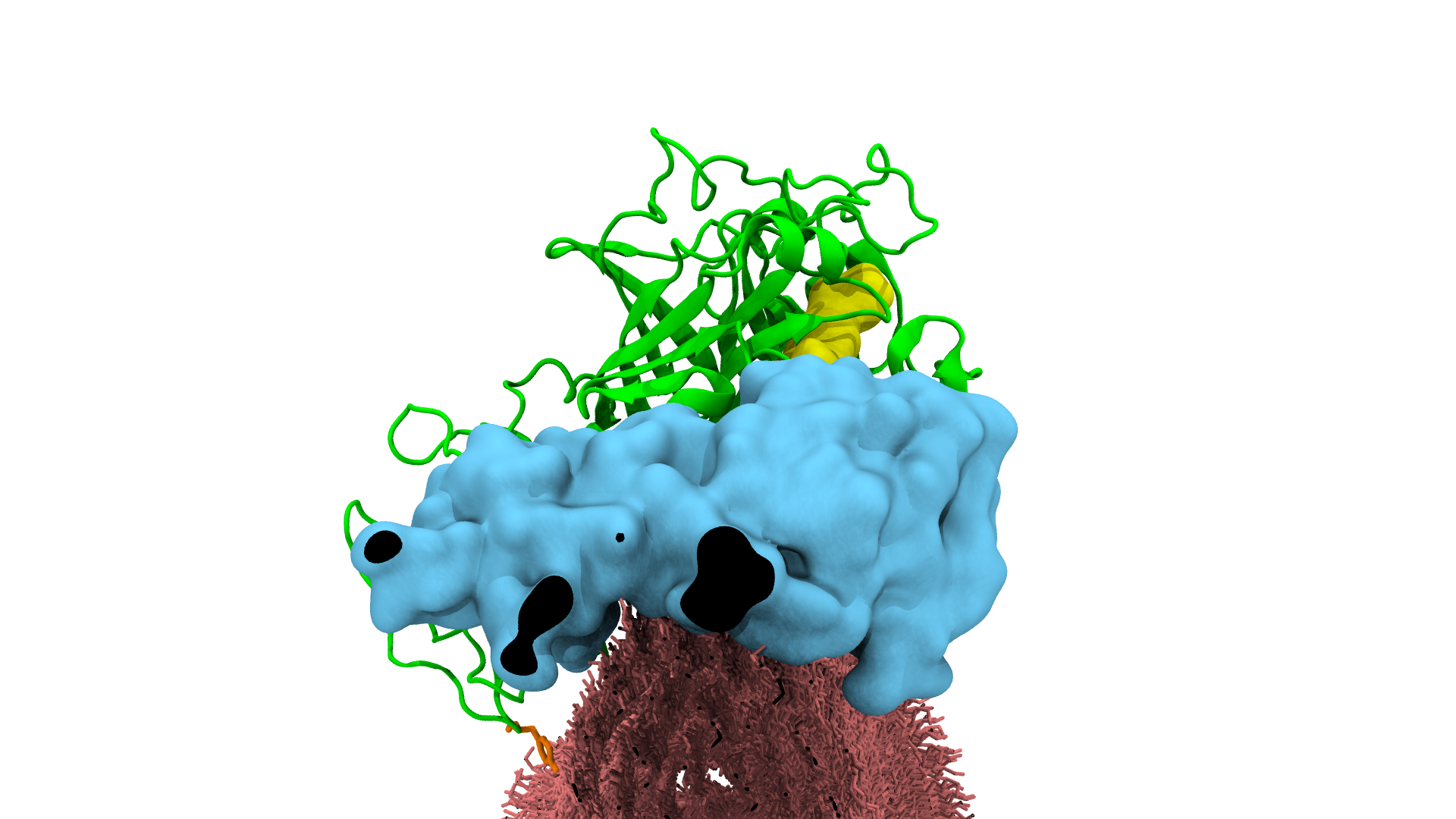

Supplement: Supplementary file 11 — 10.1186/s13068-015-0379-8 A zip archive containing a gallery of each of the cellulases that bound to cellulose in the context of their environment. Each image within the gallery is one snapshot taken from the end of the trajectory showing the relative position of each enzyme (green) that makes contact with the cellulose (red). Nearby lignins are shown in blue, and the substrate tunnel is a yellow surface to orient the viewer. The three tyrosine residues are shown in orange. Note that for each protein, there are 4 images, taken from different relative orientations to the cellulose fibril (0, 90, 180, and 270), and are labeled accordingly in their filenames. [file 13068_2015_379_MOESM11_ESM.zip › gallery/C-3_P-19_90.png]

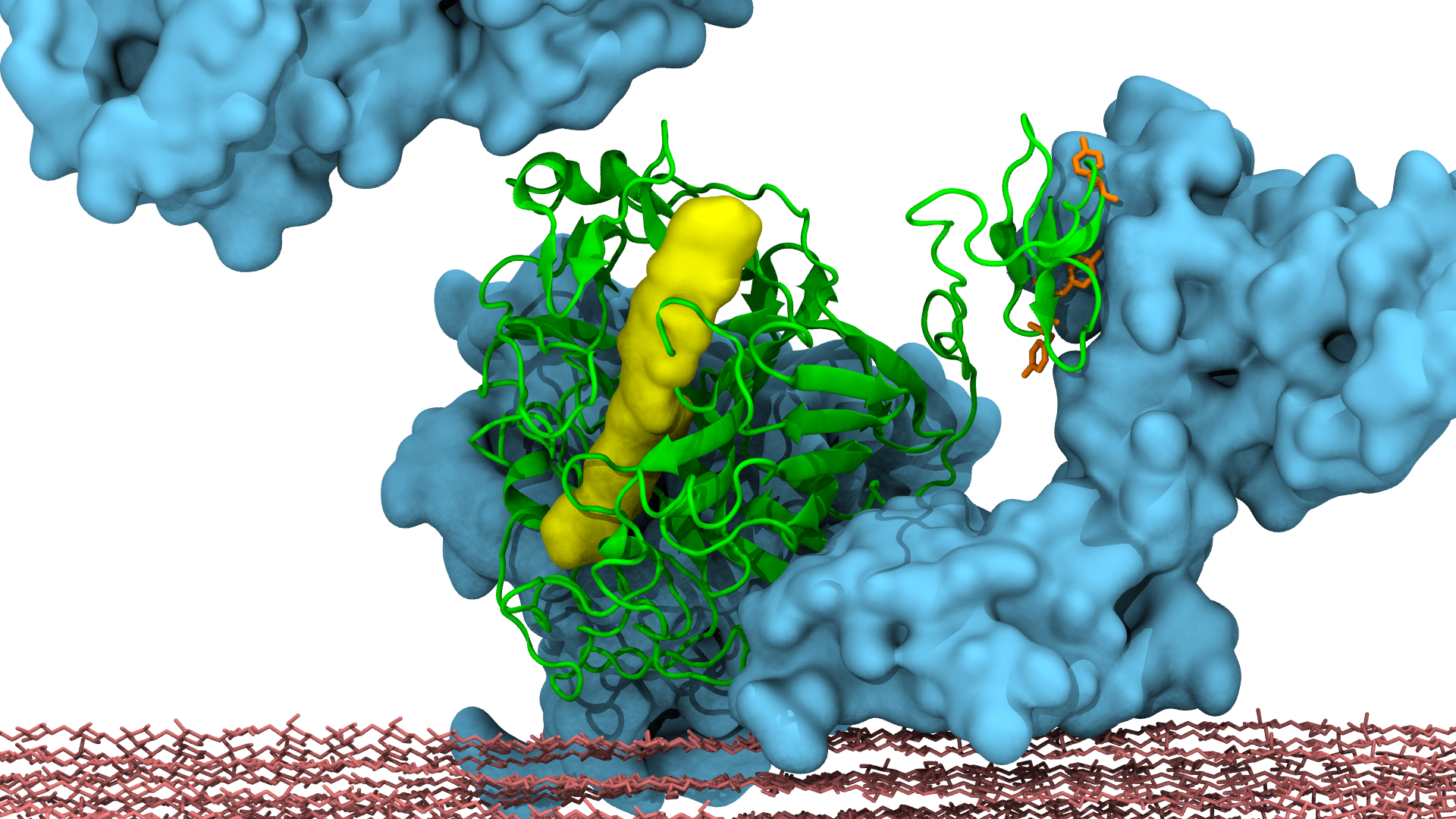

Supplement: Supplementary file 11 — 10.1186/s13068-015-0379-8 A zip archive containing a gallery of each of the cellulases that bound to cellulose in the context of their environment. Each image within the gallery is one snapshot taken from the end of the trajectory showing the relative position of each enzyme (green) that makes contact with the cellulose (red). Nearby lignins are shown in blue, and the substrate tunnel is a yellow surface to orient the viewer. The three tyrosine residues are shown in orange. Note that for each protein, there are 4 images, taken from different relative orientations to the cellulose fibril (0, 90, 180, and 270), and are labeled accordingly in their filenames. [file 13068_2015_379_MOESM11_ESM.zip › gallery/C-3_P-43_0.png]

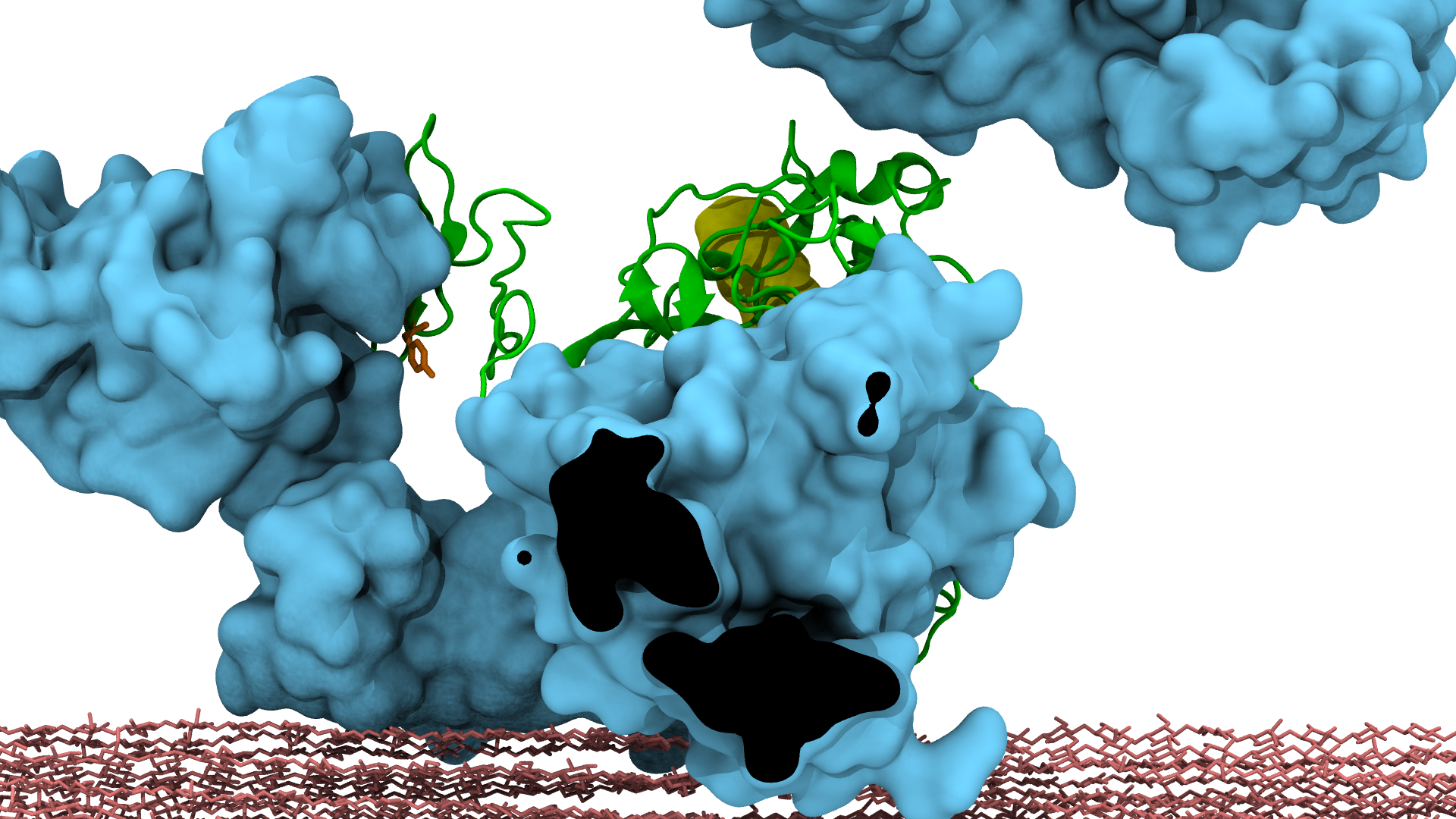

Supplement: Supplementary file 11 — 10.1186/s13068-015-0379-8 A zip archive containing a gallery of each of the cellulases that bound to cellulose in the context of their environment. Each image within the gallery is one snapshot taken from the end of the trajectory showing the relative position of each enzyme (green) that makes contact with the cellulose (red). Nearby lignins are shown in blue, and the substrate tunnel is a yellow surface to orient the viewer. The three tyrosine residues are shown in orange. Note that for each protein, there are 4 images, taken from different relative orientations to the cellulose fibril (0, 90, 180, and 270), and are labeled accordingly in their filenames. [file 13068_2015_379_MOESM11_ESM.zip › gallery/C-3_P-43_180.png]

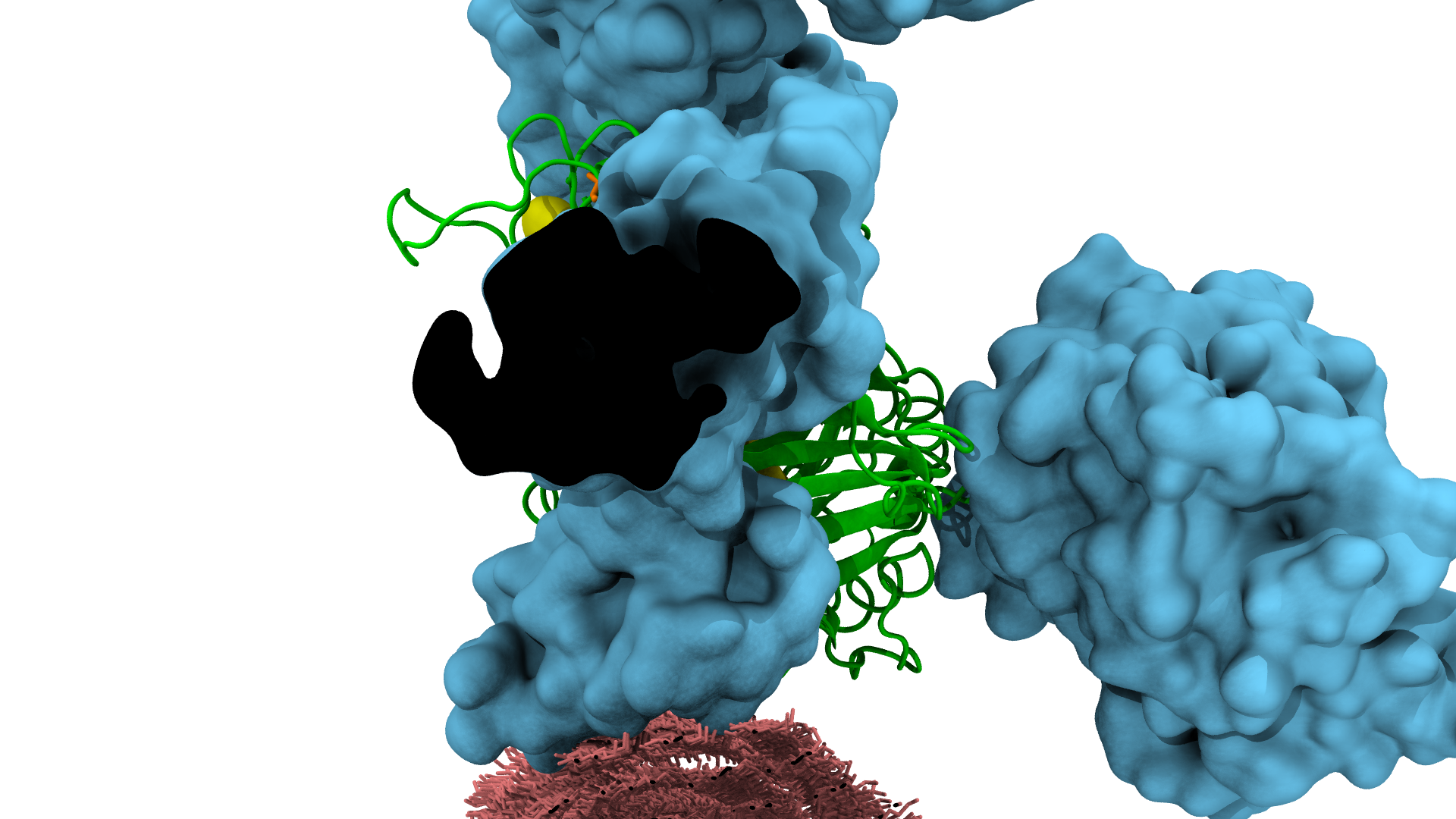

Supplement: Supplementary file 11 — 10.1186/s13068-015-0379-8 A zip archive containing a gallery of each of the cellulases that bound to cellulose in the context of their environment. Each image within the gallery is one snapshot taken from the end of the trajectory showing the relative position of each enzyme (green) that makes contact with the cellulose (red). Nearby lignins are shown in blue, and the substrate tunnel is a yellow surface to orient the viewer. The three tyrosine residues are shown in orange. Note that for each protein, there are 4 images, taken from different relative orientations to the cellulose fibril (0, 90, 180, and 270), and are labeled accordingly in their filenames. [file 13068_2015_379_MOESM11_ESM.zip › gallery/C-3_P-43_270.png]

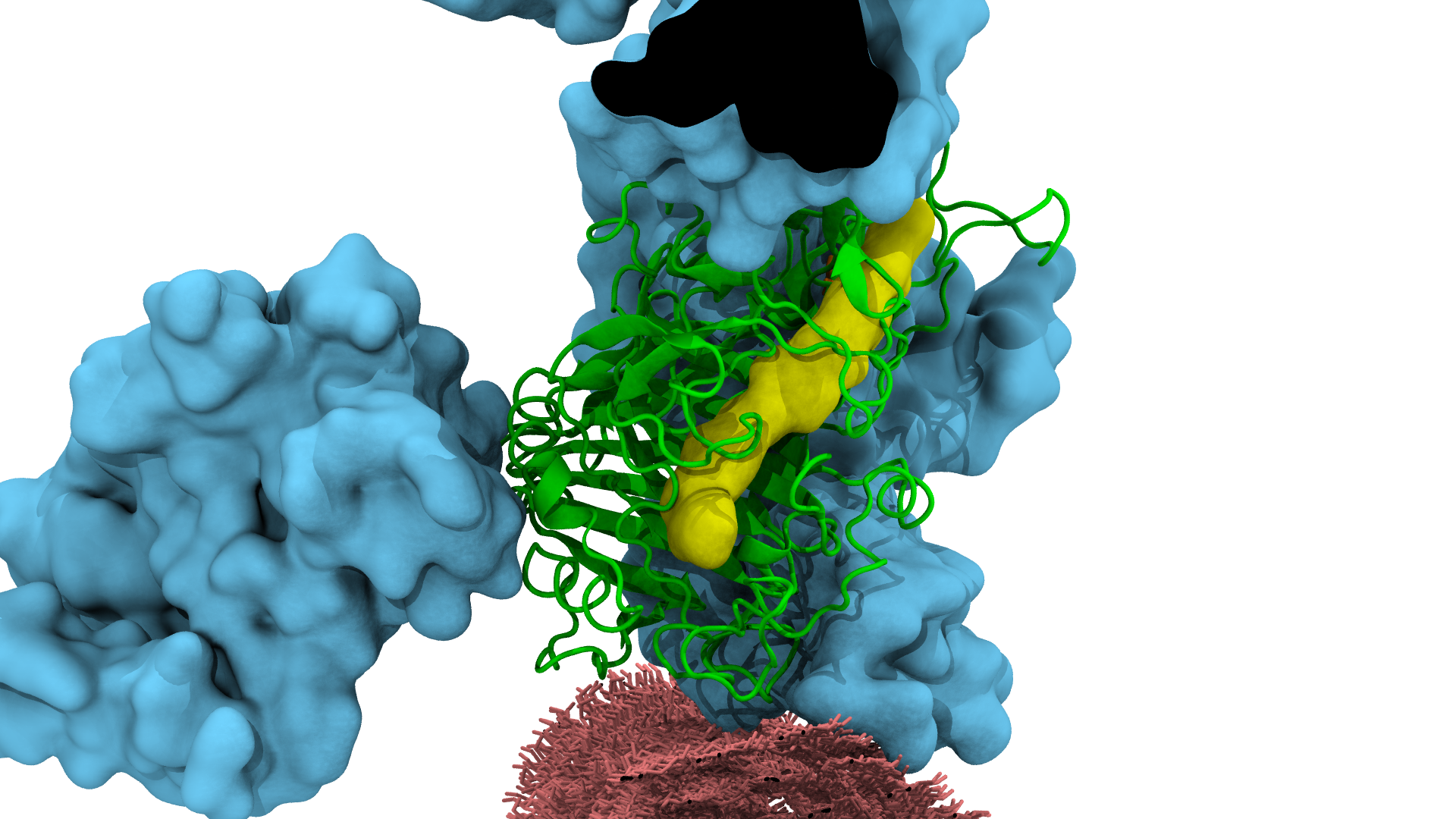

Supplement: Supplementary file 11 — 10.1186/s13068-015-0379-8 A zip archive containing a gallery of each of the cellulases that bound to cellulose in the context of their environment. Each image within the gallery is one snapshot taken from the end of the trajectory showing the relative position of each enzyme (green) that makes contact with the cellulose (red). Nearby lignins are shown in blue, and the substrate tunnel is a yellow surface to orient the viewer. The three tyrosine residues are shown in orange. Note that for each protein, there are 4 images, taken from different relative orientations to the cellulose fibril (0, 90, 180, and 270), and are labeled accordingly in their filenames. [file 13068_2015_379_MOESM11_ESM.zip › gallery/C-3_P-43_90.png]

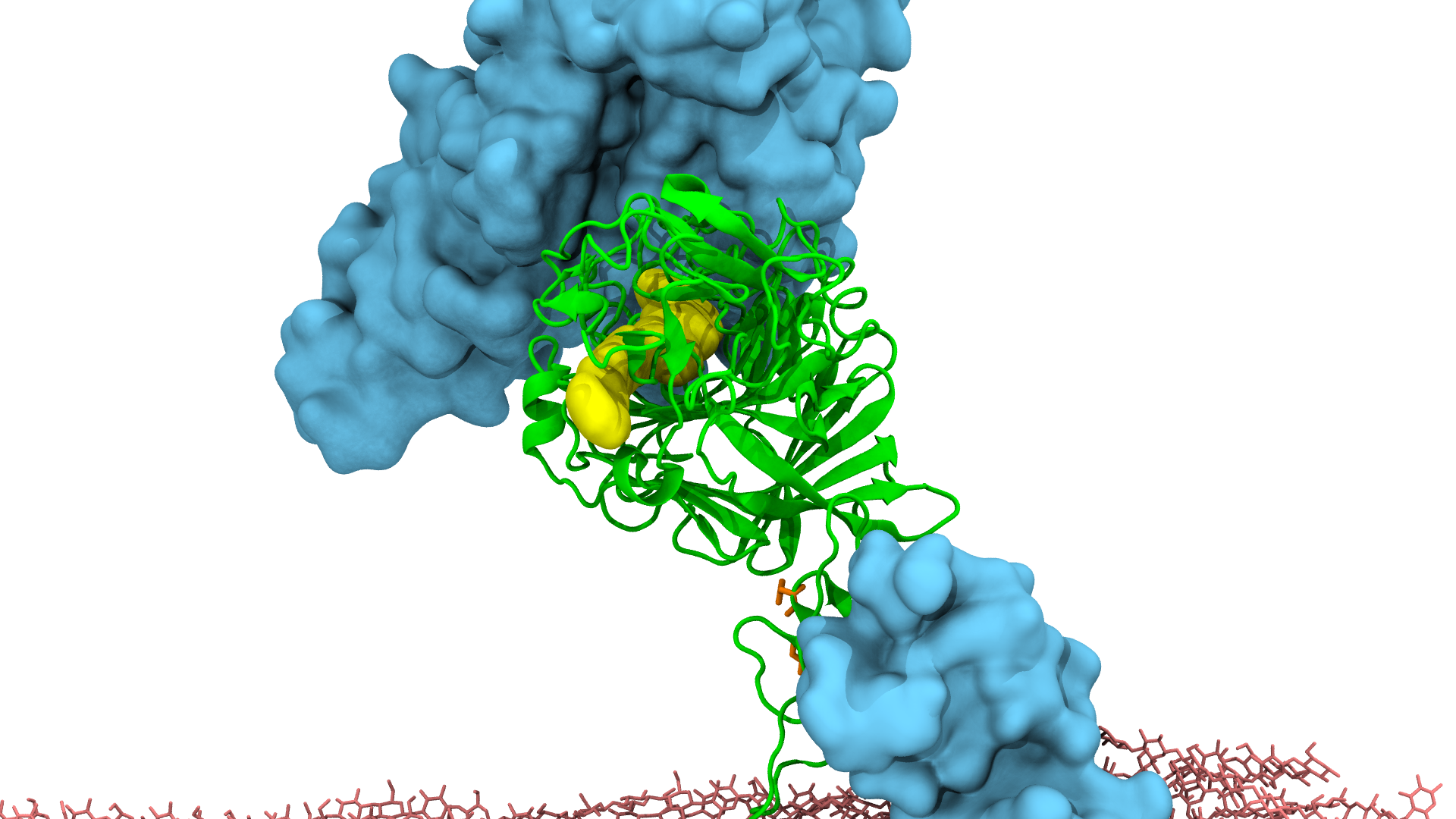

Supplement: Supplementary file 11 — 10.1186/s13068-015-0379-8 A zip archive containing a gallery of each of the cellulases that bound to cellulose in the context of their environment. Each image within the gallery is one snapshot taken from the end of the trajectory showing the relative position of each enzyme (green) that makes contact with the cellulose (red). Nearby lignins are shown in blue, and the substrate tunnel is a yellow surface to orient the viewer. The three tyrosine residues are shown in orange. Note that for each protein, there are 4 images, taken from different relative orientations to the cellulose fibril (0, 90, 180, and 270), and are labeled accordingly in their filenames. [file 13068_2015_379_MOESM11_ESM.zip › gallery/C-4_P-07_0.png]

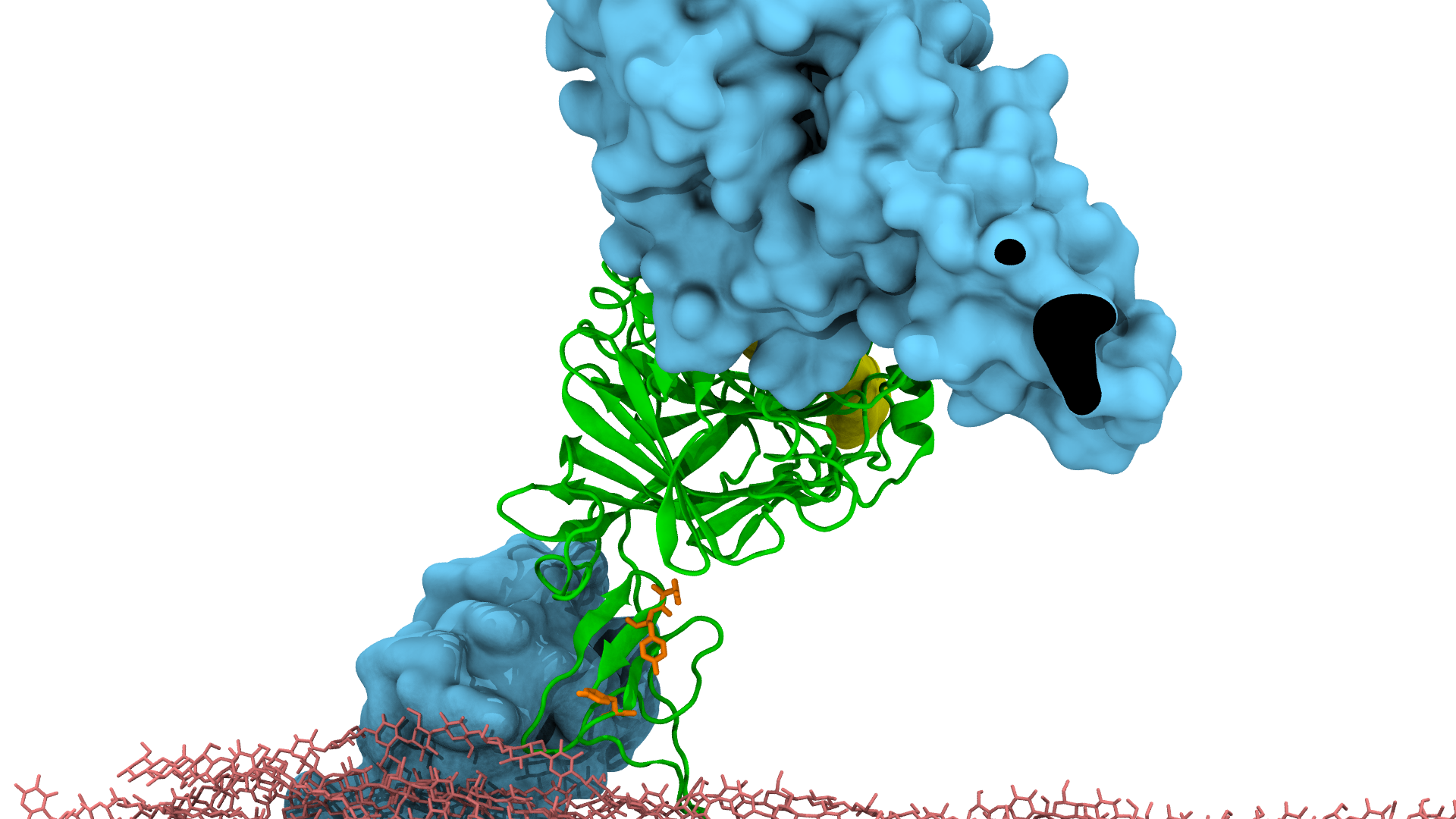

Supplement: Supplementary file 11 — 10.1186/s13068-015-0379-8 A zip archive containing a gallery of each of the cellulases that bound to cellulose in the context of their environment. Each image within the gallery is one snapshot taken from the end of the trajectory showing the relative position of each enzyme (green) that makes contact with the cellulose (red). Nearby lignins are shown in blue, and the substrate tunnel is a yellow surface to orient the viewer. The three tyrosine residues are shown in orange. Note that for each protein, there are 4 images, taken from different relative orientations to the cellulose fibril (0, 90, 180, and 270), and are labeled accordingly in their filenames. [file 13068_2015_379_MOESM11_ESM.zip › gallery/C-4_P-07_180.png]

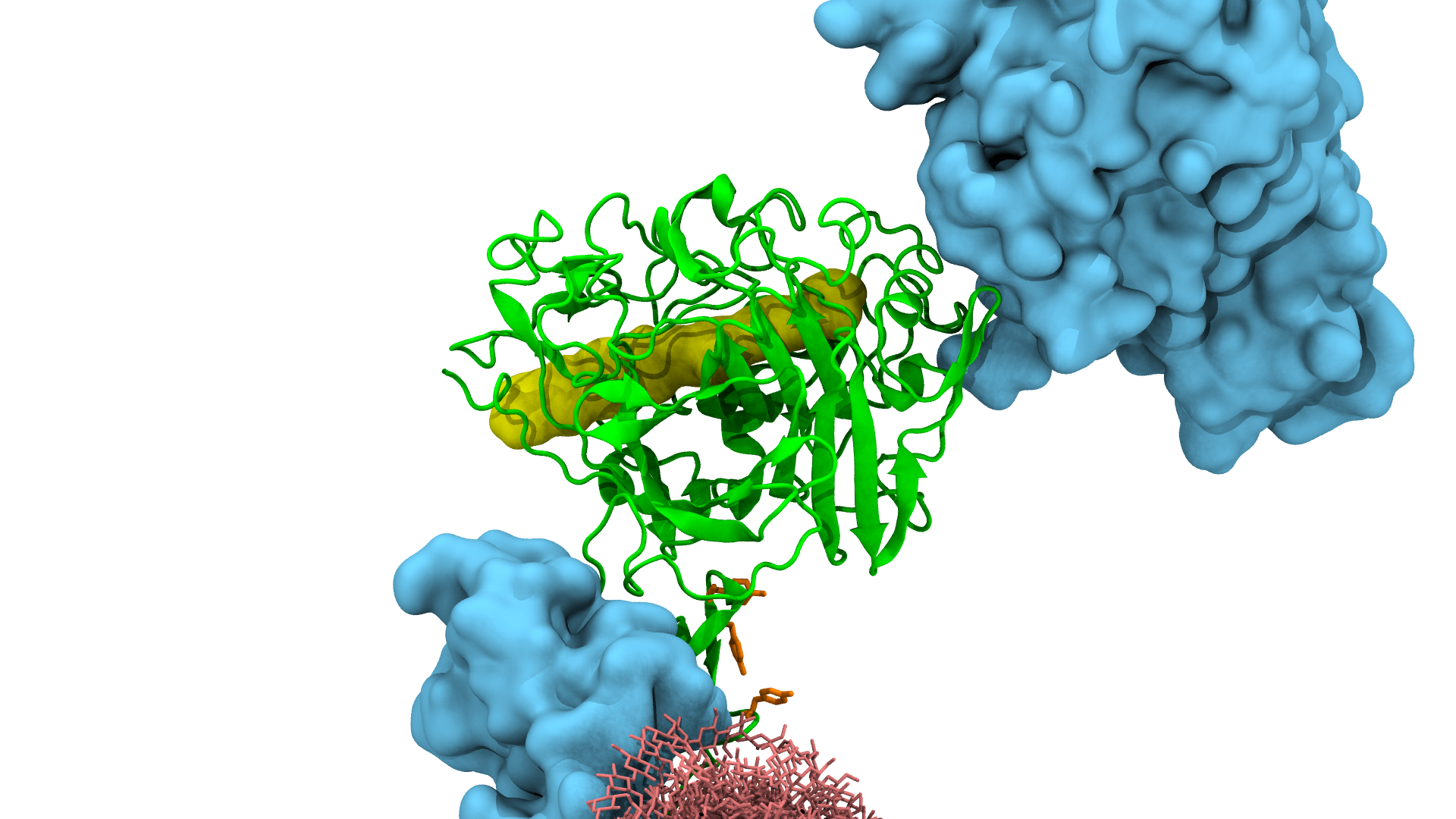

Supplement: Supplementary file 11 — 10.1186/s13068-015-0379-8 A zip archive containing a gallery of each of the cellulases that bound to cellulose in the context of their environment. Each image within the gallery is one snapshot taken from the end of the trajectory showing the relative position of each enzyme (green) that makes contact with the cellulose (red). Nearby lignins are shown in blue, and the substrate tunnel is a yellow surface to orient the viewer. The three tyrosine residues are shown in orange. Note that for each protein, there are 4 images, taken from different relative orientations to the cellulose fibril (0, 90, 180, and 270), and are labeled accordingly in their filenames. [file 13068_2015_379_MOESM11_ESM.zip › gallery/C-4_P-07_270.png]

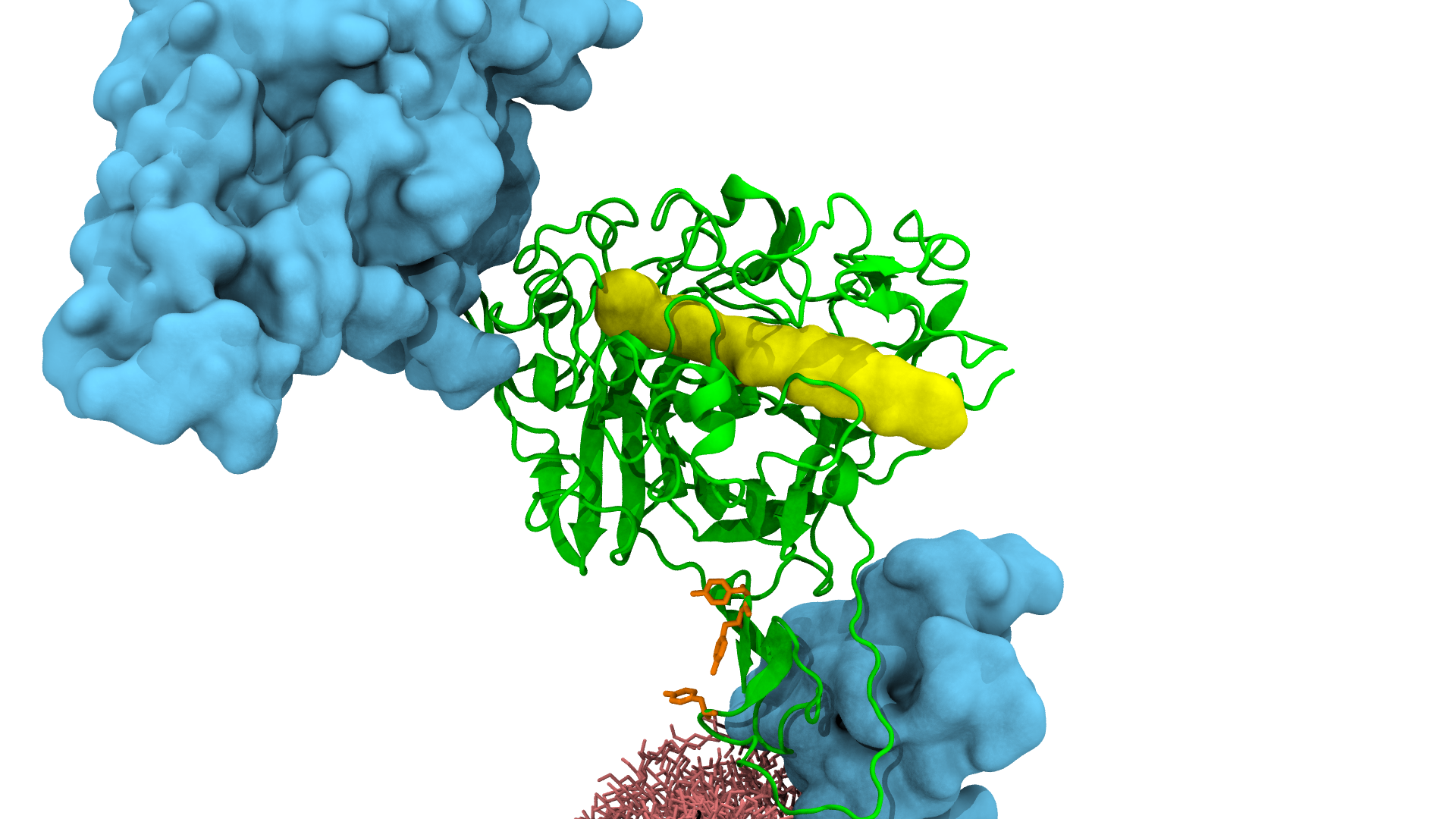

Supplement: Supplementary file 11 — 10.1186/s13068-015-0379-8 A zip archive containing a gallery of each of the cellulases that bound to cellulose in the context of their environment. Each image within the gallery is one snapshot taken from the end of the trajectory showing the relative position of each enzyme (green) that makes contact with the cellulose (red). Nearby lignins are shown in blue, and the substrate tunnel is a yellow surface to orient the viewer. The three tyrosine residues are shown in orange. Note that for each protein, there are 4 images, taken from different relative orientations to the cellulose fibril (0, 90, 180, and 270), and are labeled accordingly in their filenames. [file 13068_2015_379_MOESM11_ESM.zip › gallery/C-4_P-07_90.png]

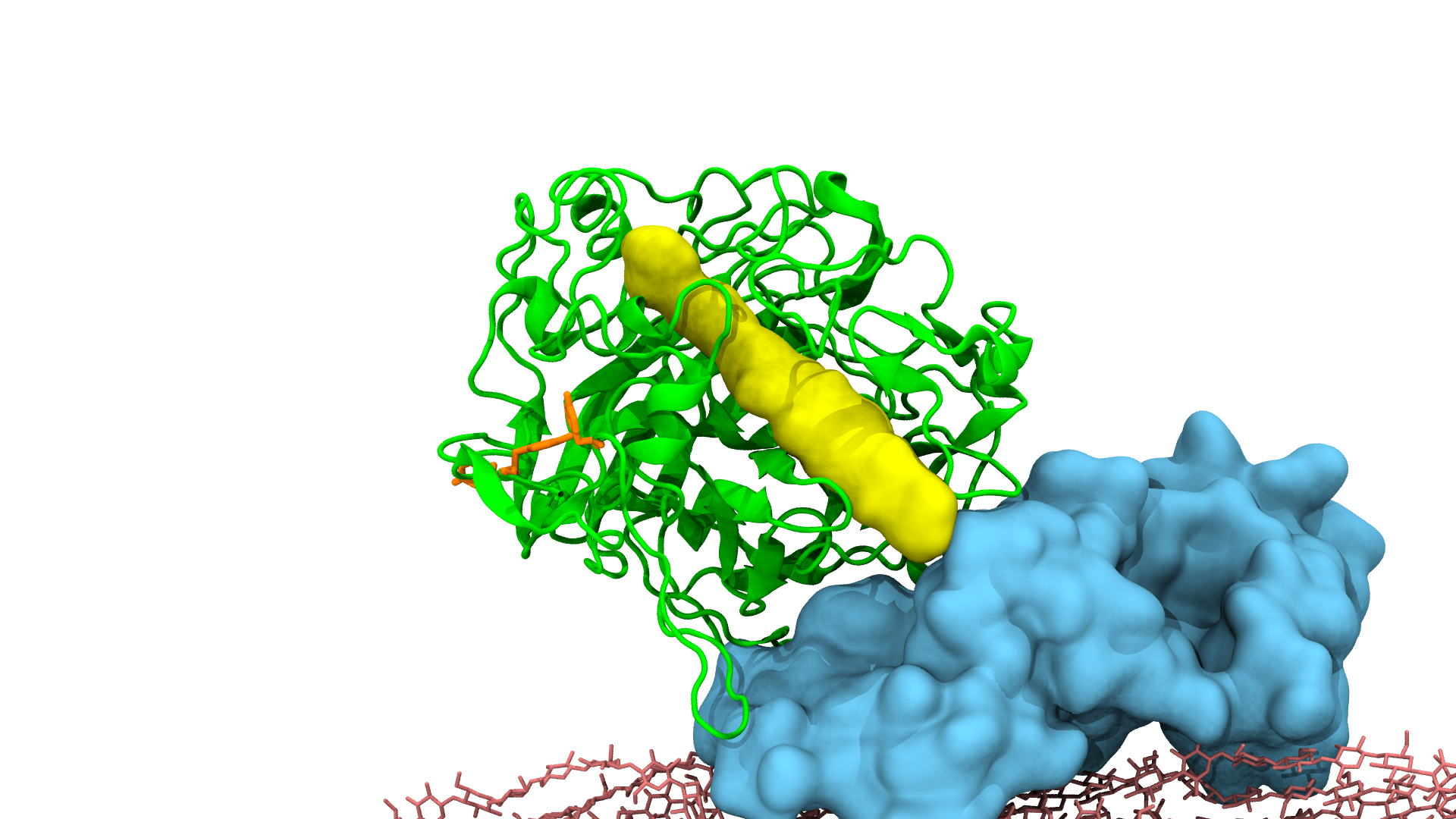

Supplement: Supplementary file 11 — 10.1186/s13068-015-0379-8 A zip archive containing a gallery of each of the cellulases that bound to cellulose in the context of their environment. Each image within the gallery is one snapshot taken from the end of the trajectory showing the relative position of each enzyme (green) that makes contact with the cellulose (red). Nearby lignins are shown in blue, and the substrate tunnel is a yellow surface to orient the viewer. The three tyrosine residues are shown in orange. Note that for each protein, there are 4 images, taken from different relative orientations to the cellulose fibril (0, 90, 180, and 270), and are labeled accordingly in their filenames. [file 13068_2015_379_MOESM11_ESM.zip › gallery/C-4_P-18_0.png]

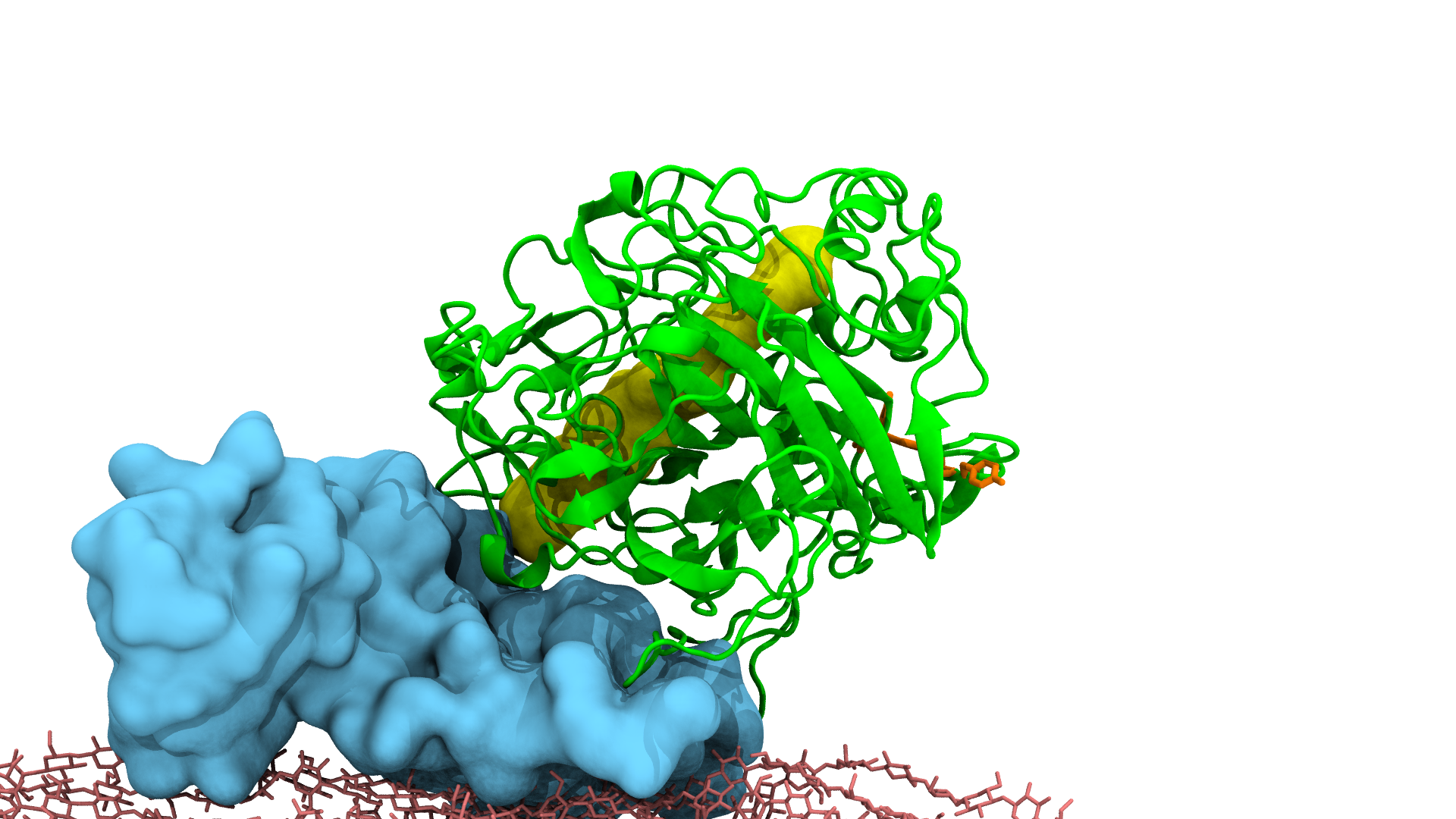

Supplement: Supplementary file 11 — 10.1186/s13068-015-0379-8 A zip archive containing a gallery of each of the cellulases that bound to cellulose in the context of their environment. Each image within the gallery is one snapshot taken from the end of the trajectory showing the relative position of each enzyme (green) that makes contact with the cellulose (red). Nearby lignins are shown in blue, and the substrate tunnel is a yellow surface to orient the viewer. The three tyrosine residues are shown in orange. Note that for each protein, there are 4 images, taken from different relative orientations to the cellulose fibril (0, 90, 180, and 270), and are labeled accordingly in their filenames. [file 13068_2015_379_MOESM11_ESM.zip › gallery/C-4_P-18_180.png]

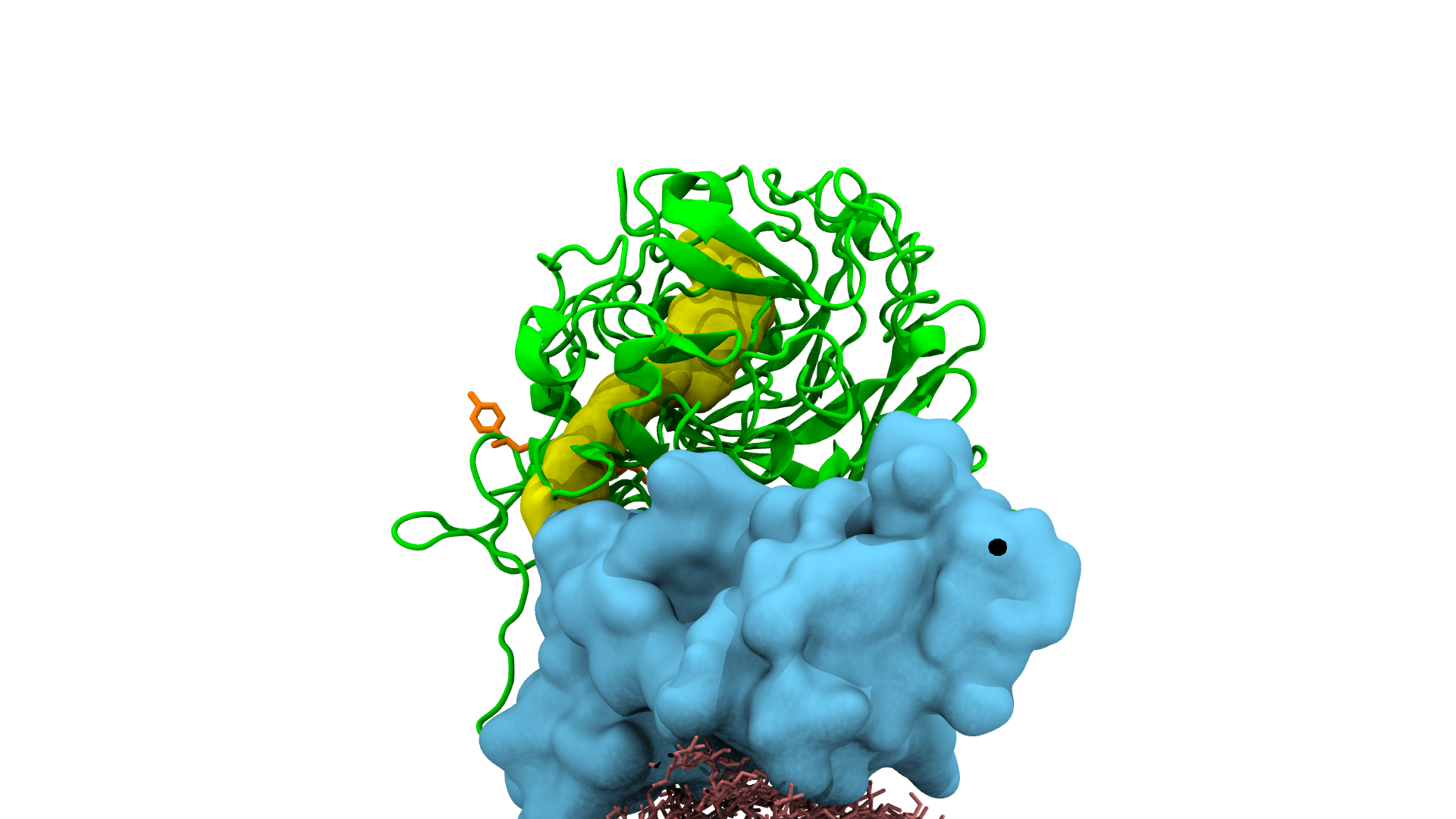

Supplement: Supplementary file 11 — 10.1186/s13068-015-0379-8 A zip archive containing a gallery of each of the cellulases that bound to cellulose in the context of their environment. Each image within the gallery is one snapshot taken from the end of the trajectory showing the relative position of each enzyme (green) that makes contact with the cellulose (red). Nearby lignins are shown in blue, and the substrate tunnel is a yellow surface to orient the viewer. The three tyrosine residues are shown in orange. Note that for each protein, there are 4 images, taken from different relative orientations to the cellulose fibril (0, 90, 180, and 270), and are labeled accordingly in their filenames. [file 13068_2015_379_MOESM11_ESM.zip › gallery/C-4_P-18_270.png]

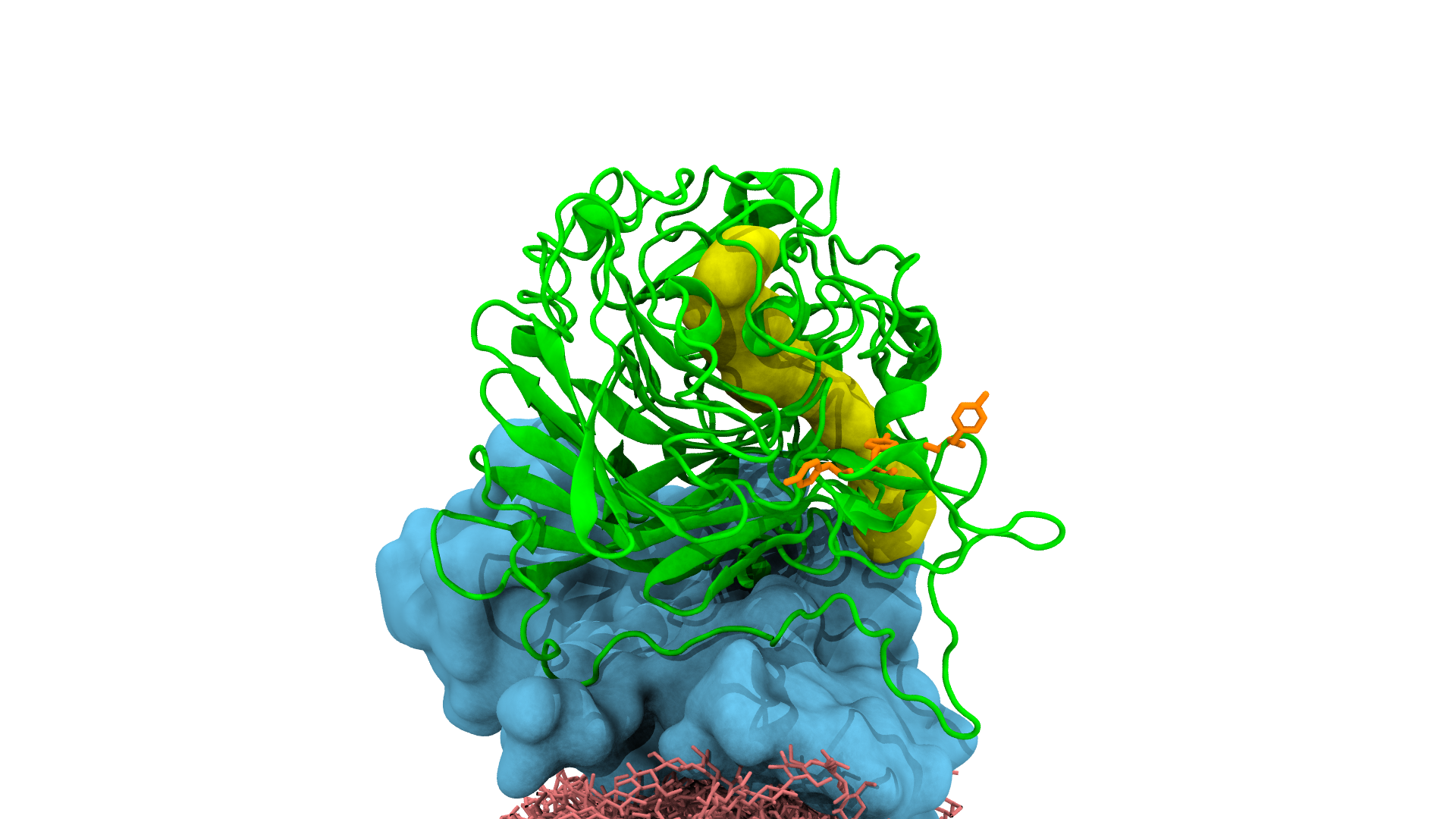

Supplement: Supplementary file 11 — 10.1186/s13068-015-0379-8 A zip archive containing a gallery of each of the cellulases that bound to cellulose in the context of their environment. Each image within the gallery is one snapshot taken from the end of the trajectory showing the relative position of each enzyme (green) that makes contact with the cellulose (red). Nearby lignins are shown in blue, and the substrate tunnel is a yellow surface to orient the viewer. The three tyrosine residues are shown in orange. Note that for each protein, there are 4 images, taken from different relative orientations to the cellulose fibril (0, 90, 180, and 270), and are labeled accordingly in their filenames. [file 13068_2015_379_MOESM11_ESM.zip › gallery/C-4_P-18_90.png]

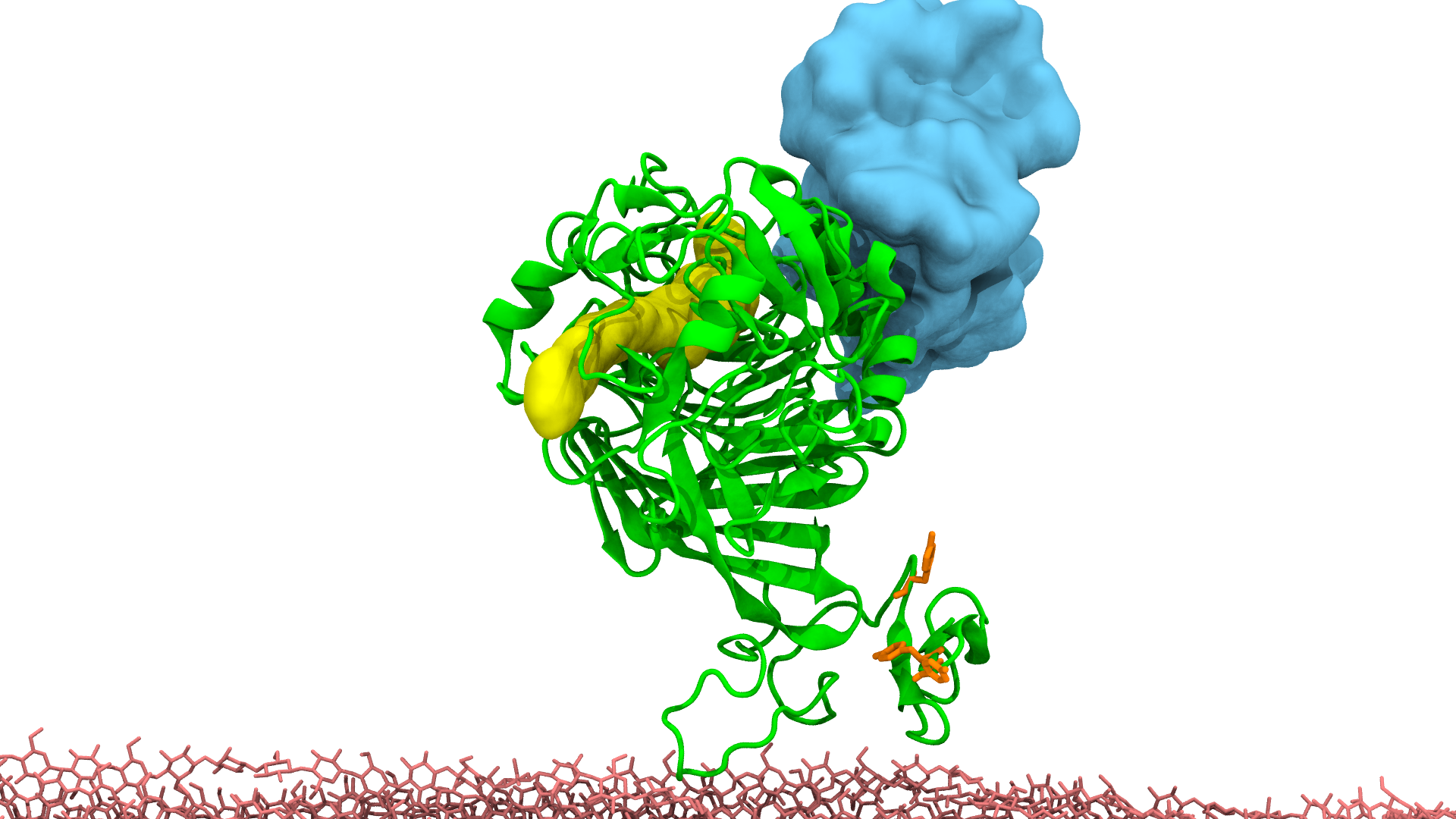

Supplement: Supplementary file 11 — 10.1186/s13068-015-0379-8 A zip archive containing a gallery of each of the cellulases that bound to cellulose in the context of their environment. Each image within the gallery is one snapshot taken from the end of the trajectory showing the relative position of each enzyme (green) that makes contact with the cellulose (red). Nearby lignins are shown in blue, and the substrate tunnel is a yellow surface to orient the viewer. The three tyrosine residues are shown in orange. Note that for each protein, there are 4 images, taken from different relative orientations to the cellulose fibril (0, 90, 180, and 270), and are labeled accordingly in their filenames. [file 13068_2015_379_MOESM11_ESM.zip › gallery/C-4_P-22_0.png]

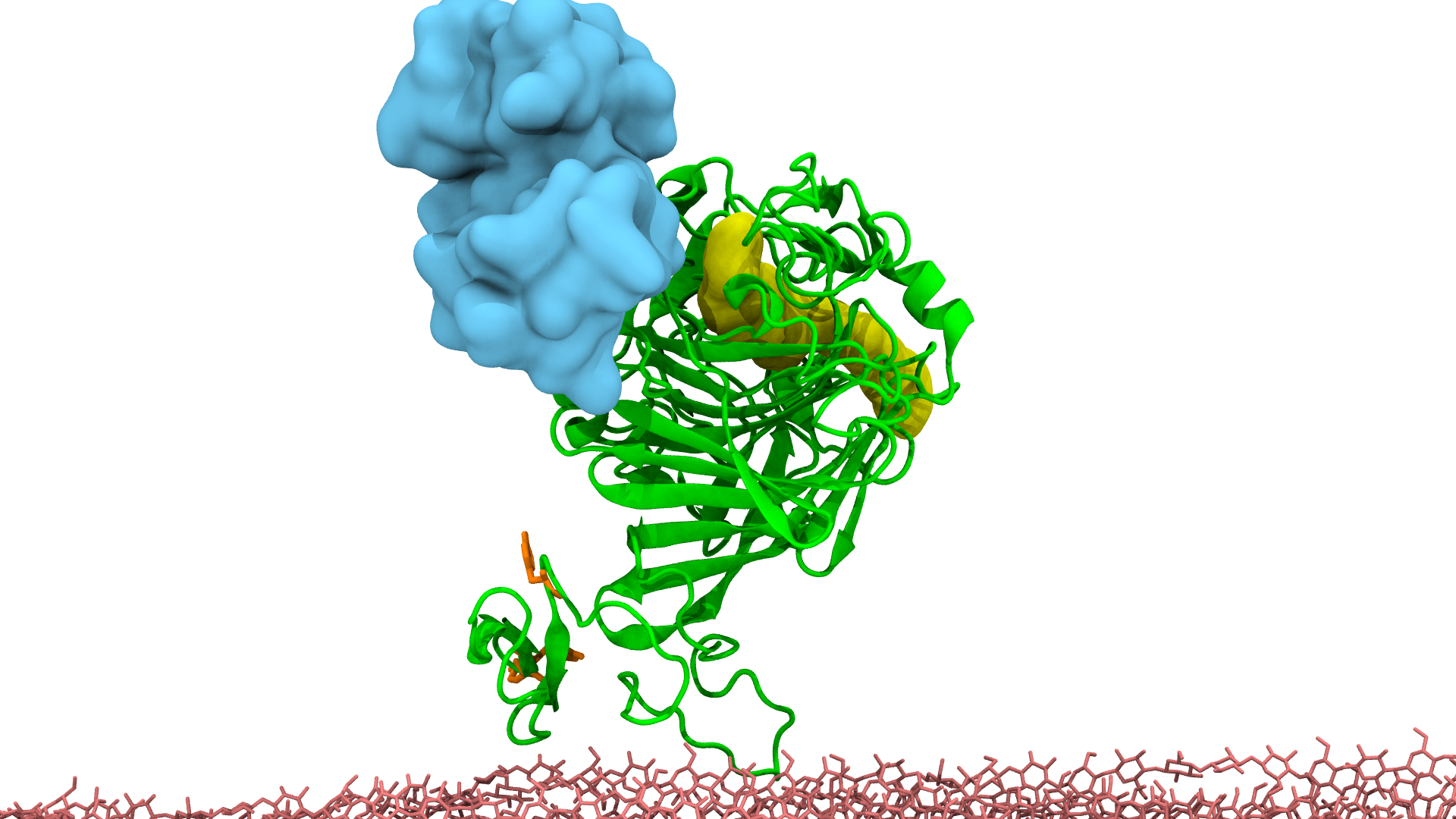

Supplement: Supplementary file 11 — 10.1186/s13068-015-0379-8 A zip archive containing a gallery of each of the cellulases that bound to cellulose in the context of their environment. Each image within the gallery is one snapshot taken from the end of the trajectory showing the relative position of each enzyme (green) that makes contact with the cellulose (red). Nearby lignins are shown in blue, and the substrate tunnel is a yellow surface to orient the viewer. The three tyrosine residues are shown in orange. Note that for each protein, there are 4 images, taken from different relative orientations to the cellulose fibril (0, 90, 180, and 270), and are labeled accordingly in their filenames. [file 13068_2015_379_MOESM11_ESM.zip › gallery/C-4_P-22_180.png]

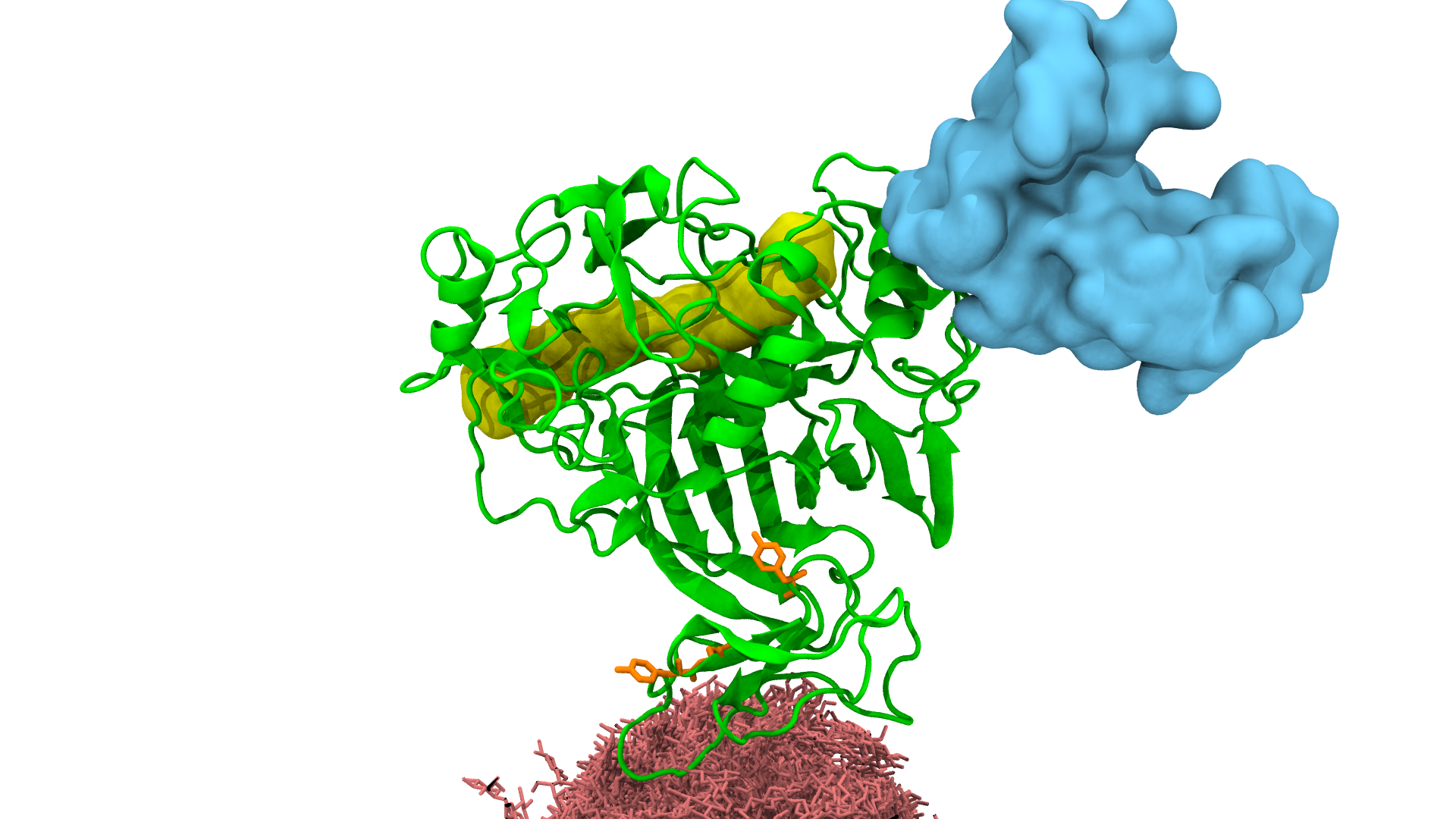

Supplement: Supplementary file 11 — 10.1186/s13068-015-0379-8 A zip archive containing a gallery of each of the cellulases that bound to cellulose in the context of their environment. Each image within the gallery is one snapshot taken from the end of the trajectory showing the relative position of each enzyme (green) that makes contact with the cellulose (red). Nearby lignins are shown in blue, and the substrate tunnel is a yellow surface to orient the viewer. The three tyrosine residues are shown in orange. Note that for each protein, there are 4 images, taken from different relative orientations to the cellulose fibril (0, 90, 180, and 270), and are labeled accordingly in their filenames. [file 13068_2015_379_MOESM11_ESM.zip › gallery/C-4_P-22_270.png]

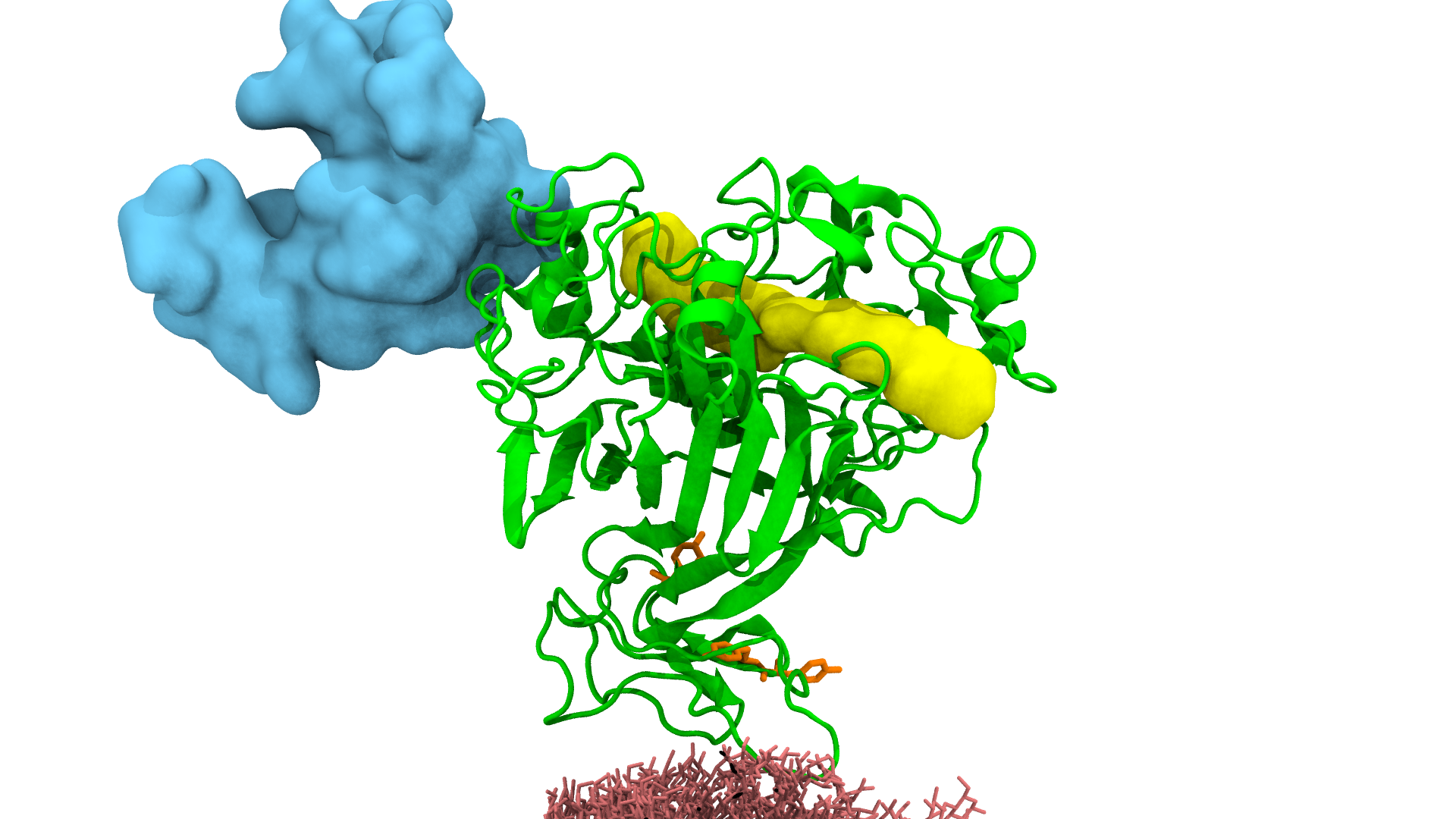

Supplement: Supplementary file 11 — 10.1186/s13068-015-0379-8 A zip archive containing a gallery of each of the cellulases that bound to cellulose in the context of their environment. Each image within the gallery is one snapshot taken from the end of the trajectory showing the relative position of each enzyme (green) that makes contact with the cellulose (red). Nearby lignins are shown in blue, and the substrate tunnel is a yellow surface to orient the viewer. The three tyrosine residues are shown in orange. Note that for each protein, there are 4 images, taken from different relative orientations to the cellulose fibril (0, 90, 180, and 270), and are labeled accordingly in their filenames. [file 13068_2015_379_MOESM11_ESM.zip › gallery/C-4_P-22_90.png]

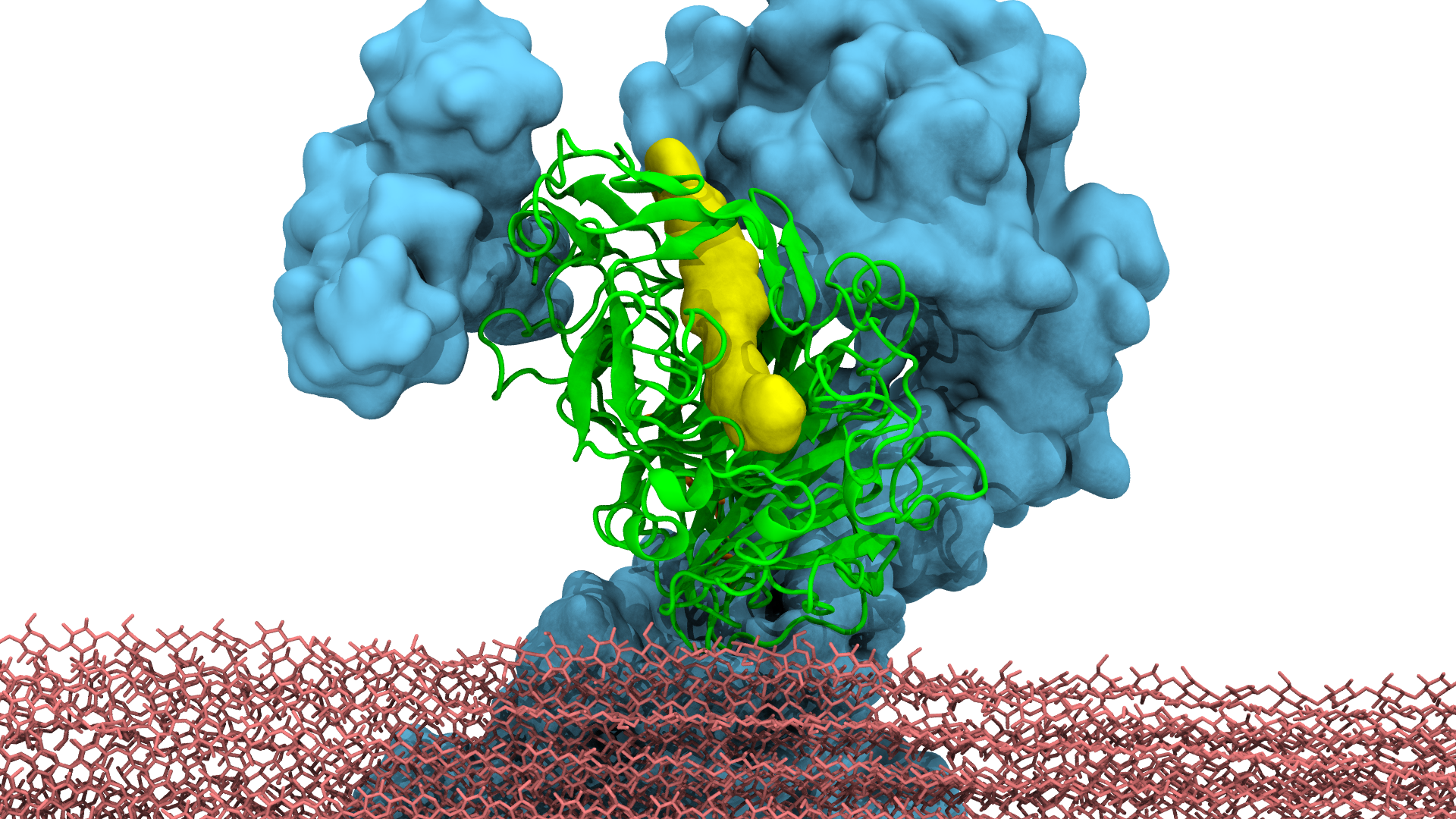

Supplement: Supplementary file 11 — 10.1186/s13068-015-0379-8 A zip archive containing a gallery of each of the cellulases that bound to cellulose in the context of their environment. Each image within the gallery is one snapshot taken from the end of the trajectory showing the relative position of each enzyme (green) that makes contact with the cellulose (red). Nearby lignins are shown in blue, and the substrate tunnel is a yellow surface to orient the viewer. The three tyrosine residues are shown in orange. Note that for each protein, there are 4 images, taken from different relative orientations to the cellulose fibril (0, 90, 180, and 270), and are labeled accordingly in their filenames. [file 13068_2015_379_MOESM11_ESM.zip › gallery/C-4_P-45_0.png]

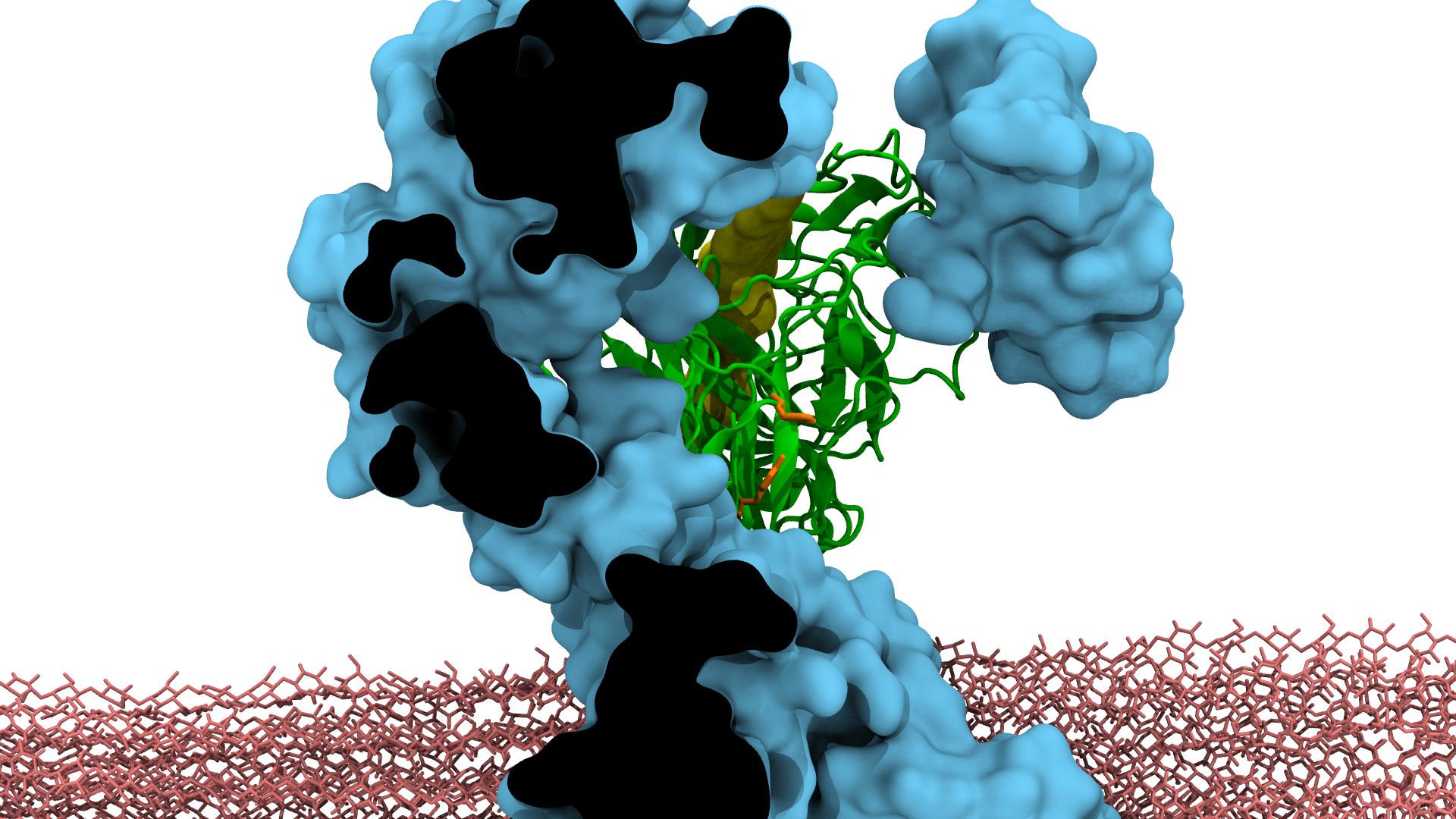

Supplement: Supplementary file 11 — 10.1186/s13068-015-0379-8 A zip archive containing a gallery of each of the cellulases that bound to cellulose in the context of their environment. Each image within the gallery is one snapshot taken from the end of the trajectory showing the relative position of each enzyme (green) that makes contact with the cellulose (red). Nearby lignins are shown in blue, and the substrate tunnel is a yellow surface to orient the viewer. The three tyrosine residues are shown in orange. Note that for each protein, there are 4 images, taken from different relative orientations to the cellulose fibril (0, 90, 180, and 270), and are labeled accordingly in their filenames. [file 13068_2015_379_MOESM11_ESM.zip › gallery/C-4_P-45_180.png]

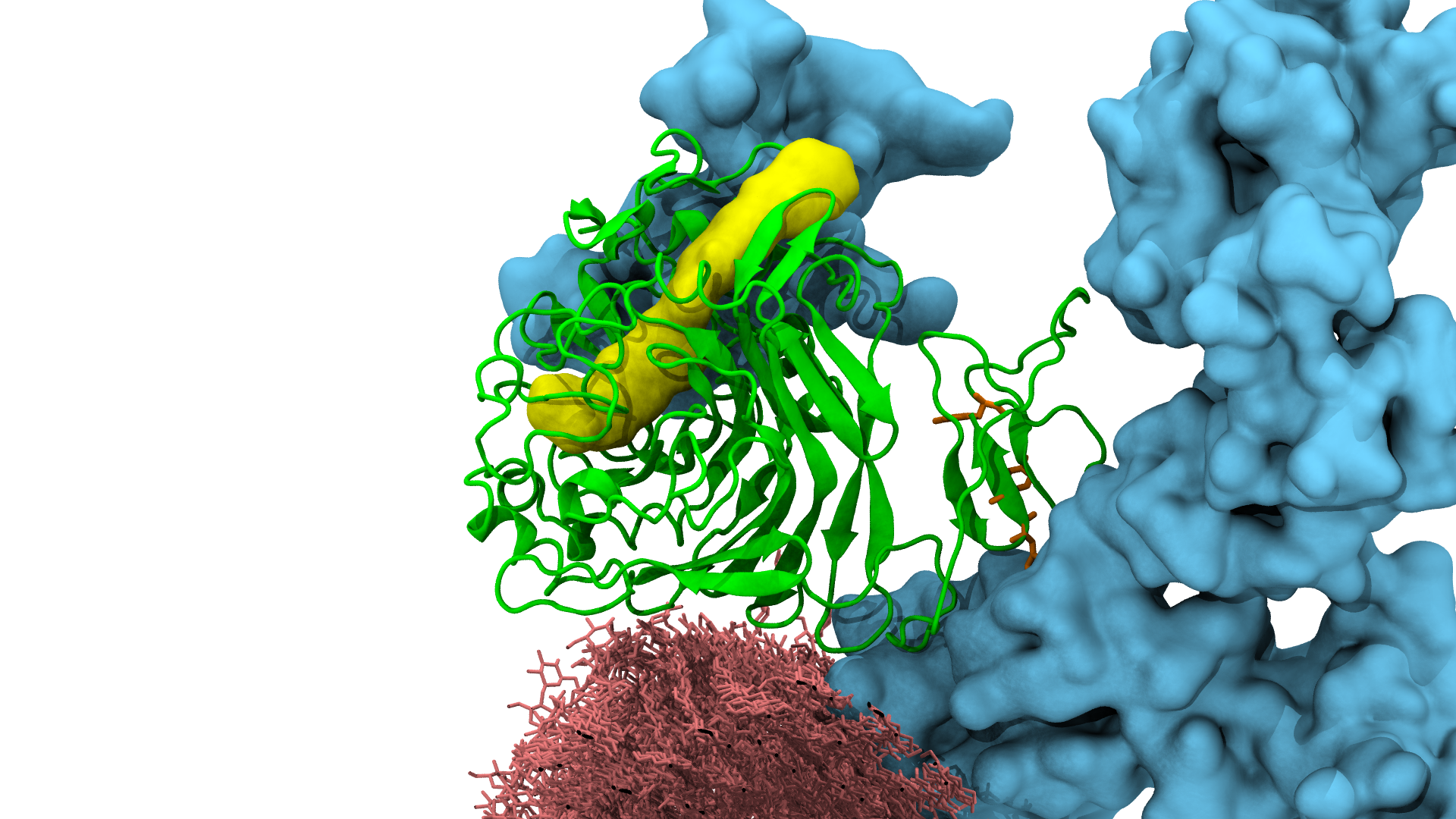

Supplement: Supplementary file 11 — 10.1186/s13068-015-0379-8 A zip archive containing a gallery of each of the cellulases that bound to cellulose in the context of their environment. Each image within the gallery is one snapshot taken from the end of the trajectory showing the relative position of each enzyme (green) that makes contact with the cellulose (red). Nearby lignins are shown in blue, and the substrate tunnel is a yellow surface to orient the viewer. The three tyrosine residues are shown in orange. Note that for each protein, there are 4 images, taken from different relative orientations to the cellulose fibril (0, 90, 180, and 270), and are labeled accordingly in their filenames. [file 13068_2015_379_MOESM11_ESM.zip › gallery/C-4_P-45_270.png]

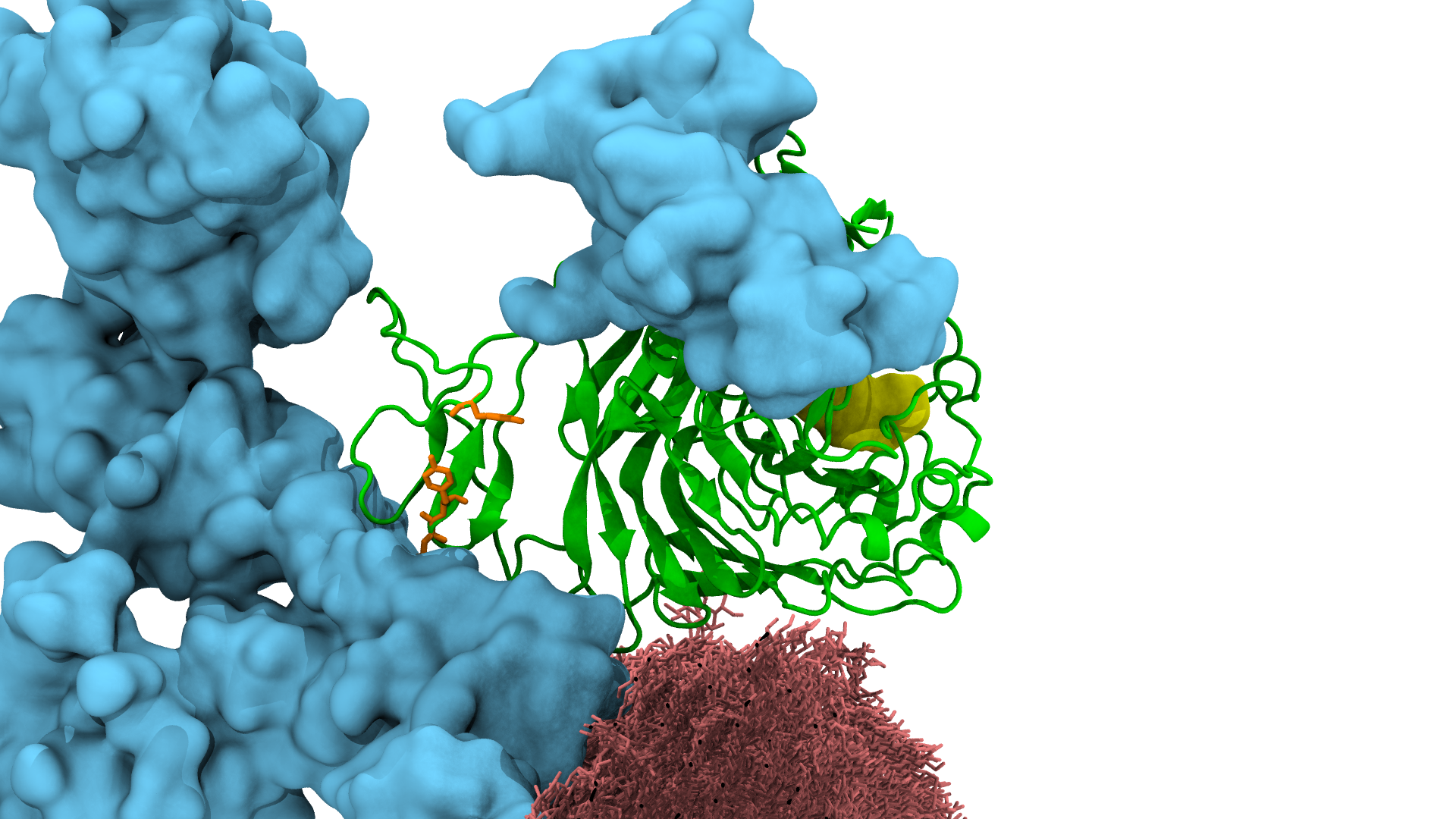

Supplement: Supplementary file 11 — 10.1186/s13068-015-0379-8 A zip archive containing a gallery of each of the cellulases that bound to cellulose in the context of their environment. Each image within the gallery is one snapshot taken from the end of the trajectory showing the relative position of each enzyme (green) that makes contact with the cellulose (red). Nearby lignins are shown in blue, and the substrate tunnel is a yellow surface to orient the viewer. The three tyrosine residues are shown in orange. Note that for each protein, there are 4 images, taken from different relative orientations to the cellulose fibril (0, 90, 180, and 270), and are labeled accordingly in their filenames. [file 13068_2015_379_MOESM11_ESM.zip › gallery/C-4_P-45_90.png]

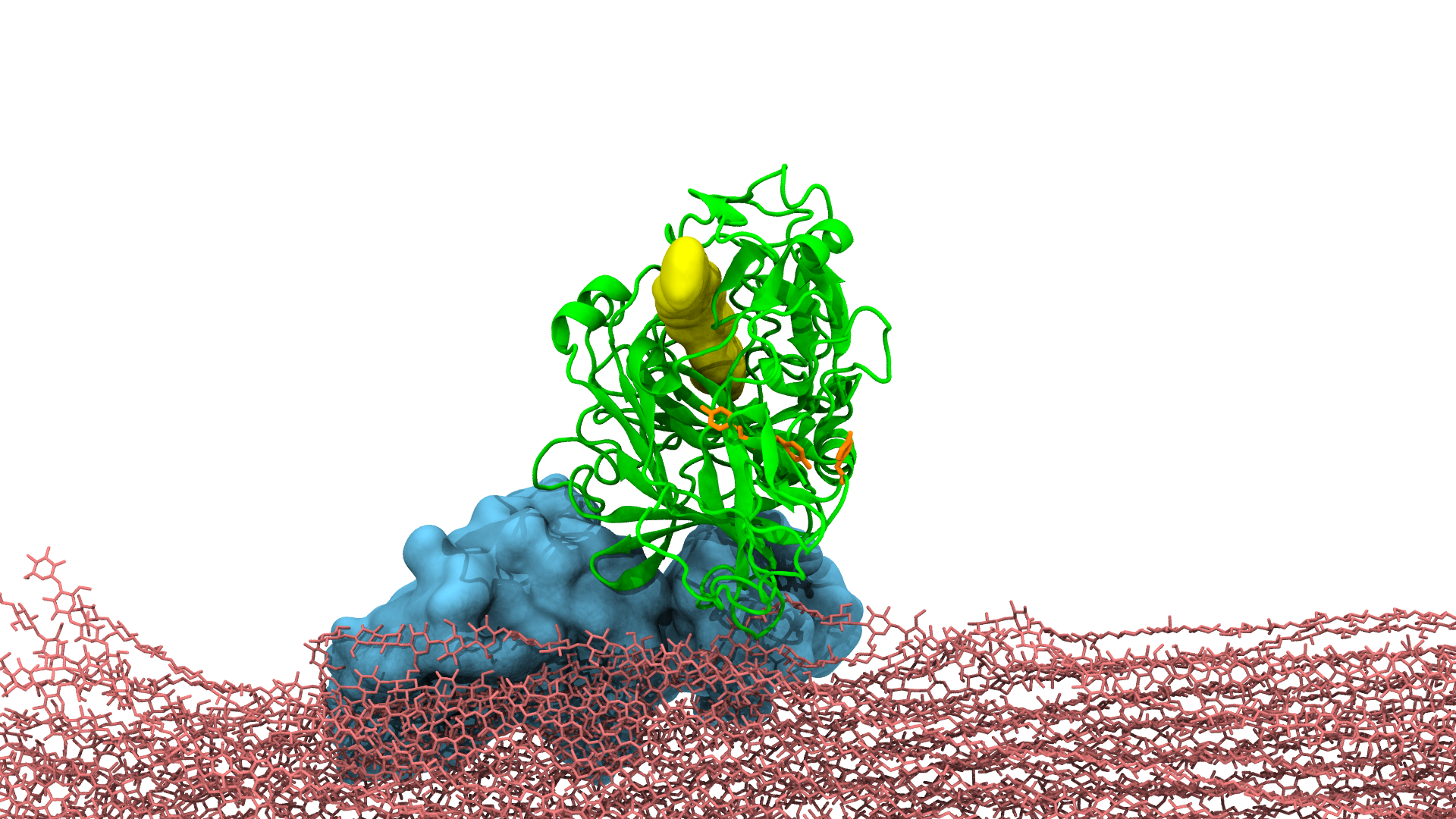

Supplement: Supplementary file 11 — 10.1186/s13068-015-0379-8 A zip archive containing a gallery of each of the cellulases that bound to cellulose in the context of their environment. Each image within the gallery is one snapshot taken from the end of the trajectory showing the relative position of each enzyme (green) that makes contact with the cellulose (red). Nearby lignins are shown in blue, and the substrate tunnel is a yellow surface to orient the viewer. The three tyrosine residues are shown in orange. Note that for each protein, there are 4 images, taken from different relative orientations to the cellulose fibril (0, 90, 180, and 270), and are labeled accordingly in their filenames. [file 13068_2015_379_MOESM11_ESM.zip › gallery/C-5_P-27_0.png]

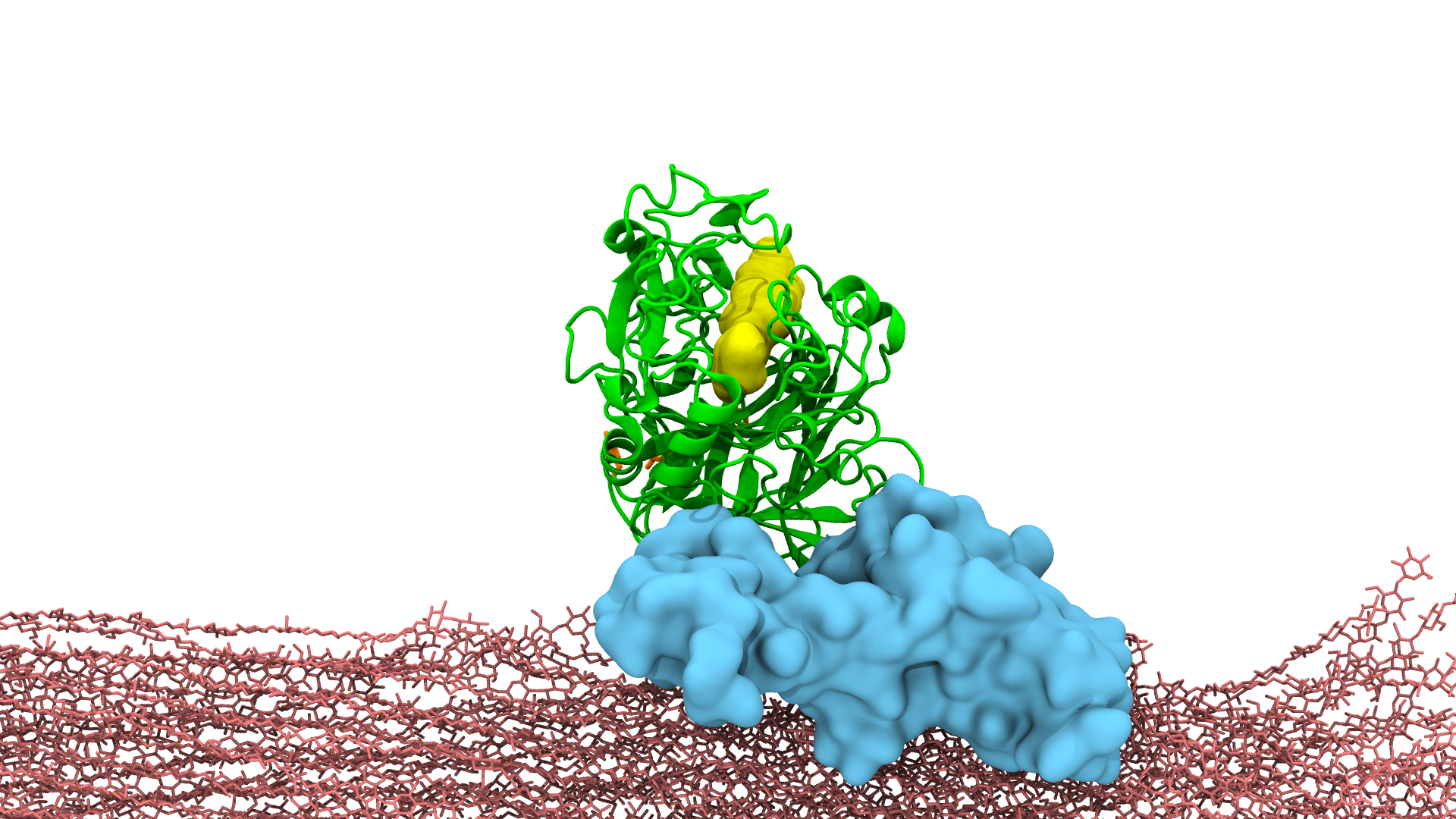

Supplement: Supplementary file 11 — 10.1186/s13068-015-0379-8 A zip archive containing a gallery of each of the cellulases that bound to cellulose in the context of their environment. Each image within the gallery is one snapshot taken from the end of the trajectory showing the relative position of each enzyme (green) that makes contact with the cellulose (red). Nearby lignins are shown in blue, and the substrate tunnel is a yellow surface to orient the viewer. The three tyrosine residues are shown in orange. Note that for each protein, there are 4 images, taken from different relative orientations to the cellulose fibril (0, 90, 180, and 270), and are labeled accordingly in their filenames. [file 13068_2015_379_MOESM11_ESM.zip › gallery/C-5_P-27_180.png]

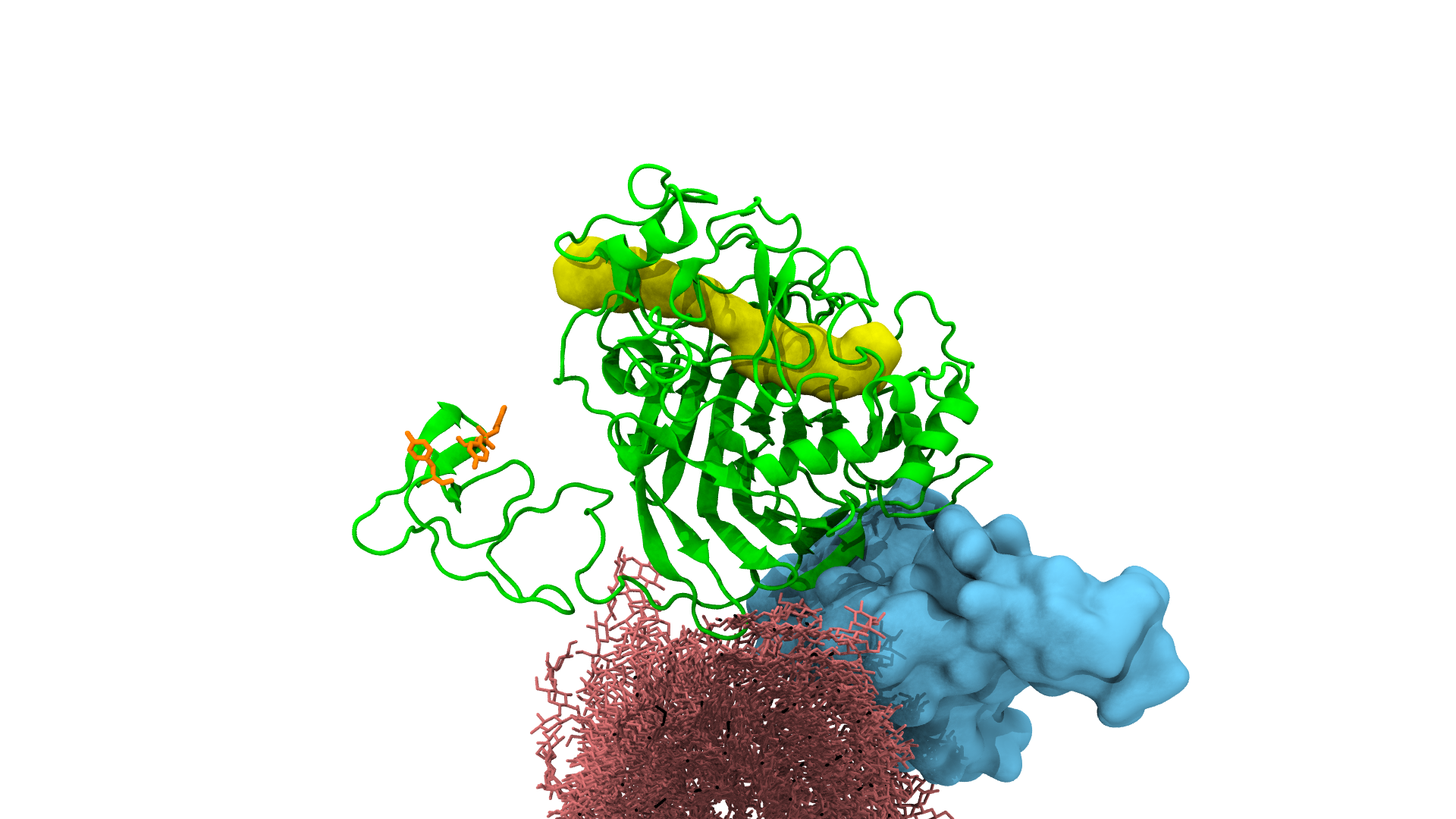

Supplement: Supplementary file 11 — 10.1186/s13068-015-0379-8 A zip archive containing a gallery of each of the cellulases that bound to cellulose in the context of their environment. Each image within the gallery is one snapshot taken from the end of the trajectory showing the relative position of each enzyme (green) that makes contact with the cellulose (red). Nearby lignins are shown in blue, and the substrate tunnel is a yellow surface to orient the viewer. The three tyrosine residues are shown in orange. Note that for each protein, there are 4 images, taken from different relative orientations to the cellulose fibril (0, 90, 180, and 270), and are labeled accordingly in their filenames. [file 13068_2015_379_MOESM11_ESM.zip › gallery/C-5_P-27_270.png]

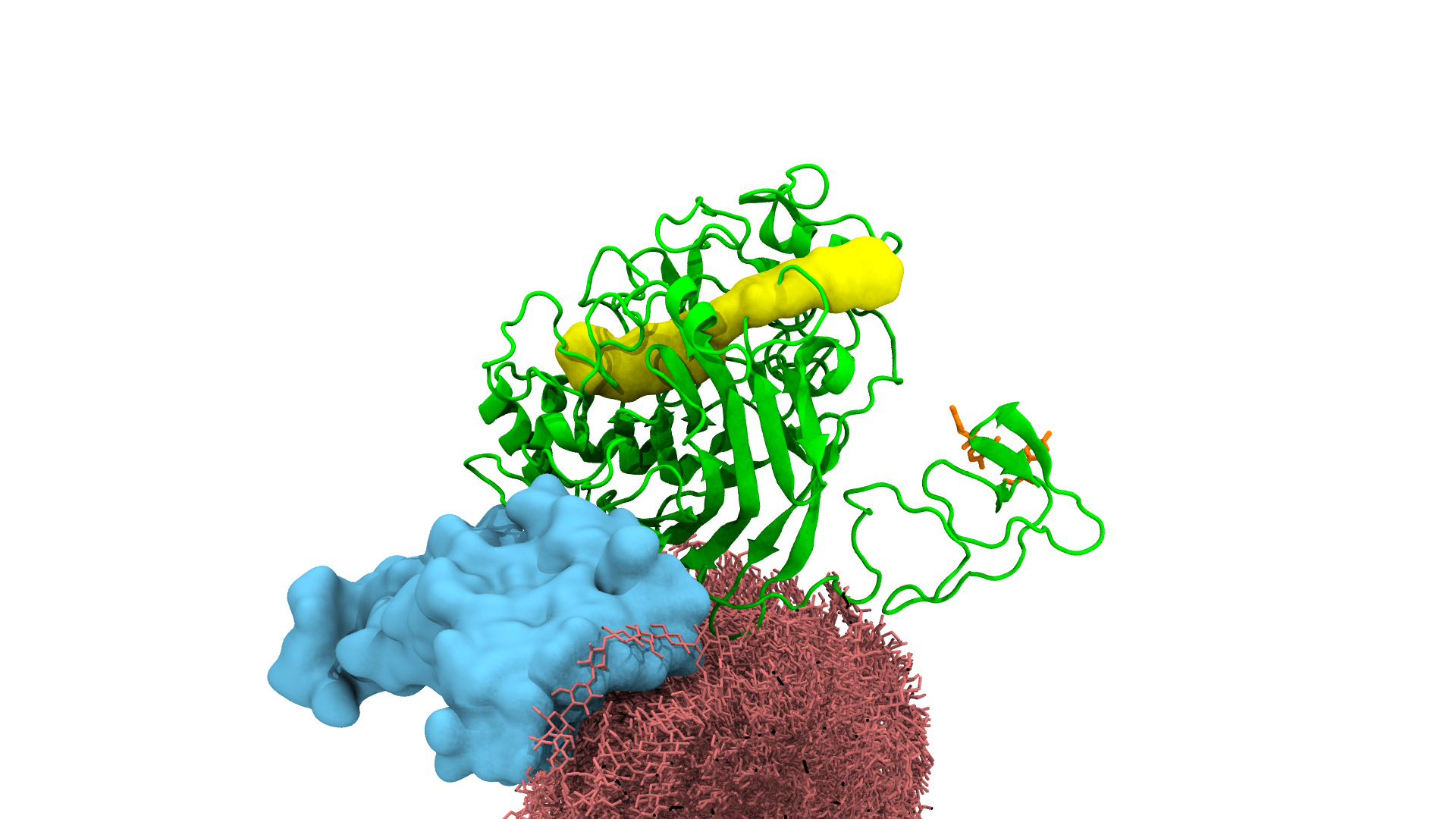

Supplement: Supplementary file 11 — 10.1186/s13068-015-0379-8 A zip archive containing a gallery of each of the cellulases that bound to cellulose in the context of their environment. Each image within the gallery is one snapshot taken from the end of the trajectory showing the relative position of each enzyme (green) that makes contact with the cellulose (red). Nearby lignins are shown in blue, and the substrate tunnel is a yellow surface to orient the viewer. The three tyrosine residues are shown in orange. Note that for each protein, there are 4 images, taken from different relative orientations to the cellulose fibril (0, 90, 180, and 270), and are labeled accordingly in their filenames. [file 13068_2015_379_MOESM11_ESM.zip › gallery/C-5_P-27_90.png]

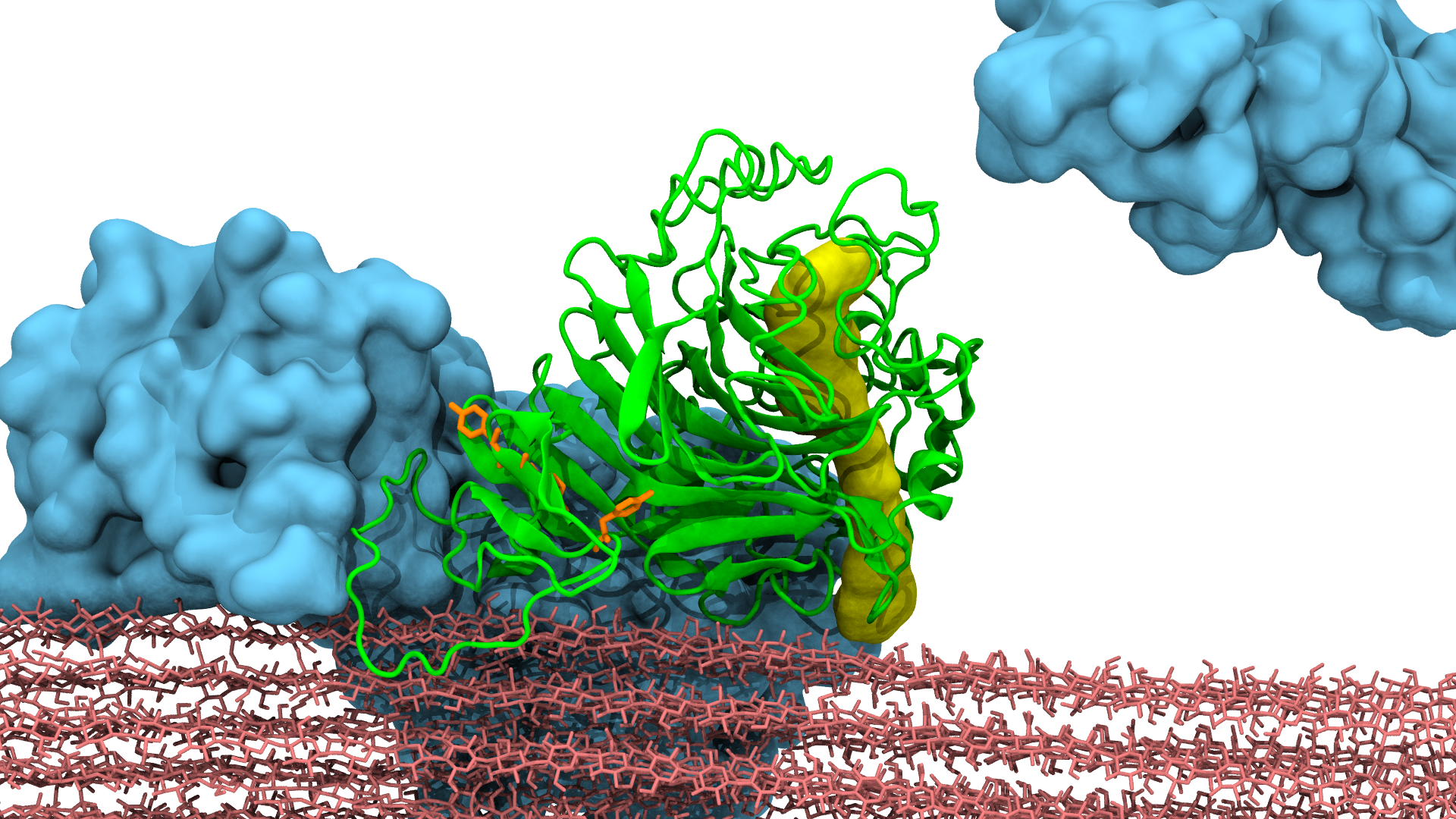

Supplement: Supplementary file 11 — 10.1186/s13068-015-0379-8 A zip archive containing a gallery of each of the cellulases that bound to cellulose in the context of their environment. Each image within the gallery is one snapshot taken from the end of the trajectory showing the relative position of each enzyme (green) that makes contact with the cellulose (red). Nearby lignins are shown in blue, and the substrate tunnel is a yellow surface to orient the viewer. The three tyrosine residues are shown in orange. Note that for each protein, there are 4 images, taken from different relative orientations to the cellulose fibril (0, 90, 180, and 270), and are labeled accordingly in their filenames. [file 13068_2015_379_MOESM11_ESM.zip › gallery/C-5_P-44_0.png]

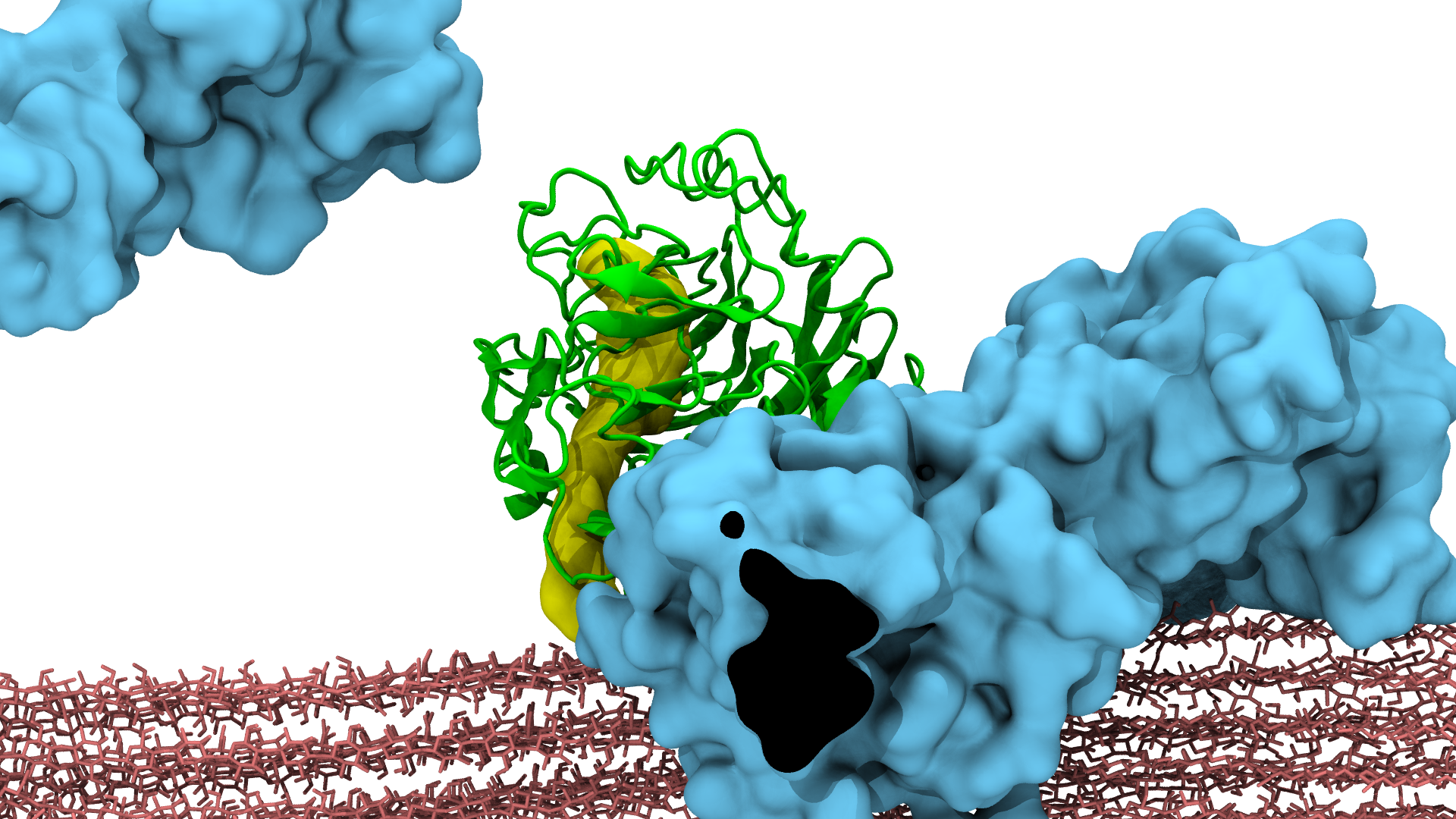

Supplement: Supplementary file 11 — 10.1186/s13068-015-0379-8 A zip archive containing a gallery of each of the cellulases that bound to cellulose in the context of their environment. Each image within the gallery is one snapshot taken from the end of the trajectory showing the relative position of each enzyme (green) that makes contact with the cellulose (red). Nearby lignins are shown in blue, and the substrate tunnel is a yellow surface to orient the viewer. The three tyrosine residues are shown in orange. Note that for each protein, there are 4 images, taken from different relative orientations to the cellulose fibril (0, 90, 180, and 270), and are labeled accordingly in their filenames. [file 13068_2015_379_MOESM11_ESM.zip › gallery/C-5_P-44_180.png]

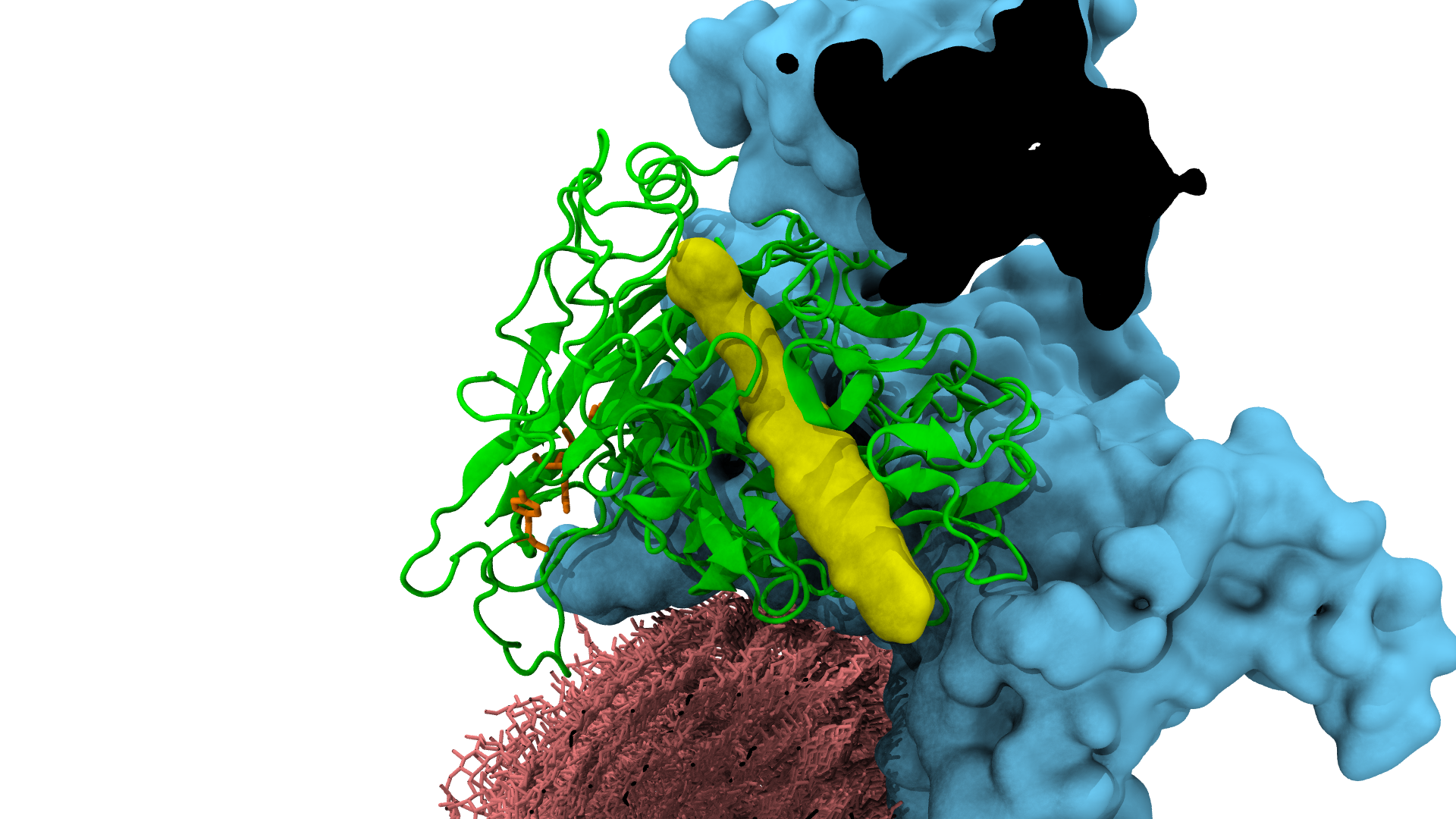

Supplement: Supplementary file 11 — 10.1186/s13068-015-0379-8 A zip archive containing a gallery of each of the cellulases that bound to cellulose in the context of their environment. Each image within the gallery is one snapshot taken from the end of the trajectory showing the relative position of each enzyme (green) that makes contact with the cellulose (red). Nearby lignins are shown in blue, and the substrate tunnel is a yellow surface to orient the viewer. The three tyrosine residues are shown in orange. Note that for each protein, there are 4 images, taken from different relative orientations to the cellulose fibril (0, 90, 180, and 270), and are labeled accordingly in their filenames. [file 13068_2015_379_MOESM11_ESM.zip › gallery/C-5_P-44_270.png]

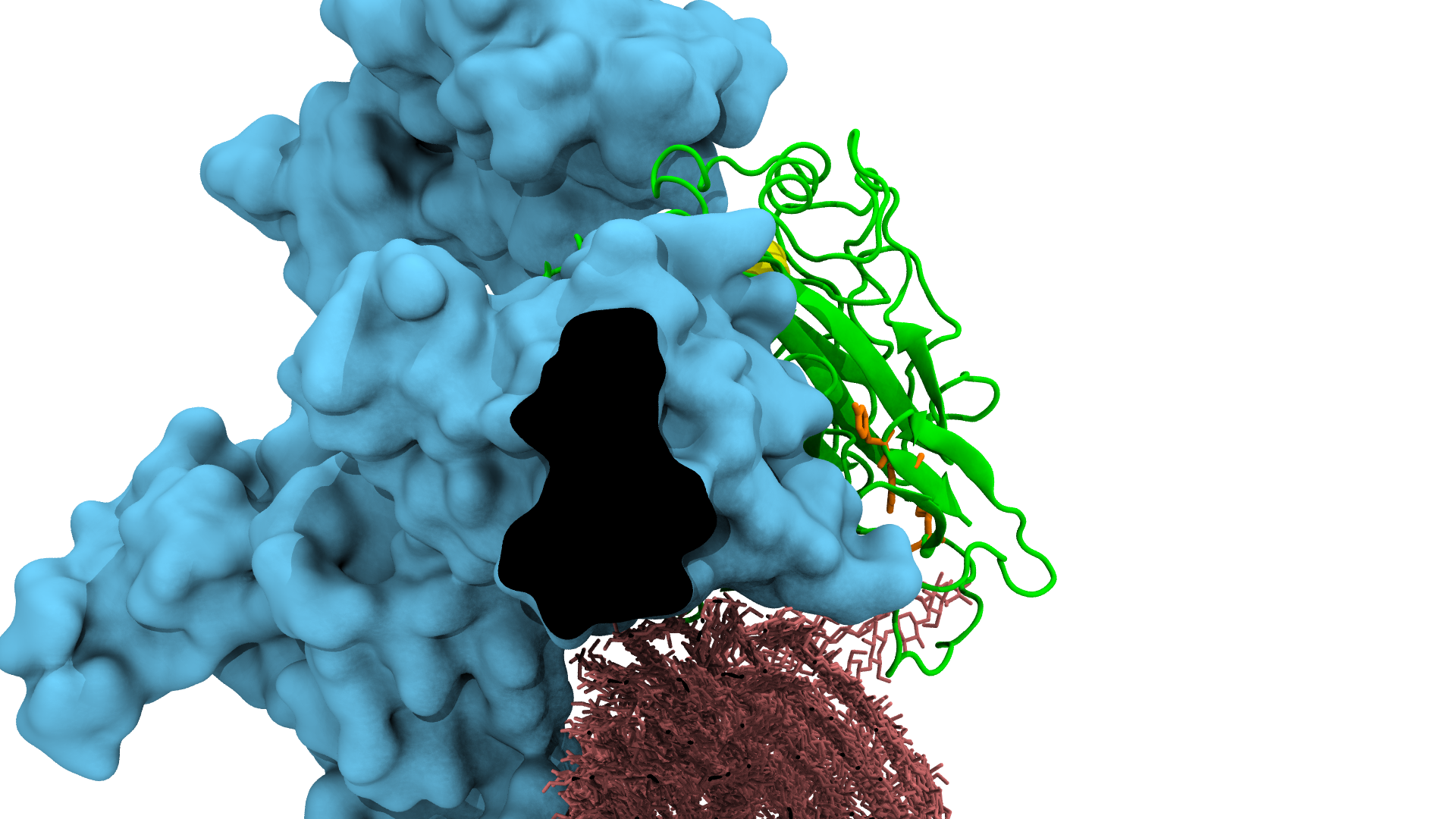

Supplement: Supplementary file 11 — 10.1186/s13068-015-0379-8 A zip archive containing a gallery of each of the cellulases that bound to cellulose in the context of their environment. Each image within the gallery is one snapshot taken from the end of the trajectory showing the relative position of each enzyme (green) that makes contact with the cellulose (red). Nearby lignins are shown in blue, and the substrate tunnel is a yellow surface to orient the viewer. The three tyrosine residues are shown in orange. Note that for each protein, there are 4 images, taken from different relative orientations to the cellulose fibril (0, 90, 180, and 270), and are labeled accordingly in their filenames. [file 13068_2015_379_MOESM11_ESM.zip › gallery/C-5_P-44_90.png]

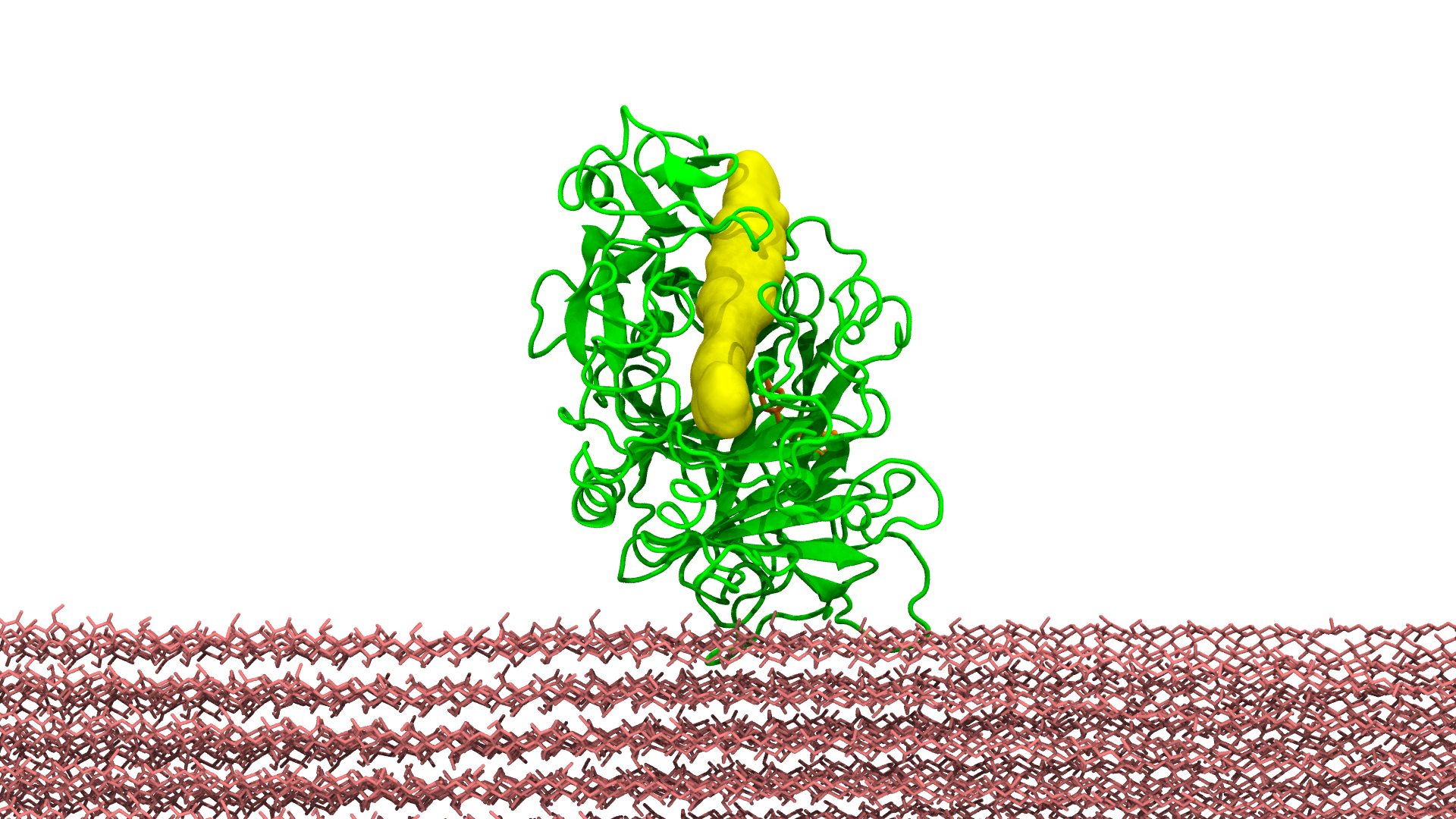

Supplement: Supplementary file 11 — 10.1186/s13068-015-0379-8 A zip archive containing a gallery of each of the cellulases that bound to cellulose in the context of their environment. Each image within the gallery is one snapshot taken from the end of the trajectory showing the relative position of each enzyme (green) that makes contact with the cellulose (red). Nearby lignins are shown in blue, and the substrate tunnel is a yellow surface to orient the viewer. The three tyrosine residues are shown in orange. Note that for each protein, there are 4 images, taken from different relative orientations to the cellulose fibril (0, 90, 180, and 270), and are labeled accordingly in their filenames. [file 13068_2015_379_MOESM11_ESM.zip › gallery/C-6_P-17_0.png]

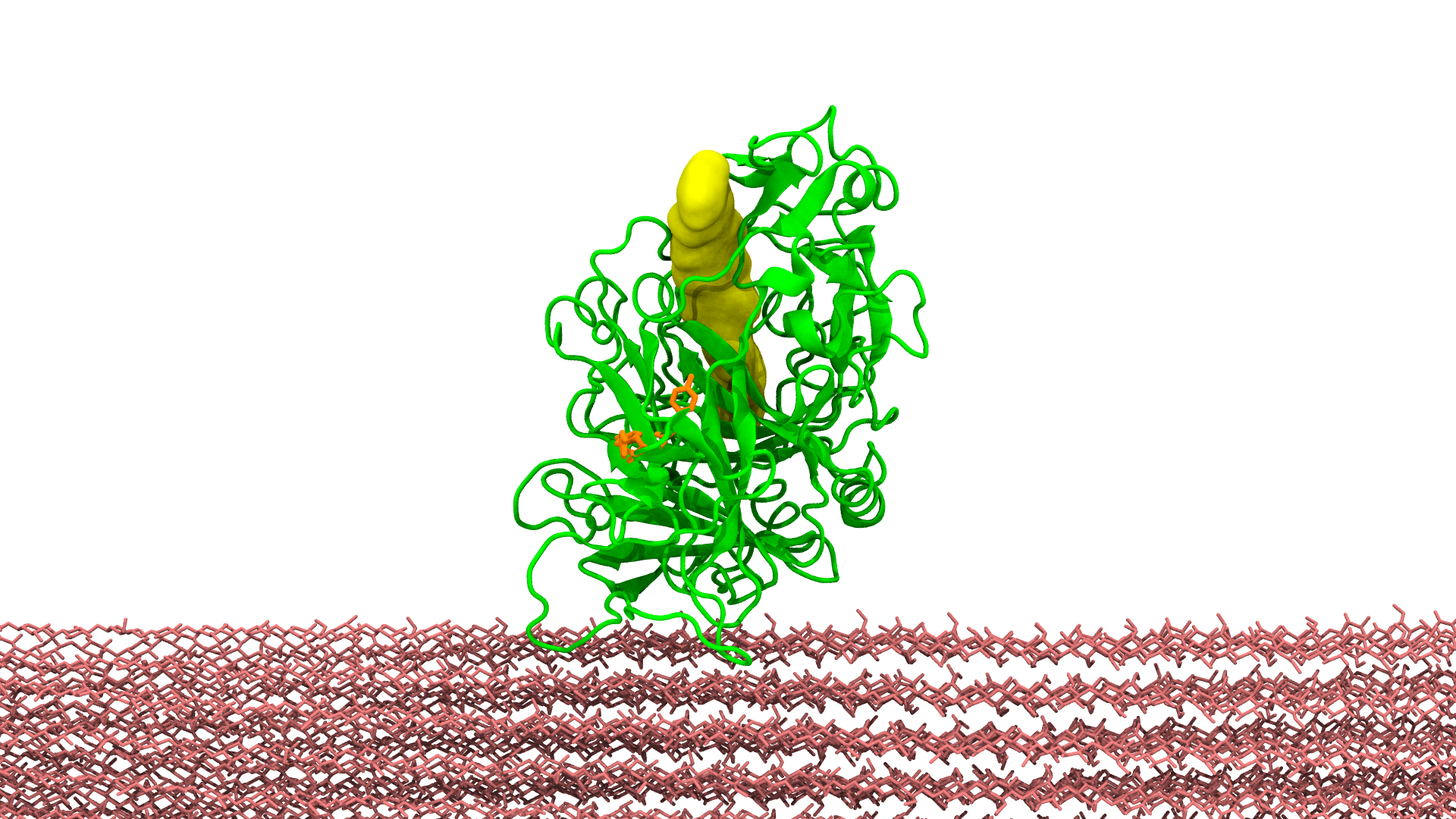

Supplement: Supplementary file 11 — 10.1186/s13068-015-0379-8 A zip archive containing a gallery of each of the cellulases that bound to cellulose in the context of their environment. Each image within the gallery is one snapshot taken from the end of the trajectory showing the relative position of each enzyme (green) that makes contact with the cellulose (red). Nearby lignins are shown in blue, and the substrate tunnel is a yellow surface to orient the viewer. The three tyrosine residues are shown in orange. Note that for each protein, there are 4 images, taken from different relative orientations to the cellulose fibril (0, 90, 180, and 270), and are labeled accordingly in their filenames. [file 13068_2015_379_MOESM11_ESM.zip › gallery/C-6_P-17_180.png]

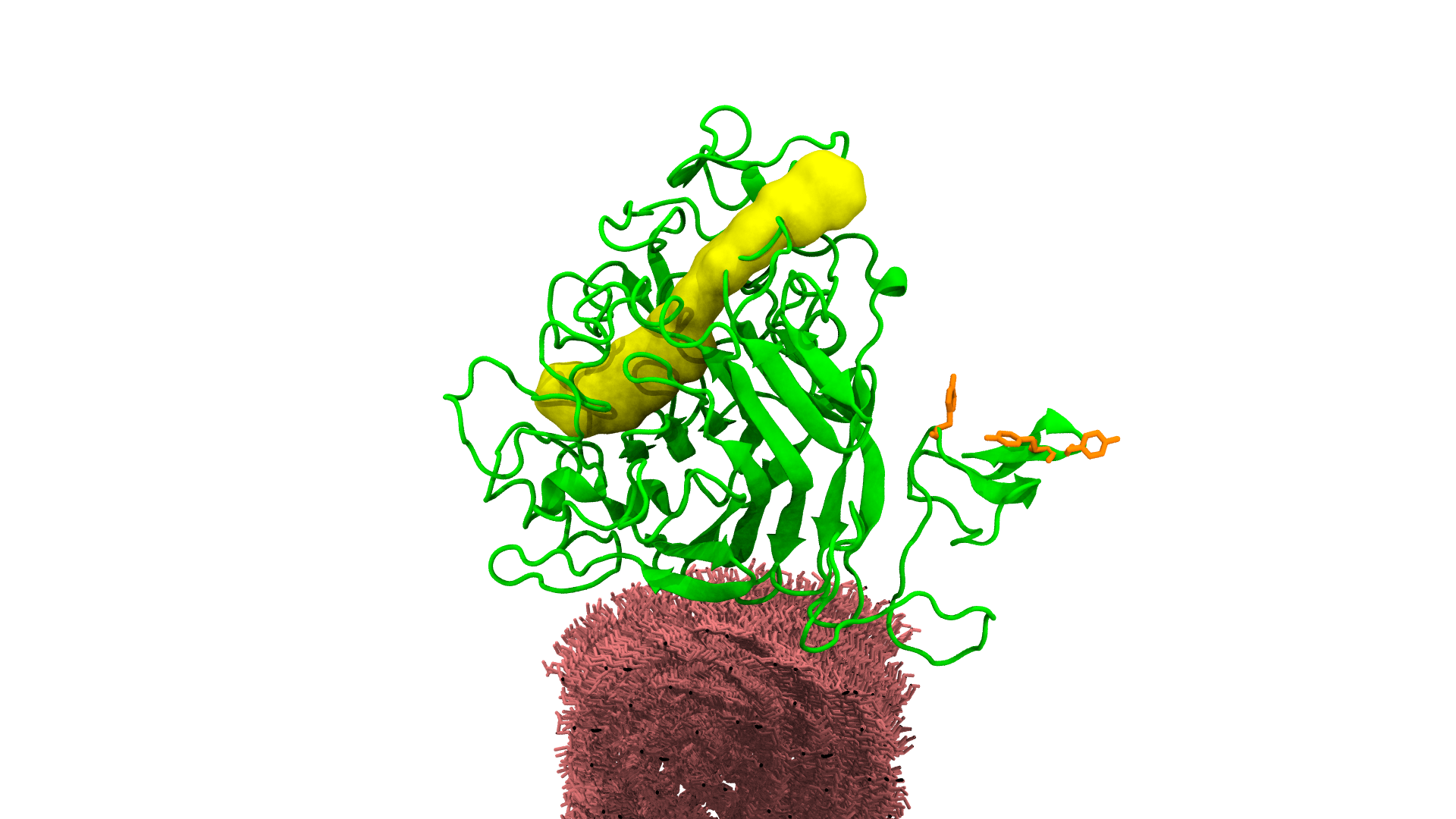

Supplement: Supplementary file 11 — 10.1186/s13068-015-0379-8 A zip archive containing a gallery of each of the cellulases that bound to cellulose in the context of their environment. Each image within the gallery is one snapshot taken from the end of the trajectory showing the relative position of each enzyme (green) that makes contact with the cellulose (red). Nearby lignins are shown in blue, and the substrate tunnel is a yellow surface to orient the viewer. The three tyrosine residues are shown in orange. Note that for each protein, there are 4 images, taken from different relative orientations to the cellulose fibril (0, 90, 180, and 270), and are labeled accordingly in their filenames. [file 13068_2015_379_MOESM11_ESM.zip › gallery/C-6_P-17_270.png]

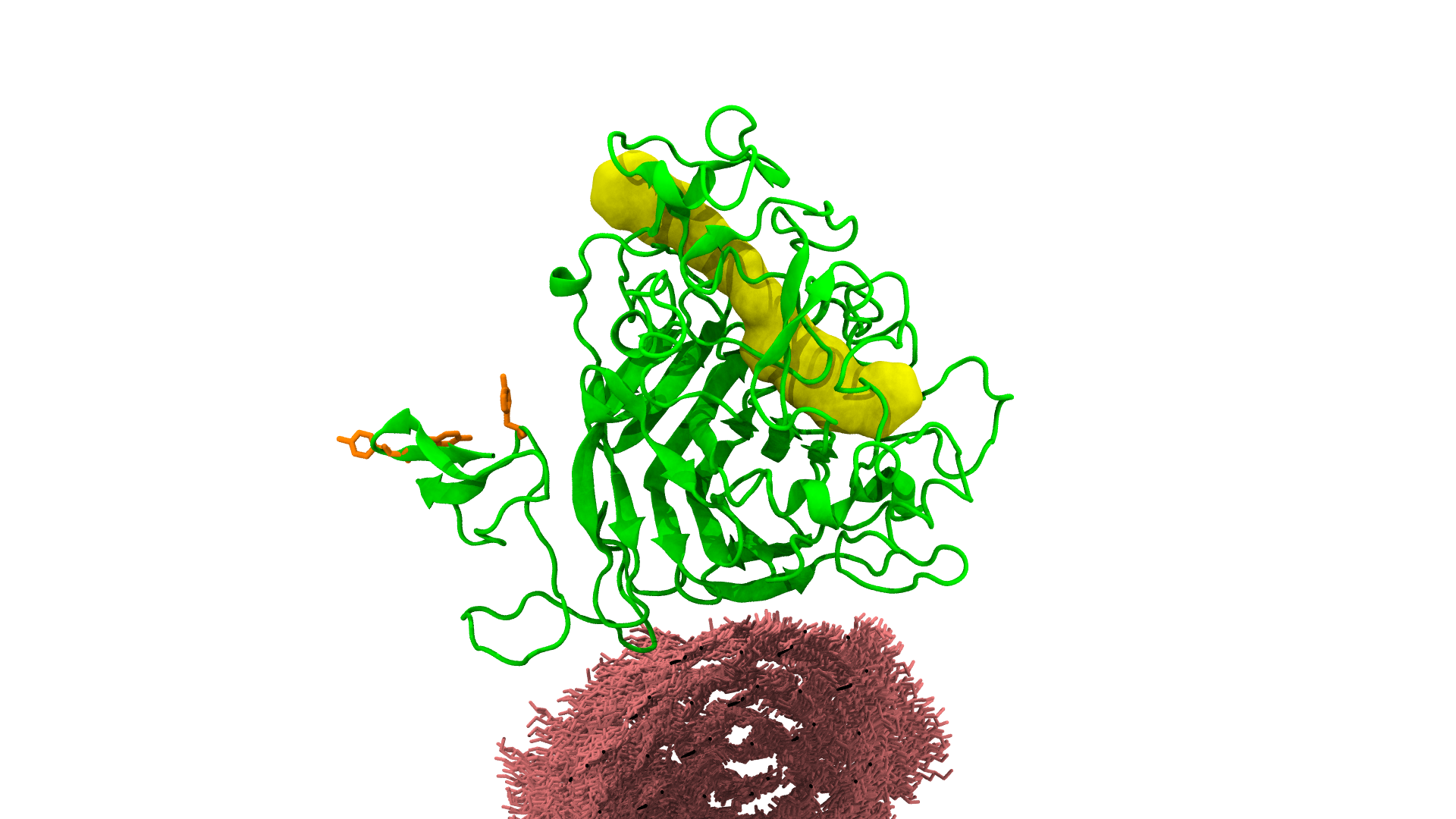

Supplement: Supplementary file 11 — 10.1186/s13068-015-0379-8 A zip archive containing a gallery of each of the cellulases that bound to cellulose in the context of their environment. Each image within the gallery is one snapshot taken from the end of the trajectory showing the relative position of each enzyme (green) that makes contact with the cellulose (red). Nearby lignins are shown in blue, and the substrate tunnel is a yellow surface to orient the viewer. The three tyrosine residues are shown in orange. Note that for each protein, there are 4 images, taken from different relative orientations to the cellulose fibril (0, 90, 180, and 270), and are labeled accordingly in their filenames. [file 13068_2015_379_MOESM11_ESM.zip › gallery/C-6_P-17_90.png]

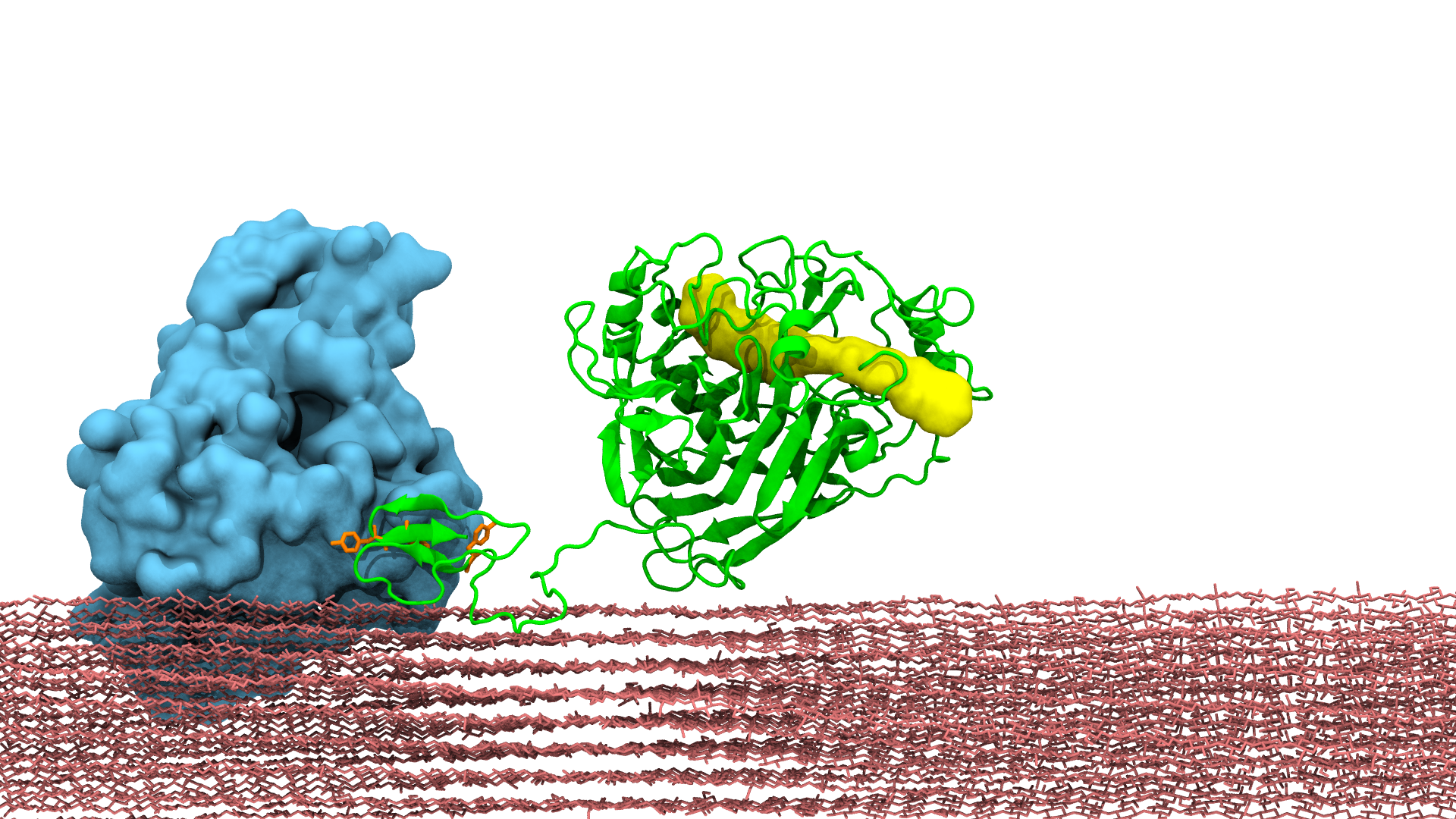

Supplement: Supplementary file 11 — 10.1186/s13068-015-0379-8 A zip archive containing a gallery of each of the cellulases that bound to cellulose in the context of their environment. Each image within the gallery is one snapshot taken from the end of the trajectory showing the relative position of each enzyme (green) that makes contact with the cellulose (red). Nearby lignins are shown in blue, and the substrate tunnel is a yellow surface to orient the viewer. The three tyrosine residues are shown in orange. Note that for each protein, there are 4 images, taken from different relative orientations to the cellulose fibril (0, 90, 180, and 270), and are labeled accordingly in their filenames. [file 13068_2015_379_MOESM11_ESM.zip › gallery/C-6_P-26_0.png]

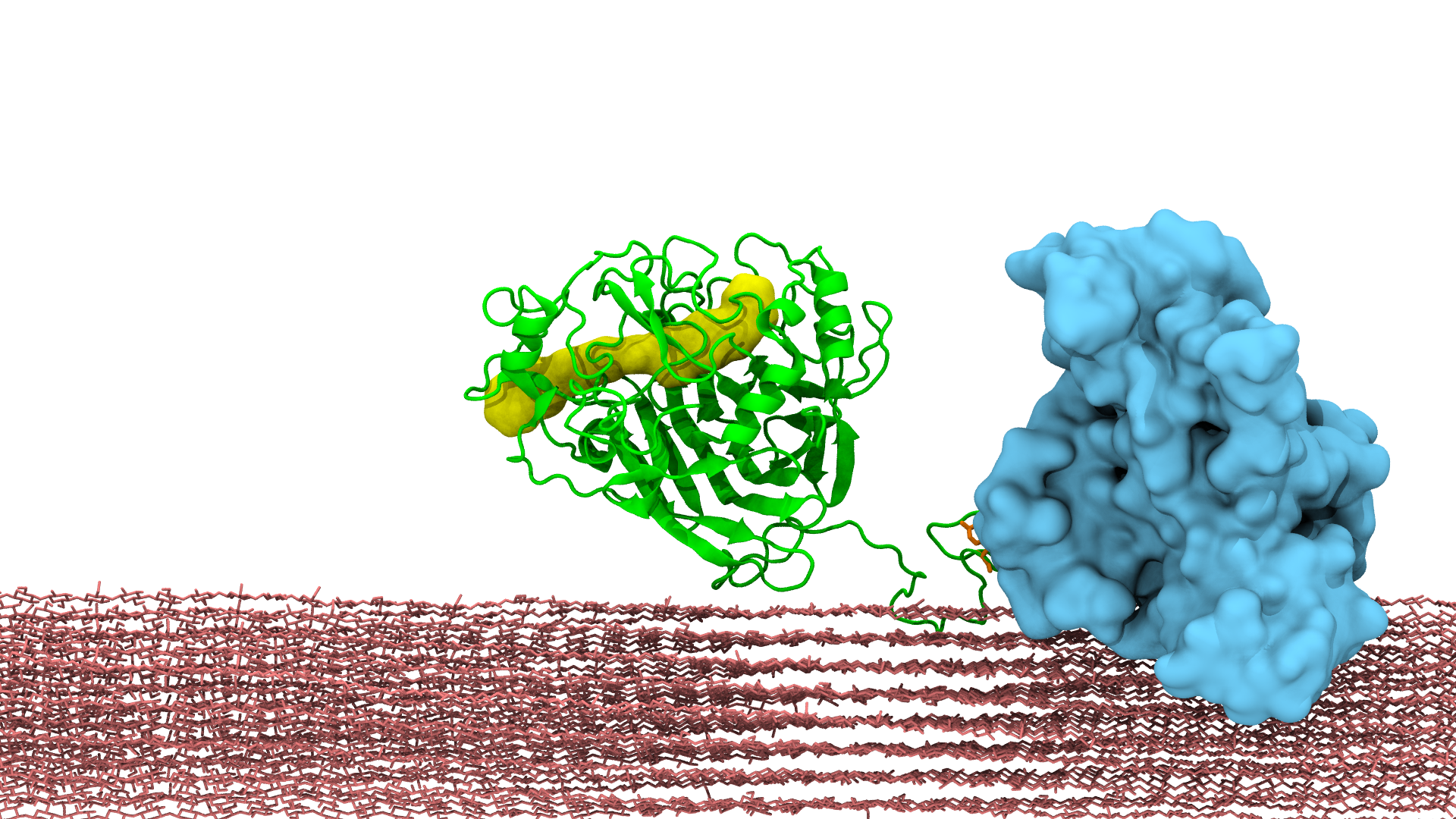

Supplement: Supplementary file 11 — 10.1186/s13068-015-0379-8 A zip archive containing a gallery of each of the cellulases that bound to cellulose in the context of their environment. Each image within the gallery is one snapshot taken from the end of the trajectory showing the relative position of each enzyme (green) that makes contact with the cellulose (red). Nearby lignins are shown in blue, and the substrate tunnel is a yellow surface to orient the viewer. The three tyrosine residues are shown in orange. Note that for each protein, there are 4 images, taken from different relative orientations to the cellulose fibril (0, 90, 180, and 270), and are labeled accordingly in their filenames. [file 13068_2015_379_MOESM11_ESM.zip › gallery/C-6_P-26_180.png]

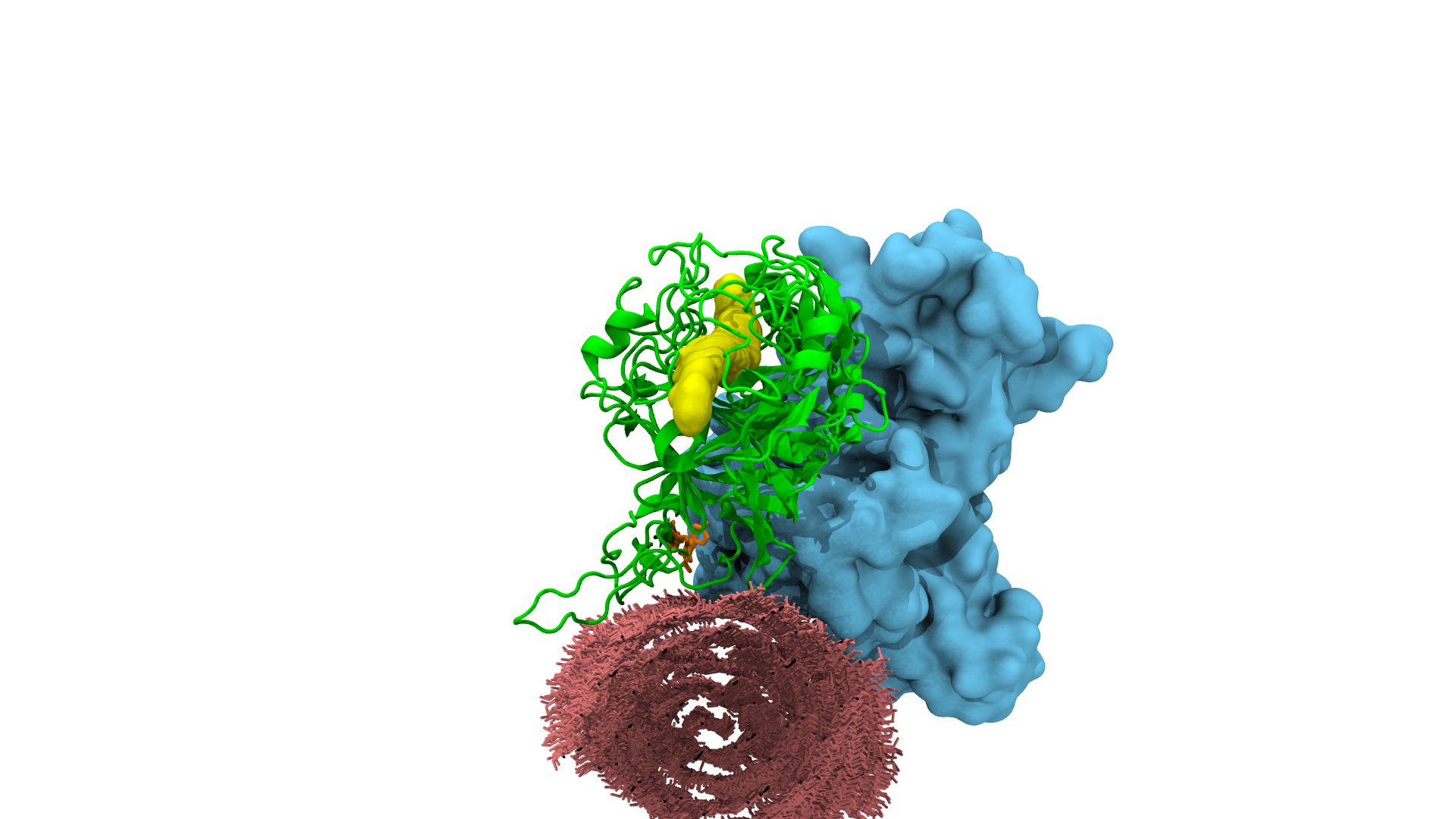

Supplement: Supplementary file 11 — 10.1186/s13068-015-0379-8 A zip archive containing a gallery of each of the cellulases that bound to cellulose in the context of their environment. Each image within the gallery is one snapshot taken from the end of the trajectory showing the relative position of each enzyme (green) that makes contact with the cellulose (red). Nearby lignins are shown in blue, and the substrate tunnel is a yellow surface to orient the viewer. The three tyrosine residues are shown in orange. Note that for each protein, there are 4 images, taken from different relative orientations to the cellulose fibril (0, 90, 180, and 270), and are labeled accordingly in their filenames. [file 13068_2015_379_MOESM11_ESM.zip › gallery/C-6_P-26_270.png]

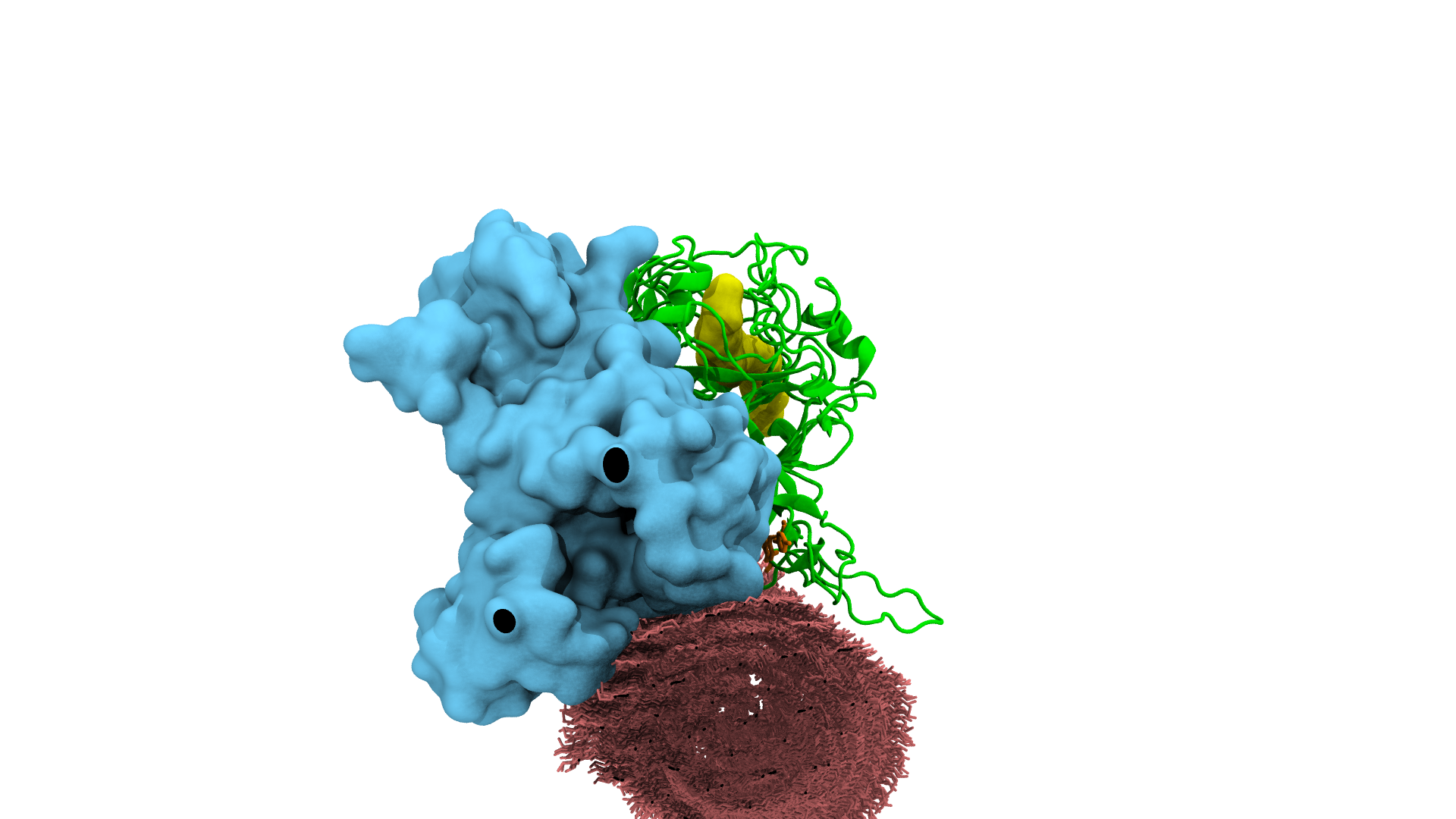

Supplement: Supplementary file 11 — 10.1186/s13068-015-0379-8 A zip archive containing a gallery of each of the cellulases that bound to cellulose in the context of their environment. Each image within the gallery is one snapshot taken from the end of the trajectory showing the relative position of each enzyme (green) that makes contact with the cellulose (red). Nearby lignins are shown in blue, and the substrate tunnel is a yellow surface to orient the viewer. The three tyrosine residues are shown in orange. Note that for each protein, there are 4 images, taken from different relative orientations to the cellulose fibril (0, 90, 180, and 270), and are labeled accordingly in their filenames. [file 13068_2015_379_MOESM11_ESM.zip › gallery/C-6_P-26_90.png]

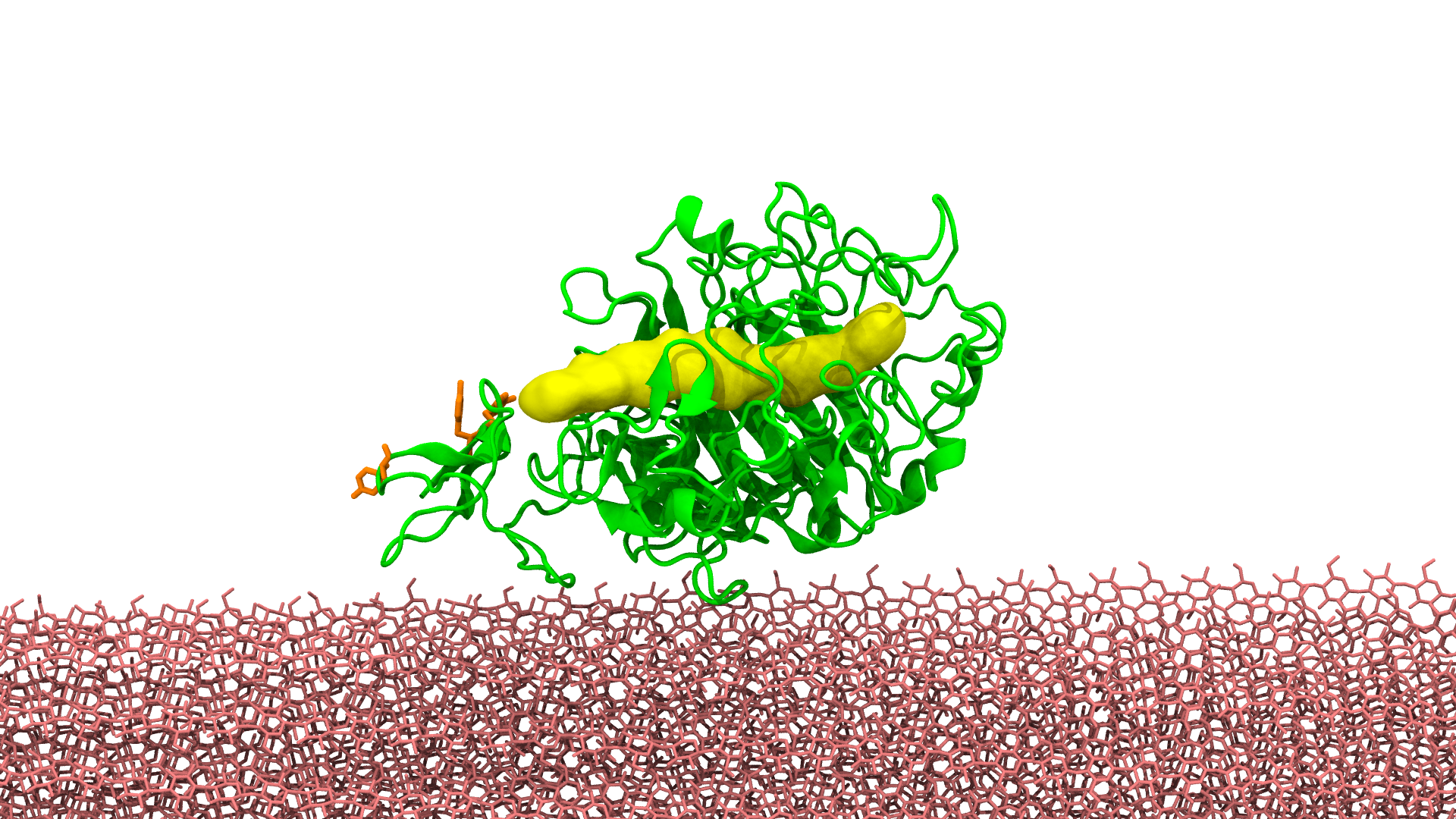

Supplement: Supplementary file 11 — 10.1186/s13068-015-0379-8 A zip archive containing a gallery of each of the cellulases that bound to cellulose in the context of their environment. Each image within the gallery is one snapshot taken from the end of the trajectory showing the relative position of each enzyme (green) that makes contact with the cellulose (red). Nearby lignins are shown in blue, and the substrate tunnel is a yellow surface to orient the viewer. The three tyrosine residues are shown in orange. Note that for each protein, there are 4 images, taken from different relative orientations to the cellulose fibril (0, 90, 180, and 270), and are labeled accordingly in their filenames. [file 13068_2015_379_MOESM11_ESM.zip › gallery/C-6_P-31_0.png]

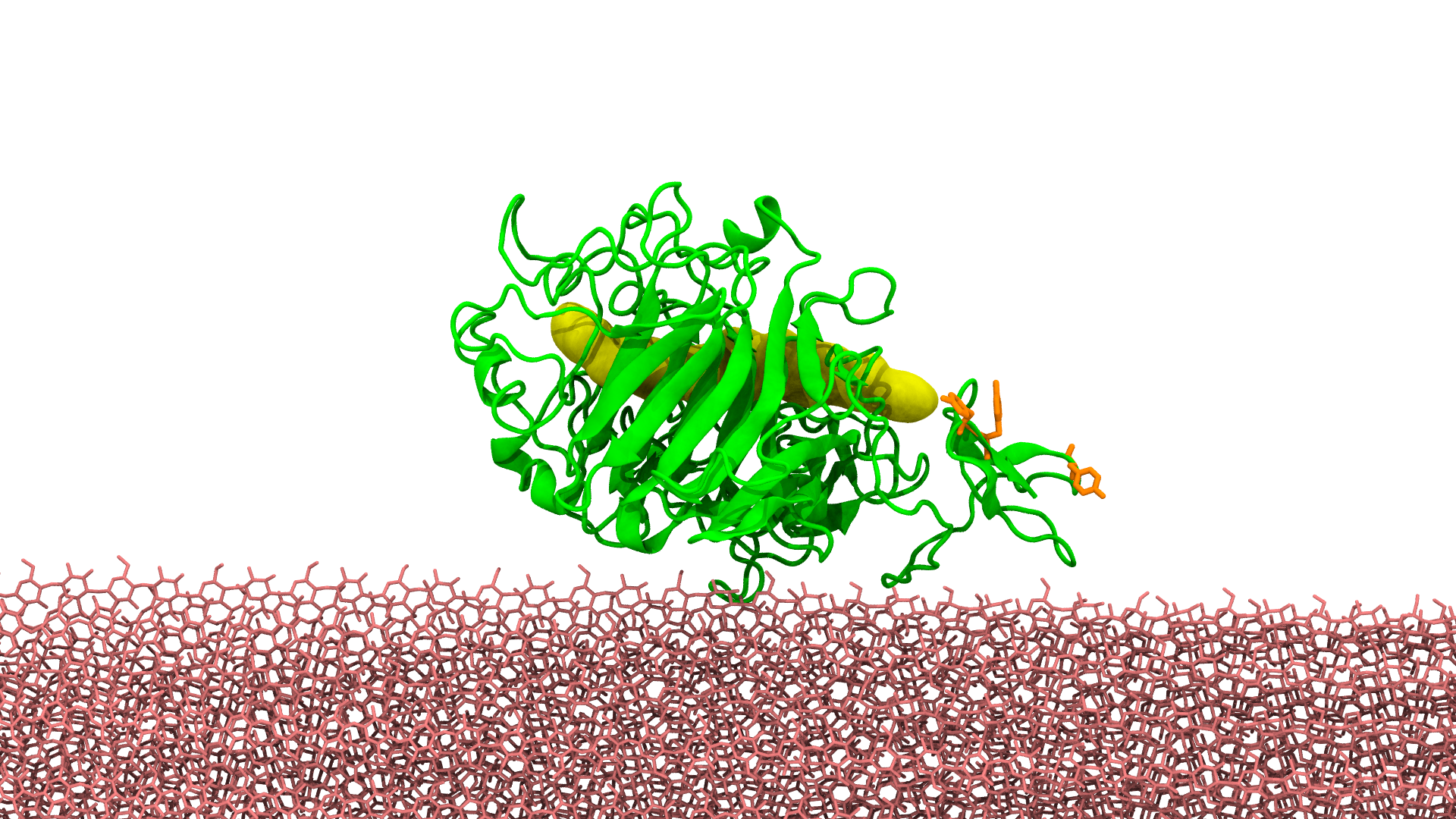

Supplement: Supplementary file 11 — 10.1186/s13068-015-0379-8 A zip archive containing a gallery of each of the cellulases that bound to cellulose in the context of their environment. Each image within the gallery is one snapshot taken from the end of the trajectory showing the relative position of each enzyme (green) that makes contact with the cellulose (red). Nearby lignins are shown in blue, and the substrate tunnel is a yellow surface to orient the viewer. The three tyrosine residues are shown in orange. Note that for each protein, there are 4 images, taken from different relative orientations to the cellulose fibril (0, 90, 180, and 270), and are labeled accordingly in their filenames. [file 13068_2015_379_MOESM11_ESM.zip › gallery/C-6_P-31_180.png]

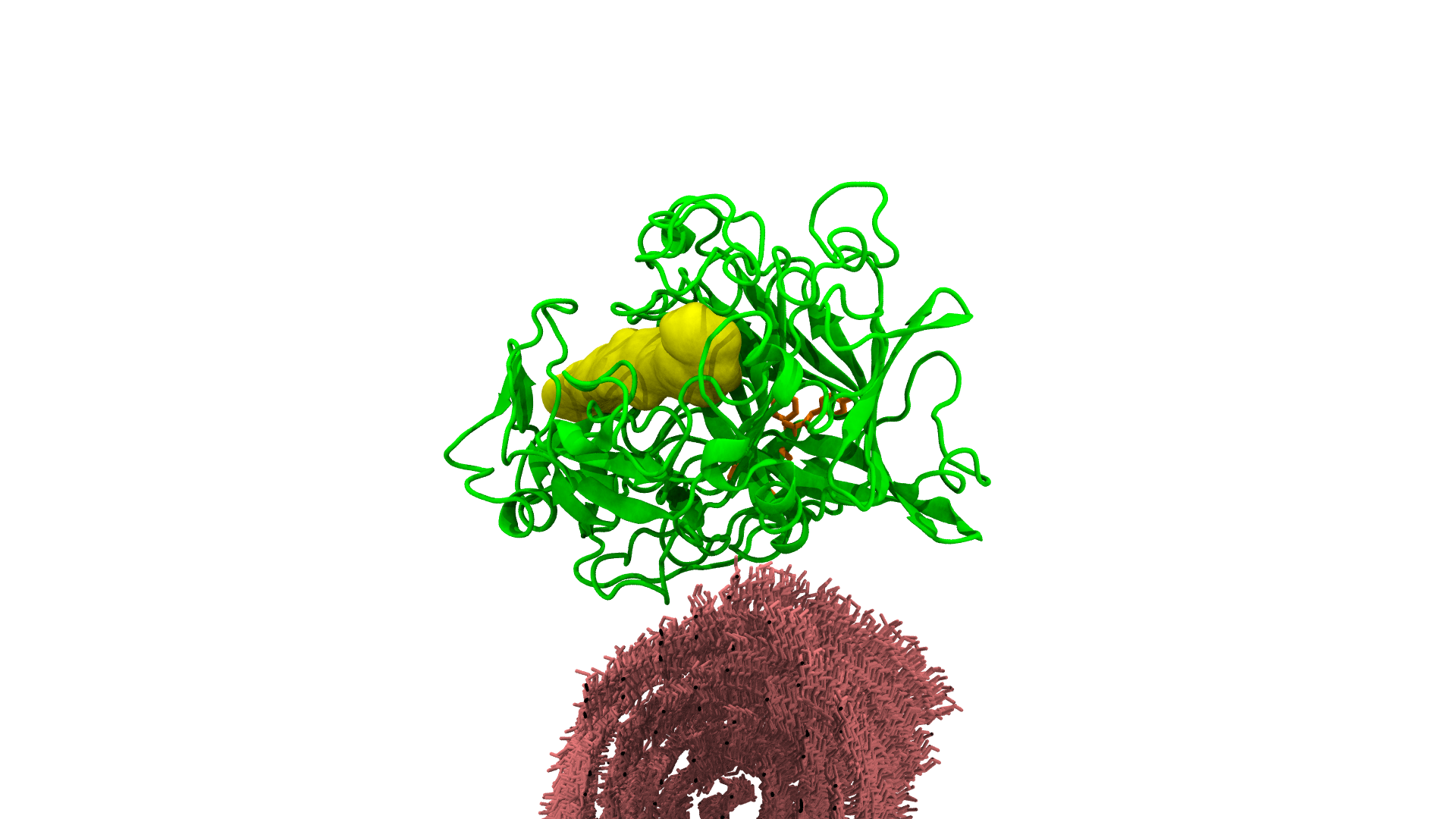

Supplement: Supplementary file 11 — 10.1186/s13068-015-0379-8 A zip archive containing a gallery of each of the cellulases that bound to cellulose in the context of their environment. Each image within the gallery is one snapshot taken from the end of the trajectory showing the relative position of each enzyme (green) that makes contact with the cellulose (red). Nearby lignins are shown in blue, and the substrate tunnel is a yellow surface to orient the viewer. The three tyrosine residues are shown in orange. Note that for each protein, there are 4 images, taken from different relative orientations to the cellulose fibril (0, 90, 180, and 270), and are labeled accordingly in their filenames. [file 13068_2015_379_MOESM11_ESM.zip › gallery/C-6_P-31_270.png]

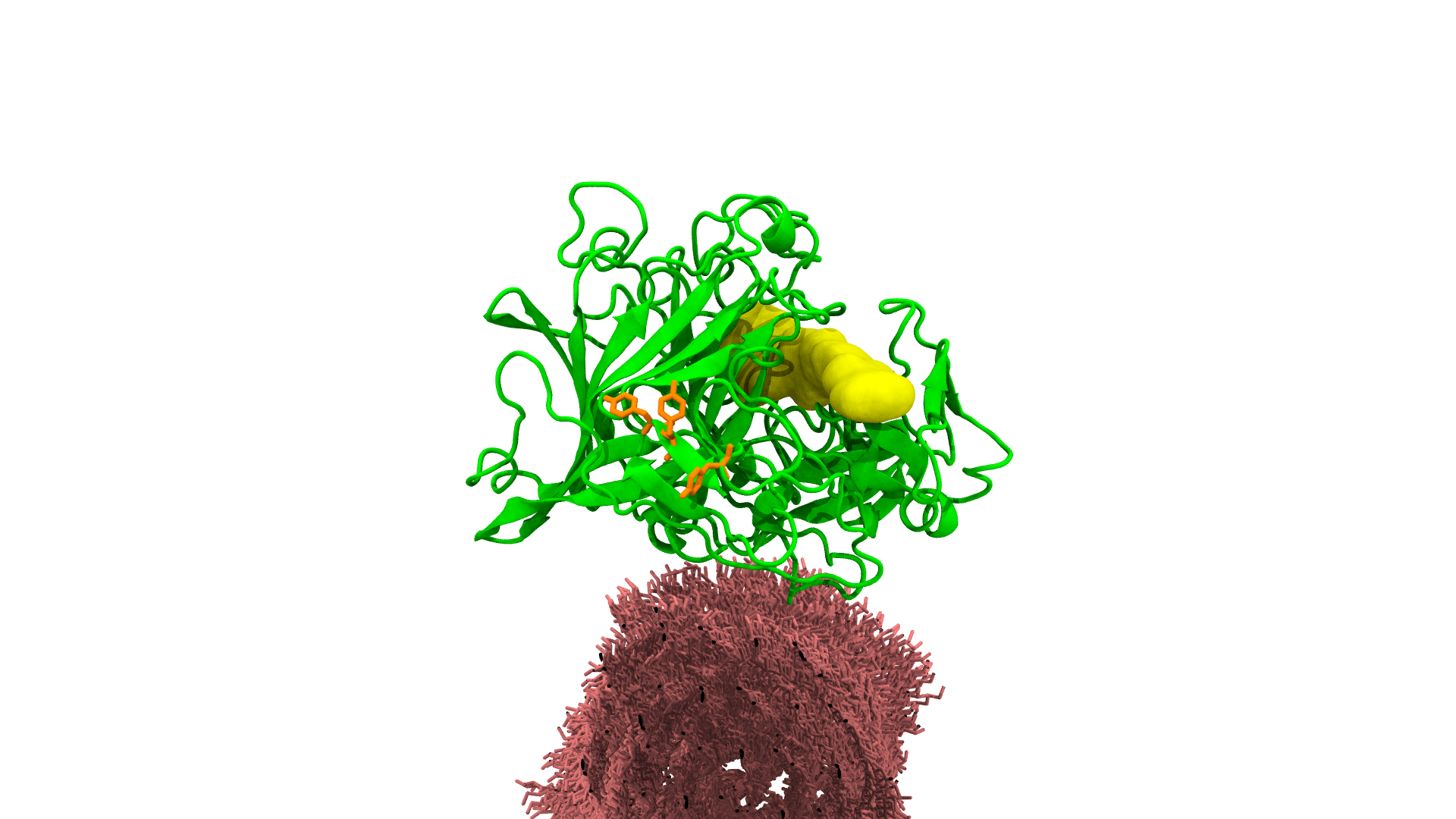

Supplement: Supplementary file 11 — 10.1186/s13068-015-0379-8 A zip archive containing a gallery of each of the cellulases that bound to cellulose in the context of their environment. Each image within the gallery is one snapshot taken from the end of the trajectory showing the relative position of each enzyme (green) that makes contact with the cellulose (red). Nearby lignins are shown in blue, and the substrate tunnel is a yellow surface to orient the viewer. The three tyrosine residues are shown in orange. Note that for each protein, there are 4 images, taken from different relative orientations to the cellulose fibril (0, 90, 180, and 270), and are labeled accordingly in their filenames. [file 13068_2015_379_MOESM11_ESM.zip › gallery/C-6_P-31_90.png]

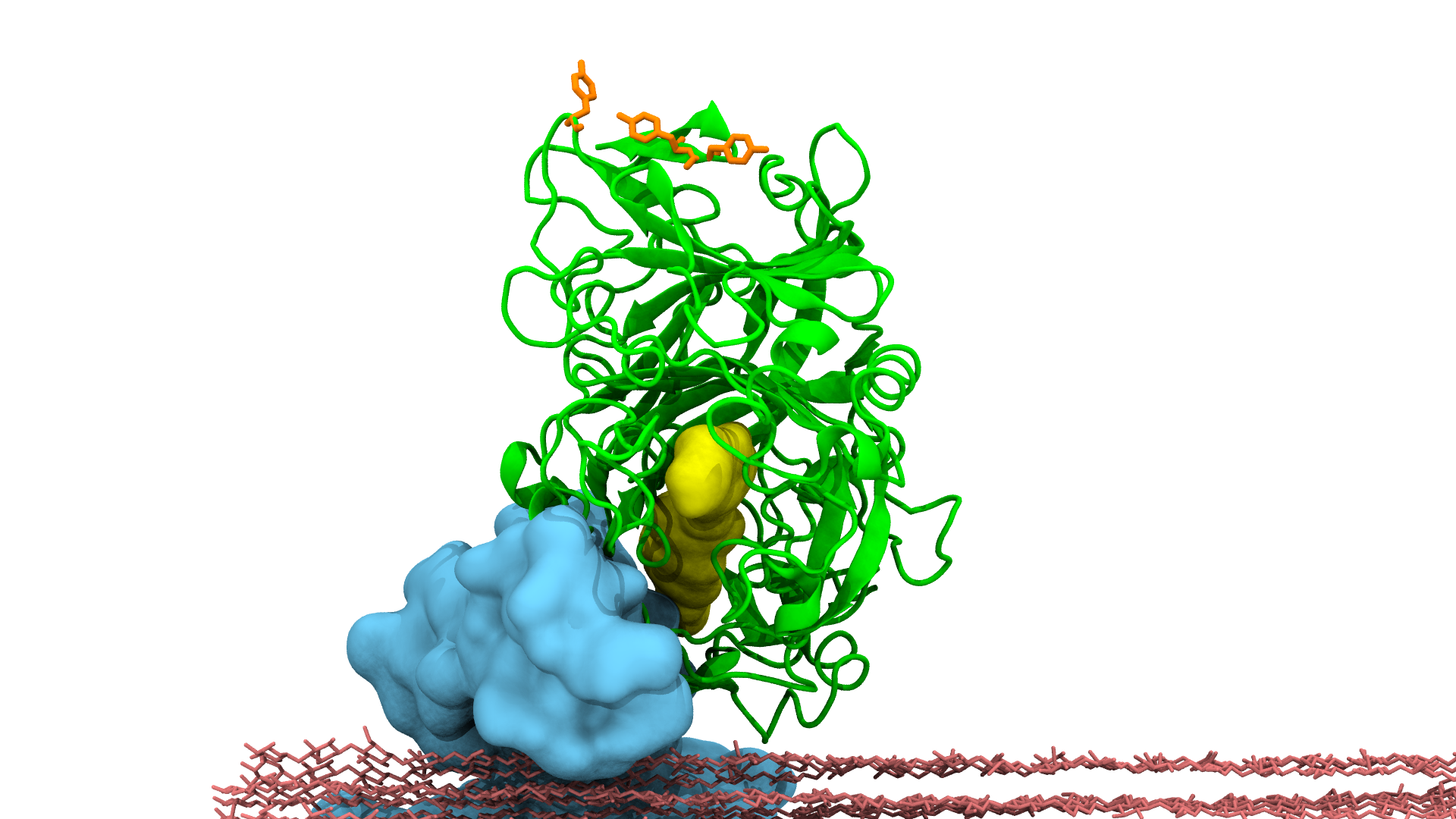

Supplement: Supplementary file 11 — 10.1186/s13068-015-0379-8 A zip archive containing a gallery of each of the cellulases that bound to cellulose in the context of their environment. Each image within the gallery is one snapshot taken from the end of the trajectory showing the relative position of each enzyme (green) that makes contact with the cellulose (red). Nearby lignins are shown in blue, and the substrate tunnel is a yellow surface to orient the viewer. The three tyrosine residues are shown in orange. Note that for each protein, there are 4 images, taken from different relative orientations to the cellulose fibril (0, 90, 180, and 270), and are labeled accordingly in their filenames. [file 13068_2015_379_MOESM11_ESM.zip › gallery/C-6_P-38_0.png]

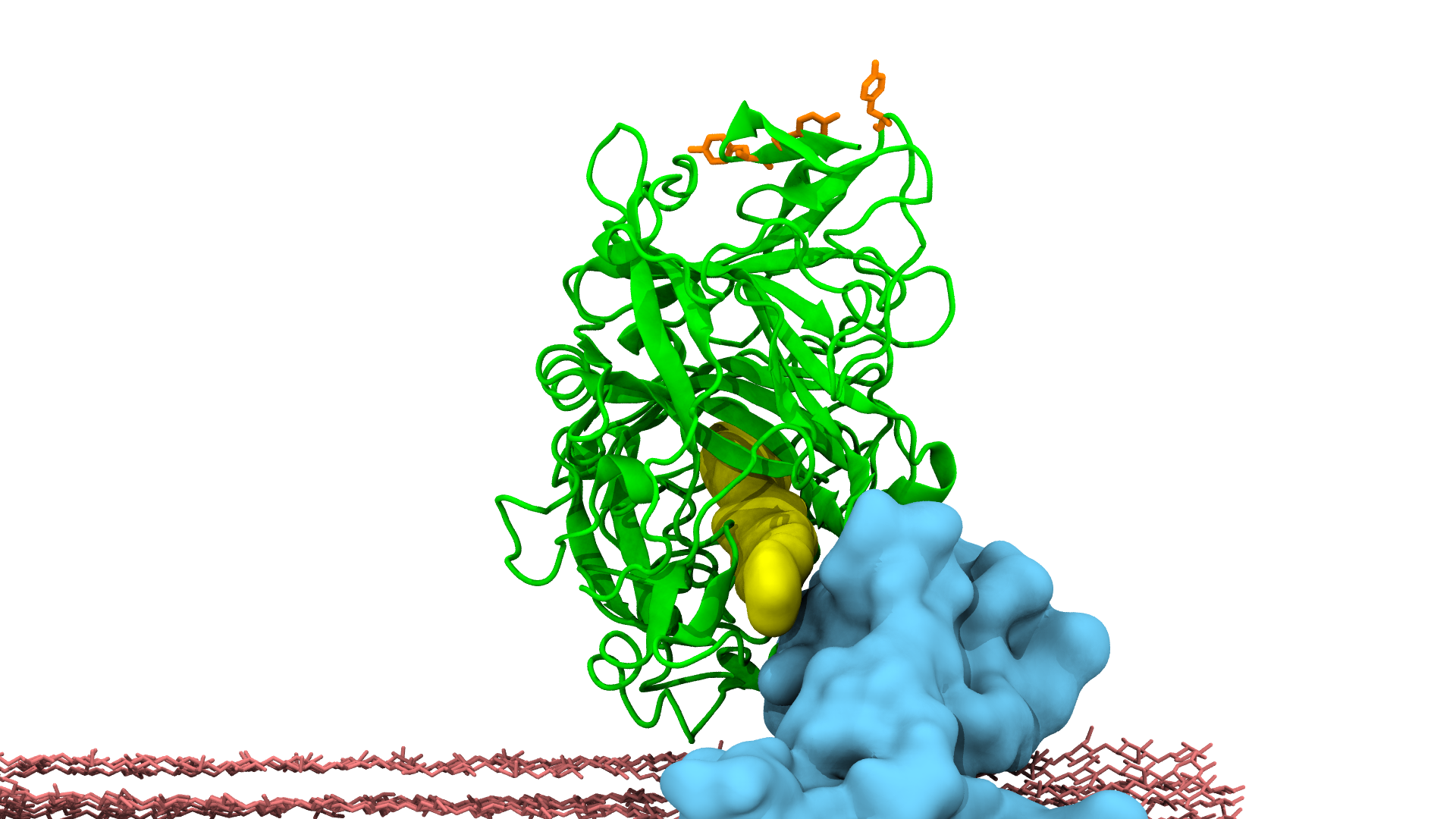

Supplement: Supplementary file 11 — 10.1186/s13068-015-0379-8 A zip archive containing a gallery of each of the cellulases that bound to cellulose in the context of their environment. Each image within the gallery is one snapshot taken from the end of the trajectory showing the relative position of each enzyme (green) that makes contact with the cellulose (red). Nearby lignins are shown in blue, and the substrate tunnel is a yellow surface to orient the viewer. The three tyrosine residues are shown in orange. Note that for each protein, there are 4 images, taken from different relative orientations to the cellulose fibril (0, 90, 180, and 270), and are labeled accordingly in their filenames. [file 13068_2015_379_MOESM11_ESM.zip › gallery/C-6_P-38_180.png]

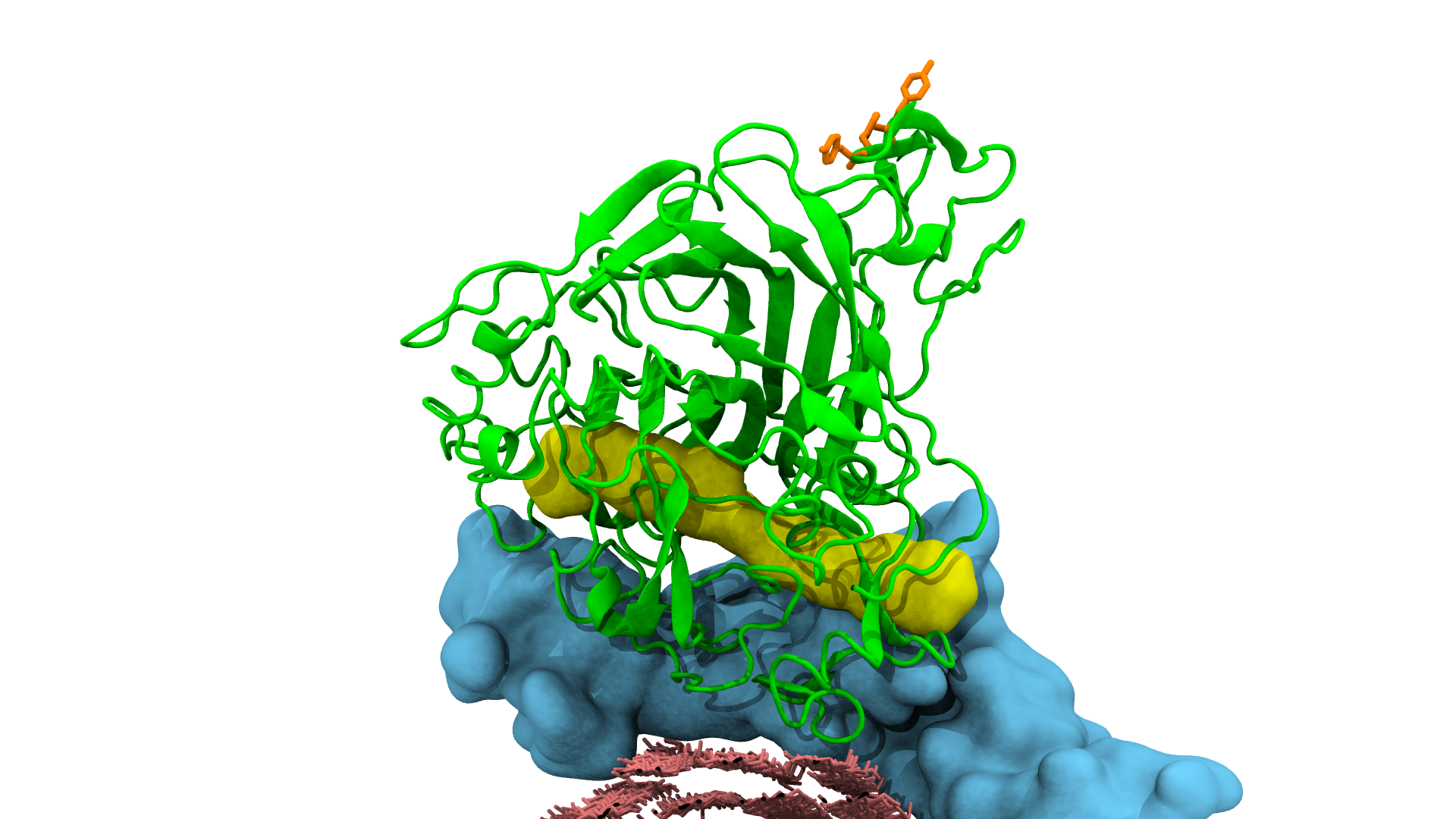

Supplement: Supplementary file 11 — 10.1186/s13068-015-0379-8 A zip archive containing a gallery of each of the cellulases that bound to cellulose in the context of their environment. Each image within the gallery is one snapshot taken from the end of the trajectory showing the relative position of each enzyme (green) that makes contact with the cellulose (red). Nearby lignins are shown in blue, and the substrate tunnel is a yellow surface to orient the viewer. The three tyrosine residues are shown in orange. Note that for each protein, there are 4 images, taken from different relative orientations to the cellulose fibril (0, 90, 180, and 270), and are labeled accordingly in their filenames. [file 13068_2015_379_MOESM11_ESM.zip › gallery/C-6_P-38_270.png]

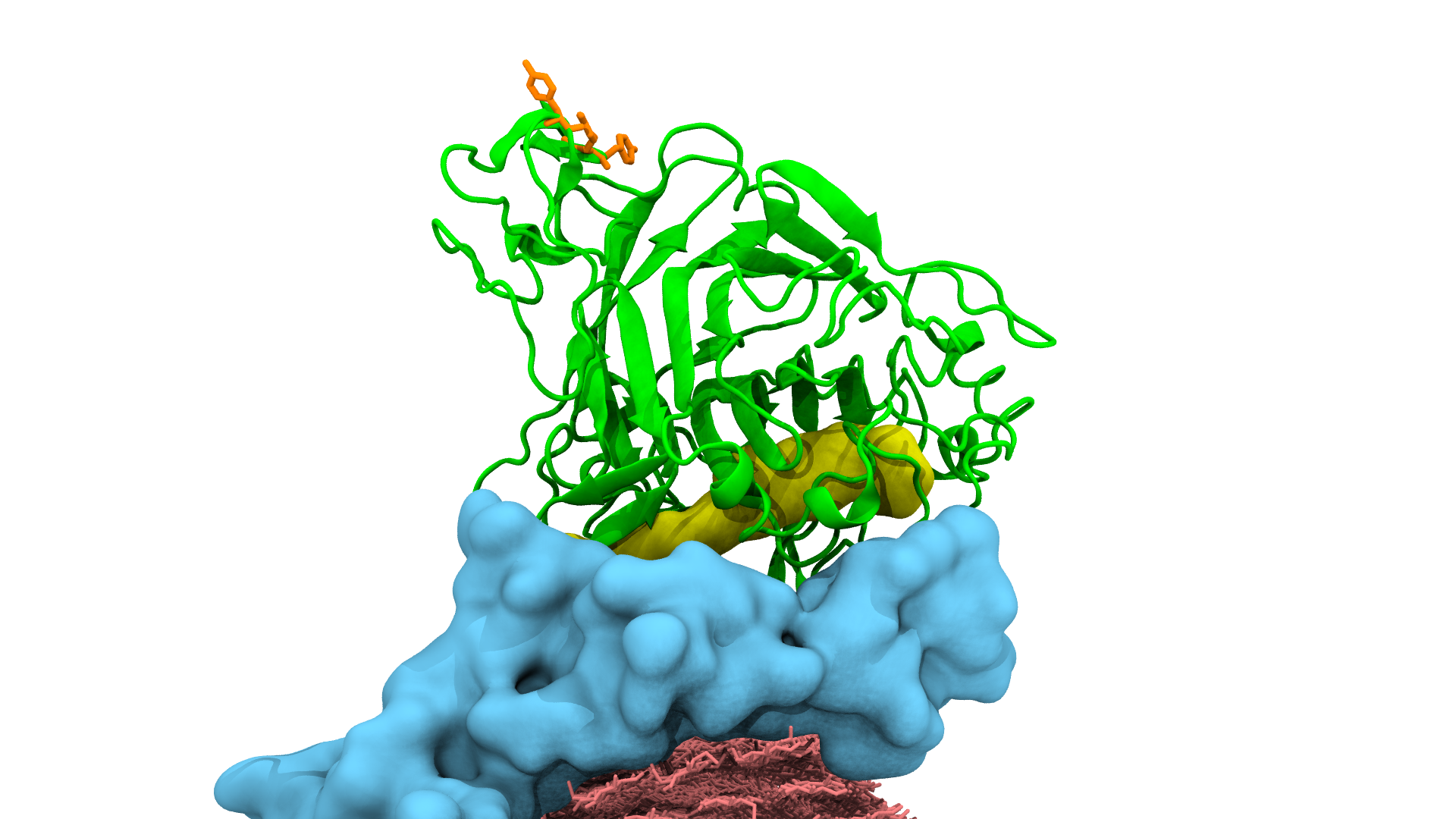

Supplement: Supplementary file 11 — 10.1186/s13068-015-0379-8 A zip archive containing a gallery of each of the cellulases that bound to cellulose in the context of their environment. Each image within the gallery is one snapshot taken from the end of the trajectory showing the relative position of each enzyme (green) that makes contact with the cellulose (red). Nearby lignins are shown in blue, and the substrate tunnel is a yellow surface to orient the viewer. The three tyrosine residues are shown in orange. Note that for each protein, there are 4 images, taken from different relative orientations to the cellulose fibril (0, 90, 180, and 270), and are labeled accordingly in their filenames. [file 13068_2015_379_MOESM11_ESM.zip › gallery/C-6_P-38_90.png]
